# Mood stabilizers and/or antipsychotics for bipolar disorder in the maintenance phase: a systematic review and network meta-analysis of randomized controlled trials

**DOI:** 10.1038/s41380-020-00946-6

**Published:** 2020-11-11

**Authors:** Taro Kishi, Toshikazu Ikuta, Yuki Matsuda, Kenji Sakuma, Makoto Okuya, Kazuo Mishima, Nakao Iwata

**Affiliations:** 1 Department of Psychiatry, Fujita Health University School of Medicine, Toyoake, Aichi 470-1192 Japan; 2 Department of Communication Sciences and Disorders, School of Applied Sciences, University of Mississippi, Oxford, MS 38677 USA; 3 Department of Psychiatry, Jikei University School of Medicine, Minato-ku, Tokyo 105-8461 Japan; 4 Department of Neuropsychiatry, Akita University Graduate School of Medicine, Akita, 010-8543 Japan

**Keywords:** Bipolar disorder, Drug discovery

## Abstract

We searched Embase, PubMed, and CENTRAL from inception until 22 May 2020 to investigate which antipsychotics and/or mood stabilizers are better for patients with bipolar disorder in the maintenance phase. We performed two categorical network meta-analyses. The first included monotherapy studies and studies in which the two drugs used were specified (i.e., aripiprazole, aripiprazole once monthly, aripiprazole+lamotrigine, aripiprazole+valproate, asenapine, carbamazepine, lamotrigine, lamotrigine+valproate, lithium, lithium+oxcarbazepine, lithium+valproate, olanzapine, paliperidone, quetiapine, risperidone long-acting injection, valproate, and placebo). The second included studies on second-generation antipsychotic combination therapies (SGAs) (i.e., aripiprazole, lurasidone, olanzapine, quetiapine, and ziprasidone) with lithium or valproate (LIT/VAL) compared with placebo with LIT/VAL. Outcomes were recurrence/relapse rate of any mood episode (RR-any, primary), depressive episode (RR-dep) and manic/hypomanic/mixed episode (RR-mania), discontinuation, mortality, and individual adverse events. Risk ratios and 95% credible interval were calculated. Forty-one randomized controlled trials were identified (*n* = 9821; mean study duration, 70.5 ± 36.6 weeks; percent female, 54.1%; mean age, 40.7 years). All active treatments other than carbamazepine, lamotrigine+valproate (no data) and paliperidone outperformed the placebo for RR-any. Aripiprazole+valproate, lamotrigine, lamotrigine+valproate, lithium, olanzapine, and quetiapine outperformed placebo for RR-dep. All active treatments, other than aripiprazole+valproate, carbamazepine, lamotrigine, and lamotrigine+valproate, outperformed placebo for RR-mania. Asenapine, lithium, olanzapine, quetiapine, and valproate outperformed placebo for all-cause discontinuation. All SGAs+LIT/VALs other than olanzapine+LIT/VAL outperformed placebo+LIT/VAL for RR-any. Lurasidone+LIT/VAL and quetiapine+LIT/VAL outperformed placebo+LIT/VAL for RR-dep. Aripiprazole+LIT/VAL and quetiapine+LIT/VAL outperformed placebo+LIT/VAL for RR-mania. Lurasidone+LIT/VAL and quetiapine+LIT/VAL outperformed placebo+LIT/VAL for all-cause discontinuation. Treatment efficacy, tolerability, and safety profiles differed among treatments.

## Introduction

Bipolar disorder (BD) is a common chronic mental disorder and a major contributor to the global burden of disease, with a worldwide prevalence of ~1% [1–3]. Patients with BD repeatedly and irregularly present mania/hypomania or depression during their lifetimes, which can result in social and occupational disability [4].

Pharmacological treatments are among the primary treatments for BD [4, 5]. The most recent guidelines state that clinicians and patients should take the maintenance phase into account when selecting acute phase treatments [6]. A previous network meta-analysis (NMA) reported that, compared with placebo, lithium and quetiapine reduced the recurrence or relapse rate of any mood, depressive, or manic, hypomanic/mixed episodes [7]. Recently, aripiprazole once monthly (AOM) and asenapine were approved for the treatment of BD [8]. We performed a systematic review and NMA of the efficacy, tolerability, and safety of antipsychotics and/or mood stabilizers, and we conducted a risk-benefit analysis of each medication for patients with BD in the maintenance phase.

## Methods

This study was performed according to the Preferred Reporting Items for Systematic Reviews and Meta-Analyses guidelines (PRISMA Checklist) [9] and was registered on Open Science Framework (https://osf.io/h4nwp). The literature search, data extraction, and data input into spreadsheets for analysis were performed simultaneously and independently by at least two authors (TK, TI, YM, KS, and MO). The authors double-checked the accuracy of data transfer and calculations in the study.

### Search strategy and inclusion criteria

The information about the literature search is shown in Supplementary Fig. 1. Inclusion criteria were (1) randomized controlled trials (RCTs) of antipsychotics and/or mood stabilizers lasting at least 12 weeks; (2) studies including adult patients with any BD subtype in the maintenance phase; (3) studies including patients with any mood symptoms at recruitment; (4) open studies and those with any level of blinding; and (5) studies with/without an enrichment designs. Exclusion criteria were (1) studies with child/ adolescent patients with BD; (2) continuation studies which randomly assigned patients with acute symptoms to treatment groups; (3) monotherapy and/or combination therapy studies of antidepressants with mood stabilizers or antipsychotics.

### Data synthesis and outcome measures

The primary outcome was recurrence/relapse rate of any mood episode. Secondary outcomes were recurrence/relapse rate of depressive episodes, recurrence/relapse rate of manic/hypomanic/mixed episodes, all-cause discontinuation, and discontinuation rate due to adverse events. Other outcomes were mortality rate and incidence of individual adverse events. Divalproex was classified as part of the valproate group. Definitions of recurrence/relapses are shown in Supplementary Table 1.

### Data extraction

We analyzed the extracted data based on intention-to-treat or modified intention-to-treat principles. When data required for meta-analysis were missing in the articles, we searched for these data in published systematic review articles. Although we attempted to contact the original study investigators to obtain unpublished data, we did not succeed in obtaining these data from all of them.

### Meta-analysis methods

Based on the results of our literature search (Supplementary Fig. 1 and Supplementary Table 1), we planned to perform two categorical NMAs. The first included (1) placebo-controlled and head-to-head trials of monotherapy of antipsychotics and/or mood stabilizers, and (2) combination or augmentation studies in which the two drugs used were specified. The second NMA included studies in which second-generation antipsychotics (SGAs) combined with lithium or valproate (LIT/VAL) were compared with placebo-LIT/VAL. A Bayesian NMA based on random-effects models [10] was conducted using the netmeta package [11]. We fitted random-effects frequentist NMAs, in which we assumed a common random-effects standard deviation for all comparisons in the network. The risk ratio (RR) and 95% credible interval (95% CI) were calculated. The heterogeneity standard deviation was also calculated for all outcomes. The odds ratios and their 95% CIs were calculated for mortality rate and completed suicide rate because incidences of these outcomes were very rare (Supplementary Appendix 1.6–1.7). We assessed network heterogeneity using τ^2^ with the netmeta package. We conducted a statistical evaluation of consistency using the design-by-treatment test (globally) and the node-splitting approach or Separate Direct from Indirect Evidence test (locally). The Bayesian analyses also estimated rank probabilities (i.e., probability of each treatment obtaining each possible rank as shown by their relative effects). The surface under the cumulative ranking area was calculated to rank the interventions. We also performed a meta-regression analysis in the first NMA to examine whether some potentially confounding factors (e.g., publication year, duration of study, number of total patients, percent female, and mean age) were associated with the extent of effect on primary and secondary outcomes. In addition to the analyses conducted previously [7], we also performed sensitivity analyses for primary and secondary outcomes in the first NMA, in which we gave only half the weight to (1) studies that included both patients with bipolar disorder I (BDI) and with other BD (when focusing on studies including only patients with BDI); (2) studies that included rapid-cycling patients with BD (when focusing on studies including only non-rapid-cycling patients with BD because rapid-cycling BD is considered to be more difficult to stabilize than non-rapid-cycling BD); (3) non-double-blind studies (when focusing on double-blind studies); (4) study arms that were “enriched” (when focusing on nonenriched studies); and (5) study arms supported by industry sponsors (when focusing on non-industry sponsorship studies) [12]. We did not perform meta-regression and sensitivity analyses in the second NMA because only six studies were included. In addition, the methodological quality of the included articles was assessed according to the Cochrane Risk of Bias criteria [13]. Funnel plots were used to explore potential publication bias. Lastly, we incorporated results into the Confidence in Network Meta-Analysis (CINeMA) application to assess the credibility of findings from each NMA [14]. CINeMA grades the confidence in results of each treatment comparison as high, moderate, low, or very low.

## Results

### Study characteristics

A flow diagram of the literature search is shown in Supplementary Fig. 1. We eliminated 2724 articles based on a review of the abstract and/or title. A review of the full texts of the remaining 59 articles resulted in the elimination of a further 21 articles. This left 38 included in the analysis [15–52]. Three additional studies [53–55] were identified following a manual search through the reference lists of the previous review article [7]. No further studies were found in the clinical trial registers. Although two studies included antidepressant treatment arms [15, 28], these studies were included in the NMA because they had both lithium arm and placebo arm. Hence, 41 studies, including a total of 9821 patients, with mean study duration of 70.5 ± 36.6 weeks, were identified and included in this study. Characteristics of these studies are shown in Supplementary Table 1. The percent female was 54.1%, and the mean age was 40.7 years. Twenty-three studies included only patients with BDI. Just four studies included only patients who had depressive episodes at recruitment. Sixteen studies included patients with rapid-cycling BD and 25 studies used enrichment designs. One perphenazine study [51] and two risperidone long-acting injection (RISLAI) studies [43, 44] were not included in the NMA because no arms of the study connected to the treatment arms of other studies [51]. Detailed methodological quality analyses of the studies based on the Cochrane Risk of Bias criteria are presented in Supplementary Fig. 2. Three studies were open-label studies [27, 30, 43]. Twenty-nine studies were industry-sponsored studies. Supplementary Tables 2.1 and 2.2 show the results of primary outcome in the individual study included in our systematic review.

AOM, aripiprazole, aripiprazole+lamotrigine, aripiprazole+valproate, asenapine, carbamazepine, lamotrigine, lamotrigine+valproate, lithium, lithium+oxcarbazepine, lithium+valproate, olanzapine, paliperidone, quetiapine, RISLAI, valproate, and placebo arms were included in the first NMA (32 studies and 7113 patients). Aripiprazole, lurasidone, quetiapine, olanzapine, or ziprasidone combined with LIT/VAL and LIT/VAL arms were included in the second NMA (6 studies and 2498 patients).

### Results of the first network meta-analysis

Results of the first NMA are shown in Supplementary Appendix 1.1–1.17.

### Primary and secondary outcomes

**Fig. 1:**
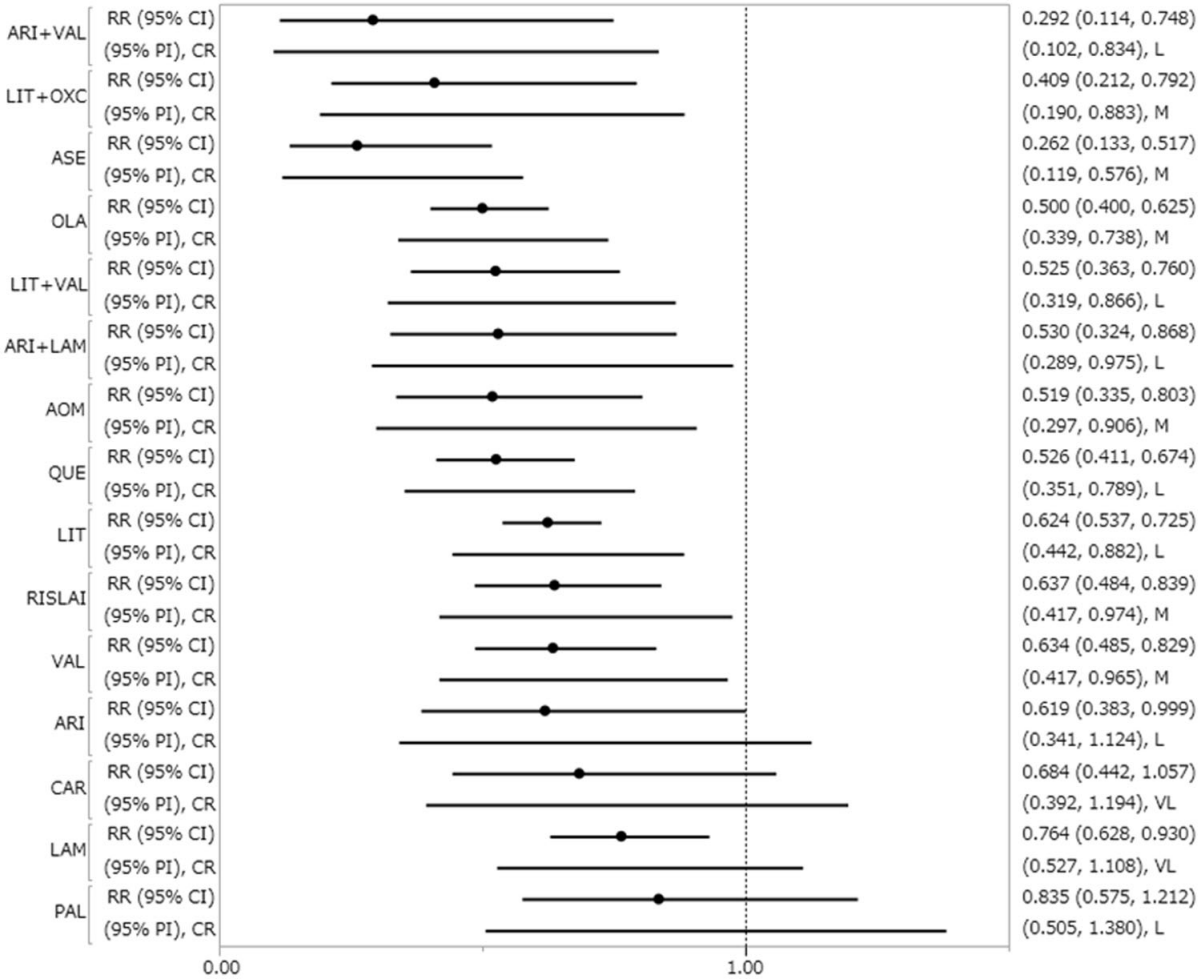
Recurrence/relapse rate of any mood episode. Drugs were compared with placebo. To visualize heterogeneity, we used prediction intervals in the forest plot. The confidence level estimated by CINeMA is shown next to 95% PI (L: low, M: moderate, VL: very low). 95% CI: 95% credible interval, 95% PI: prediction interval, CR: confidence rating, RR: risk ratio. AOM aripiprazole once monthly, ARI aripiprazole, ASE asenapine, CAR carbamazepine, LAM lamotrigine, LIT lithium, OLA olanzapine, OXC oxcarbazepine, PAL paliperidone, QUE quetiapine, RISLAI risperidone long-acting injectable, VAL valproate.

AOM, aripiprazole, aripiprazole+lamotrigine, aripiprazole+valproate, asenapine, lamotrigine, lithium, lithium+oxcarbazepine, lithium+valproate, olanzapine, quetiapine, RISLAI, and valproate outperformed placebo for recurrence/relapse rate of any mood episode (Table 1, Fig. 1). The RR (95% CI) for drugs that significantly lowered recurrence/relapse rates of any mood episode ranged from 0.262 (0.133–0.517) for asenapine to 0.764 (0.628–0.930) for lamotrigine (29 RCTs, 6890 patients; Table 1, Fig. 1). Asenapine outperformed aripiprazole, carbamazepine, lamotrigine, lithium, paliperidone, RISLAI, and valproate. Aripiprazole+valproate, olanzapine, and quetiapine outperformed lamotrigine and paliperidone (Table 1).

**Table 1:** Head-to-head comparisons for recurrence/relapse of any mood episode.

AOM	0.838 (0.438, 1.604)	0.978 (0.506, 1.888)	1.776 (0.630, 5.007)	1.980 (0.882, 4.443)	0.759 (0.409, 1.406)	0.679 (0.420, 1.096)	0.831 (0.524, 1.319)	1.267 (0.574, 2.794)	0.987 (0.557, 1.749)	1.037 (0.635, 1.692)	0.621 (0.350, 1.104)	0.986 (0.597, 1.628)	0.814 (0.486, 1.363)	0.818 (0.490, 1.366)	**0.519 (0.335, 0.803)**
	ARI	1.166 (0.587, 2.319)	2.118 (0.737, 6.086)	**2.362 (1.028, 5.428)**	0.905 (0.473, 1.730)	0.810 (0.482, 1.360)	0.991 (0.600, 1.638)	1.511 (0.669, 3.415)	1.178 (0.643, 2.157)	1.237 (0.729, 2.098)	0.741 (0.404, 1.361)	1.176 (0.685, 2.017)	0.971 (0.559, 1.687)	0.976 (0.563, 1.691)	**0.619 (0.383, 0.999)**
		ARI + LAM	1.816 (0.631, 5.226)	2.025 (0.875, 4.688)	0.776 (0.405, 1.485)	0.694 (0.442, 1.090)	0.850 (0.514, 1.407)	1.295 (0.573, 2.930)	1.010 (0.550, 1.853)	1.060 (0.620, 1.814)	0.635 (0.343, 1.179)	1.008 (0.582, 1.744)	0.832 (0.474, 1.461)	0.837 (0.481, 1.454)	**0.530 (0.324, 0.868)**
			ARI + VAL	1.115 (0.350, 3.558)	0.427 (0.154, 1.183)	**0.382 (0.147, 0.995)**	0.468 (0.184, 1.190)	0.713 (0.230, 2.214)	0.556 (0.212, 1.455)	0.584 (0.223, 1.525)	**0.350 (0.127, 0.962)**	0.555 (0.211, 1.461)	0.458 (0.172, 1.219)	0.461 (0.187, 1.134)	**0.292 (0.114, 0.748)**
				ASE	**0.383 (0.171, 0.859)**	**0.343 (0.169, 0.696)**	**0.420 (0.209, 0.842)**	0.640 (0.248, 1.649)	0.499 (0.230, 1.081)	0.523 (0.256, 1.071)	**0.314 (0.144, 0.682)**	0.498 (0.241, 1.026)	**0.411 (0.197, 0.856)**	**0.413 (0.199, 0.858)**	**0.262 (0.133, 0.517)**
					CAR	0.895 (0.561, 1.427)	1.095 (0.728, 1.649)	1.670 (0.780, 3.576)	1.301 (0.762, 2.221)	1.366 (0.850, 2.197)	0.819 (0.461, 1.454)	1.299 (0.796, 2.121)	1.073 (0.643, 1.791)	1.078 (0.671, 1.734)	0.684 (0.442, 1.057)
		0.694 (0.442, 1.090)				LAM	1.224 (0.978, 1.533)	1.866 (0.945, 3.685)	1.454 (0.968, 2.184)	**1.527 (1.141, 2.044)**	0.915 (0.600, 1.396)	**1.452 (1.063, 1.984)**	1.199 (0.856, 1.679)	1.205 (0.875, 1.659)	**0.764 (0.628, 0.930)**
					1.095 (0.728, 1.649)	1.101 (0.809, 1.499)	LIT	1.524 (0.802, 2.897)	1.188 (0.842, 1.676)	1.247 (0.980, 1.587)	0.748 (0.500, 1.118)	1.186 (0.905, 1.553)	0.979 (0.719, 1.333)	0.984 (0.773, 1.253)	**0.624 (0.537, 0.725)**
							1.524 (0.802, 2.897)	LIT + OXC	0.779 (0.376, 1.615)	0.818 (0.412, 1.625)	0.491 (0.230, 1.047)	0.778 (0.388, 1.562)	0.642 (0.315, 1.310)	0.646 (0.325, 1.283)	**0.409 (0.212, 0.792)**
							1.102 (0.761, 1.595)		LIT + VAL	1.050 (0.692, 1.592)	0.630 (0.372, 1.064)	0.999 (0.647, 1.540)	0.824 (0.522, 1.303)	0.829 (0.591, 1.163)	**0.525 (0.363, 0.760)**
							1.295 (0.877, 1.913)			OLA	**0.599 (0.388, 0.926)**	0.951 (0.685, 1.320)	0.785 (0.566, 1.089)	0.789 (0.566, 1.100)	**0.500 (0.400, 0.625)**
											PAL	**1.586 (1.013, 2.482)**	1.310 (0.824, 2.082)	1.317 (0.831, 2.085)	0.835 (0.575, 1.212)
							1.159 (0.792, 1.696)					QUE	0.826 (0.571, 1.194)	0.830 (0.583, 1.181)	**0.526 (0.411, 0.674)**
										**0.613 (0.382, 0.983)**			RISLAI	1.005 (0.687, 1.472)	**0.637 (0.484, 0.839)**
			0.461 (0.187, 1.134)				1.007 (0.781, 1.299)		0.776 (0.543, 1.111)					VAL	**0.634 (0.485, 0.829)**
**0.519 (0.335, 0.803)**	**0.619 (0.383, 0.999)**			**0.262 (0.133, 0.517)**		**0.795 (0.647, 0.976)**	**0.618 (0.525, 0.728)**			**0.521 (0.400, 0.680)**	0.835 (0.575, 1.212)	**0.508 (0.393, 0.657)**	**0.613 (0.462, 0.813)**	**0.628 (0.396, 0.997)**	PLA

Results from pairwise meta-analysis are presented in the left lower half and results from network meta-analysis in the upper right half.

The boldface result indicates statistical significance.

*AOM* aripiprazole once monthly, *ARI* aripiprazole, *ASE* asenapine, *CAR* carbamazepine, *LAM* lamotrigine, *LIT* lithium, *OLA* olanzapine, *OXC* oxcarbazepine, *PAL* paliperidone, *PLA* placebo, *QUE* quetiapine, *RISLAI* risperidone long-acting injectable, *VAL* valproate.

**Fig. 2:**
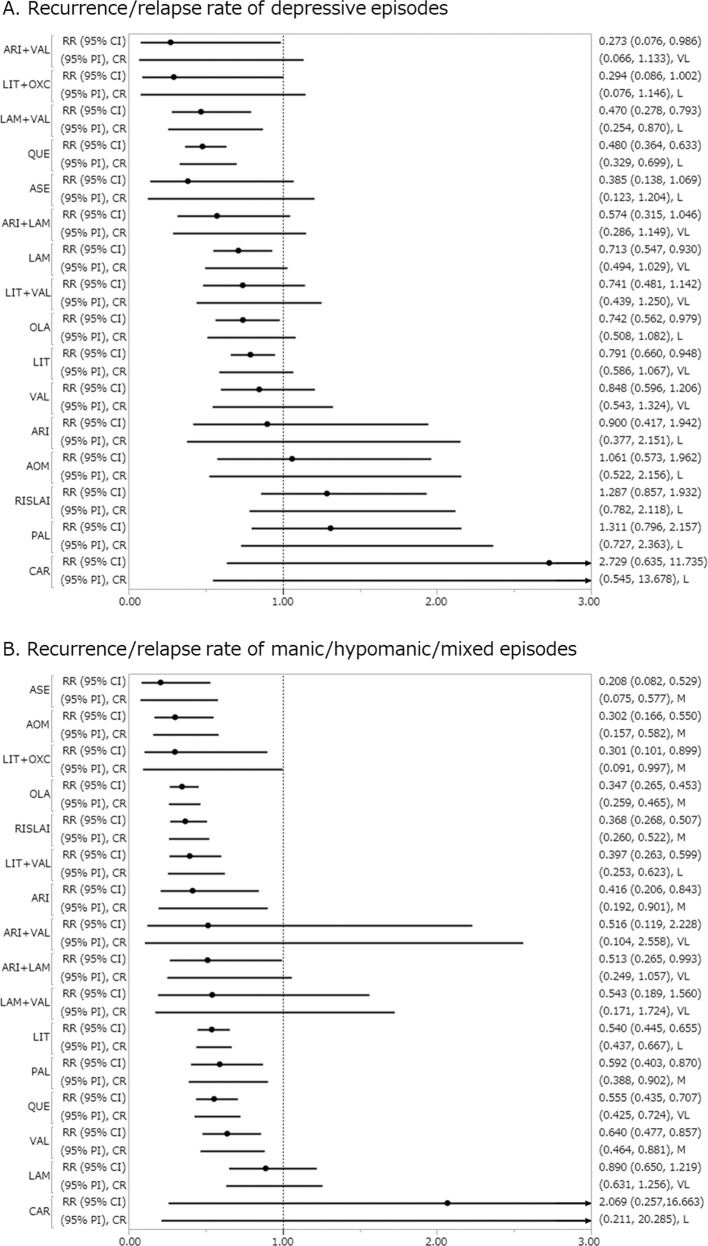
Recurrence/relapse rate of depressive episodes and manic/hypomanic/mixed episodes. **a** Recurrence/relapse rate of depressive episodes. **b** Recurrence/relapse rate of manic/hypomanic/mixed episodes. Drugs were compared with placebo. To visualize heterogeneity, we used prediction intervals in the forest plot. The confidence level estimated by CINeMA is shown next to 95% PI (L: low, M: moderate, VL: very low). 95% CI: 95% credible interval, 95% PI: prediction interval, CR confidence rating, RR risk ratio. AOM aripiprazole once monthly, ARI aripiprazole, ASE asenapine, CAR carbamazepine, LAM lamotrigine, LIT lithium, OLA olanzapine, OXC oxcarbazepine, PAL paliperidone, QUE quetiapine, RISLAI risperidone long-acting injectable, VAL valproate.

Aripiprazole+valproate, lamotrigine, lamotrigine+valproate, lithium, olanzapine, and quetiapine outperformed placebo for recurrence/relapse rate of depressive episodes, with RR (95% CI) ranging from 0.273 (0.076–0.986) for aripiprazole+valproate to 0.791 (0.660–0.948) for lithium (25 RCTs, 6438 patients; Fig. 2a). Aripiprazole+valproate outperformed carbamazepine, paliperidone, and RISLAI. Lamotrigine outperformed paliperidone and RISLAI. Lamotrigine+valproate outperformed AOM, carbamazepine, paliperidone, and RISLAI. Lithium and olanzapine outperformed RISLAI. Quetiapine outperformed AOM, carbamazepine, lamotrigine, lithium, olanzapine, paliperidone, RISLAI, and valproate.

All active treatments other than aripiprazole+valproate, carbamazepine, lamotrigine, and lamotrigine+valproate outperformed placebo for recurrence/relapse rate of manic/hypomanic/mixed episodes, with RR (95% CI) ranging from 0.208 (0.082–0.529) for asenapine to 0.640 (0.477–0.857) for valproate (25 RCTs, 6438 patients; Fig. 2b). AOM outperformed lamotrigine and valproate. Asenapine outperformed carbamazepine, lamotrigine, lithium, paliperidone, quetiapine, and valproate. Lithium outperformed lamotrigine. Lithium+valproate outperformed lamotrigine and valproate. Olanzapine outperformed lamotrigine, lithium, paliperidone, quetiapine, and valproate. Quetiapine outperformed lamotrigine. RISLAI outperformed lamotrigine, lithium, quetiapine, and valproate.

**Fig. 3:**
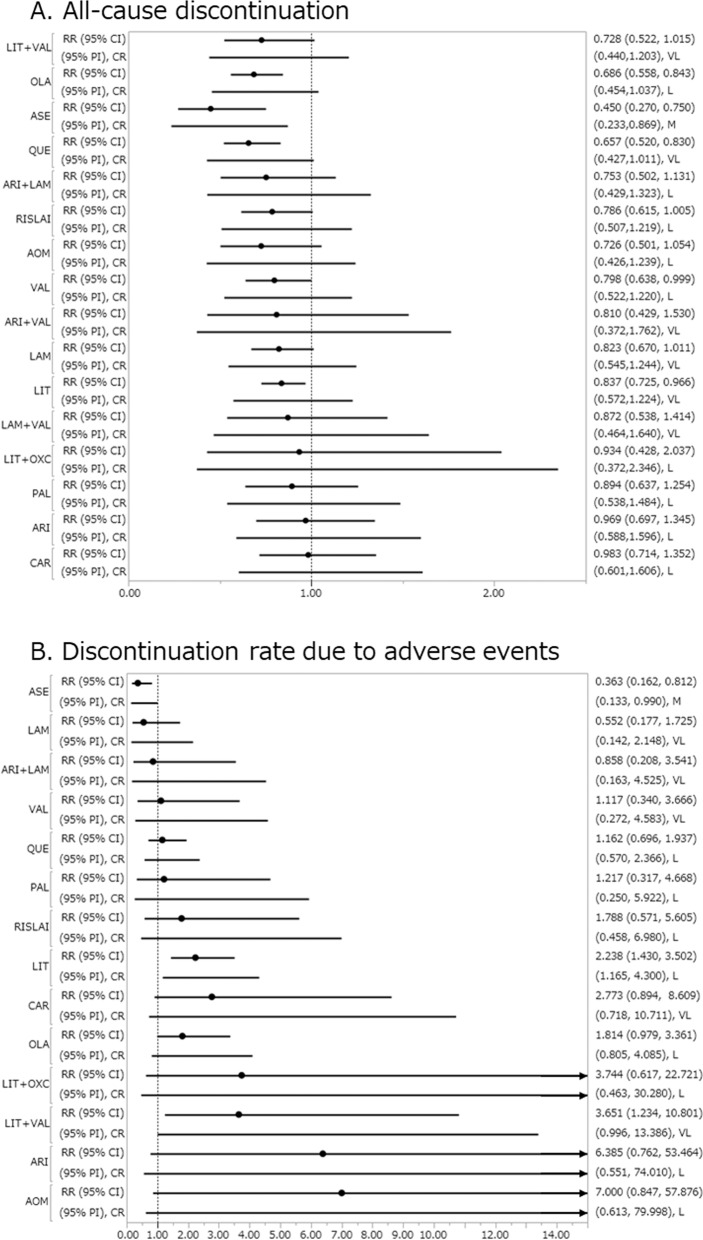
All-cause discontinuation and discontinuation rate due to adverse events. **a** All-cause discontinuation. **b** Discontinuation rate due to adverse events. Drugs were compared with placebo. To visualize heterogeneity, we used prediction intervals in the forest plot. The confidence level estimated by CINeMA is shown next to 95% PI (L: low, M: moderate, VL: very low). 95% CI: 95% credible interval, 95% PI: prediction interval, CR confidence rating, RR risk ratio. AOM aripiprazole once monthly, ARI aripiprazole, ASE asenapine, CAR carbamazepine, LAM lamotrigine, LIT lithium, OLA olanzapine, OXC oxcarbazepine, PAL paliperidone, QUE quetiapine, RISLAI risperidone long-acting injectable, VAL valproate.

Asenapine, lithium, olanzapine, quetiapine, and valproate were associated with lower all-cause discontinuation compared with placebo, with RR (95% CI) ranging from 0.450 (0.270–0.750) for asenapine to 0.837 (0.725–0.966) for lithium (29 RCTs, 6988 patients; Fig. 3a). Asenapine outperformed aripiprazole, carbamazepine, lamotrigine, lithium, paliperidone, and valproate. Quetiapine was outperformed by carbamazepine.

Only asenapine was associated with a lower discontinuation due to adverse events compared with placebo, with RR (95% CI) 0.363 (0.162–0.812) (21 RCTs, 6107 patients; Fig. 3b). Lithium and lithium+valproate were associated with higher discontinuation due to adverse events compared with placebo, with RR (95% CI) 2.238 (1.430–3.502) and 3.651 (1.234–10.801), respectively (21 RCTs, 6107 patients; Fig. 3b). Asenapine outperformed AOM, aripiprazole, carbamazepine, lithium, lithium+oxcarbazepine, lithium+valproate, olanzapine, quetiapine, and RISLAI. Lamotrigine outperformed AOM, aripiprazole, carbamazepine, lithium, and lithium+valproate. Quetiapine outperformed lithium. Valproate outperformed lithium+valproate.

Aripiprazole+valproate ranked first for reduction of the recurrence/relapse rate of any mood episode and depressive episodes. Asenapine was selected the best drug for reducing manic/hypomanic/mixed episodes and discontinuation due to adverse events. Lithium+valproate had the least incidence of all-cause discontinuation. Supplementary Appendix 2.1–2.3 shows two-dimensional graphs of the primary and secondary outcomes.

### Meta-regression analysis of primary and secondary efficacy outcomes

A significant association between the extent of effect on the recurrence/relapse rate of manic/hypomanic/mixed episodes and the duration of study was detected (beta = –0.497; 95% CI = –0.985, –0.004; *p* < 0.001). The heterogeneity variance of the meta-regression analysis was reduced by 21% compared with the unadjusted analysis. Although the unadjusted analysis demonstrated that aripiprazole, aripiprazole+lamotrigine, and paliperidone outperformed placebo in the recurrence/relapse rate of manic/hypomanic/mixed episodes, these differences were not statistically significant in the meta-regression analysis. We did not find any associations between the extent of effect in primary and other secondary outcomes and potentially confounding factors (Supplementary Appendix 1.1–1.5).

### Sensitivity analyses for primary and secondary outcomes

Relative reduction in heterogeneity variance for recurrence/relapse of any mood episodes for sensitivity analyses focusing on studies including only non-rapid-cycling patients with BD, nonenriched studies, and those not sponsored by industry were 29%, 21%, and 29%, respectively (Supplementary Appendix 1.1). Although outcomes with aripiprazole and aripiprazole+valproate were superior to placebo in the unadjusted analysis, the results did not reach statistical significance in the sensitivity analyses. The results of other comparisons for this outcome in the unadjusted and sensitivity analyses were similar. We did not detect relative reductions in heterogeneity variance for other outcomes in any of the sensitivity analyses (Supplementary Appendix 1.2–1.5).

### Mortality rate and incidence of individual adverse events

Mortality and completed suicide rates were low and similar for all treatments. Aripiprazole was associated with a higher incidence of extrapyramidal symptoms/use of anticholinergic agents compared with carbamazepine. Lithium was associated with a higher incidence of extrapyramidal symptoms/use of anticholinergic agents compared with placebo, carbamazepine, lamotrigine, olanzapine, and quetiapine. Valproate was associated with a higher incidence of extrapyramidal symptoms/use of anticholinergic agents compared with placebo, carbamazepine, lamotrigine, and quetiapine. Olanzapine was associated with a higher incidence of somnolence compared with placebo, lamotrigine, and lithium. Olanzapine and quetiapine were associated with a lower incidence of insomnia compared with placebo, lamotrigine, and lithium. RISLAI was associated with a higher incidence of prolactin-related adverse events compared with placebo. Lithium was associated with a higher incidence of dry mouth compared with valproate, and quetiapine was associated with a higher incidence of dry mouth compared with placebo and valproate. Lamotrigine, lithium, olanzapine, quetiapine, valproate, and placebo were associated with a higher incidence of headache compared with RISLAI. Valproate was associated with a higher incidence of headache compared with AOM. Lamotrigine was associated with a higher incidence of nausea compared with quetiapine. Lithium was associated with a higher incidence of nausea compared with placebo, olanzapine, and quetiapine. Valproate was associated with a higher incidence of nausea compared with placebo and quetiapine. Lithium was associated with a higher incidence of diarrhea compared with placebo and lamotrigine.

### Heterogeneity, inconsistency, and results of the first network meta-analysis graded using the CINeMA system

Global heterogeneity was low to moderate for most outcomes other than insomnia, dry mouth, and increased weight (Supplementary Appendix 1.1–1.17). We also did not detect considerable heterogeneities for most of the outcomes in certain comparisons (Supplementary Appendix 1.1–1.17). We did not find significant global inconsistencies in the primary and secondary outcomes. Percent inconsistency loops in the recurrence/relapse of any mood episode, depressive episodes, manic/hypomanic/mixed episodes, all-cause discontinuation, and discontinuation due to adverse events were: 0%, 13.6%, 9.1%, 0%, and 0%, respectively. However, we detected global inconsistency in insomnia and increased weight. We did not analyze global inconsistencies in prolactin-related adverse events and dry mouth due to insufficient data. Funnel plots with fewer than ten studies might not be meaningful. The confidence in evidence was often low or very low.

### Results of the second network meta-analysis

Results of the second NMA are shown in Supplementary Appendix 3.1–3.11. Aripiprazole+LIT/VAL, lurasidone+LIT/VAL, quetiapine+LIT/VAL, and ziprasidone+LIT/VAL were superior to placebo+LIT/VAL in the recurrence/relapse rate of any mood episode. Moreover, lurasidone+LIT/VAL and quetiapine+LIT/VAL were superior to olanzapine+LIT/VAL. Lurasidone+LIT/VAL and quetiapine+LIT/VAL were superior to placebo+LIT/VAL in the recurrence/relapse rate of depressive episodes, and lurasidone+LIT/VAL and quetiapine+LIT/VAL were superior to aripiprazole+LIT/VAL and ziprasidone+LIT/VAL. Aripiprazole+LIT/VAL and quetiapine+LIT/VAL were superior to placebo+LIT/VAL in the recurrence/relapse rate of manic/hypomanic/mixed episodes, and lurasidone+LIT/VAL and quetiapine+LIT/VAL were associated with lower all-cause discontinuation compared with placebo+LIT/VAL. Quetiapine+LIT/VAL was associated with a higher incidence of somnolence compared with placebo+LIT/VAL. Olanzapine+LIT/VAL and quetiapine+LIT/VAL were associated with a lower incidence of insomnia compared with placebo+LIT/VAL. Olanzapine+LIT/VAL and quetiapine+LIT/VAL were associated with a higher incidence of increased weight compared with placebo+LIT/VAL and aripiprazole+LIT/VAL. We did not examine local heterogeneity, and global and local inconsistency for any outcomes in the second NMA due to insufficient data. The confidence in evidence of the second NMA was very low.

## Discussion

We performed a systematic review and NMAs of efficacy, acceptability, tolerability, and safety for mono- or combination therapies using mood stabilizers and/or antipsychotics in the treatment of adult patients with BD in the maintenance phase. We extended a previous NMA by two SGAs (i.e., asenapine and AOM), by investigating many more adverse effects and by examining efficacy and safety of various combination therapies using SGA and LIT/VAL [7]. Overall, most of the mood stabilizers and/or antipsychotics reduced the recurrence/relapse rates of any mood episode. However, when examining individual mood symptoms, both drug types appeared to be more effective for treating mania than depression.

Aripiprazole+valproate was the best treatment for reducing the recurrence/relapse rates of any mood episode and depressive episodes. However, these significances disappeared during sensitivity analyses adjusting for enrichment design and sponsorship. Lithium+oxcarbazepine ranked high with respect to reducing the recurrence/relapse rates of any mood episode (2nd), depressive episodes (2nd), and manic/hypomanic/mixed episodes (3rd). Lamotrigine+valproate ranked third for reducing the recurrence/relapse rate of depressive episodes. However, these results were based on only one small study (<50 patients in each treatment arm). Lithium+valproate ranked first for all-cause discontinuation, based on the results of a single open-label study. We deemed the result inconclusive, given the CINeMA rating showed low and very low confidence levels for these treatments.

Asenapine ranked high with respect to reducing the recurrence/relapse rates of any mood episode (3rd), manic/hypomanic/mixed episodes (1st), all-cause discontinuation (3rd), and discontinuation due to adverse events (1st), which might represent novel insights into the pharmacological treatment of patients with BD in the maintenance phase. Although it did not prevent recurrence/relapse of depressive episodes, asenapine ranked fifth for outcome. It should be noted that this ranking was made from only one 26-week, double-blind, randomized, placebo-controlled trial of asenapine. Furthermore, asenapine carries the risk of oral hypoesthesia [34], and this distinctive side effect makes it difficult to blind [13]; the asenapine study might therefore be subject to performance and detection biases.

Olanzapine and quetiapine outperformed placebo in all efficacy outcomes and all-cause discontinuation. Quetiapine results should be interpreted with caution because all the quetiapine studies included in our meta-analysis used enrichment designs and were industry sponsored. However, sensitivity analyses adjusting for these factors demonstrated that quetiapine outperformed placebo in all efficacy outcomes. Thus, olanzapine and quetiapine showed good efficacy and acceptability in adult patients with BD in the maintenance phase. However, olanzapine and quetiapine carry a risk of somnolence and dry mouth, respectively. The second NMA demonstrated that combination therapies of these SGAs with LIT/VAL also carried the risk of increased weight.

Recent treatment guidelines recommend lithium as a first-line drug for the treatment of adult patients with BD in the maintenance phase [6, 56, 57]. The numbers of studies and patients treated with lithium were the largest among the active drugs included in our study (19 studies and 1335 patients). A recent meta-review including RCTs and non-RCTs reported that lithium had anti-suicidal effects for patients with psychiatric disorders including BD [58], although our meta-analysis did not show this effect. Our meta-analysis demonstrated that lithium outperformed placebo in all efficacy outcomes; however, it did not rank highly for the outcomes. Although lithium outperformed placebo regarding all-cause discontinuation, lithium increased discontinuation due to adverse events, and carried risks of extrapyramidal symptoms/use of anticholinergic agents, nausea, and diarrhea. However, given only 17 of 19 lithium studies included in our meta-analysis did not use enrichment designs, most patients assigned lithium included in our meta-analysis were not evaluated for efficacy, acceptability, tolerability, and safety of lithium prior to the assignment. However, sensitivity analysis of enrichment designs using the design-adjusted model demonstrated similar results to the unadjusted analysis. Accordingly, we concluded that lithium still had benefits for patients with BD in the maintenance phase, providing that due care is taken of its side effects.

A Finnish nationwide cohort of 18,018 patients with BD (mean follow-up time = 7.2 years) demonstrated that lithium and long-acting injectable (LAI) antipsychotics were effective in preventing hospitalization due to mental or physical illness compared with no drug use [59]. Unlike the results of our meta-analysis, the study indicated that lithium was superior to other mood stabilizers and that LAI antipsychotics are markedly better than identical oral formulations of antipsychotics. Quetiapine (most widely used in the study population) showed only an 8% risk reduction. Thus, there appear to be inconsistencies between the results of our meta-analysis, which included RCTs (providing the most robust evidence), and those of the cohort study (reflecting “real-world” routine clinical practice). We could not simply compare results between the studies for the following reasons [59, 60]. First, the study durations of RCTs are generally shorter than those of non-RCT studies. Second, the symptoms of trial populations are evaluated in more detail than those of patient populations in clinical practice. Hence, symptoms might be detected earlier, and earlier intervention given to trial populations than to patients in clinical practice. Third, because RCTs often have stringent inclusion and exclusion criteria (e.g., excluding patients with the most comorbidities and the highest severity of illness, such as suicidal ideation and suicidal attempt), trial populations are often not representative of those in clinical practice.

Our study has several limitations. First, the confidence in evidence of the first NMA was often low or very low. In the primary outcome, confidence levels were deemed to be low or very low in 90.8% of comparisons with placebo. Second, we did not perform the inconsistency test for dry mouth and prolactin-related adverse events for the first NMA and all outcomes for second NMA. Third, the range of study durations included in our meta-analysis was 17.3–171.4 weeks. Thus, the long-term efficacy and safety of drugs still need to be verified. Fourth, we did not cover important clinical issues that might inform treatment decision-making in routine clinical practice (e.g., combination with nonpharmacological treatments). Fifth, a cost-effectiveness analysis should be performed and included in the decision-making process.

In conclusion, our study represents the most comprehensive evidence currently available to guide the initial choice of pharmacological treatment for adult patients with BD in the maintenance phase. Clinicians and patients should consider the maintenance phase when selecting the treatment for the acute phase of BD.

## Supplementary information

APC

PRISMA 2009 checklist

31 supplementary appendices, 2 supplementary figures, and 2 supplementary tables

## References

[1] DSM–5. Diagnostic and Statistical Manual of Mental Disorders. American Psychiatric Association; 2013.

[2] Alonso J, Petukhova M, Vilagut G, Chatterji S, Heeringa S, Ustun TB (2011). Days out of role due to common physical and mental conditions: results from the WHO World Mental Health surveys. Mol Psychiatry.

[3] Ferrari AJ, Stockings E, Khoo JP, Erskine HE, Degenhardt L, Vos T (2016). The prevalence and burden of bipolar disorder: findings from the Global Burden of Disease Study 2013. Bipolar Disord.

[4] Grande I, Berk M, Birmaher B, Vieta E (2016). Bipolar disorder. Lancet.

[5] Baldessarini RJ, Tondo L, Vazquez GH (2019). Pharmacological treatment of adult bipolar disorder. Mol Psychiatry.

[6] Yatham LN, Kennedy SH, Parikh SV, Schaffer A, Bond DJ, Frey BN (2018). Canadian network for mood and anxiety treatments (CANMAT) and international society for bipolar disorders (ISBD) 2018 guidelines for the management of patients with bipolar disorder. Bipolar Disord.

[7] Miura T, Noma H, Furukawa TA, Mitsuyasu H, Tanaka S, Stockton S (2014). Comparative efficacy and tolerability of pharmacological treatments in the maintenance treatment of bipolar disorder: a systematic review and network meta-analysis. Lancet Psychiatry.

[8] FDA. U.S. Food and Drug Administration. 10.1080/15360280801989401 28792814

[9] Moher D, Liberati A, Tetzlaff J, Altman DG, Group P (2009). Preferred reporting items for systematic reviews and meta-analyses: the PRISMA statement. Bmj.

[10] DerSimonian R, Laird N (1986). Meta-analysis in clinical trials. Controlled Clin trials.

[11] Rücker G, Schwarzer G, Krahn U, König J. netmeta: network meta-analysis using frequentist methods (R package version 0.9-5). https://CRANR-projectorg/package=netmeta 2017; (accessed March 14, 2020).

[12] van Valkenhoef G, Lu G, de Brock B, Hillege H, Ades AE, Welton NJ (2012). Automating network meta-analysis. Res Synth Methods.

[13] Higgins J, Thomas J, Chandler J, Cumpston M, Li T, Page M, et al. Cochrane Handbook for Systematic Reviews of Interventions version 6.0. https://www.trainingcochraneorg/handbook 2019. 10.1002/14651858.ED000142 PMC10284251 31643080

[14] Salanti G, Del Giovane C, Chaimani A, Caldwell DM, Higgins JP (2014). Evaluating the quality of evidence from a network meta-analysis. PLoS ONE.

[15] Amsterdam JD, Shults J (2010). Efficacy and safety of long-term fluoxetine versus lithium monotherapy of bipolar II disorder: a randomized, double-blind, placebo-substitution study. Am J Psychiatry.

[16] Berwaerts J, Melkote R, Nuamah I, Lim P (2012). A randomized, placebo- and active-controlled study of paliperidone extended-release as maintenance treatment in patients with bipolar I disorder after an acute manic or mixed episode. J Affect Disord.

[17] Bowden CL, Calabrese JR, McElroy SL, Gyulai L, Wassef A, Petty F (2000). A randomized, placebo-controlled 12-month trial of divalproex and lithium in treatment of outpatients with bipolar I disorder. Divalproex Maintenance Study Group. Arch Gen Psychiatry.

[18] Bowden CL, Calabrese JR, Sachs G, Yatham LN, Asghar SA, Hompland M (2003). A placebo-controlled 18-month trial of lamotrigine and lithium maintenance treatment in recently manic or hypomanic patients with bipolar I disorder. Arch Gen Psychiatry.

[19] Calabrese JR, Bowden CL, Sachs G, Yatham LN, Behnke K, Mehtonen OP (2003). A placebo-controlled 18-month trial of lamotrigine and lithium maintenance treatment in recently depressed patients with bipolar I disorder. J Clin Psychiatry.

[20] Calabrese JR, Sanchez R, Jin N, Amatniek J, Cox K, Johnson B (2017). Efficacy and safety of aripiprazole once-monthly in the maintenance treatment of bipolar I disorder: a double-blind, placebo-controlled, 52-week randomized withdrawal study. J Clin Psychiatry.

[21] Calabrese JR, Shelton MD, Rapport DJ, Youngstrom EA, Jackson K, Bilali S (2005). A 20-month, double-blind, maintenance trial of lithium versus divalproex in rapid-cycling bipolar disorder. Am J Psychiatry.

[22] Calabrese JR, Suppes T, Bowden CL, Sachs GS, Swann AC, McElroy SL (2000). A double-blind, placebo-controlled, prophylaxis study of lamotrigine in rapid-cycling bipolar disorder. Lamictal 614 Study Group. J Clin Psychiatry.

[23] Coxhead N, Silverstone T, Cookson J (1992). Carbamazepine versus lithium in the prophylaxis of bipolar affective disorder. Acta Psychiatr Scand.

[24] Dunner DL, Stallone F, Fieve RR (1976). Lithium carbonate and affective disorders. V: a double-blind study of prophylaxis of depression in bipolar illness. Arch Gen Psychiatry.

[25] Fieve RR, Kumbaraci T, Dunner DL (1976). Lithium prophylaxis of depression in bipolar I, bipolar II, and unipolar patients. Am J Psychiatry.

[26] Hartong EG, Moleman P, Hoogduin CA, Broekman TG, Nolen WA, LitCar G (2003). Prophylactic efficacy of lithium versus carbamazepine in treatment-naive bipolar patients. J Clin Psychiatry.

[27] Geddes JR, Goodwin GM, Rendell J, Azorin JM, Cipriani A, Ostacher MJ (2010). Lithium plus valproate combination therapy versus monotherapy for relapse prevention in bipolar I disorder (BALANCE): a randomised open-label trial. Lancet.

[28] Kane JM, Quitkin FM, Rifkin A, Ramos-Lorenzi JR, Nayak DD, Howard A (1982). Lithium carbonate and imipramine in the prophylaxis of unipolar and bipolar II illness: a prospective, placebo-controlled comparison. Arch Gen Psychiatry.

[29] Keck PE, Calabrese JR, McIntyre RS, McQuade RD, Carson WH, Eudicone JM (2007). Aripiprazole monotherapy for maintenance therapy in bipolar I disorder: a 100-week, double-blind study versus placebo. J Clin Psychiatry.

[30] Kleindienst N, Greil W (2000). Differential efficacy of lithium and carbamazepine in the prophylaxis of bipolar disorder: results of the MAP study. Neuropsychobiology.

[31] Prien RF, Caffey EM, Klett CJ (1973). Prophylactic efficacy of lithium carbonate in manic-depressive illness. Report of the Veterans Administration and National Institute of Mental Health collaborative study group. Arch Gen Psychiatry.

[32] Prien RF, Klett CJ, Caffey EM (1973). Lithium carbonate and imipramine in prevention of affective episodes. A comparison in recurrent affective illness. Arch Gen Psychiatry.

[33] Quiroz JA, Yatham LN, Palumbo JM, Karcher K, Kushner S, Kusumakar V (2010). Risperidone long-acting injectable monotherapy in the maintenance treatment of bipolar I disorder. Biol Psychiatry.

[34] Szegedi A, Durgam S, Mackle M, Yu SY, Wu X, Mathews M (2018). Randomized, double-blind, placebo-controlled trial of asenapine maintenance therapy in adults with an acute manic or mixed episode associated with Bipolar I disorder. Am J Psychiatry.

[35] Tohen M, Calabrese JR, Sachs GS, Banov MD, Detke HC, Risser R (2006). Randomized, placebo-controlled trial of olanzapine as maintenance therapy in patients with bipolar I disorder responding to acute treatment with olanzapine. Am J Psychiatry.

[36] Tohen M, Greil W, Calabrese JR, Sachs GS, Yatham LN, Oerlinghausen BM (2005). Olanzapine versus lithium in the maintenance treatment of bipolar disorder: a 12-month, randomized, double-blind, controlled clinical trial. Am J Psychiatry.

[37] Vieta E, Cruz N, Garcia-Campayo J, de Arce R, Manuel Crespo J, Valles V (2008). A double-blind, randomized, placebo-controlled prophylaxis trial of oxcarbazepine as adjunctive treatment to lithium in the long-term treatment of bipolar I and II disorder. Int J Neuropsychopharmacol.

[38] Vieta E, Montgomery S, Sulaiman AH, Cordoba R, Huberlant B, Martinez L (2012). A randomized, double-blind, placebo-controlled trial to assess prevention of mood episodes with risperidone long-acting injectable in patients with bipolar I disorder. Eur Neuropsychopharmacol.

[39] Vieta E, Suppes T, Eggens I, Persson I, Paulsson B, Brecher M (2008). Efficacy and safety of quetiapine in combination with lithium or divalproex for maintenance of patients with bipolar I disorder (international trial 126). J Affect Disord.

[40] Weisler RH, Nolen WA, Neijber A, Hellqvist A, Paulsson B (2011). Trial 144 Study I. Continuation of quetiapine versus switching to placebo or lithium for maintenance treatment of bipolar I disorder (Trial 144: a randomized controlled study). J Clin Psychiatry.

[41] Woo YS, Bahk WM, Chung MY, Kim DH, Yoon BH, Lee JH (2011). Aripiprazole plus divalproex for recently manic or mixed patients with bipolar I disorder: a 6-month, randomized, placebo-controlled, double-blind maintenance trial. Hum Psychopharmacol.

[42] Young AH, McElroy SL, Olausson B, Paulsson B, Embolden I (2014). Embolden III. A randomised, placebo-controlled 52-week trial of continued quetiapine treatment in recently depressed patients with bipolar I and bipolar II disorder. World J Biol Psychiatry.

[43] Yatham LN, Fallu A, Binder CE. A 6-month randomized open-label comparison of continuation of oral atypical antipsychotic therapy or switch to long acting injectable risperidone in patients with bipolar disorder. Acta Psychiatr Scand. 2007;116(suppl):50–6. 10.1111/j.1600-0447.2007.01059.x 17688463

[44] Macfadden W, Alphs L, Haskins JT, Turner N, Turkoz I, Bossie C (2009). A randomized, double-blind, placebo-controlled study of maintenance treatment with adjunctive risperidone long-acting therapy in patients with bipolar I disorder who relapse frequently. Bipolar Disord.

[45] Tohen M, Chengappa KN, Suppes T, Baker RW, Zarate CA, Bowden CL (2004). Relapse prevention in bipolar I disorder: 18-month comparison of olanzapine plus mood stabiliser v. mood stabiliser alone. Br J Psychiatry.

[46] Bowden CL, Vieta E, Ice KS, Schwartz JH, Wang PP, Versavel M (2010). Ziprasidone plus a mood stabilizer in subjects with bipolar I disorder: a 6-month, randomized, placebo-controlled, double-blind trial. J Clin Psychiatry.

[47] Calabrese JR, Pikalov A, Streicher C, Cucchiaro J, Mao Y, Loebel A (2017). Lurasidone in combination with lithium or valproate for the maintenance treatment of bipolar I disorder. Eur Neuropsychopharmacol.

[48] Marcus R, Khan A, Rollin L, Morris B, Timko K, Carson W (2011). Efficacy of aripiprazole adjunctive to lithium or valproate in the long-term treatment of patients with bipolar I disorder with an inadequate response to lithium or valproate monotherapy: a multicenter, double-blind, randomized study. Bipolar Disord.

[49] Suppes T, Vieta E, Liu S, Brecher M, Paulsson B, Trial I (2009). Maintenance treatment for patients with bipolar I disorder: results from a north american study of quetiapine in combination with lithium or divalproex (trial 127). Am J Psychiatry.

[50] Bowden CL, Singh V, Weisler R, Thompson P, Chang X, Quinones M (2012). Lamotrigine vs. lamotrigine plus divalproex in randomized, placebo-controlled maintenance treatment for bipolar depression. Acta Psychiatr Scand.

[51] Zarate CA, Tohen M (2004). Double-blind comparison of the continued use of antipsychotic treatment versus its discontinuation in remitted manic patients. Am J Psychiatry.

[52] Carlson BX, Ketter TA, Sun W, Timko K, McQuade RD, Sanchez R (2012). Aripiprazole in combination with lamotrigine for the long-term treatment of patients with bipolar I disorder (manic or mixed): a randomized, multicenter, double-blind study (CN138-392). Bipolar Disord.

[53] Cundall RL, Brooks PW, Murray LG (1972). A controlled evaluation of lithium prophylaxis in affective disorders. Psychol Med.

[54] Koyama T, Higuchi T, Yamawaki S, Kanba S, Terao T, Shinohara A (2011). Study SCA104779, an evaluation of BW430C (lamotrigine) versus placebo in the prevention of mood episodes in bipolar I disorder patients. Jpn J Clin Psychiatry.

[55] Melia PI (1970). Prophylactic lithium: a double-blind trial in recurrent affective disorders. Br J Psychiatry.

[56] Fountoulakis KN, Grunze H, Vieta E, Young A, Yatham L, Blier P (2017). The International College of Neuro-Psychopharmacology (CINP) Treatment Guidelines for Bipolar Disorder in Adults (CINP-BD-2017), Part 3: The Clinical Guidelines. Int J Neuropsychopharmacol.

[57] Goodwin GM, Haddad PM, Ferrier IN, Aronson JK, Barnes T, Cipriani A (2016). Evidence-based guidelines for treating bipolar disorder: Revised third edition recommendations from the British Association for Psychopharmacology. J Psychopharmacol.

[58] Smith KA, Cipriani A (2017). Lithium and suicide in mood disorders: updated meta-review of the scientific literature. Bipolar Disord.

[59] Lahteenvuo M, Tanskanen A, Taipale H, Hoti F, Vattulainen P, Vieta E (2018). Real-world effectiveness of pharmacologic treatments for the prevention of rehospitalization in a finnish nationwide cohort of patients with bipolar disorder. JAMA Psychiatry.

[60] Blonde L, Khunti K, Harris SB, Meizinger C, Skolnik NS (2018). Interpretation and impact of real-world clinical data for the practicing clinician. Adv Ther.

