# Supplementary material for: Mood stabilizers and/or antipsychotics for bipolar disorder in the maintenance phase: a systematic review and network meta-analysis of randomized controlled trials
Source: Mol Psychiatry. 2020 Nov 11;26(8):4146–57. doi: 10.1038/s41380-020-00946-6 (PMC8550938; doi:10.1038/s41380-020-00946-6)
Supplement: Supplementary file 3 — 31 supplementary appendices, 2 supplementary figures, and 2 supplementary tables [file 41380_2020_946_MOESM3_ESM.pdf]

Supplementary Figure 1. Flow diagram of literature search

We searched Embase, PubMed, and the Cochrane Central Register of Controlled Trials for studies published prior to May 22, 2020. The search terms included: (bipolar disorder OR mania OR manic OR hypomania OR hypo-mania OR rapid cycle OR rapid-cycle OR bipolar depression OR affective) AND (randomized OR random OR randomly) AND (depot OR decanoate OR enanthate OR long acting injectable OR microsphere OR once monthly OR palmitate OR pamoate OR valproic acid OR valproate OR divalproate OR divalproex OR carbamazepine OR oxcarbazepine OR risperidone OR olanzapine OR aripiprazole OR quetiapine OR perospirone OR ziprasidone OR clozapine OR amisulpride OR asenapine OR blonanserin OR clothiapine OR iloperidone OR lurasidone OR mosapramine OR paliperidone OR remoxipride OR sertindole OR sulpiride OR tiapride OR chlorpromazine OR thioridazine OR mesoridazine OR loxapine OR molindone OR perphenazine OR thiothixene OR trifluoperazine OR haloperidol OR fluphenazine OR droperidol OR zuclopenthixol OR pimozide OR flupenthixol OR prochlorperazine OR lithium OR lamotrigine) AND (placebo). No language restriction was applied to the literature search. The authors assessed the retrieved reports against the inclusion and exclusion criteria and selected those that were eligible. In addition, the reference lists of the included articles and review articles were manually searched for additional relevant published and unpublished research, including conference abstracts. We also searched the clinical trial registries [ClinicalTrials.gov (http://clinicaltrials.gov/) and the World Health Organization International Clinical Trials Registry Platform (http://www.who.int/ictrp/search/en/)] to ensure the set of RCTs was comprehensive and to minimize the influence of publication bias. Any discrepancies in selected articles were resolved by consensus in a meeting among the authors. Monotherapy and/or combination therapy studies of antidepressants with mood stabilizers or antipsychotics. Recent meta-analyses demonstrated that antidepressants might increase the risk of mood swings with no improvement in prophylaxis of new depressive episodes in patients with BD,[1] and a previous NMA reported that lithium plus imipramine might increase relapse/recurrence of manic episodes in BD type I.[2] Therefore, we excluded antidepressant studies from our meta-analysis.

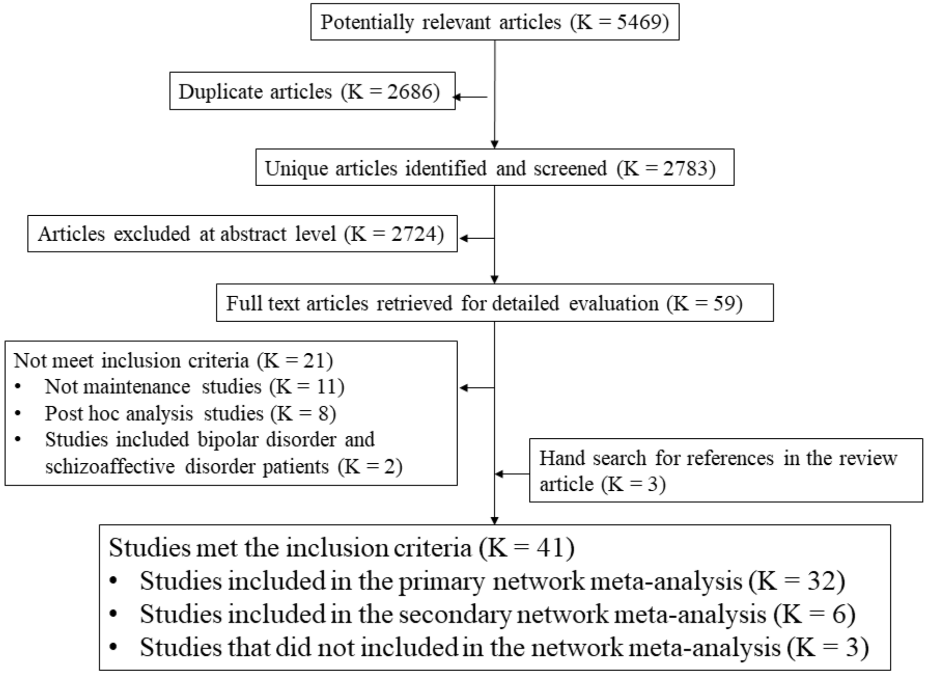

1. Liu B, et al., Efficacy and safety of long-term antidepressant treatment for bipolar disorders - A meta-analysis of randomized controlled trials. J Affect Disord 2017; 223: 41-48.

2. Miura T, et al., Comparative efficacy and tolerability of pharmacological treatments in the maintenance treatment of bipolar disorder: a systematic review and network meta-analysis. Lancet Psychiatry 2014; 1(5): 351-359.

Did not meet inclusion criteria (K = 21)

Ahlfors, U. G., Baastrup, P. C., Dencker, S. J., Elgen, K., Lingjaerde, O., Pedersen, V., Schou, M. & Aaskoven, O. (1981). Flupenthixol decanoate in recurrent manic-depressive illness. A comparison with lithium. Acta Psychiatr Scand 64, 226-37.

Altamura, A. C., Russo, M., Vismara, S. & Mundo, E. (2004). Comparative evaluation of olanzapine efficacy in the maintenance treatment of bipolar disorder. J Clin Psychopharmacol 24, 454-6.

Amsterdam, J. D., Lorenzo-Luaces, L., Soeller, I., Li, S. Q., Mao, J. J. & DeRubeis, R. J. (2015). Safety and effectiveness of continuation antidepressant versus mood stabilizer monotherapy for relapse-prevention of bipolar II depression: A randomized, double-blind, parallel-group, prospective study. J Affect Disord 185, 31-7.

Berk, M., Daglas, R., Dandash, O., Yucel, M., Henry, L., Hallam, K., Macneil, C., Hasty, M., Pantelis, C., Murphy, B. P., Kader, L., Damodaran, S., Wong, M. T. H., Conus, P., Ratheesh, A., McGorry, P. D. & Cotton, S. M. (2017). Quetiapine v. lithium in the maintenance phase following a first episode of mania: randomised controlled trial. Br J Psychiatry 210, 413-421.

Bobo, W. V., Epstein, R. A., Lynch, A., Patton, T. D., Bossaller, N. A. & Shelton, R. C. (2011). A randomized open comparison of long-acting injectable risperidone and treatment as usual for prevention of relapse, rehospitalization, and urgent care referral in community-treated patients with rapid cycling bipolar disorder. Clin Neuropharmacol 34, 224-33.

Brown, E., Dunner, D. L., McElroy, S. L., Keck, P. E., Adams, D. H., Degenhardt, E., Tohen, M. & Houston, J. P. (2009). Olanzapine/fluoxetine combination vs. lamotrigine in the 6-month treatment of bipolar I depression. Int J Neuropsychopharmacol 12, 773-82.

Chengappa, K. N., Turkin, S. R., Schlicht, P. J., Murphy, S. L., Brar, J. S., Fagiolini, A., Houck, P. R., Garbutt, R. G. & Fredrick, N. (2010). A Pilot, 15-month, randomised effectiveness trial of Risperidone long-acting injection (RLAI) versus oral atypical antipsychotic agents (AAP) in persons with bipolar disorder. Acta Neuropsychiatr 22, 68-80.

Daglas, R., Cotton, S. M., Allott, K., Yucel, M., Macneil, C. A., Hasty, M. K., Murphy, B., Pantelis, C., Hallam, K. T., Henry, L. P., Conus, P., Ratheesh, A., Kader, L., Wong, M. T., McGorry, P. D. & Berk, M. (2016). A single-blind, randomised controlled trial on the effects of lithium and quetiapine monotherapy on the trajectory of cognitive functioning in first episode mania: A 12-month follow-up study. Eur Psychiatry 31, 20-8.

Gao, K., Goto, T., Yuan, C., Brownrigg, B., Conroy, C., Chan, P. K., Serrano, M. B., Ganocy, S. J., Fang, F. & Calabrese, J. R. (2018). A Pilot Study of the Effectiveness of Lithium Versus Quetiapine Immediate Release Monotherapy in Patients With Bipolar Spectrum Disorders. J Clin Psychopharmacol 38, 422-434.

Goldberg, J. F., Bowden, C. L., Calabrese, J. R., Ketter, T. A., Dann, R. S., Frye, M. A., Suppes, T. & Post, R. M. (2008). Six-month prospective life charting of mood symptoms with lamotrigine monotherapy versus placebo in rapid cycling bipolar disorder. Biol Psychiatry 63, 125-30.

Greil, W., Ludwig-Mayerhofer, W., Erazo, N., Schochlin, C., Schmidt, S., Engel, R. R., Czernik, A., Giedke, H., Muller-Oerlinghausen, B., Osterheider, M., Rudolf, G. A., Sauer, H., Tegeler, J. & Wetterling, T. (1997). Lithium versus carbamazepine in the maintenance treatment of bipolar disorders--a randomised study. J Affect Disord 43, 151-61.

Juruena, M. F., Ottoni, G. L., Machado-Vieira, R., Carneiro, R. M., Weingarthner, N., Marquardt, A. R., Fleig, S. S., Broilo, L. & Busnello, E. A. (2009). Bipolar I and II disorder residual symptoms: oxcarbazepine and carbamazepine as add-on treatment to lithium in a double-blind, randomized trial. Prog Neuropsychopharmacol Biol Psychiatry 33, 94-9.

Kang, M. G., Qian, H., Keramatian, K., Chakrabarty, T., Saraf, G., Lam, R. W., Wong, H. & Yatham, L. N. (2020). Lithium vs valproate in the maintenance treatment of bipolar I disorder: A post- hoc analysis of a randomized double-blind placebo-controlled trial. Aust N Z J Psychiatry 54, 298-307.

Ketter, T. A., Houston, J. P., Adams, D. H., Risser, R. C., Meyers, A. L., Williamson, D. J. & Tohen, M. (2006). Differential efficacy of olanzapine and lithium in preventing manic or mixed recurrence in patients with bipolar I disorder based on number of previous manic or mixed episodes. J Clin Psychiatry 67, 95-101.

Licht, R. W., Nielsen, J. N., Gram, L. F., Vestergaard, P. & Bendz, H. (2010). Lamotrigine versus lithium as maintenance treatment in bipolar I disorder: an open, randomized effectiveness study mimicking clinical practice. The 6th trial of the Danish University Antidepressant Group (DUAG-6). Bipolar Disord 12, 483-93.

Maina, G., Albert, U., Rosso, G. & Bogetto, F. (2008). Olanzapine or lamotrigine addition to lithium in remitted bipolar disorder patients with anxiety disorder comorbidity: a randomized, single-blind, pilot study. J Clin Psychiatry 69, 609-16.

McIntyre, R. S., Cohen, M., Zhao, J., Alphs, L., Macek, T. A. & Panagides, J. (2010). Asenapine for long-term treatment of bipolar disorder: a double-blind 40-week extension study. J Affect Disord 126, 358-65.

Nierenberg, A. A., McElroy, S. L., Friedman, E. S., Ketter, T. A., Shelton, R. C., Deckersbach, T., McInnis, M. G., Bowden, C. L., Tohen, M., Kocsis, J. H., Calabrese, J. R., Kinrys, G., Bobo, W. V., Singh, V., Kamali, M., Kemp, D., Brody, B., Reilly-Harrington, N. A., Sylvia, L. G., Shesler, L. W., Bernstein, E. E., Schoenfeld, D., Rabideau, D. J., Leon, A. C., Faraone, S. & Thase, M. E. (2016). Bipolar CHOICE (Clinical Health Outcomes Initiative in Comparative Effectiveness): a pragmatic 6-month trial of lithium versus quetiapine for bipolar disorder. J Clin Psychiatry 77, 90-9.

Pae, C. U., Masand, P. S., Mandel, F. S. & O'Gorman, C. (2012). Achieving and sustaining remission in bipolar I disorder with ziprasidone : a post hoc analysis of a 24-week, double-blind, placebo-controlled study. Clin Drug Investig 32, 747-54.

Suppes, T., Marangell, L. B., Bernstein, I. H., Kelly, D. I., Fischer, E. G., Zboyan, H. A., Snow, D. E., Martinez, M., Al Jurdi, R., Shivakumar, G., Sureddi, S. & Gonzalez, R. (2008). A single blind comparison of lithium and lamotrigine for the treatment of bipolar II depression. J Affect Disord 111, 334-43.

Tohen, M., Ketter, T. A., Zarate, C. A., Suppes, T., Frye, M., Altshuler, L., Zajecka, J., Schuh, L. M., Risser, R. C., Brown, E. & Baker, R. W. (2003). Olanzapine versus divalproex sodium for the treatment of acute mania and maintenance of remission: a 47-week study. Am J Psychiatry 160, 1263-71.

<u>Hand search for reference in the review article* (K = 3)</u>

Cundall, R. L., Brooks, P. W. & Murray, L. G. (1972). A controlled evaluation of lithium prophylaxis in affective disorders. Psychol Med 2, 308-11.

Koyama, T., Higuchi, T., Yamawaki, S., Kanba, S., Terao, T. & Shinohara, A. (2011). Study SCA104779, an evaluation of BW430C (lamotrigine) versus placebo in the prevention of mood episodes in bipolar I disorder patients. Japanese Journal of Clinical Psychiatry 40, 369-383.

Melia, P. I. (1970). Prophylactic lithium: a double-blind trial in recurrent affective disorders. Br J Psychiatry 116, 621-4.

*Review article

Miura, T., Noma, H., Furukawa, T. A., Mitsuyasu, H., Tanaka, S., Stockton, S., Salanti, G., Motomura, K., Shimano-Katsuki, S., Leucht, S., Cipriani, A., Geddes, J. R. & Kanba, S. (2014). Comparative efficacy and tolerability of pharmacological treatments in the maintenance treatment of bipolar disorder: a systematic review and network meta-analysis. Lancet Psychiatry 1, 351-9.

Studies included in the primary network meta-analysis (K = 32)

Amsterdam, J. D. & Shults, J. (2010). Efficacy and safety of long-term fluoxetine versus lithium monotherapy of bipolar II disorder: a randomized, double-blind, placebo-substitution study. Am J Psychiatry 167, 792-800.

Berwaerts, J., Melkote, R., Nuamah, I. & Lim, P. (2012). A randomized, placebo- and active-controlled study of paliperidone extended-release as maintenance treatment in patients with bipolar I disorder after an acute manic or mixed episode. J Affect Disord 138, 247-58.

Bowden, C. L., Calabrese, J. R., McElroy, S. L., Gyulai, L., Wassef, A., Petty, F., Pope, H. G., Jr., Chou, J. C., Keck, P. E., Jr., Rhodes, L. J., Swann, A. C., Hirschfeld, R. M. & Wozniak, P. J. (2000). A randomized, placebo-controlled 12-month trial of divalproex and lithium in treatment of outpatients with bipolar I disorder. Divalproex Maintenance Study Group. Arch Gen Psychiatry 57, 481-9.

Bowden, C. L., Calabrese, J. R., Sachs, G., Yatham, L. N., Asghar, S. A., Hompland, M., Montgomery, P., Earl, N., Smoot, T. M., DeVeaugh-Geiss, J. & Lamictal 606 Study, G. (2003). A placebo-controlled 18-month trial of lamotrigine and lithium maintenance treatment in recently manic or hypomanic patients with bipolar I disorder. Arch Gen Psychiatry 60, 392-400.

Bowden CL, Singh V, Weisler R, Thompson P, Chang X, Quinones M, Mintz J. (2012). Lamotrigine vs. lamotrigine plus divalproex in randomized, placebo-controlled maintenance treatment for bipolar depression. Acta Psychiatr Scand. 126(5):342-50.

Calabrese, J. R., Bowden, C. L., Sachs, G., Yatham, L. N., Behnke, K., Mehtonen, O. P., Montgomery, P., Ascher, J., Paska, W., Earl, N., DeVeaugh-Geiss, J. & Lamictal 605 Study, G. (2003). A placebo-controlled 18-month trial of lamotrigine and lithium maintenance treatment in recently depressed patients with bipolar I disorder. J Clin Psychiatry 64, 1013-24.

Calabrese, J. R., Sanchez, R., Jin, N., Amatniek, J., Cox, K., Johnson, B., Perry, P., Hertel, P., Such, P., Salzman, P. M., McQuade, R. D., Nyilas, M. & Carson, W. H. (2017b). Efficacy and Safety of Aripiprazole Once-Monthly in the Maintenance Treatment of Bipolar I Disorder: A Double-Blind, Placebo-Controlled, 52-Week Randomized Withdrawal Study. J Clin Psychiatry 78, 324-331.

Calabrese, J. R., Shelton, M. D., Rapport, D. J., Youngstrom, E. A., Jackson, K., Bilali, S., Ganocy, S. J. & Findling, R. L. (2005). A 20-month, double-blind, maintenance trial of lithium versus divalproex in rapid-cycling bipolar disorder. Am J Psychiatry 162, 2152-61.

Calabrese, J. R., Suppes, T., Bowden, C. L., Sachs, G. S., Swann, A. C., McElroy, S. L., Kusumakar, V., Ascher, J. A., Earl, N. L., Greene, P. L. & Monaghan, E. T. (2000). A double-blind, placebo-controlled, prophylaxis study of lamotrigine in rapid-cycling bipolar disorder. Lamictal 614 Study Group. J Clin Psychiatry 61, 841-50.

Carlson BX, Ketter TA, Sun W, Timko K, McQuade RD, Sanchez R, Vester-Blokland E, Marcus R. (2012). Aripiprazole in combination with lamotrigine for the long-term treatment of patients with bipolar I disorder (manic or mixed): a randomized, multicenter, double-blind study (CN138-392). Bipolar Disord. 14(1):41-53.

Coxhead, N., Silverstone, T. & Cookson, J. (1992). Carbamazepine versus lithium in the prophylaxis of bipolar affective disorder. Acta Psychiatr Scand 85, 114-8.

Cundall, R. L., Brooks, P. W. & Murray, L. G. (1972). A controlled evaluation of lithium prophylaxis in affective disorders. Psychol Med 2, 308-11.

Dunner, D. L., Stallone, F. & Fieve, R. R. (1976). Lithium carbonate and affective disorders. V: A double-blind study of prophylaxis of depression in bipolar illness. Arch Gen Psychiatry 33, 117-20.

Fieve, R. R., Kumbaraci, T. & Dunner, D. L. (1976). Lithium prophylaxis of depression in bipolar I, bipolar II, and unipolar patients. Am J Psychiatry 133, 925-9.

Geddes, J. R., Goodwin, G. M., Rendell, J., Azorin, J. M., Cipriani, A., Ostacher, M. J., Morriss, R., Alder, N. & Juszczak, E. (2010). Lithium plus valproate combination therapy versus

monotherapy for relapse prevention in bipolar I disorder (BALANCE): a randomised open-label trial. Lancet 375, 385-95.

Hartong, E. G., Moleman, P., Hoogduin, C. A., Broekman, T. G., Nolen, W. A. & LitCar, G. (2003). Prophylactic efficacy of lithium versus carbamazepine in treatment-naive bipolar patients. J Clin Psychiatry 64, 144-51.

Kane, J. M., Quitkin, F. M., Rifkin, A., Ramos-Lorenzi, J. R., Nayak, D. D. & Howard, A. (1982). Lithium carbonate and imipramine in the prophylaxis of unipolar and bipolar II illness: a prospective, placebo-controlled comparison. Arch Gen Psychiatry 39, 1065-9.

Keck, P. E., Jr., Calabrese, J. R., McIntyre, R. S., McQuade, R. D., Carson, W. H., Eudicone, J. M., Carlson, B. X., Marcus, R. N., Sanchez, R. & Aripiprazole Study, G. (2007). Aripiprazole monotherapy for maintenance therapy in bipolar I disorder: a 100-week, double-blind study versus placebo. J Clin Psychiatry 68, 1480-91.

Kleindienst, N. & Greil, W. (2000). Differential efficacy of lithium and carbamazepine in the prophylaxis of bipolar disorder: results of the MAP study. Neuropsychobiology 42 Suppl 1, 2-10.

Koyama, T., Higuchi, T., Yamawaki, S., Kanba, S., Terao, T. & Shinohara, A. (2011). Study SCA104779, an evaluation of BW430C (lamotrigine) versus placebo in the prevention of mood episodes in bipolar I disorder patients. Japanese Journal of Clinical Psychiatry 40, 369-383.

Melia, P. I. (1970). Prophylactic lithium: a double-blind trial in recurrent affective disorders. Br J Psychiatry 116, 621-4.

Prien, R. F., Klett, C. J. & Caffey, E. M., Jr. (1973a). Lithium carbonate and imipramine in prevention of affective episodes. A comparison in recurrent affective illness. Arch Gen Psychiatry 29, 420-5.

Prien, R. F., Caffey, E. M., Jr. & Klett, C. J. (1973b). Prophylactic efficacy of lithium carbonate in manic-depressive illness. Report of the Veterans Administration and National Institute of Mental Health collaborative study group. Arch Gen Psychiatry 28, 337-41.

Quiroz, J. A., Yatham, L. N., Palumbo, J. M., Karcher, K., Kushner, S. & Kusumakar, V. (2010). Risperidone long-acting injectable monotherapy in the maintenance treatment of bipolar I disorder. Biol Psychiatry 68, 156-62.

Szegedi, A., Durgam, S., Mackle, M., Yu, S. Y., Wu, X., Mathews, M. & Landbloom, R. P. (2018). Randomized, Double-Blind, Placebo-Controlled Trial of Asenapine Maintenance Therapy in Adults With an Acute Manic or Mixed Episode Associated With Bipolar I Disorder. Am J Psychiatry 175, 71-79.

Tohen, M., Calabrese, J. R., Sachs, G. S., Banov, M. D., Detke, H. C., Risser, R., Baker, R. W., Chou, J. C. & Bowden, C. L. (2006). Randomized, placebo-controlled trial of olanzapine as maintenance therapy in patients with bipolar I disorder responding to acute treatment with olanzapine. Am J Psychiatry 163, 247-56.

Tohen, M., Greil, W., Calabrese, J. R., Sachs, G. S., Yatham, L. N., Oerlinghausen, B. M., Koukopoulos, A., Cassano, G. B., Grunze, H., Licht, R. W., Dell'Osso, L., Evans, A. R., Risser, R., Baker, R. W., Crane, H., Dossenbach, M. R. & Bowden, C. L. (2005). Olanzapine versus lithium in the maintenance treatment of bipolar disorder: a 12-month, randomized, double-blind, controlled clinical trial. Am J Psychiatry 162, 1281-90.

Vieta, E., Cruz, N., Garcia-Campayo, J., de Arce, R., Manuel Crespo, J., Valles, V., Perez-Blanco, J., Roca, E., Manuel Olivares, J., Morinigo, A., Fernandez-Villamor, R. & Comes, M. (2008a). A double-blind, randomized, placebo-controlled prophylaxis trial of oxcarbazepine as adjunctive treatment to lithium in the long-term treatment of bipolar I and II disorder. Int J Neuropsychopharmacol 11, 445-52.

Vieta, E., Montgomery, S., Sulaiman, A. H., Cordoba, R., Huberlant, B., Martinez, L. & Schreiner, A. (2012). A randomized, double-blind, placebo-controlled trial to assess prevention of mood episodes with risperidone long-acting injectable in patients with bipolar I disorder. Eur Neuropsychopharmacol 22, 825-35.

Weisler, R. H., Nolen, W. A., Neijber, A., Hellqvist, A., Paulsson, B. & Trial 144 Study, I. (2011). Continuation of quetiapine versus switching to placebo or lithium for maintenance treatment of bipolar I disorder (Trial 144: a randomized controlled study). J Clin Psychiatry 72, 1452-64.

Woo, Y. S., Bahk, W. M., Chung, M. Y., Kim, D. H., Yoon, B. H., Lee, J. H., Ahn, Y. M., Chung, S. K., Kim, J. G., Lee, K. H. & Paik, K. C. (2011). Aripiprazole plus divalproex for recently manic or mixed patients with bipolar I disorder: a 6-month, randomized, placebo-controlled, double-blind maintenance trial. Hum Psychopharmacol 26, 543-53.

Young, A. H., McElroy, S. L., Olausson, B., Paulsson, B., Embolden, I. & Embolden, I. I. I. (2014). A randomised, placebo-controlled 52-week trial of continued quetiapine treatment in recently depressed patients with bipolar I and bipolar II disorder. World J Biol Psychiatry 15, 96-112.

<u>Studies included in the secondary network meta-analysis (K = 6)</u>

Bowden, C. L., Vieta, E., Ice, K. S., Schwartz, J. H., Wang, P. P. & Versavel, M. (2010). Ziprasidone plus a mood stabilizer in subjects with bipolar I disorder: a 6-month, randomized, placebo-controlled, double-blind trial. J Clin Psychiatry 71, 130-7.

Calabrese, J. R., Pikalov, A., Streicher, C., Cucchiaro, J., Mao, Y. & Loebel, A. (2017a). Lurasidone in combination with lithium or valproate for the maintenance treatment of bipolar I disorder. Eur Neuropsychopharmacol 27, 865-876.

Marcus, R., Khan, A., Rollin, L., Morris, B., Timko, K., Carson, W. & Sanchez, R. (2011). Efficacy of aripiprazole adjunctive to lithium or valproate in the long-term treatment of patients with bipolar I disorder with an inadequate response to lithium or valproate monotherapy: a multicenter, double-blind, randomized study. Bipolar Disord 13, 133-44.

Suppes, T., Vieta, E., Liu, S., Brecher, M., Paulsson, B. & Trial, I. (2009). Maintenance treatment for patients with bipolar I disorder: results from a north american study of quetiapine in combination with lithium or divalproex (trial 127). Am J Psychiatry 166, 476-88.

Tohen, M., Chengappa, K. N., Suppes, T., Baker, R. W., Zarate, C. A., Bowden, C. L., Sachs, G. S., Kupfer, D. J., Ghaemi, S. N., Feldman, P. D., Risser, R. C., Evans, A. R. & Calabrese, J. R. (2004). Relapse prevention in bipolar I disorder: 18-month comparison of olanzapine plus mood stabiliser v. mood stabiliser alone. Br J Psychiatry 184, 337-45.

Vieta, E., Suppes, T., Eggens, I., Persson, I., Paulsson, B. & Brecher, M. (2008). Efficacy and safety of quetiapine in combination with lithium or divalproex for maintenance of patients with bipolar I disorder (international trial 126). J Affect Disord 109, 251-63.

<u>Studies that were not included in the network meta-analysis (K = 3)</u>

Macfadden, W., Alphs, L., Haskins, J. T., Turner, N., Turkoz, I., Bossie, C., Kujawa, M. & Mahmoud, R. (2009). A randomized, double-blind, placebo-controlled study of maintenance treatment with adjunctive risperidone long-acting therapy in patients with bipolar I disorder who relapse frequently. Bipolar Disord 11, 827-39.

Yatham, L. N., Fallu, A. & Binder, C. E. (2007). A 6-month randomized open-label comparison of continuation of oral atypical antipsychotic therapy or switch to long acting injectable risperidone in patients with bipolar disorder. Acta Psychiatr Scand Suppl, 50-6.

Zarate, C. A., Jr. & Tohen, M. (2004). Double-blind comparison of the continued use of antipsychotic treatment versus its discontinuation in remitted manic patients. Am J Psychiatry 161, 169-71.

Supplementary Figure 2. Risk of bias summary

	Keck 2007	Kane 1982	Hartong 2003	Geddes 2010	Fieve 1976	Dunner 1976	Cundall 1972	Coxhead 1992	Carlson 2012	Calabrese 2017b	Calabrese 2017a	Calabrese 2005	Calabrese 2003	Calabrese 2000	Bowden 2012	Bowden 2010	Bowden 2003	Bowden 2000	Berwaerts 2012	Amsterdam 2010
Random sequence generation (selection bias)	?	?	?	+	?	?	?	?	?	+	?	?	?	?	?	?	?	?	?	?
Allocation concealment (selection bias)	?	?	?	+	?	?	?	?	?	+	?	?	?	?	?	?	?	?	?	?
Blinding of participants and personnel (performance bias)	+	+	+	−	+	+	+	+	+	+	?	+	+	+	+	+	+	+	+	+
Blinding of outcome assessment (detection bias)	+	+	+	+	+	+	+	+	+	+	?	+	+	+	+	+	+	+	+	+
Incomplete outcome data (attrition bias)	+	+	+	+	+	+	+	+	+	+	+	+	+	+	+	+	?	+	+	+
Selective reporting (reporting bias)	+	+	+	+	+	−	+	+	+	+	+	+	+	+	−	+	?	+	+	+
Other bias	+	+	+	−	−	−	−	−	+	+	+	+	−	−	+	+	−	+	+	+

	Zarate 2004	Young 2014	Yatham 2007	Woo 2011	Weisler 2011	Vieta 2012	Vieta 2008b	Vieta 2008a	Tohen 2006	Tohen 2005	Tohen 2004	Szegedi 2018	Suppes 2009	Quiroz 2010	Prien 1973b	Prien 1973a	Melia 1970	Marcus 2011	Macfadden 2009	Koyama 2011	Kleindienst 2000
Random sequence generation (selection bias)	?	?	−	?	+	?	?	+	?	?	+	?	?	+	?	?	?	+	+	?	+
Allocation concealment (selection bias)	?	?	−	?	+	?	?	?	?	?	+	?	?	+	?	?	?	+	+	?	+
Blinding of participants and personnel (performance bias)	?	+	−	+	+	+	?	+	+	+	+	+	?	+	?	?	+	+	+	+	−
Blinding of outcome assessment (detection bias)	?	+	−	+	+	+	?	+	+	+	+	+	?	+	+	+	+	+	+	+	?
Incomplete outcome data (attrition bias)	+	+	+	−	−	+	+	+	+	+	+	+	+	+	+	+	?	?	+	+	−
Selective reporting (reporting bias)	+	+	+	+	+	+	+	?	+	+	+	+	+	+	−	−	+	+	+	+	+
Other bias	−	+	−	+	+	+	+	+	+	+	−	+	+	+	−	−	−	+	+	−	+

Other bias: insufficient assessment of recurrent of mood episode

Supplementary Table 1. Study characteristics

Supplementary Table 1-1. Monotherapy studies

Study name, study duration, sponsor	Diagnosis (%BDII, criteria), mood status at recruitment	%female, mean age	%rapid cycling	Pre-randomization treatment	Double-blind treatment (n)*	Definition of relapse
Amsterdam 2010*, 50 wk, AC	BDII (100%, DSM-IV-TR), DE	46.3%, 38.0 yr	ni	FLU 20-80 mg/d	(1) LIT 0.5-1.5 mmol/L (26)<br>(2) PLA (27)	(1) MDE in DSM-IV-TR and HAMD ≥ 14<br>(2) HME in DSM-IV-TR (symptoms ≥ 4 and ≥4d) and YMRS ≥ 12
Berwaerts 2012*, 171.4 wk, IN	BDI (0%, DSM-IV), MaE or MiE	53.4%, 40.0 yr	0%	PAL 3-12 mg/d	<u>(1) PAL 3-12 mg/d (152)</u><br>(2) PLA (148)	(1) YMRS ≥ 15 and CGI-BP-S for mania ≥ 4<br>(2) YMRS < 15 and MADRS ≥ 16 and CGI-BP-S for depression ≥ 4<br>(3) Hospitalization<br>(4) Additional therapeutic intervention
Bowden 2000*, 52 wk, IN	BDI (0%, DSM-III-R), MaE, MiE or EU	50.9%, 39.2 yr	ni	LIT or DIV	(1) DIV 71-125 μg/mL (187)<br>(2) LIT 0.8-1.2 mmol/L (91)<br>(3) PLA (94)	(1) Depression : Additional AD intervention or discontinuation because of symptoms<br>(2) Mania: MRS ≥ 16 or hospitalization
Bowden 2003*, 76 wk, IN	BDI (0%, DSM-IV), MaE or HME	52.8%, 41.1 yr	ni	LAM 100-200 mg/d	<u>(1) LAM 100-400 mg/d (59)</u><br>(2) LIT 0.8-1.1 mEq/L (47)<br>(3) PLA (70)	(1) Additional therapeutic intervention including ECT
Calabrese 2000*, 26 wk, IN	BDI or II (28.9%, DSM-IV), MaE, HME, MiE, DE or EU	57.2%, 38.0 yr	100%	LAM 100-200 mg/d	<u>(1) LAM 100-500 mg/d (93)</u><br>(2) PLA (89)	(1) Additional pharmacological intervention
Calabrese 2003*, 76 wk, IN	BDI (0%, DSM-IV), DE	54.9%, 43.4 yr	ni	LAM ≥ 100 mg/d	<u>(1) LAM 200-400 mg/d (171)</u><br>(2) LIT 0.8-1.1 mEq/L (121)<br>(3) PLA (121)	(1) Additional therapeutic intervention including ECT

Study name, study duration, sponsor	Diagnosis (%BDII, criteria), mood status at recruitment	%female, mean age	%rapid cycling	Pre-randomization treatment	Double-blind treatment (n)*	Definition of relapse
Calabrese 2005*, 80 wk, AC	BDI or II (60.0%, DSM-IV), MaE, HME, MiE, DE or EU	51.7%. 37.1 yr	100%	DIV ≥ 50 μg/ml + LIT ≥ 0.8 mEq/l	(1) DIV ≥50 μg/mL (28)<br>(2) LIT ≥0.8 mEq/L (32)	(1) HAMD24 ≥ 20 for 8 wk<br>(2) YMRS ≥ 20 for 8 wk
Calabrese 2017b, 52 wk, IN	BDI (0%, DSM-IV-TR), MaE or MiE	57.5%, 40.6%	0%	AOM 400 mg/4w	(1) AOM 400 mg/4w (133)<br>(2) PLA (133)	(1) YMRS ≥ 15<br>(2) MADRS ≥ 15<br>(3) CGI-BP-S > 4<br>(4) Hospitalization<br>(5) Additional therapeutic intervention<br>(6) Discontinuation due to AE of worsening BDI<br>(7) Discontinuation due to lack of efficacy<br>(8) Active suicidality
Coxhead 1992*, 52 wk, IN	BD (ni, DSM-III), EU	67.7%, 48.0 yr	ni	LIT	(1) CAR 38-51 mmol/L (15)<br>(2) LIT 0.65-1.0 mEq/L (16)	(1) Author defined
Cundall 1972*, 26 wk, AC	Manic-Depressive (ni, ni), ni	61.5%, 53.7 yr	ni	LIT	(1) LIT 0.5-1.2 mEq/L (8)<br>(2) PLA (5)	(1) diagnosed clinically<br>(2) Hospitalization
Dunner 1976*, 65 wk, AC	BDII, BD others (100%, Feighner), EU	57.5%, 51.2 yr	15.0%	ni	(1) LIT 0.8-1.2 mEq/L (16)<br>(2) PLA (24)	(1) Additional pharmacological intervention
Fieve 1976*, 64 (mean) wk, AC	BDI or II (34.0%. Feighner), EU	50.9%, 46.6	3.8%	ni	(1) LIT 0.7-1.3 mEq/L (24)<br>(2) PLA (29)	(1) Author defined
Geddes 2010*, 104 wk, AC	BDI (0%, DSM-IV), EU	48.6%, 43.5 yr	ni	LIT 0.4-1.0 mEq/l + VAL ≥ 750 mg/d	(1) LIT 0.4-1.0 mEq/L (110)<br>(2) VAL 750-1250 mg/d (110)<br>(3) LIT + VAL (110)	(1) Hospitalization<br>(2) Additional therapeutic intervention

Study name, study duration, sponsor	Diagnosis (%BDII, criteria), mood status at recruitment	%female, mean age	%rapid cycling	Pre-randomization treatment	Double-blind treatment (n)*	Definition of relapse
Hartong 2003*, 104 wk, IN	BDI or II (ni, DSM-III-R), EU	54.3%, 41.9 yr	ni	< 6 m use CAR or LIT	(1) CAR 6-10 mg/L (30)<br>(2) LIT 0.6-1.0 mmol/L (23)	(1) DE in DSM-IV<br>(2) MaE/HME in DSM-IV
Kane 1982*, 104 wk, AC	BDII (100%, RDC), EU	69.4%, 47.5 yr	ni	IMI 150 mg/d	(1) LIT 0.8-1.2 mEq/L (4)<br>(2) PLA (7)	(1) Major depression for a wk (RDC)<br>(2) Mania for a wk (RDC)<br>(3) Minor depression for 4 wks (RDC)<br>(4) Hypomania for 4 wks (RDC)
Keck 2007*, 100 wk, IN	BDI (0%, DSM-IV), MaE or MiE	67.1%, 39.7 yr	17.5%	ARI 15-30 mg/d	(1) ARI 15-30 mg/d (78)<br>(2) PLA (83)	(1) Hospitalization<br>(2) Additional therapeutic intervention<br>(3) Discontinuation due to lack of efficacy
Kleindienst 2000*, 130 wk, AC	BDI, II or NOS (33.3%, DSM-IV), MaE, HME, MiE or DE	56.5%, 40.0 yr	ni	Free decision	(1) CAR 4-12 μg/mL (85)<br>(2) LIT 0.6-0.8 mEq/L (86)	(1) Author defined
Koyama 2011*, 26 wk, IN	BDI (0%, DSM-IV-TR), MaE, MiE, DE or EU	56.3%, 42.8 yr	ni	LAM 100-200 mg/d	(1) LAM 100-200 mg/d (45)<br>(2) PLA (58)	(1) Additional pharmacological intervention
Melia 1970*, 104 wk, IN	BD (ni, ICD-9), EU	90.9%, 51.5 yr	ni	LIT	(1) LIT 500-1500 mg/d (5)<br>(2) PLA (6)	(1) Sufficient severity to admission to hospital
Prien 1973a*, 17.3 wk, AC	BD (ni, ni), DE	23.0%, 45.7 yr	ni	LIT or IMI	(1) LIT 0.5-1.4 mEq/L (18)<br>(2) PLA (13)	(1) Additional pharmacological intervention<br>(2) Hospitalization
Prien 1973b*, 104 wk, AC	Manic-depressive manic type (ni, ni), MaE or HME	35.1%, 44 (median) yr	ni	LIT	(1) LIT 0.5-1.4 mEq/L (101)<br>(2) PLA (104)	(1) Additional pharmacological intervention<br>(2) Hospitalization

Study name, study duration, sponsor	Diagnosis (%BDII, criteria), mood status at recruitment	%female, mean age	%rapid cycling	Pre-randomization treatment	Double-blind treatment (n)*	Definition of relapse
Quiroz 2010*, 104 wk, IN	BDI (0%, DSM-IV-TR), MaE, MiE or EU	48.5%, 39.0 yr	0%	RIS-LAI 12.5, 25, 37.5, 50 mg/2wk	(1) RIS-LAI 12.5-50 mg/2wk (154)<br>(2) PLA (149)	(1) YMRS > 12<br>(2) MADRS > 12<br>(3) CGI-S > 4<br>(3) Hospitalization<br>(4) Additional therapeutic intervention including increase dosage of RIS<br>(5) Any mood episode in DSM-IV
Szegedi 2018, 26 wk, IN	BDI (0%, DSM-IV-TR), MaE or MiE	54.8%, 41.9 yr	0%	ASE 5 or 10 mg bid	(1) ASE 10-20 mg (126)<br>(2) PLA (127)	(1) YMRS ≥ 16<br>(2) MADRS ≥ 16<br>(3) Hospitalization<br>(4) Additional therapeutic intervention<br>(5) Discontinuation because of a mood event
Tohen 2005*, 48 wk, IN	BDI (0%, DSM-IV), MaE or MiE	52.9%, 42.4 yr	3%	LIT 0.6-1.2 mEq/l + OLA<br>5-20 mg/d	(1) LIT 0.6-1.2 mEq/L (214)<br>(2) OLA 5-20 mg/d (217)	(1) HAMD ≥ 15<br>(2) YMRS ≥ 15
Tohen 2006*, 48 wk, IN	BDI (0%, DSM-IV), MaE or MiE	61.2%, 40.6 yr	49.6%	OLA 5-20 mg/d	(1) OLA 5-20 mg/d (225)<br>(2) PLA (136)	(1) HAMD ≥ 15<br>(2) YMRS ≥ 15<br>(3) Hospitalization
Vieta 2012*, 78 wk, IN	BDI (0%, DSM-IV-TR), MaE, MiE or EU	52.1%, 36.9 yr	0%	RIS-LAI 25, 37.5, 50 mg/2wk	(1) OLA 10 mg/d (131)<br>(2) RIS-LAI 25-50 mg/d (132)<br>(3) PLA (135)	(1) YMRS > 12<br>(2) MADRS > 12<br>(3) CGI-S ≥ 4<br>(3) Hospitalization<br>(4) Additional therapeutic intervention including increase dosage of RIS

Study name, study duration, sponsor	Diagnosis (%BDII, criteria), mood status at recruitment	%female, mean age	%rapid cycling	Pre-randomization treatment	Double-blind treatment (n)*	Definition of relapse
Weisler 2011*, 104 wk, IN	BDI (0%, DSM-IV), MaE, MiE, DE or EU	51.5%, 39.5 yr	13.4%	QUE 300-800 mg/d	(1) LIT 0.6-1.2 mEq/L (364)<br>(2) QUE 300-800 mg/d (404)<br>(3) PLA (404)	(1) YMRS ≥ 20<br>(2) MADRS ≥ 20<br>(3) Additional pharmacological intervention<br>(4) Hospitalization<br>(5) Discontinuation because of a mood event
Young 2014*, 52 wk, IN	BDI or II (39.6%, DSM-IV), DE	59.6%, 40.1 yr	8.7%	QUE 300 or 600 mg/d	(1) QUE 300-600 mg/d (291)<br>(2) PLA (294)	(1) YMRS ≥ 16<br>(2) MADRS ≥ 20<br>(3) Hospitalization<br>(4) Additional therapeutic intervention<br>(5) Discontinuation because of a mood event

Supplementary Table 1-2. Combination therapy studies

Study name, study duration, sponsor	Diagnosis (%BDII, criteria), mood status at recruitment	%female, mean age	%rapid cycling	Pre-randomization treatment	Double-blind treatment (n)*	Definition of relapse
Bowden 2010, 24 wk, IN	BDI (0%, DSM-IV), MaE or MiE	53.8%, 38.8 yr	ni	ZIP 80-160 mg/d + LIT 0.6-1.2 mEq/l or VAL 50-125 µg/ml	<u>(1) ZIP 80-160 mg/d + LIT 0.6-1.2 mEq/L or VAL 50-125 µg/mL (127)</u><br>(2) PLA + LIT 0.6-1.2 mEq/L or VAL 50-125 µg/mL (113)	(1) Additional therapeutic intervention
Bowden 2012*, 34.7 wk, AC	BDI or II (21.0%, DSM-IV), DE or EU	57.4%, 40.7 yr	ni	LAM 50-200 mg/d<br>+DIV 45-120 µg/ml	<u>(1) LAM 50-200 mg/d + DIV 500-2500 mg/d (41)</u><br>(2) PLA + LAM 50-200 mg/d (45)	(1) MRS ≥ 15<br>(2) MADRS ≥ 15
Calabrese 2017a, 28 wk, IN	BDI (0%, DSM-IV-TR), MaE, MiE or DE	56.3%, 44.5 yr	13.3%	LUR 20-80mg/d + LIT 0.4-1.2 mEq/l or VAL 50-125 µg/ml	<u>(1) LUR 20-80mg/d + LIT 0.4-1.2 mEq/L or VAL 50-125 µg/mL (246)</u><br>(2) PLA + LIT 0.4-1.2mEq/L or VAL 50-125 µg/mL (250)	(1) YMRS ≥ 18<br>(2) MADRS ≥ 18<br>(3) CGI-BP-S ≥ 4<br>(4) Hospitalization<br>(5) Any mood episode in DSM-IV-TR<br>(6) Discontinuation because of a mood event
Carlson 2012*, 52 wk, IN	BDI (0%, DSM-IV-TR), MaE or MiE	65.0%. 39.1 yr	27.1%	ARI 10-30 mg/d +LAM<br>100-200 mg/d	<u>(1) ARI 10-30 mg/d + LAM 100-200 mg/d (178)</u><br>(2) PLA + LAM100-200 mg/d (173)	(1) Hospitalization<br>(2) SAE or worsening disease (and YMRS > 14 and/or MADRS > 16)<br>(3) Discontinuation due to lack of efficacy (and YMRS > 14 and/or MADRS > 16)

Study name, study duration, sponsor	Diagnosis (%BDII, criteria), mood status at recruitment	%female, mean age	%rapid cycling	Pre-randomization treatment	Double-blind treatment (n)*	Definition of relapse
Macfadden 2009, 52 wk, IN	BDI (0%, DSM-IV-TR), MaE, HME, MiE, DE or EU	28.2%. 38.9 yr	100%	TAU	(1) RISLAI 25-50 mg/d + TAU (65)<br>(2) TAU (59)	(1) YMRS > 15 and CGI-BP-S ≥ 4, CGI-BP-C ≥ 6 or GAF decrease >10<br>(2) MADRS > 16 and CGI-BP-S ≥ 4, CGI-BP-C ≥ 6 or GAF decrease >10<br>(3) Hospitalization [(1) or (2)]<br>(4) Hospitalization (ISST > 7)
Marcus 2011, 52 wk, IN	BDI (0%, DSM-IV-TR), MaE or MiE	54.9%, 39.0 yr	3.9%	ARI 10-30 mg/d + LIT 0.6-1.0 mmol/l or VAL 50-125 μg/ml	(1) ARI 10-30 mg/d + LIT 0.6-1.0 mmol/L or VAL 50-125 μg/mL (168)<br>(2) PLA + LIT 0.6-1.0 mmol/L or VAL 50-125 μg/mL (169)	(1) Hospitalization<br>(2) SAE of worsening disease (and YMRS > 16 and/or MADRS > 16)<br>(3) Discontinuation due to lack of efficacy (and YMRS > 16 and/or MADRS > 16)
Suppes 2009, 104 wk, IN	BDI (0%, DSM-IV), MaE, MiE or DE	52.5%, 40.1 yr	51.0%	QUE 400-800 mg/d + DIV 50-125 μg/ml or LIT 0.5-1.2 mEq/l	(1) QUE 400-800 mg/d + DIV 50-125 μg/mL or LIT 0.5-1.2 mEq/L (310)<br>(2) PLA + DIV 50-125 μg/mL or LIT 0.5-1.2 mEq/L (313)	(1) YMRS ≥ 20<br>(2) MADRS ≥ 20<br>(3) Hospitalization<br>(4) Additional therapeutic intervention<br>(5) Discontinuation because of a mood event
Tohen 2004, 78 wk, IN	BDI (0%, DSM-IV), MaE or MiE	51.5%, 41.3 yr	41.4%	OLA 5-20 mg/d + LIT 0.6-1.2 mmol/l or VAL 50-125 μg/ml	(1) OLA 5-20 mg/d + LIT 0.6-1.2 mmol/L or VAL 50-125 μg/mL (51)<br>(2) PLA + LIT 0.6-1.2 mmol/L or VAL 50-125 μg/mL (48)	(1) HAMD ≥ 15<br>(2) YMRS ≥ 15<br>(3) Any mood episode in DSM-IV

Study name, study duration, sponsor	Diagnosis (%BDII, criteria), mood status at recruitment	%female, mean age	%rapid cycling	Pre-randomization treatment	Double-blind treatment (n)*	Definition of relapse
Vieta 2008a*, 52 wk, IN	BDI or II (23.6%, DSM-IV-TR), EU	65.5%, 43.5 yr	ni	LIT ≥ 0.6 mEq/l	(1) OXC 300-1200 mg/d + LIT ≥ 0.6 mEq/L (26)<br>(2) PLA + LIT ≥ 0.6 mEq/L (29)	(1) YMRS > 12<br>(2) MADRS > 20<br>(3) MaE, HME, MiE or DE in DSM-IV
Vieta 2008b, 104 wk, IN	BDI (0%, DSM-IV), MaE, MiE or DE	55.0%, 42.1 yr	23.9%	QUE 400-800 mg/d + DIV 50-125 μg/ml or LIT 0.5-1.2 mEq/l	(1) QUE 400-800 mg/d + DIV 50-125 μg/mL or LIT 0.5-1.2 mEq/L (336)<br>(2) PLA + DIV 50-125 μg/mL or LIT 0.5-1.2 mEq/L (367)	(1) YMRS ≥ 20<br>(2) MADRS ≥ 20<br>(3) Hospitalization<br>(4) Additional therapeutic intervention<br>(5) Discontinuation because of a mood event
Woo 2011*, 24 wk, IN	BDI (0%, DSM-IV), MaE or MiE	61.4%, 38.3 yr	ni	ARI 10-30 mg/d + DIV 50-120 μg/ml	(1) ARI 10-30 mg/d + DIV 50-120 μg/mL (40)<br>(2) PLA + DIV 50-120 μg/mL (43)	(1) YMRS ≥ 15<br>(2) MADRS ≥ 16<br>(3) Hospitalization<br>(4) Additional therapeutic intervention
Yatham 2007, 26 wk, IN	BDI or II (34.7%, DSM-IV-TR), ni	51.0%, 40.9 yr	ni	OLA, QUE or RIS in combination with a maximum of two of LAM, LIT, or VAL	(1) RISLAI 25-50 mg/d (23)<br>(2) OLA mean 8 mg/d, QUE mean 352.3 mg/d or RIS mean 1.4 mg/d (26)<br>Patients who had taken LAM, LIT, or VAL continue them	ni
Zarate 2004, 26 wk, AC	BD (ni, DSM-IV), MaE or MiE	78.9%, 35.8 yr	0%	PER 4-64 mg/d + CAR 4-12 mg/l, LIT 0.6-1.2 mEq/l or VAL 50-125 mg/l	(1) PER 4-64 mg/d + CAR 4-12 mg/L, LIT 0.6-1.2 mEq/L or VAL 50-125 mg/L (18)<br>(2) PLA + CAR 4-12 mg/L, LIT 0.6-1.2 mEq/L or VAL 50-125 mg/L (19)	(1) Any mood episode in DSM-IV

*This study was included in the previous meta-analysis (Miura et al., 2014).

**The study with enrichment design was underlined.

The following studies were included in the previous meta-analysis (Miura et al., 2014), but not our study because the studies did not have multiple treatment arms other than antidepressants.

Amsterdam JD, Shults J. Fluoxetine monotherapy of bipolar type II and bipolar NOS major depression: a double-blind, placebo-substitution, continuation study. Int Clin Psychopharmacol. 2005 Sep;20(5):257-64.

Kane JM, Quitkin FM, Rifkin A, Ramos-Lorenzi JR, Saraf K, Howard A, Klein DF. Prophylactic lithium with and without imipramine for bipolar I patients: a double-blind study [proceedings]. Psychopharmacol Bull. 1981 Jan;17(1):144-5.

Prien RF, Kupfer DJ, Mansky PA, Small JG, Tuason VB, Voss CB, Johnson WE. Drug therapy in the prevention of recurrences in unipolar and bipolar affective disorders. Report of the NIMH Collaborative Study Group comparing lithium carbonate, imipramine, and a lithium carbonate-imipramine combination. Arch Gen Psychiatry. 1984 Nov;41(11):1096-104.

AC: academia, AD: antidepressant, AOM: aripiprazole once-monthly, AP: antipsychotic, ARI: aripiprazole, ASE: asenapine, BD: bipolar disorder, CAR: carbamazepine, CGI-BP-S(C): Clinical Global Impressions of Bipolar Disorder-Severity(-Change), d: day, DIV: divalproex, DSM: Diagnostic and Statistical Manual of Mental Disorders, ECT: electroconvulsive therapy, EU: euthymia, FLU: fluoxetine, GAF: Global Assessment of Functioning, HAMD: Hamilton Rating Scale for Depression, HME: hypomanic episode, ICD: International Classification of Diseases, IMI: imipramine, IN: industry, ISST: InterSePT Scale for Suicidal Thinking, LAM: lamotrigine, LIT: lithium, MaE: manic episode/ (M)DE: (major) depressive episode, MiE: mixed episode, MRS: Mania Rating Scale, n: number of patients, ni: not information, NOS: not otherwise specified, OXC: oxcarbazepine, PAL: paliperidone, PER: perphenazine, PLA: placebo, QUE: quetiapine, RDC: Research Diagnostic Criteria, RIS-LAI: risperidone long-acting injectable, SAE: serious adverse event, TAU: treatment-as-usual (TAU consisted of any number or combinationsof antidepressants, mood stabilizers or anxiolytics), VAL: valproate, YMRS: Young Mania Rating Scale, yr: year, wk: week, ZIP: ziprasidone

Supplementary Table 2. The primary outcome (or main outcome) of the individual study included in our systematic review

Supplementary Table 2-1. Monotherapy studies

Study name	The primary outcome (or main outcome) of the individual study*
Amsterdam 2010	Time to relapse or recurrence of depressive episodes (LIT vs PLA): not information
Berwaerts 2012	Time to recurrence of any mood symptoms: PAL > PLA
Bowden 2000	Time to recurrence of any mood episode: DIV = PLA, DIV = LIT, LIT = PLA
Bowden 2003	Time to intervention for any mood episode: LAM > PLA, LIT > PLA, LAM = LIT
Calabrese 2000	Time to additional pharmacotherapy for emerging symptoms: LAM = PLA
Calabrese 2003	Time to additional pharmacotherapy for emerging symptoms: LAM > PLA, LIT > PLA, LAM = LIT
Calabrese 2005	Time to treatment for a mood episode: DIV = LIT
Calabrese 2017b	Time to recurrence of any mood episode: AOM > PLA
Coxhead 1992	Relapse rate: CAR = LIT
Cundall 1972	Relapse rate: LIT > PLA
Dunner 1976	Hypomanic episodes/patient year: LIT > PLA, depressive episodes/patient year: LIT = PLA
Fieve 1976	Frequency of depressive episodes: LIT > PLA, dropouts due to depression: LIT > PLA
Geddes 2010	Initiation of new intervention for an emergent mood episode: LIT+VAL > VAL, LIT+VAL > LIT, LIT > VAL
Hartong 2003	Recurrence of an episode of (hypo)mania or major depression: LIT > CAR
Kane 1982	Relapses of any type: LIT > PLA
Keck 2007	Time to relapse of any mood episode: ARI > PLA
Kleindienst 2000	Hospitalization: LIT > CAR
Koyama 2011	Time to withdrawal from study: LAM > PLA
Melia 1970	Length of remission: LIT > PLA
Prien 1973a	Number of affective episodes: LIT > PLA
Prien 1973b	Number of affective episodes: LIT > PLA
Quiroz 2010	Time to recurrence for any mood episode: RISLAI > PLA
Szegedi 2018	Time to recurrence of any mood event: ASE > PLA

Study name	The primary outcome (or main outcome) of the individual study*
Tohen 2005	Symptomatic recurrence of any mood episode following remission of mania or depression: LIT = OLA
Tohen 2006	Time to symptomatic relapse into any mood episode: OLA > PLA
Vieta 2012	Time to recurrence of any mood episode: RISLAI = PLA, OLA > PLA
Weisler 2011	Time to recurrence of any mood episode: QUE > PLA
Young 2014	Time to recurrence of a mood event: QUE > PLA

Supplementary Table 2-2. Combination therapy studies

Study name	The primary outcome (or main outcome) of the individual study*
Bowden 2010	Time to intervention for a mood episode: ZIP+LIT/VAL > PLA+LIT/VAL
Bowden 2012	Time to depressive episode: LAM+DIV = PLA+LAM
Calabrese 2017a	Time to recurrence of any mood episode: LUR+LIT/VAL = PLA+LIT/VAL
Carlson 2012	Time from randomization to relapse into a manic／mixed episode: ARI+LAM = PLA+LAM
Macfadden 2009	Time to relapse to any mood episode: RISLAI+TAU > PLA+TAU
Marcus 2011	Time from randomization to relapse due to any mood episode: ARI+LIT/VAL > PLA+LIT/VAL
Suppes 2009	Time to recurrence of any mood event: QUE+LIT/DIV > PLA+LIT/DIV
Tohen 2004	Time to syndromic relapse: OLA+LIT/VAL = PLA+LIT/VAL
Vieta 2008a	Length of the remission period: LIT+OXC = LIT+PLA
Vieta 2008b	Time to recurrence of any mood event: QUE+LIT/DIV > PLA+LIT/DIV
Woo 2011	Time from randomization to relapse of any mood episode: ARI+DIV = PLA+DIV
Yatham 2007	Safety and efficacy outcomes: RIS-LAI+oral OLA, QUE or RIS = oral OLA, QUE or RIS
Zarate 2004	Time to relapse into depression: PLA + CAR, LIT or VAL > PER + CAR, LIT or VAL

* A > B means that A was superior to B. AOM: aripiprazole once-monthly, ARI: aripiprazole, ASE: asenapine, CAR: carbamazepine, DIV: divalproex, LAM: lamotrigine, LIT: lithium, LUR: lurasidone, OXC: oxcarbazepine, PAL: paliperidone, PER: perphenazine, PLA: placebo, QUE: quetiapine, RIS-LAI: risperidone long-acting injectable, TAU: treatment-as-usual (TAU consisted of any number or combinations of antidepressants, mood stabilizers or anxiolytics), VAL: valproate, ZIP: ziprasidone

Supplementary Appendix 1. Results of first network meta-analysis.

1.1. Recurrence/relapse rate of any mood episode (29 studies, 6890 patients)

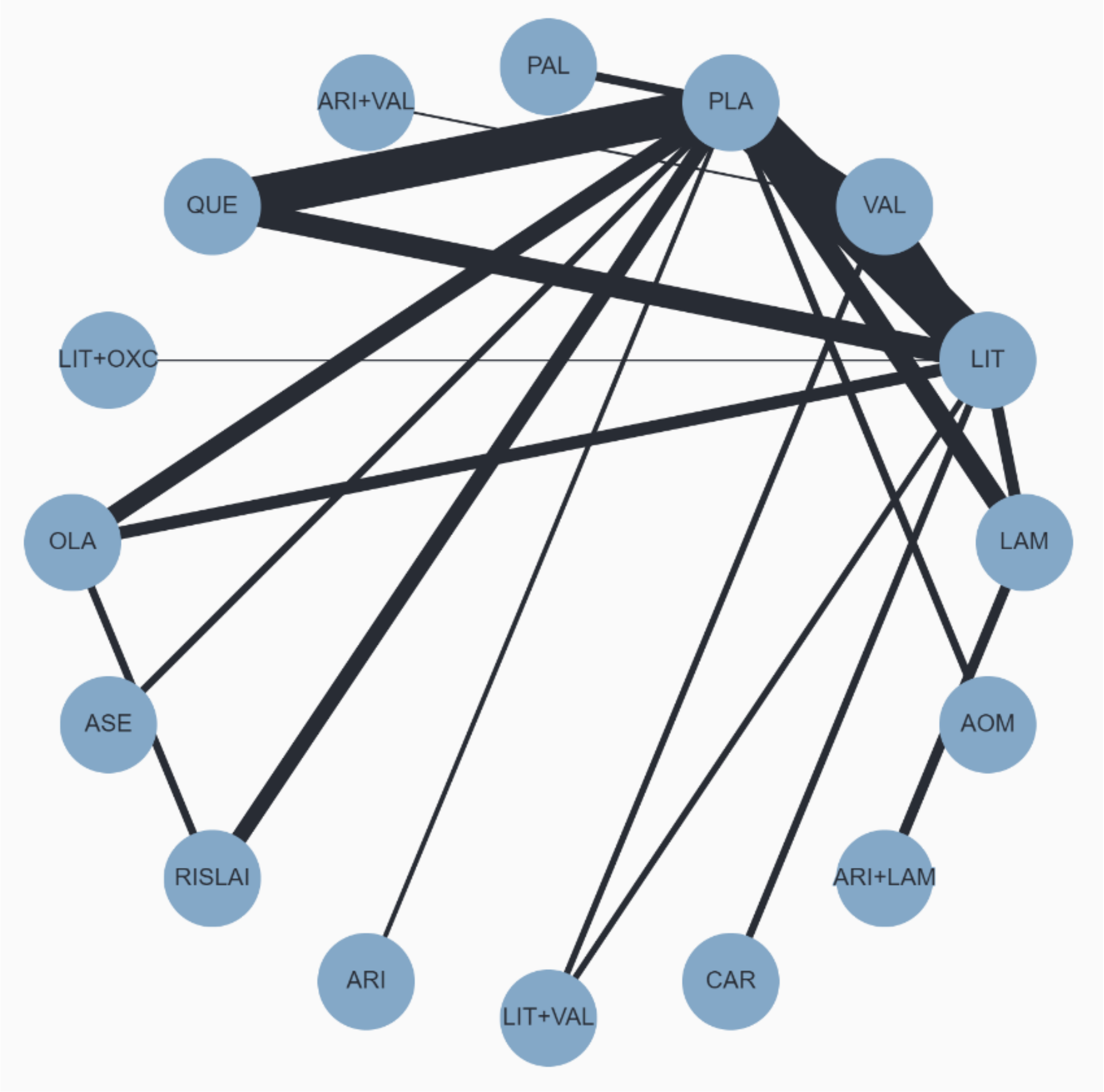

League table

Results from the pairwise meta-analysis are presented in the left lower half and results from the network meta-analysis in the upper right half.

The boldface result indicates statistical significance.

AOM	0.838 (0.438, 1.604)	0.978 (0.506, 1.888)	1.776 (0.630, 5.007)	1.980 (0.882, 4.443)	0.759 (0.409, 1.406)	0.679 (0.420, 1.096)	0.831 (0.524, 1.319)	1.267 (0.574, 2.794)	0.987 (0.557, 1.749)	1.037 (0.635, 1.692)	0.621 (0.350, 1.104)	0.986 (0.597, 1.628)	0.814 (0.486, 1.363)	0.818 (0.490, 1.366)	**0.519 (0.335, 0.803)**
	ARI	1.166 (0.587, 2.319)	2.118 (0.737, 6.086)	**2.362 (1.028, 5.428)**	0.905 (0.473, 1.730)	0.810 (0.482, 1.360)	0.991 (0.600, 1.638)	1.511 (0.669, 3.415)	1.178 (0.643, 2.157)	1.237 (0.729, 2.098)	0.741 (0.404, 1.361)	1.176 (0.685, 2.017)	0.971 (0.559, 1.687)	0.976 (0.563, 1.691)	**0.619 (0.383, 0.999)**
		ARI+LAM	1.816 (0.631, 5.226)	2.025 (0.875, 4.688)	0.776 (0.405, 1.485)	0.694 (0.442, 1.090)	0.850 (0.514, 1.407)	1.295 (0.573, 2.930)	1.010 (0.550, 1.853)	1.060 (0.620, 1.814)	0.635 (0.343, 1.179)	1.008 (0.582, 1.744)	0.832 (0.474, 1.461)	0.837 (0.481, 1.454)	**0.530 (0.324, 0.868)**
			ARI+VAL	1.115 (0.350, 3.558)	0.427 (0.154, 1.183)	**0.382 (0.147, 0.995)**	0.468 (0.184, 1.190)	0.713 (0.230, 2.214)	0.556 (0.212, 1.455)	0.584 (0.223, 1.525)	**0.350 (0.127, 0.962)**	0.555 (0.211, 1.461)	0.458 (0.172, 1.219)	0.461 (0.187, 1.134)	**0.292 (0.114, 0.748)**
				ASE	**0.383 (0.171, 0.859)**	**0.343 (0.169, 0.696)**	**0.420 (0.209, 0.842)**	0.640 (0.248, 1.649)	0.499 (0.230, 1.081)	0.523 (0.256, 1.071)	**0.314 (0.144, 0.682)**	0.498 (0.241, 1.026)	**0.411 (0.197, 0.856)**	**0.413 (0.199, 0.858)**	**0.262 (0.133, 0.517)**
					CAR	0.895 (0.561, 1.427)	1.095 (0.728, 1.649)	1.670 (0.780, 3.576)	1.301 (0.762, 2.221)	1.366 (0.850, 2.197)	0.819 (0.461, 1.454)	1.299 (0.796, 2.121)	1.073 (0.643, 1.791)	1.078 (0.671, 1.734)	0.684 (0.442, 1.057)
		0.694 (0.442, 1.090)				LAM	1.224 (0.978, 1.533)	1.866 (0.945, 3.685)	1.454 (0.968, 2.184)	**1.527 (1.141, 2.044)**	0.915 (0.600, 1.396)	**1.452 (1.063, 1.984)**	1.199 (0.856, 1.679)	1.205 (0.875, 1.659)	**0.764 (0.628, 0.930)**
					1.095 (0.728, 1.649)	1.101 (0.809, 1.499)	LIT	1.524 (0.802, 2.897)	1.188 (0.842, 1.676)	1.247 (0.980, 1.587)	0.748 (0.500, 1.118)	1.186 (0.905, 1.553)	0.979 (0.719, 1.333)	0.984 (0.773, 1.253)	**0.624 (0.537, 0.725)**

							1.524 (0.802, 2.897)	LIT+OXC	0.779 (0.376, 1.615)	0.818 (0.412, 1.625)	0.491 (0.230, 1.047)	0.778 (0.388, 1.562)	0.642 (0.315, 1.310)	0.646 (0.325, 1.283)	**0.409 (0.212, 0.792)**
							1.102 (0.761, 1.595)		LIT+VAL	1.050 (0.692, 1.592)	0.630 (0.372, 1.064)	0.999 (0.647, 1.540)	0.824 (0.522, 1.303)	0.829 (0.591, 1.163)	**0.525 (0.363, 0.760)**
							1.295 (0.877, 1.913)			OLA	**0.599 (0.388, 0.926)**	0.951 (0.685, 1.320)	0.785 (0.566, 1.089)	0.789 (0.566, 1.100)	**0.500 (0.400, 0.625)**
											PAL	**1.586 (1.013, 2.482)**	1.310 (0.824, 2.082)	1.317 (0.831, 2.085)	0.835 (0.575, 1.212)
							1.159 (0.792, 1.696)					QUE	0.826 (0.571, 1.194)	0.830 (0.583, 1.181)	**0.526 (0.411, 0.674)**
										**0.613 (0.382, 0.983)**			RISLAI	1.005 (0.687, 1.472)	**0.637 (0.484, 0.839)**
			0.461 (0.187, 1.134)				1.007 (0.781, 1.299)		0.776 (0.543, 1.111)					VAL	**0.634 (0.485, 0.829)**
**0.519 (0.335, 0.803)**	**0.619 (0.383, 0.999)**			**0.262 (0.133, 0.517)**		**0.795 (0.647, 0.976)**	**0.618 (0.525, 0.728)**			**0.521 (0.400, 0.680)**	0.835 (0.575, 1.212)	**0.508 (0.393, 0.657)**	**0.613 (0.462, 0.813)**	**0.628 (0.396, 0.997)**	PLA

Evaluation of heterogeneity and inconsistency

We inferred the magnitude of heterogeneity by comparing the estimated $\tau 2$ to empirical distributions of heterogeneity typically found in meta-analyses. The predictive τ^2 distribution for mental health outcomes according to Rhodes et al. has a median of 0.049 and an IQR of 0.01– 0.242. Low heterogeneity could be considered when the estimated τ^2 is less than the 25% quantile of the empirical distribution, moderate heterogeneity for τ^2 between 25% and 50% quantile and high heterogeneity for τ^2 larger than the 50% quantile. We evaluated global consistency under the assumption of a full design-by-treatment interaction model in netmeta.

Rhodes KM, Turner RM, Higgins JPT. Empirical evidence about inconsistency among studies in a pairwise meta-analysis. Research synthesis methods 2016; 7: 346–70.

Between study variance (τ^2)	Heterogeneity assessment	Random-effects design-by-treatment interaction model		
		Q	df	p
0.021	Low	7.776	10	0.651

Inconsistency

	NMA, RR (95%CI)	Direct, RR (95%CI)	Indirect, RR (95%CI)	Inconsistency measures	
				Ratio of risk ratios	P value
LAM vs LIT	1.224 (0.978, 1.533)	1.101 (0.809, 1.499)	1.380 (0.994, 1.915)	0.798 (0.509, 1.252)	0.326
LAM vs PLA	0.764 (0.628, 0.930)	0.795 (0.647, 0.976)	0.489 (0.246, 0.975)	1.624 (0.791, 3.334)	0.186
LIT vs LIT+VAL	1.188 (0.842, 1.676)	1.102 (0.761, 1.595)	1.933 (0.754, 4.957)	0.570 (0.207, 1.567)	0.276
LIT vs OLA	1.247 (0.980, 1.587)	1.295 (0.877, 1.913)	1.219 (0.897, 1.655)	1.062 (0.647, 1.744)	0.811
LIT vs QUE	1.186 (0.905, 1.553)	1.159 (0.792, 1.696)	1.214 (0.828, 1.780)	0.954 (0.556, 1.637)	0.865
LIT vs VAL	0.984 (0.773, 1.253)	1.007 (0.781, 1.299)	0.793 (0.364, 1.729)	1.270 (0.560, 2.884)	0.567
LIT vs PLA	0.624 (0.537, 0.725)	0.618 (0.525, 0.728)	0.653 (0.454, 0.939)	0.947 (0.636, 1.411)	0.788
LIT+VAL vs VAL	0.829 (0.591, 1.163)	0.776 (0.543, 1.111)	1.424 (0.508, 3.990)	0.545 (0.183, 1.624)	0.276
OLA vs RISLAI	0.785 (0.566, 1.089)	0.613 (0.382, 0.983)	0.987 (0.627, 1.553)	0.621 (0.322, 1.195)	0.153
OLA vs PLA	0.500 (0.400, 0.625)	0.521 (0.400, 0.680)	0.454 (0.302, 0.683)	1.149 (0.706, 1.871)	0.576
QUE vs PLA	0.526 (0.411, 0.674)	0.508 (0.393, 0.657)	0.801 (0.330, 1.946)	0.634 (0.252, 1.598)	0.334
RISLAI vs PLA	0.637 (0.484, 0.839)	0.613 (0.462, 0.813)	1.222 (0.383, 3.898)	0.502 (0.152, 1.656)	0.258
VAL vs PLA	0.634 (0.485, 0.829)	0.628 (0.396, 0.997)	0.637 (0.458, 0.886)	0.987 (0.560, 1.740)	0.963

Rank probabilities

	Rank 1	Rank 2	Rank 3	Rank 4	Rank 5	Rank 6	Rank 7	Rank 8	Rank 9	Rank 10	Rank 11	Rank 12	Rank 13	Rank 14	Rank 15	Rank 16
AOM	0.007	0.048	0.119	0.140	0.117	0.105	0.095	0.080	0.063	0.055	0.051	0.048	0.035	0.024	0.012	0.002
ARI	0.004	0.021	0.045	0.064	0.069	0.074	0.075	0.079	0.073	0.077	0.081	0.094	0.086	0.083	0.054	0.022
ARI+LAM	0.008	0.044	0.099	0.123	0.093	0.092	0.082	0.080	0.069	0.064	0.068	0.065	0.051	0.034	0.020	0.009
ARI+VAL	0.302	0.283	0.148	0.064	0.041	0.032	0.025	0.020	0.016	0.013	0.013	0.012	0.009	0.010	0.008	0.005
ASE	0.553	0.293	0.089	0.029	0.015	0.006	0.006	0.003	0.002	0.002	0.001	0.001	0.001	0.000	0.000	0.000
CAR	0.000	0.001	0.003	0.008	0.013	0.019	0.024	0.037	0.041	0.058	0.083	0.116	0.153	0.197	0.171	0.079
LAM	0.000	0.000	0.000	0.000	0.001	0.001	0.003	0.007	0.014	0.032	0.068	0.144	0.284	0.302	0.140	0.004
LIT	0.000	0.000	0.000	0.002	0.004	0.020	0.063	0.137	0.229	0.245	0.186	0.093	0.020	0.003	0.000	0.000
LIT+OXC	0.116	0.246	0.241	0.102	0.070	0.051	0.044	0.027	0.023	0.018	0.018	0.013	0.013	0.010	0.005	0.003
LIT+VAL	0.005	0.027	0.097	0.152	0.139	0.127	0.124	0.097	0.075	0.054	0.040	0.029	0.018	0.011	0.004	0.001
OLA	0.003	0.021	0.091	0.169	0.215	0.190	0.134	0.086	0.048	0.023	0.012	0.005	0.003	0.001	0.000	0.000
PAL	0.000	0.001	0.001	0.003	0.006	0.006	0.012	0.017	0.019	0.029	0.046	0.075	0.127	0.194	0.304	0.160
QUE	0.002	0.013	0.057	0.122	0.157	0.173	0.157	0.118	0.082	0.052	0.035	0.021	0.010	0.004	0.001	0.000
RISLAI	0.000	0.001	0.007	0.014	0.032	0.055	0.079	0.098	0.109	0.121	0.148	0.153	0.102	0.058	0.021	0.002
VAL	0.000	0.001	0.003	0.010	0.031	0.051	0.078	0.115	0.138	0.158	0.150	0.131	0.086	0.036	0.013	0.000
PLA	0.000	0.000	0.000	0.000	0.000	0.000	0.000	0.000	0.000	0.000	0.000	0.000	0.003	0.036	0.249	0.712

SUCRA

ARI+VAL	0.85
LIT+OXC	0.83
ASE	0.81
OLA	0.78
LIT+VAL	0.70
ARI+LAM	0.55
AOM	0.53
QUE	0.52
LIT	0.49
RISLAI	0.42
VAL	0.41
ARI	0.40
CAR	0.30
LAM	0.23
PAL	0.16
PLA	0.02

Comparison-adjusted funnel plot of studies of drugs versus placebo

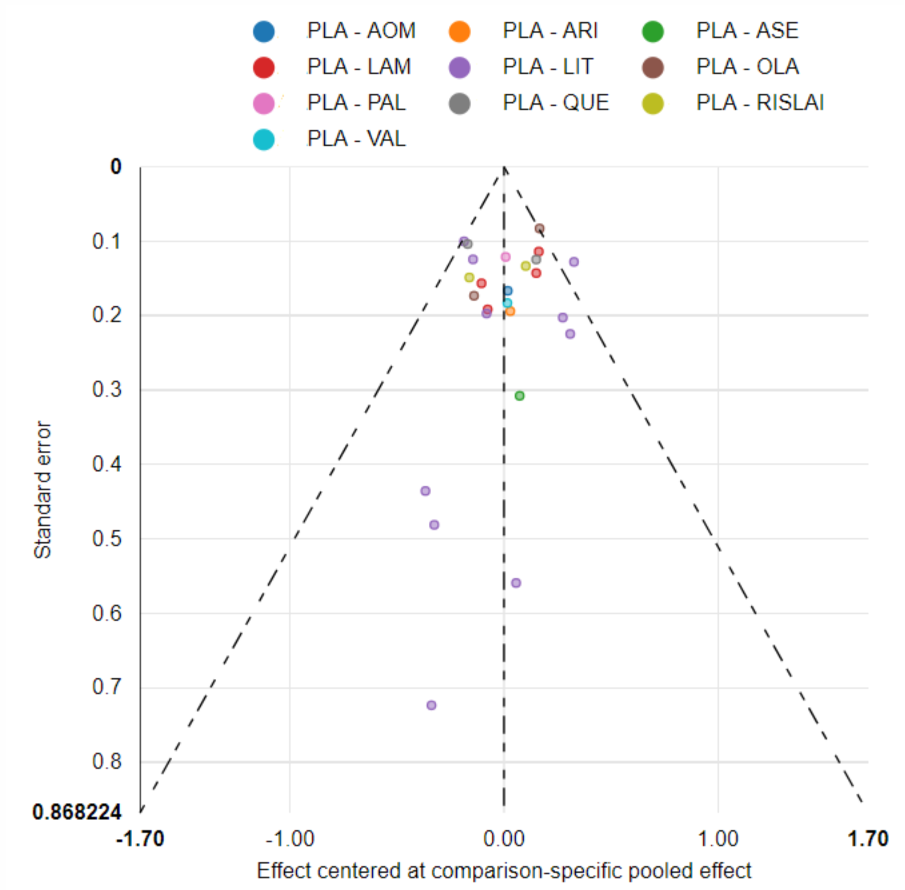

CINeMA confidence rating

(1) Within-study bias

We referred to the following article:

Furukawa TA, et al., Comparative efficacy and acceptability of first-generation and second-generation antidepressants in the acute treatment of major depression: protocol for a network meta-analysis. BMJ Open. 2016 Jul 8;6(7):e010919. doi: 10.1136/bmjopen-2015-010919.

(2) Reporting bias

Comparison-adjusted funnel plots with fewer than ten studies are not meaningful. Therefore, all comparisons were "Major concerns."

(3) Indirectness

No indirectness assumed.

(3) Imprecision

A clinically meaningful threshold was set at a risk ratio of higher or lower than 1.

(4) Heterogeneity

We used recommendations automatically provided by CINeMA.

(5) Incoherence

We used recommendations automatically provided by CINeMA.

Comparison	Number of studies	Within-study bias	Reporting bias	Indirectness	Imprecision	Heterogeneity	Incoherence	Confidence rating
AOM:PLA	1	No concerns	Suspected	No concerns	No concerns	No concerns	No concerns	Moderate
ARI:PLA	1	No concerns	Suspected	No concerns	No concerns	Major concerns	No concerns	Low
ARI+LAM:LAM	1	No concerns	Suspected	No concerns	Major concerns	No concerns	No concerns	Low
ARI+VAL:VAL	1	Some concerns	Suspected	No concerns	Major concerns	No concerns	No concerns	Very low
ASE:PLA	1	No concerns	Suspected	No concerns	No concerns	No concerns	No concerns	Moderate
CAR:LIT	3	Major concerns	Suspected	No concerns	Major concerns	No concerns	No concerns	Very low
LAM:LIT	2	Some concerns	Suspected	No concerns	Major concerns	No concerns	No concerns	Very low
LAM:PLA	4	Some concerns	Suspected	No concerns	No concerns	Major concerns	No concerns	Very low
LIT:LIT+OXC	1	No concerns	Suspected	No concerns	Major concerns	No concerns	No concerns	Low
LIT:LIT+VAL	1	Major concerns	Suspected	No concerns	Major concerns	No concerns	No concerns	Very low
LIT:OLA	1	No concerns	Suspected	No concerns	Major concerns	No concerns	No concerns	Low
LIT:PLA	10	Some concerns	Suspected	No concerns	No concerns	No concerns	No concerns	Low
LIT:QUE	1	Some concerns	Suspected	No concerns	Major concerns	No concerns	No concerns	Very low
LIT:VAL	3	Major concerns	Suspected	No concerns	Major concerns	No concerns	No concerns	Very low
LIT+VAL:VAL	1	Major concerns	Suspected	No concerns	Major concerns	No concerns	No concerns	Very low
OLA:PLA	2	No concerns	Suspected	No concerns	No concerns	No concerns	No concerns	Moderate
OLA:RISLAI	1	No concerns	Suspected	No concerns	Major concerns	No concerns	No concerns	Low
PAL:PLA	1	No concerns	Suspected	No concerns	Major concerns	No concerns	No concerns	Low
PLA:QUE	2	Some concerns	Suspected	No concerns	No concerns	No concerns	No concerns	Low
PLA:RISLAI	2	No concerns	Suspected	No concerns	No concerns	No concerns	No concerns	Moderate
PLA:VAL	1	No concerns	Suspected	No concerns	No concerns	No concerns	No concerns	Moderate
AOM:ARI	0	No concerns	Suspected	No concerns	Major concerns	No concerns	No concerns	Low
AOM:ARI+LAM	0	No concerns	Suspected	No concerns	Major concerns	No concerns	No concerns	Low
AOM:ARI+VAL	0	No concerns	Suspected	No concerns	Major concerns	No concerns	No concerns	Low
AOM:ASE	0	No concerns	Suspected	No concerns	Major concerns	No concerns	No concerns	Low
AOM:CAR	0	No concerns	Suspected	No concerns	Major concerns	No concerns	No concerns	Low
AOM:LAM	0	Some concerns	Suspected	No concerns	Major concerns	No concerns	No concerns	Very low
AOM:LIT	0	No concerns	Suspected	No concerns	Major concerns	No concerns	No concerns	Low
AOM:LIT+OXC	0	No concerns	Suspected	No concerns	Major concerns	No concerns	No concerns	Low
AOM:LIT+VAL	0	No concerns	Suspected	No concerns	Major concerns	No concerns	No concerns	Low
AOM:OLA	0	No concerns	Suspected	No concerns	Major concerns	No concerns	No concerns	Low
AOM:PAL	0	No concerns	Suspected	No concerns	Major concerns	No concerns	No concerns	Low

AOM:QUE	0	No concerns	Suspected	No concerns	Major concerns	No concerns	No concerns	Low
AOM:RISLAI	0	No concerns	Suspected	No concerns	Major concerns	No concerns	No concerns	Low
AOM:VAL	0	No concerns	Suspected	No concerns	Major concerns	No concerns	No concerns	Low
ARI:ARI+LAM	0	No concerns	Suspected	No concerns	Major concerns	No concerns	No concerns	Low
ARI:ARI+VAL	0	No concerns	Suspected	No concerns	Major concerns	No concerns	No concerns	Low
ARI:ASE	0	No concerns	Suspected	No concerns	No concerns	Major concerns	No concerns	Low
ARI:CAR	0	No concerns	Suspected	No concerns	Major concerns	No concerns	No concerns	Low
ARI:LAM	0	Some concerns	Suspected	No concerns	Major concerns	No concerns	No concerns	Very low
ARI:LIT	0	No concerns	Suspected	No concerns	Major concerns	No concerns	No concerns	Low
ARI:LIT+OXC	0	No concerns	Suspected	No concerns	Major concerns	No concerns	No concerns	Low
ARI:LIT+VAL	0	No concerns	Suspected	No concerns	Major concerns	No concerns	No concerns	Low
ARI:OLA	0	No concerns	Suspected	No concerns	Major concerns	No concerns	No concerns	Low
ARI:PAL	0	No concerns	Suspected	No concerns	Major concerns	No concerns	No concerns	Low
ARI:QUE	0	No concerns	Suspected	No concerns	Major concerns	No concerns	No concerns	Low
ARI:RISLAI	0	No concerns	Suspected	No concerns	Major concerns	No concerns	No concerns	Low
ARI:VAL	0	No concerns	Suspected	No concerns	Major concerns	No concerns	No concerns	Low
ARI+LAM:ARI+VAL	0	Some concerns	Suspected	No concerns	Major concerns	No concerns	No concerns	Very low
ARI+LAM:ASE	0	No concerns	Suspected	No concerns	Major concerns	No concerns	No concerns	Low
ARI+LAM:CAR	0	Some concerns	Suspected	No concerns	Major concerns	No concerns	No concerns	Very low
ARI+LAM:LIT	0	Some concerns	Suspected	No concerns	Major concerns	No concerns	No concerns	Very low
ARI+LAM:LIT+OXC	0	No concerns	Suspected	No concerns	Major concerns	No concerns	No concerns	Low
ARI+LAM:LIT+VAL	0	No concerns	Suspected	No concerns	Major concerns	No concerns	No concerns	Low
ARI+LAM:OLA	0	No concerns	Suspected	No concerns	Major concerns	No concerns	No concerns	Low
ARI+LAM:PAL	0	No concerns	Suspected	No concerns	Major concerns	No concerns	No concerns	Low
ARI+LAM:PLA	0	Some concerns	Suspected	No concerns	No concerns	No concerns	No concerns	Low
ARI+LAM:QUE	0	Some concerns	Suspected	No concerns	Major concerns	No concerns	No concerns	Very low
ARI+LAM:RISLAI	0	No concerns	Suspected	No concerns	Major concerns	No concerns	No concerns	Low
ARI+LAM:VAL	0	No concerns	Suspected	No concerns	Major concerns	No concerns	No concerns	Low
ARI+VAL:ASE	0	No concerns	Suspected	No concerns	Major concerns	No concerns	No concerns	Low
ARI+VAL:CAR	0	Some concerns	Suspected	No concerns	Major concerns	No concerns	No concerns	Very low
ARI+VAL:LAM	0	Some concerns	Suspected	No concerns	No concerns	Major concerns	No concerns	Very low
ARI+VAL:LIT	0	Some concerns	Suspected	No concerns	Major concerns	No concerns	No concerns	Very low
ARI+VAL:LIT+OXC	0	No concerns	Suspected	No concerns	Major concerns	No concerns	No concerns	Low

ARI+VAL:LIT+VAL	0	Major concerns	Suspected	No concerns	Major concerns	No concerns	No concerns	Very low
ARI+VAL:OLA	0	No concerns	Suspected	No concerns	Major concerns	No concerns	No concerns	Low
ARI+VAL:PAL	0	No concerns	Suspected	No concerns	No concerns	Major concerns	No concerns	Low
ARI+VAL:PLA	0	Some concerns	Suspected	No concerns	No concerns	No concerns	No concerns	Low
ARI+VAL:QUE	0	Some concerns	Suspected	No concerns	Major concerns	No concerns	No concerns	Very low
ARI+VAL:RISLAI	0	No concerns	Suspected	No concerns	Major concerns	No concerns	No concerns	Low
ASE:CAR	0	No concerns	Suspected	No concerns	No concerns	No concerns	No concerns	Moderate
ASE:LAM	0	Some concerns	Suspected	No concerns	No concerns	No concerns	No concerns	Low
ASE:LIT	0	No concerns	Suspected	No concerns	No concerns	No concerns	No concerns	Moderate
ASE:LIT+OXC	0	No concerns	Suspected	No concerns	Major concerns	No concerns	No concerns	Low
ASE:LIT+VAL	0	No concerns	Suspected	No concerns	Major concerns	No concerns	No concerns	Low
ASE:OLA	0	No concerns	Suspected	No concerns	Major concerns	No concerns	No concerns	Low
ASE:PAL	0	No concerns	Suspected	No concerns	No concerns	No concerns	No concerns	Moderate
ASE:QUE	0	No concerns	Suspected	No concerns	Major concerns	No concerns	No concerns	Low
ASE:RISLAI	0	No concerns	Suspected	No concerns	No concerns	No concerns	No concerns	Moderate
ASE:VAL	0	No concerns	Suspected	No concerns	No concerns	No concerns	No concerns	Moderate
CAR:LAM	0	Some concerns	Suspected	No concerns	Major concerns	No concerns	No concerns	Very low
CAR:LIT+OXC	0	No concerns	Suspected	No concerns	Major concerns	No concerns	No concerns	Low
CAR:LIT+VAL	0	Major concerns	Suspected	No concerns	Major concerns	No concerns	No concerns	Very low
CAR:OLA	0	No concerns	Suspected	No concerns	Major concerns	No concerns	No concerns	Low
CAR:PAL	0	No concerns	Suspected	No concerns	Major concerns	No concerns	No concerns	Low
CAR:PLA	0	Some concerns	Suspected	No concerns	Major concerns	No concerns	No concerns	Very low
CAR:QUE	0	Some concerns	Suspected	No concerns	Major concerns	No concerns	No concerns	Very low
CAR:RISLAI	0	No concerns	Suspected	No concerns	Major concerns	No concerns	No concerns	Low
CAR:VAL	0	Major concerns	Suspected	No concerns	Major concerns	No concerns	No concerns	Very low
LAM:LIT+OXC	0	Some concerns	Suspected	No concerns	Major concerns	No concerns	No concerns	Very low
LAM:LIT+VAL	0	Major concerns	Suspected	No concerns	Major concerns	No concerns	No concerns	Very low
LAM:OLA	0	No concerns	Suspected	No concerns	No concerns	Major concerns	No concerns	Low
LAM:PAL	0	Some concerns	Suspected	No concerns	Major concerns	No concerns	No concerns	Very low
LAM:QUE	0	Some concerns	Suspected	No concerns	No concerns	Major concerns	No concerns	Very low
LAM:RISLAI	0	No concerns	Suspected	No concerns	Major concerns	No concerns	No concerns	Low
LAM:VAL	0	Some concerns	Suspected	No concerns	Major concerns	No concerns	No concerns	Very low
LIT:PAL	0	No concerns	Suspected	No concerns	Major concerns	No concerns	No concerns	Low

LIT:RISLAI	0	No concerns	Suspected	No concerns	Major concerns	No concerns	No concerns	Low
LIT+OXC:LIT+VAL	0	No concerns	Suspected	No concerns	Major concerns	No concerns	No concerns	Low
LIT+OXC:OLA	0	No concerns	Suspected	No concerns	Major concerns	No concerns	No concerns	Low
LIT+OXC:PAL	0	No concerns	Suspected	No concerns	Major concerns	No concerns	No concerns	Low
LIT+OXC:PLA	0	No concerns	Suspected	No concerns	No concerns	No concerns	No concerns	Moderate
LIT+OXC:QUE	0	No concerns	Suspected	No concerns	Major concerns	No concerns	No concerns	Low
LIT+OXC:RISLAI	0	No concerns	Suspected	No concerns	Major concerns	No concerns	No concerns	Low
LIT+OXC:VAL	0	No concerns	Suspected	No concerns	Major concerns	No concerns	No concerns	Low
LIT+VAL:OLA	0	No concerns	Suspected	No concerns	Major concerns	No concerns	No concerns	Low
LIT+VAL:PAL	0	No concerns	Suspected	No concerns	Major concerns	No concerns	No concerns	Low
LIT+VAL:PLA	0	Major concerns	Suspected	No concerns	No concerns	No concerns	No concerns	Low
LIT+VAL:QUE	0	Major concerns	Suspected	No concerns	Major concerns	No concerns	No concerns	Very low
LIT+VAL:RISLAI	0	No concerns	Suspected	No concerns	Major concerns	No concerns	No concerns	Low
OLA:PAL	0	No concerns	Suspected	No concerns	No concerns	Major concerns	No concerns	Low
OLA:QUE	0	No concerns	Suspected	No concerns	Major concerns	No concerns	No concerns	Low
OLA:VAL	0	No concerns	Suspected	No concerns	Major concerns	No concerns	No concerns	Low
PAL:QUE	0	No concerns	Suspected	No concerns	No concerns	Major concerns	No concerns	Low
PAL:RISLAI	0	No concerns	Suspected	No concerns	Major concerns	No concerns	No concerns	Low
PAL:VAL	0	No concerns	Suspected	No concerns	Major concerns	No concerns	No concerns	Low
QUE:RISLAI	0	No concerns	Suspected	No concerns	Major concerns	No concerns	No concerns	Low
QUE:VAL	0	Some concerns	Suspected	No concerns	Major concerns	No concerns	No concerns	Very low
RISLAI:VAL	0	No concerns	Suspected	No concerns	Major concerns	No concerns	No concerns	Low

Meta-regression analysis

	Primary analysis	Publication year	Duration of study	Number of total patients	Percent female	Mean age
Heterogeneity SD	0.145	0.164	0.262	0.153	0.118	0.164
Relative change in the variance		+13%	+81%	+6%	-19%	+13%
β , median (95% CI)		0.076 (-0.220, 0.412)	-0.460 (-1.084, 0.306)	-0.066 (-0.244, 0.157)	0.253 (-0.065, 0.533)	-0.008 (-0.357, 0.300)
Interventions vs placebo	RR (95%CI)	RR (95%CI)	RR (95%CI)	RR (95%CI)	RR (95%CI)	RR (95%CI)
AOM	**0.519 (0.335, 0.803)**	**0.492 (0.285, 0.812)**	**0.302 (0.136, 0.697)**	**0.514 (0.313, 0.822)**	**0.509 (0.328, 0.776)**	**0.510 (0.308, 0.844)**
ARI	**0.619 (0.383, 0.999)**	0.601 (0.350, 1.017)	0.521 (0.198, 1.304)	0.603 (0.355, 1.008)	**0.546 (0.331, 0.900)**	0.608 (0.346, 1.029)
ARI+LAM	**0.530 (0.324, 0.868)**	**0.535 (0.304, 0.919)**	**0.303 (0.119, 0.747)**	**0.535 (0.311, 0.898)**	**0.545 (0.336, 0.879)**	**0.533 (0.304, 0.923)**
ARI+VAL	**0.292 (0.114, 0.748)**	**0.317 (0.120, 0.791)**	**0.145 (0.034, 0.519)**	**0.318 (0.115, 0.792)**	**0.334 (0.129, 0.786)**	**0.320 (0.120, 0.788)**
ASE	**0.262 (0.133, 0.517)**	**0.243 (0.109, 0.476)**	**0.138 (0.048, 0.387)**	**0.253 (0.119, 0.498)**	**0.258 (0.125, 0.489)**	**0.251 (0.118, 0.504)**
CAR	0.684 (0.442, 1.057)	0.761 (0.490, 1.211)	0.502 (0.247, 1.066)	0.765 (0.499, 1.189)	0.787 (0.531, 1.188)	0.748 (0.484, 1.203)
LAM	**0.764 (0.628, 0.930)**	**0.754 (0.599, 0.939)**	**0.507 (0.325, 0.777)**	**0.763 (0.609, 0.940)**	**0.777 (0.635, 0.935)**	**0.756 (0.600, 0.941)**
LIT	**0.624 (0.537, 0.725)**	**0.620 (0.517, 0.743)**	**0.379 (0.270, 0.504)**	**0.626 (0.522, 0.734)**	**0.649 (0.549, 0.750)**	**0.611 (0.514, 0.722)**
LIT+OXC	**0.409 (0.212, 0.792)**	**0.387 (0.184, 0.777)**	**0.161 (0.042, 0.593)**	**0.396 (0.188, 0.770)**	**0.401 (0.197, 0.778)**	**0.388 (0.184, 0.766)**
LIT+VAL	**0.525 (0.363, 0.760)**	**0.521 (0.334, 0.799)**	**0.253 (0.108, 0.556)**	**0.525 (0.342, 0.775)**	**0.546 (0.371, 0.773)**	**0.514 (0.331, 0.779)**
OLA	**0.500 (0.400, 0.625)**	**0.489 (0.371, 0.621)**	**0.227 (0.141, 0.355)**	**0.506 (0.379, 0.636)**	**0.505 (0.398, 0.617)**	**0.493 (0.365, 0.635)**
PAL	0.835 (0.575, 1.212)	0.801 (0.502, 1.264)	1.306 (0.365, 4.010)	0.833 (0.545, 1.259)	0.858 (0.586, 1.242)	0.829 (0.524, 1.289)
QUE	**0.526 (0.411, 0.674)**	**0.508 (0.377, 0.690)**	**0.362 (0.222, 0.574)**	**0.557 (0.388, 0.774)**	**0.526 (0.413, 0.671)**	**0.519 (0.382, 0.699)**
RISLAI	**0.637 (0.484, 0.839)**	**0.614 (0.447, 0.858)**	**0.461 (0.255, 0.801)**	**0.638 (0.472, 0.874)**	**0.672 (0.504, 0.883)**	**0.627 (0.444, 0.884)**
VAL	**0.634 (0.485, 0.829)**	**0.625 (0.455, 0.844)**	**0.414 (0.228, 0.681)**	**0.633 (0.461, 0.839)**	**0.665 (0.491, 0.858)**	**0.618 (0.450, 0.832)**

Sensitivity analysis

Model fit statistics	Primary analysis	Focusing on studies including only BDI	Focusing on studies including only non rapid-cycling BD patients	Focusing on double-blind studies	Focusing on non-enriched studies	Focusing on non-industry sponsorship studies
Heterogeneity SD	0.145	0.142	0.103	0.147	0.115	0.103
Relative change in the variance		-2%	-29%	+1%	-21%	-29%
Interventions vs placebo	RR (95%CI)	RR (95%CI)	RR (95%CI)	RR (95%CI)	RR (95%CI)	RR (95%CI)
AOM	**0.519 (0.335, 0.803)**	**0.514 (0.324, 0.816)**	**0.506 (0.334, 0.770)**	**0.514 (0.319, 0.808)**	**0.507 (0.281, 0.880)**	**0.506 (0.289, 0.848)**
ARI	**0.619 (0.383, 0.999)**	**0.607 (0.364, 0.994)**	0.598 (0.310, 1.062)	0.609 (0.356, 1.011)	0.601 (0.309, 1.082)	0.597 (0.305, 1.107)
ARI+LAM	**0.530 (0.324, 0.868)**	**0.531 (0.315, 0.882)**	**0.543 (0.293, 0.982)**	**0.539 (0.317, 0.931)**	**0.548 (0.293, 0.996)**	**0.545 (0.294, 0.988)**
ARI+VAL	**0.292 (0.114, 0.748)**	**0.320 (0.117, 0.780)**	0.363 (0.099, 1.136)	**0.316 (0.113, 0.781)**	0.366 (0.092, 1.077)	0.351 (0.086, 1.125)
ASE	**0.262 (0.133, 0.517)**	**0.251 (0.115, 0.480)**	**0.251 (0.121, 0.473)**	**0.250 (0.117, 0.493)**	**0.246 (0.088, 0.577)**	**0.246 (0.090, 0.576)**
CAR	0.684 (0.442, 1.057)	0.766 (0.448, 1.349)	0.759 (0.440, 1.307)	0.798 (0.501, 1.306)	0.749 (0.505, 1.132)	0.670 (0.444, 1.028)
LAM	**0.764 (0.628, 0.930)**	**0.753 (0.601, 0.927)**	**0.764 (0.602, 0.950)**	**0.762 (0.614, 0.930)**	**0.767 (0.599, 0.954)**	**0.767 (0.600, 0.953)**
LIT	**0.624 (0.537, 0.725)**	**0.618 (0.522, 0.724)**	**0.604 (0.504, 0.723)**	**0.613 (0.521, 0.717)**	**0.618 (0.520, 0.728)**	**0.599 (0.506, 0.701)**
LIT+OXC	**0.409 (0.212, 0.792)**	**0.378 (0.136, 0.889)**	**0.363 (0.130, 0.833)**	**0.389 (0.187, 0.749)**	**0.385 (0.190, 0.729)**	**0.379 (0.193, 0.719)**
LIT+VAL	**0.525 (0.363, 0.760)**	**0.520 (0.348, 0.781)**	**0.511 (0.331, 0.774)**	**0.507 (0.317, 0.780)**	**0.525 (0.357, 0.749)**	**0.512 (0.353, 0.729)**
OLA	**0.500 (0.400, 0.625)**	**0.498 (0.386, 0.625)**	**0.493 (0.383, 0.614)**	**0.498 (0.381, 0.624)**	**0.504 (0.389, 0.634)**	**0.503 (0.389, 0.640)**
PAL	0.835 (0.575, 1.212)	0.825 (0.555, 1.219)	0.827 (0.579, 1.186)	0.828 (0.551, 1.249)	0.827 (0.530, 1.268)	0.817 (0.532, 1.254)
QUE	**0.526 (0.411, 0.674)**	**0.508 (0.384, 0.688)**	**0.508 (0.386, 0.678)**	**0.520 (0.396, 0.682)**	**0.514 (0.384, 0.689)**	**0.510 (0.384, 0.675)**
RISLAI	**0.637 (0.484, 0.839)**	**0.633 (0.468, 0.839)**	**0.624 (0.481, 0.804)**	**0.632 (0.469, 0.850)**	**0.629 (0.446, 0.873)**	**0.623 (0.450, 0.854)**
VAL	**0.634 (0.485, 0.829)**	**0.634 (0.460, 0.830)**	**0.625 (0.456, 0.852)**	**0.611 (0.448, 0.814)**	**0.635 (0.472, 0.825)**	**0.629 (0.469, 0.817)**

1.2. Recurrence/relapse of depressive episodes (25 RCTs, 6438 patients)

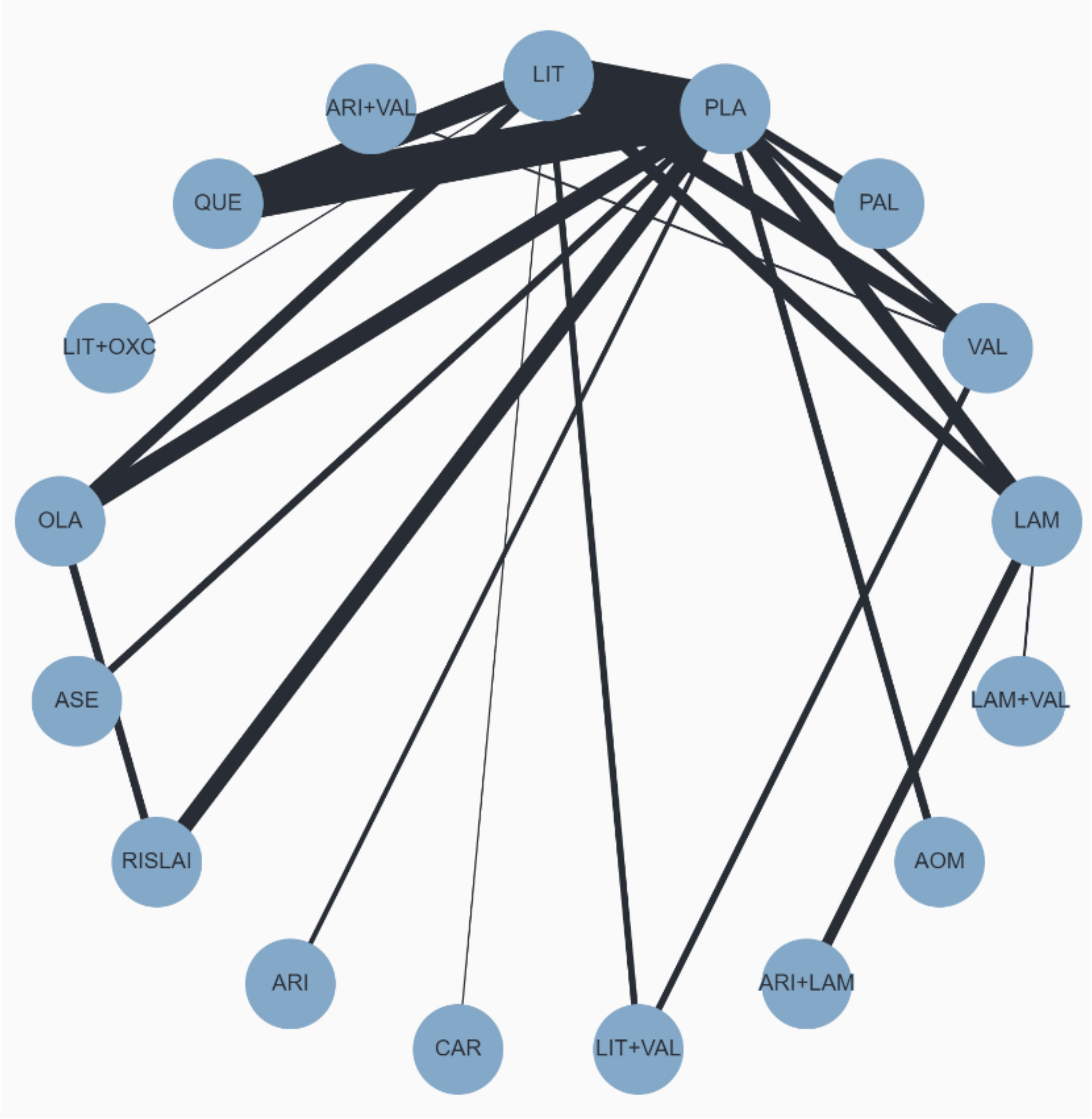

League table

Results from pairwise meta-analysis are presented in the left lower half and results from network meta-analysis in the upper right half.

The boldface result indicates statistical significance.

AOM	1.178 (0.440, 3.153)	1.849 (0.782, 4.368)	3.879 (0.935, 16.087)	2.758 (0.837, 9.091)	0.389 (0.080, 1.892)	1.487 (0.761, 2.905)	**2.258 (1.006, 5.065)**	1.341 (0.706, 2.546)	3.606 (0.915, 14.208)	1.431 (0.675, 3.034)	1.430 (0.728, 2.808)	0.809 (0.367, 1.786)	**2.212 (1.127, 4.342)**	0.824 (0.394, 1.722)	1.251 (0.616, 2.541)	1.061 (0.573, 1.962)
	ARI	1.569 (0.591, 4.164)	3.293 (0.738, 14.690)	2.341 (0.652, 8.412)	0.330 (0.063, 1.716)	1.262 (0.560, 2.847)	1.917 (0.756, 4.860)	1.138 (0.517, 2.508)	3.061 (0.720, 13.008)	1.215 (0.503, 2.935)	1.214 (0.536, 2.749)	0.687 (0.275, 1.717)	1.878 (0.829, 4.252)	0.699 (0.293, 1.669)	1.062 (0.456, 2.474)	0.900 (0.417, 1.942)
		ARI+LAM	2.098 (0.513, 8.587)	1.492 (0.456, 4.882)	0.210 (0.044, 1.010)	0.804 (0.469, 1.379)	1.221 (0.604, 2.468)	0.725 (0.395, 1.333)	1.951 (0.503, 7.572)	0.774 (0.375, 1.599)	0.773 (0.401, 1.493)	**0.438 (0.201, 0.955)**	1.196 (0.620, 2.311)	**0.446 (0.216, 0.920)**	0.677 (0.342, 1.338)	0.574 (0.315, 1.046)
			ARI+VAL	0.711 (0.138, 3.666)	**0.100 (0.015, 0.689)**	0.383 (0.104, 1.409)	0.582 (0.147, 2.310)	0.346 (0.097, 1.235)	0.930 (0.160, 5.392)	0.369 (0.102, 1.338)	0.369 (0.100, 1.363)	**0.209 (0.053, 0.826)**	0.570 (0.154, 2.109)	**0.212 (0.055, 0.815)**	0.322 (0.094, 1.107)	**0.273 (0.076, 0.986)**
				ASE	**0.141 (0.024, 0.837)**	0.539 (0.188, 1.550)	0.819 (0.260, 2.582)	0.486 (0.172, 1.373)	1.308 (0.265, 6.450)	0.519 (0.171, 1.574)	0.518 (0.180, 1.495)	**0.293 (0.094, 0.915)**	0.802 (0.278, 2.313)	**0.299 (0.099, 0.898)**	0.454 (0.154, 1.337)	0.385 (0.138, 1.069)
					CAR	3.826 (0.876, 16.715)	**5.810 (1.243, 27.161)**	3.450 (0.812, 14.667)	**9.279 (1.405, 61.283)**	3.683 (0.821, 16.524)	3.679 (0.838, 16.158)	2.082 (0.446, 9.725)	**5.692 (1.295, 25.011)**	2.120 (0.467, 9.627)	3.219 (0.732, 14.160)	2.729 (0.635, 11.735)
		0.804 (0.469, 1.379)				LAM	1.518 (0.966, 2.386)	0.902 (0.680, 1.196)	2.425 (0.699, 8.418)	0.963 (0.593, 1.563)	0.962 (0.660, 1.402)	**0.544 (0.309, 0.957)**	**1.488 (1.020, 2.170)**	**0.554 (0.342, 0.899)**	0.841 (0.554, 1.277)	**0.713 (0.547, 0.930)**
						1.518 (0.966, 2.386)	LAM+VAL	0.594 (0.349, 1.012)	1.597 (0.425, 6.003)	0.634 (0.327, 1.230)	0.633 (0.351, 1.141)	**0.358 (0.174, 0.739)**	0.980 (0.544, 1.765)	**0.365 (0.188, 0.708)**	0.554 (0.300, 1.025)	**0.470 (0.278, 0.793)**

					3.450 (0.812, 14.667)	0.832 (0.591, 1.171)		LIT	2.690 (0.800, 9.039)	1.067 (0.716, 1.590)	1.066 (0.783, 1.452)	0.604 (0.355, 1.026)	**1.650 (1.208, 2.252)**	**0.615 (0.395, 0.956)**	0.933 (0.680, 1.280)	**0.791 (0.660, 0.948)**
								2.690 (0.800, 9.039)	LIT+OXC	0.397 (0.111, 1.422)	0.396 (0.114, 1.385)	**0.224 (0.060, 0.842)**	0.613 (0.175, 2.144)	**0.229 (0.063, 0.830)**	0.347 (0.099, 1.214)	0.294 (0.086, 1.002)
								0.897 (0.587, 1.372)		LIT+VAL	0.999 (0.606, 1.647)	0.565 (0.292, 1.093)	1.546 (0.936, 2.552)	0.576 (0.319, 1.039)	0.874 (0.603, 1.268)	0.741 (0.481, 1.142)
											OLA	0.566 (0.320, 1.001)	**1.547 (1.049, 2.282)**	**0.576 (0.365, 0.910)**	0.875 (0.567, 1.350)	**0.742 (0.562, 0.979)**
												PAL	**2.734 (1.546, 4.834)**	1.018 (0.535, 1.937)	1.546 (0.840, 2.846)	1.311 (0.796, 2.157)
													QUE	**0.373 (0.228, 0.609)**	**0.566 (0.366, 0.874)**	**0.480 (0.364, 0.633)**
														RISLAI	1.518 (0.889, 2.593)	1.287 (0.857, 1.932)
			0.322 (0.094, 1.107)							0.780 (0.531, 1.145)					VAL	0.848 (0.596, 1.206)
1.061 (0.573, 1.962)	0.900 (0.417, 1.942)			0.385 (0.138, 1.069)		**0.748 (0.563, 0.992)**									**0.402 (0.191, 0.848)**	PLA

Evaluation of heterogeneity and inconsistency

Between study variance (τ^2)	Heterogeneity assessment	Random-effects design-by-treatment interaction model		
		Q	df	p
0.011	Low	12.796	10	0.235

Inconsistency

	NMA, RR (95%CI)	Direct, RR (95%CI)	Indirect, RR (95%CI)	Inconsistency measures	
				Ratio of risk ratios	P value
LAM vs LIT	0.902 (0.680, 1.196)	0.832 (0.591, 1.171)	1.069 (0.650, 1.759)	0.778 (0.425, 1.423)	0.415
LAM vs PLA	0.713 (0.547, 0.930)	0.748 (0.563, 0.992)	0.511 (0.240, 1.088)	1.463 (0.653, 3.281)	0.355
**LIT vs LIT+VAL**	**1.067 (0.716, 1.590)**	**0.897 (0.587, 1.372)**	**3.875 (1.217, 12.333)**	**0.232 (0.067, 0.795)**	**0.020**
LIT vs OLA	1.066 (0.783, 1.452)	0.972 (0.546, 1.731)	1.107 (0.768, 1.594)	0.878 (0.443, 1.739)	0.709
LIT vs QUE	1.650 (1.208, 2.252)	1.511 (0.958, 2.381)	1.783 (1.163, 2.733)	0.847 (0.454, 1.582)	0.603
LIT vs VAL	0.933 (0.680, 1.280)	0.865 (0.623, 1.200)	2.585 (0.777, 8.601)	0.335 (0.096, 1.163)	0.085
LIT vs PLA	0.791 (0.660, 0.948)	0.828 (0.681, 1.006)	0.593 (0.362, 0.970)	1.397 (0.822, 2.373)	0.216
**LIT+VAL vs VAL**	**0.874 (0.603, 1.268)**	**0.780 (0.531, 1.145)**	**4.823 (1.090, 21.336)**	**0.162 (0.035, 0.751)**	**0.020**
OLA vs RISLAI	0.576 (0.365, 0.910)	0.484 (0.246, 0.951)	0.668 (0.359, 1.241)	0.724 (0.289, 1.813)	0.491
OLA vs PLA	0.742 (0.562, 0.979)	0.718 (0.524, 0.984)	0.832 (0.461, 1.501)	0.863 (0.442, 1.684)	0.666
QUE vs PLA	0.480 (0.364, 0.633)	0.463 (0.349, 0.615)	0.978 (0.273, 3.497)	0.474 (0.128, 1.748)	0.262
RISLAI vs PLA	1.287 (0.857, 1.932)	1.212 (0.791, 1.857)	2.304 (0.611, 8.686)	0.526 (0.130, 2.120)	0.366
**VAL vs PLA**	**0.848 (0.596, 1.206)**	**0.402 (0.191, 0.848)**	**1.050 (0.704, 1.566)**	**0.383 (0.164, 0.893)**	**0.026**

Rank probabilities

	Rank 1	Rank 2	Rank 3	Rank 4	Rank 5	Rank 6	Rank 7	Rank 8	Rank 9	Rank 10	Rank 11	Rank 12	Rank 13	Rank 14	Rank 15	Rank 16	Rank 17
AOM	0.000	0.002	0.005	0.009	0.019	0.023	0.033	0.038	0.043	0.049	0.062	0.097	0.124	0.157	0.161	0.151	0.028
ARI	0.007	0.014	0.027	0.039	0.056	0.064	0.069	0.057	0.057	0.059	0.069	0.092	0.089	0.103	0.093	0.087	0.019
ARI+LAM	0.013	0.041	0.090	0.121	0.148	0.153	0.100	0.075	0.052	0.046	0.048	0.043	0.028	0.023	0.013	0.006	0.001
ARI+VAL	0.293	0.259	0.154	0.086	0.053	0.040	0.023	0.016	0.016	0.011	0.014	0.008	0.007	0.007	0.006	0.004	0.002
ASE	0.211	0.252	0.192	0.096	0.066	0.046	0.032	0.021	0.014	0.016	0.016	0.014	0.009	0.007	0.005	0.004	0.000
CAR	0.000	0.000	0.001	0.001	0.002	0.001	0.002	0.003	0.003	0.004	0.004	0.008	0.010	0.018	0.036	0.070	0.838
LAM	0.000	0.000	0.002	0.010	0.043	0.117	0.200	0.192	0.164	0.131	0.079	0.043	0.016	0.004	0.001	0.000	0.000
LAM+VAL	0.042	0.125	0.211	0.235	0.161	0.094	0.050	0.027	0.019	0.011	0.010	0.008	0.004	0.003	0.001	0.001	0.000
LIT	0.000	0.000	0.000	0.001	0.005	0.017	0.048	0.116	0.201	0.247	0.213	0.118	0.029	0.005	0.001	0.000	0.000
LIT+OXC	0.426	0.246	0.127	0.062	0.046	0.030	0.016	0.012	0.009	0.007	0.006	0.005	0.003	0.003	0.002	0.001	0.000
LIT+VAL	0.001	0.006	0.017	0.044	0.074	0.115	0.128	0.126	0.114	0.107	0.103	0.074	0.041	0.029	0.017	0.006	0.001
OLA	0.000	0.002	0.004	0.015	0.048	0.100	0.143	0.165	0.148	0.130	0.122	0.082	0.032	0.009	0.003	0.000	0.000
PAL	0.000	0.000	0.000	0.001	0.003	0.004	0.008	0.009	0.012	0.018	0.025	0.042	0.078	0.137	0.238	0.359	0.067
QUE	0.008	0.054	0.167	0.273	0.249	0.140	0.056	0.029	0.014	0.005	0.003	0.002	0.000	0.000	0.000	0.000	0.000
RISLAI	0.000	0.000	0.000	0.000	0.000	0.001	0.004	0.004	0.007	0.011	0.023	0.044	0.091	0.166	0.311	0.293	0.044
VAL	0.000	0.001	0.003	0.008	0.028	0.057	0.087	0.111	0.129	0.140	0.161	0.135	0.077	0.039	0.020	0.006	0.000
PLA	0.000	0.000	0.000	0.000	0.000	0.000	0.000	0.000	0.002	0.009	0.042	0.185	0.365	0.294	0.091	0.013	0.000

SUCRA

ARI+VAL	0.89
LIT+OXC	0.88
LAM+VAL	0.88
QUE	0.75
ASE	0.74
ARI+LAM	0.68
LAM	0.57
LIT+VAL	0.54
OLA	0.50
LIT	0.47
VAL	0.44
ARI	0.34
AOM	0.25
PLA	0.24
RISLAI	0.14
PAL	0.14
CAR	0.05

Comparison-adjusted funnel plot of studies of drugs versus placebo

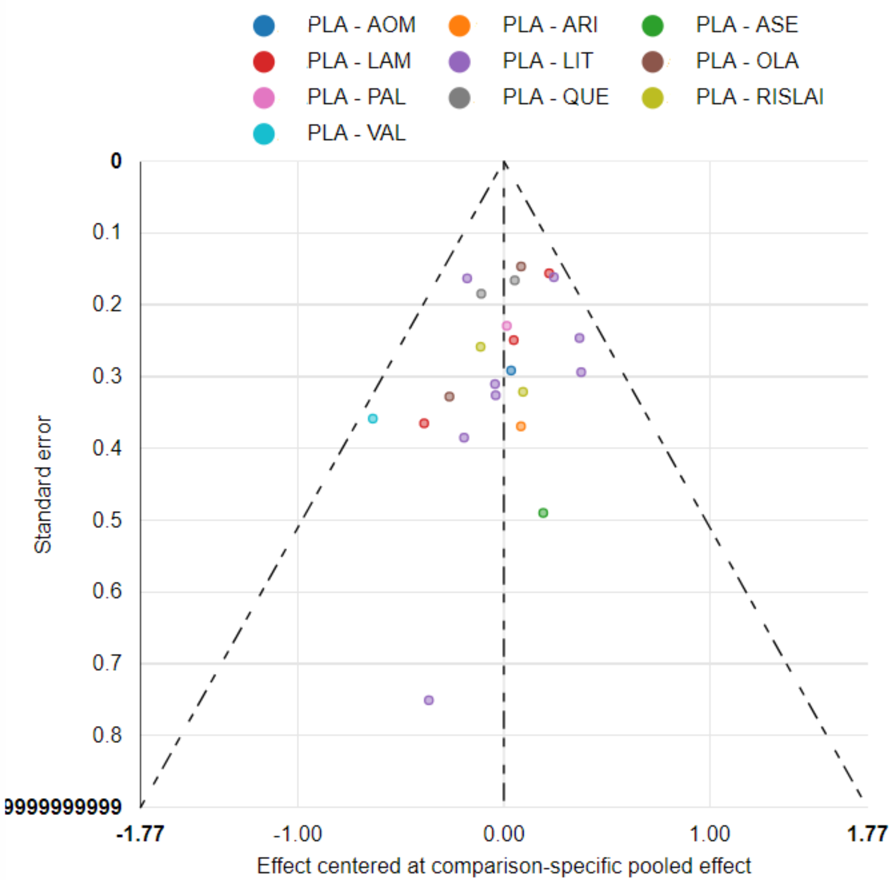

CINeMA rating

Comparison	Number of studies	Within-study bias	Reporting bias	Indirectness	Imprecision	Heterogeneity	Incoherence	Confidence rating
AOM:PLA	1	No concerns	Suspected	No concerns	Major concerns	No concerns	No concerns	Low
ARI:PLA	1	No concerns	Suspected	No concerns	Major concerns	No concerns	No concerns	Low
ARI+LAM:LAM	1	No concerns	Suspected	No concerns	Major concerns	No concerns	No concerns	Low
ARI+VAL:VAL	1	Some concerns	Suspected	No concerns	Major concerns	No concerns	No concerns	Very low
ASE:PLA	1	No concerns	Suspected	No concerns	Major concerns	No concerns	No concerns	Low
CAR:LIT	1	No concerns	Suspected	No concerns	Major concerns	No concerns	No concerns	Low
LAM:LAM+VAL	1	Some concerns	Suspected	No concerns	Major concerns	No concerns	No concerns	Very low
LAM:LIT	2	Some concerns	Suspected	No concerns	Major concerns	No concerns	No concerns	Very low
LAM:PLA	3	Some concerns	Suspected	No concerns	No concerns	Major concerns	No concerns	Very low
LIT:LIT+OXC	1	No concerns	Suspected	No concerns	Major concerns	No concerns	No concerns	Low
LIT:LIT+VAL	1	Major concerns	Suspected	No concerns	Major concerns	No concerns	Major concerns	Very low
LIT:OLA	1	No concerns	Suspected	No concerns	Major concerns	No concerns	No concerns	Low
LIT:PLA	8	Some concerns	Suspected	No concerns	No concerns	Major concerns	No concerns	Very low
LIT:QUE	1	Some concerns	Suspected	No concerns	No concerns	No concerns	No concerns	Low
LIT:VAL	3	Major concerns	Suspected	No concerns	Major concerns	No concerns	No concerns	Very low
LIT+VAL:VAL	1	Major concerns	Suspected	No concerns	Major concerns	No concerns	Major concerns	Very low
OLA:PLA	2	No concerns	Suspected	No concerns	No concerns	Major concerns	No concerns	Low
OLA:RISLAI	1	No concerns	Suspected	No concerns	No concerns	No concerns	No concerns	Moderate
PAL:PLA	1	No concerns	Suspected	No concerns	Major concerns	No concerns	No concerns	Low
PLA:QUE	2	Some concerns	Suspected	No concerns	No concerns	No concerns	No concerns	Low
PLA:RISLAI	2	No concerns	Suspected	No concerns	Major concerns	No concerns	No concerns	Low
PLA:VAL	1	No concerns	Suspected	No concerns	Major concerns	No concerns	Major concerns	Very low
AOM:ARI	0	No concerns	Suspected	No concerns	Major concerns	No concerns	No concerns	Low
AOM:ARI+LAM	0	No concerns	Suspected	No concerns	Major concerns	No concerns	No concerns	Low
AOM:ARI+VAL	0	No concerns	Suspected	No concerns	Major concerns	No concerns	No concerns	Low
AOM:ASE	0	No concerns	Suspected	No concerns	Major concerns	No concerns	No concerns	Low
AOM:CAR	0	No concerns	Suspected	No concerns	Major concerns	No concerns	No concerns	Low
AOM:LAM	0	Some concerns	Suspected	No concerns	Major concerns	No concerns	No concerns	Very low
AOM:LAM+VAL	0	Some concerns	Suspected	No concerns	No concerns	Major concerns	No concerns	Very low
AOM:LIT	0	No concerns	Suspected	No concerns	Major concerns	No concerns	No concerns	Low
AOM:LIT+OXC	0	No concerns	Suspected	No concerns	Major concerns	No concerns	No concerns	Low
AOM:LIT+VAL	0	No concerns	Suspected	No concerns	Major concerns	No concerns	No concerns	Low

AOM:OLA	0	No concerns	Suspected	No concerns	Major concerns	No concerns	No concerns	Low
AOM:PAL	0	No concerns	Suspected	No concerns	Major concerns	No concerns	No concerns	Low
AOM:QUE	0	No concerns	Suspected	No concerns	No concerns	No concerns	No concerns	Moderate
AOM:RISLAI	0	No concerns	Suspected	No concerns	Major concerns	No concerns	No concerns	Low
AOM:VAL	0	No concerns	Suspected	No concerns	Major concerns	No concerns	No concerns	Low
ARI:ARI+LAM	0	No concerns	Suspected	No concerns	Major concerns	No concerns	No concerns	Low
ARI:ARI+VAL	0	No concerns	Suspected	No concerns	Major concerns	No concerns	No concerns	Low
ARI:ASE	0	No concerns	Suspected	No concerns	Major concerns	No concerns	No concerns	Low
ARI:CAR	0	No concerns	Suspected	No concerns	Major concerns	No concerns	No concerns	Low
ARI:LAM	0	Some concerns	Suspected	No concerns	Major concerns	No concerns	No concerns	Very low
ARI:LAM+VAL	0	Some concerns	Suspected	No concerns	Major concerns	No concerns	No concerns	Very low
ARI:LIT	0	No concerns	Suspected	No concerns	Major concerns	No concerns	No concerns	Low
ARI:LIT+OXC	0	No concerns	Suspected	No concerns	Major concerns	No concerns	No concerns	Low
ARI:LIT+VAL	0	No concerns	Suspected	No concerns	Major concerns	No concerns	No concerns	Low
ARI:OLA	0	No concerns	Suspected	No concerns	Major concerns	No concerns	No concerns	Low
ARI:PAL	0	No concerns	Suspected	No concerns	Major concerns	No concerns	No concerns	Low
ARI:QUE	0	No concerns	Suspected	No concerns	Major concerns	No concerns	No concerns	Low
ARI:RISLAI	0	No concerns	Suspected	No concerns	Major concerns	No concerns	No concerns	Low
ARI:VAL	0	No concerns	Suspected	No concerns	Major concerns	No concerns	No concerns	Low
ARI+LAM:ARI+VAL	0	Some concerns	Suspected	No concerns	Major concerns	No concerns	No concerns	Very low
ARI+LAM:ASE	0	No concerns	Suspected	No concerns	Major concerns	No concerns	No concerns	Low
ARI+LAM:CAR	0	No concerns	Suspected	No concerns	Major concerns	No concerns	No concerns	Low
ARI+LAM:LAM+VAL	0	No concerns	Suspected	No concerns	Major concerns	No concerns	No concerns	Low
ARI+LAM:LIT	0	Some concerns	Suspected	No concerns	Major concerns	No concerns	No concerns	Very low
ARI+LAM:LIT+OXC	0	No concerns	Suspected	No concerns	Major concerns	No concerns	No concerns	Low
ARI+LAM:LIT+VAL	0	No concerns	Suspected	No concerns	Major concerns	No concerns	No concerns	Low
ARI+LAM:OLA	0	No concerns	Suspected	No concerns	Major concerns	No concerns	No concerns	Low
ARI+LAM:PAL	0	No concerns	Suspected	No concerns	No concerns	Major concerns	No concerns	Low
ARI+LAM:PLA	0	Some concerns	Suspected	No concerns	Major concerns	No concerns	No concerns	Very low
ARI+LAM:QUE	0	Some concerns	Suspected	No concerns	Major concerns	No concerns	No concerns	Very low
ARI+LAM:RISLAI	0	No concerns	Suspected	No concerns	No concerns	Major concerns	No concerns	Low
ARI+LAM:VAL	0	No concerns	Suspected	No concerns	Major concerns	No concerns	No concerns	Low
ARI+VAL:ASE	0	No concerns	Suspected	No concerns	Major concerns	No concerns	No concerns	Low

ARI+VAL:CAR	0	No concerns	Suspected	No concerns	No concerns	No concerns	No concerns	Moderate
ARI+VAL:LAM	0	Some concerns	Suspected	No concerns	Major concerns	No concerns	No concerns	Very low
ARI+VAL:LAM+VAL	0	Some concerns	Suspected	No concerns	Major concerns	No concerns	No concerns	Very low
ARI+VAL:LIT	0	Some concerns	Suspected	No concerns	Major concerns	No concerns	No concerns	Very low
ARI+VAL:LIT+OXC	0	No concerns	Suspected	No concerns	Major concerns	No concerns	No concerns	Low
ARI+VAL:LIT+VAL	0	Major concerns	Suspected	No concerns	Major concerns	No concerns	No concerns	Very low
ARI+VAL:OLA	0	No concerns	Suspected	No concerns	Major concerns	No concerns	No concerns	Low
ARI+VAL:PAL	0	No concerns	Suspected	No concerns	No concerns	No concerns	No concerns	Moderate
ARI+VAL:PLA	0	Some concerns	Suspected	No concerns	No concerns	Major concerns	No concerns	Very low
ARI+VAL:QUE	0	Some concerns	Suspected	No concerns	Major concerns	No concerns	No concerns	Very low
ARI+VAL:RISLAI	0	No concerns	Suspected	No concerns	No concerns	No concerns	No concerns	Moderate
ASE:CAR	0	No concerns	Suspected	No concerns	No concerns	Major concerns	No concerns	Low
ASE:LAM	0	Some concerns	Suspected	No concerns	Major concerns	No concerns	No concerns	Very low
ASE:LAM+VAL	0	Some concerns	Suspected	No concerns	Major concerns	No concerns	No concerns	Very low
ASE:LIT	0	No concerns	Suspected	No concerns	Major concerns	No concerns	No concerns	Low
ASE:LIT+OXC	0	No concerns	Suspected	No concerns	Major concerns	No concerns	No concerns	Low
ASE:LIT+VAL	0	No concerns	Suspected	No concerns	Major concerns	No concerns	No concerns	Low
ASE:OLA	0	No concerns	Suspected	No concerns	Major concerns	No concerns	No concerns	Low
ASE:PAL	0	No concerns	Suspected	No concerns	No concerns	Major concerns	No concerns	Low
ASE:QUE	0	No concerns	Suspected	No concerns	Major concerns	No concerns	No concerns	Low
ASE:RISLAI	0	No concerns	Suspected	No concerns	No concerns	Major concerns	No concerns	Low
ASE:VAL	0	No concerns	Suspected	No concerns	Major concerns	No concerns	No concerns	Low
CAR:LAM	0	Some concerns	Suspected	No concerns	Major concerns	No concerns	No concerns	Very low
CAR:LAM+VAL	0	Some concerns	Suspected	No concerns	No concerns	No concerns	No concerns	Low
CAR:LIT+OXC	0	No concerns	Suspected	No concerns	No concerns	No concerns	No concerns	Moderate
CAR:LIT+VAL	0	Major concerns	Suspected	No concerns	Major concerns	No concerns	No concerns	Very low
CAR:OLA	0	No concerns	Suspected	No concerns	Major concerns	No concerns	No concerns	Low
CAR:PAL	0	No concerns	Suspected	No concerns	Major concerns	No concerns	No concerns	Low
CAR:PLA	0	No concerns	Suspected	No concerns	Major concerns	No concerns	No concerns	Low
CAR:QUE	0	No concerns	Suspected	No concerns	No concerns	No concerns	No concerns	Moderate
CAR:RISLAI	0	No concerns	Suspected	No concerns	Major concerns	No concerns	No concerns	Low
CAR:VAL	0	No concerns	Suspected	No concerns	Major concerns	No concerns	No concerns	Low
LAM:LIT+OXC	0	Some concerns	Suspected	No concerns	Major concerns	No concerns	No concerns	Very low

LAM:LIT+VAL	0	Some concerns	Suspected	No concerns	Major concerns	No concerns	No concerns	Very low
LAM:OLA	0	No concerns	Suspected	No concerns	Major concerns	No concerns	No concerns	Low
LAM:PAL	0	Some concerns	Suspected	No concerns	No concerns	Major concerns	No concerns	Very low
LAM:QUE	0	Some concerns	Suspected	No concerns	No concerns	Major concerns	No concerns	Very low
LAM:RISLAI	0	No concerns	Suspected	No concerns	No concerns	No concerns	No concerns	Moderate
LAM:VAL	0	Some concerns	Suspected	No concerns	Major concerns	No concerns	No concerns	Very low
LAM+VAL:LIT	0	Some concerns	Suspected	No concerns	Major concerns	No concerns	No concerns	Very low
LAM+VAL:LIT+OXC	0	Some concerns	Suspected	No concerns	Major concerns	No concerns	No concerns	Very low
LAM+VAL:LIT+VAL	0	Some concerns	Suspected	No concerns	Major concerns	No concerns	No concerns	Very low
LAM+VAL:OLA	0	Some concerns	Suspected	No concerns	Major concerns	No concerns	No concerns	Very low
LAM+VAL:PAL	0	Some concerns	Suspected	No concerns	No concerns	No concerns	No concerns	Low
LAM+VAL:PLA	0	Some concerns	Suspected	No concerns	No concerns	No concerns	No concerns	Low
LAM+VAL:QUE	0	Some concerns	Suspected	No concerns	Major concerns	No concerns	No concerns	Very low
LAM+VAL:RISLAI	0	Some concerns	Suspected	No concerns	No concerns	No concerns	No concerns	Low
LAM+VAL:VAL	0	Some concerns	Suspected	No concerns	Major concerns	No concerns	No concerns	Very low
LIT:PAL	0	No concerns	Suspected	No concerns	Major concerns	No concerns	No concerns	Low
LIT:RISLAI	0	No concerns	Suspected	No concerns	No concerns	Major concerns	No concerns	Low
LIT+OXC:LIT+VAL	0	Major concerns	Suspected	No concerns	Major concerns	No concerns	No concerns	Very low
LIT+OXC:OLA	0	No concerns	Suspected	No concerns	Major concerns	No concerns	No concerns	Low
LIT+OXC:PAL	0	No concerns	Suspected	No concerns	No concerns	No concerns	No concerns	Moderate
LIT+OXC:PLA	0	No concerns	Suspected	No concerns	Major concerns	No concerns	No concerns	Low
LIT+OXC:QUE	0	No concerns	Suspected	No concerns	Major concerns	No concerns	No concerns	Low
LIT+OXC:RISLAI	0	No concerns	Suspected	No concerns	No concerns	No concerns	No concerns	Moderate
LIT+OXC:VAL	0	No concerns	Suspected	No concerns	Major concerns	No concerns	No concerns	Low
LIT+VAL:OLA	0	No concerns	Suspected	No concerns	Major concerns	No concerns	No concerns	Low
LIT+VAL:PAL	0	No concerns	Suspected	No concerns	Major concerns	No concerns	No concerns	Low
LIT+VAL:PLA	0	Major concerns	Suspected	No concerns	Major concerns	No concerns	No concerns	Very low
LIT+VAL:QUE	0	Major concerns	Suspected	No concerns	Major concerns	No concerns	No concerns	Very low
LIT+VAL:RISLAI	0	No concerns	Suspected	No concerns	Major concerns	No concerns	No concerns	Low
OLA:PAL	0	No concerns	Suspected	No concerns	Major concerns	No concerns	No concerns	Low
OLA:QUE	0	No concerns	Suspected	No concerns	No concerns	Major concerns	No concerns	Low
OLA:VAL	0	No concerns	Suspected	No concerns	Major concerns	No concerns	No concerns	Low
PAL:QUE	0	No concerns	Suspected	No concerns	No concerns	No concerns	No concerns	Moderate

PAL:RISLAI	0	No concerns	Suspected	No concerns	Major concerns	No concerns	No concerns	Low
PAL:VAL	0	No concerns	Suspected	No concerns	Major concerns	No concerns	No concerns	Low
QUE:RISLAI	0	No concerns	Suspected	No concerns	No concerns	No concerns	No concerns	Moderate
QUE:VAL	0	Some concerns	Suspected	No concerns	No concerns	No concerns	No concerns	Low
RISLAI:VAL	0	No concerns	Suspected	No concerns	Major concerns	No concerns	No concerns	Low

Meta-regression analysis

	Primary analysis	Publication year	Duration of study	Number of total patients	Percent female	Mean age
Heterogeneity SD	0.105	0.163	0.166	0.172	0.164	0.135
Relative change in the variance		+55%	+58%	+64%	+56%	+29%
β , median (95% CI)		-0.037 (-0.391, 0.340)	-0.198 (-0.742, 0.281)	-0.090 (-0.410, 0.249)	0.065 (-0.406, 0.497)	0.189 (-0.179, 0.518)
Interventions vs placebo	RR (95%CI)	RR (95%CI)	RR (95%CI)	RR (95%CI)	RR (95%CI)	RR (95%CI)
AOM	1.061 (0.573, 1.962)	1.028 (0.496, 2.135)	0.974 (0.468, 2.032)	1.078 (0.463, 2.467)	0.998 (0.502, 2.077)	1.030 (0.517, 2.046)
ARI	0.900 (0.417, 1.942)	0.848 (0.337, 1.956)	0.921 (0.369, 2.200)	0.870 (0.319, 2.330)	0.791 (0.321, 2.030)	0.884 (0.367, 2.029)
ARI+LAM	0.574 (0.315, 1.046)	0.565 (0.262, 1.119)	0.556 (0.257, 1.100)	0.448 (0.180, 1.034)	0.575 (0.268, 1.167)	0.557 (0.270, 1.071)
ARI+VAL	**0.273 (0.076, 0.986)**	**0.308 (0.082, 0.976)**	**0.296 (0.067, 0.961)**	**0.182 (0.031, 0.790)**	0.299 (0.074, 1.008)	0.322 (0.083, 1.026)
ASE	0.385 (0.138, 1.069)	**0.354 (0.103, 0.993)**	**0.297 (0.086, 0.897)**	0.331 (0.094, 1.032)	0.352 (0.115, 1.004)	**0.335 (0.107, 0.910)**
CAR	2.729 (0.635, 11.735)	3.016 (0.001, 24.587)	3.206 (0.802, 24.878)	3.600 (0.704, 33.562)	3.178 (0.835, 19.757)	3.108 (0.800, 30.615)
LAM	**0.713 (0.547, 0.930)**	**0.680 (0.472, 0.922)**	**0.670 (0.455, 0.924)**	**0.575 (0.359, 0.885)**	**0.689 (0.473, 0.940)**	**0.664 (0.470, 0.900)**
LAM+VAL	**0.470 (0.278, 0.793)**	**0.446 (0.223, 0.811)**	**0.433 (0.207, 0.805)**	**0.221 (0.072, 0.630)**	**0.445 (0.222, 0.848)**	**0.428 (0.222, 0.783)**
LIT	**0.791 (0.660, 0.948)**	**0.761 (0.597, 0.954)**	**0.771 (0.605, 0.951)**	**0.697 (0.499, 0.978)**	**0.775 (0.598, 0.972)**	**0.750 (0.597, 0.928)**
LIT+OXC	0.294 (0.086, 1.002)	**0.253 (0.054, 0.822)**	**0.244 (0.053, 0.810)**	**0.184 (0.032, 0.797)**	**0.245 (0.055, 0.813)**	**0.234 (0.044, 0.776)**
LIT+VAL	0.741 (0.481, 1.142)	0.690 (0.389, 1.158)	0.702 (0.383, 1.188)	0.629 (0.291, 1.295)	0.704 (0.382, 1.190)	0.687 (0.398, 1.103)
OLA	**0.742 (0.562, 0.979)**	**0.712 (0.495, 0.995)**	**0.695 (0.482, 0.977)**	0.657 (0.412, 1.016)	0.708 (0.489, 1.003)	0.739 (0.516, 1.021)
PAL	1.311 (0.796, 2.157)	1.302 (0.693, 2.361)	1.727 (0.664, 4.859)	1.436 (0.693, 2.984)	1.291 (0.689, 2.387)	1.323 (0.735, 2.380)
QUE	**0.480 (0.364, 0.633)**	**0.476 (0.324, 0.684)**	**0.476 (0.330, 0.685)**	**0.437 (0.252, 0.730)**	**0.472 (0.327, 0.676)**	**0.487 (0.343, 0.676)**
RISLAI	1.287 (0.857, 1.932)	1.246 (0.768, 2.001)	1.288 (0.798, 2.113)	1.369 (0.793, 2.372)	1.265 (0.778, 2.090)	1.348 (0.827, 2.113)
VAL	0.848 (0.596, 1.206)	0.761 (0.467, 1.129)	0.768 (0.478, 1.146)	0.737 (0.400, 1.251)	0.779 (0.471, 1.168)	0.770 (0.487, 1.125)

Sensitivity analysis

Model fit statistics	Primary analysis	Focusing on studies including only BDI	Focusing on studies including only non rapid-cycling BD patients	Focusing on double-blind studies	Focusing on non-enriched studies	Focusing on non-industry sponsorship studies
Heterogeneity SD	0.105	0.157	0.118	0.123	0.157	0.131
Relative change in the variance		+50%	+12%	+17%	+50%	+25%
Interventions vs placebo	RR (95%CI)	RR (95%CI)	RR (95%CI)	RR (95%CI)	RR (95%CI)	RR (95%CI)
AOM	1.061 (0.573, 1.962)	1.020 (0.505, 2.076)	1.013 (0.508, 2.044)	1.015 (0.515, 2.003)	0.979 (0.392, 2.469)	0.977 (0.390, 2.400)
ARI	0.900 (0.417, 1.942)	0.838 (0.353, 1.923)	0.774 (0.233, 2.198)	0.835 (0.380, 1.908)	0.794 (0.242, 2.374)	0.802 (0.238, 2.305)
ARI+LAM	0.574 (0.315, 1.046)	0.564 (0.270, 1.132)	0.588 (0.237, 1.353)	0.572 (0.290, 1.063)	0.579 (0.233, 1.449)	0.596 (0.240, 1.385)
ARI+VAL	**0.273 (0.076, 0.986)**	**0.303 (0.077, 0.992)**	0.369 (0.047, 1.662)	**0.280 (0.067, 0.839)**	0.356 (0.059, 1.689)	0.401 (0.069, 1.713)
ASE	0.385 (0.138, 1.069)	**0.346 (0.113, 0.957)**	**0.345 (0.106, 0.921)**	**0.350 (0.114, 0.922)**	0.305 (0.049, 1.200)	0.318 (0.050, 1.202)
CAR	2.729 (0.635, 11.735)	4.128 (0.605, 86.834)	4.041 (0.656, 84.894)	3.080 (0.856, 19.051)	3.321 (0.788, 24.464)	3.746 (0.691, 49.730)
LAM	**0.713 (0.547, 0.930)**	**0.678 (0.475, 0.922)**	**0.686 (0.455, 0.985)**	**0.688 (0.495, 0.908)**	**0.675 (0.441, 0.993)**	0.693 (0.453, 1.000)
LAM+VAL	**0.470 (0.278, 0.793)**	**0.423 (0.179, 0.913)**	**0.437 (0.189, 0.888)**	**0.444 (0.232, 0.797)**	**0.434 (0.176, 0.920)**	**0.448 (0.223, 0.821)**
LIT	**0.791 (0.660, 0.948)**	**0.749 (0.583, 0.940)**	**0.761 (0.579, 0.976)**	**0.760 (0.618, 0.926)**	**0.763 (0.595, 0.956)**	**0.784 (0.611, 0.986)**
LIT+OXC	0.294 (0.086, 1.002)	0.219 (0.021, 1.094)	**0.207 (0.016, 0.967)**	**0.242 (0.050, 0.770)**	**0.242 (0.052, 0.800)**	**0.237 (0.055, 0.803)**
LIT+VAL	0.741 (0.481, 1.142)	0.690 (0.383, 1.139)	0.681 (0.368, 1.237)	0.654 (0.361, 1.134)	0.698 (0.383, 1.153)	0.749 (0.417, 1.222)
OLA	**0.742 (0.562, 0.979)**	**0.705 (0.492, 0.985)**	**0.683 (0.468, 0.990)**	**0.717 (0.517, 0.971)**	0.715 (0.471, 1.046)	0.711 (0.465, 1.050)
PAL	1.311 (0.796, 2.157)	1.288 (0.702, 2.367)	1.282 (0.721, 2.319)	1.281 (0.725, 2.266)	1.248 (0.606, 2.695)	1.231 (0.596, 2.579)
QUE	**0.480 (0.364, 0.633)**	**0.463 (0.311, 0.679)**	**0.468 (0.308, 0.688)**	**0.471 (0.338, 0.657)**	**0.473 (0.298, 0.712)**	**0.471 (0.305, 0.698)**
RISLAI	1.287 (0.857, 1.932)	1.240 (0.774, 1.956)	1.222 (0.794, 1.908)	1.253 (0.794, 1.940)	1.203 (0.658, 2.212)	1.184 (0.656, 2.149)
VAL	0.848 (0.596, 1.206)	0.765 (0.458, 1.121)	0.781 (0.462, 1.260)	0.703 (0.444, 1.055)	0.767 (0.466, 1.133)	0.855 (0.507, 1.278)

1.3. Recurrence/relapse rate of manic/hypomanic/mixed episodes (25 RCTs, 6438 patients)

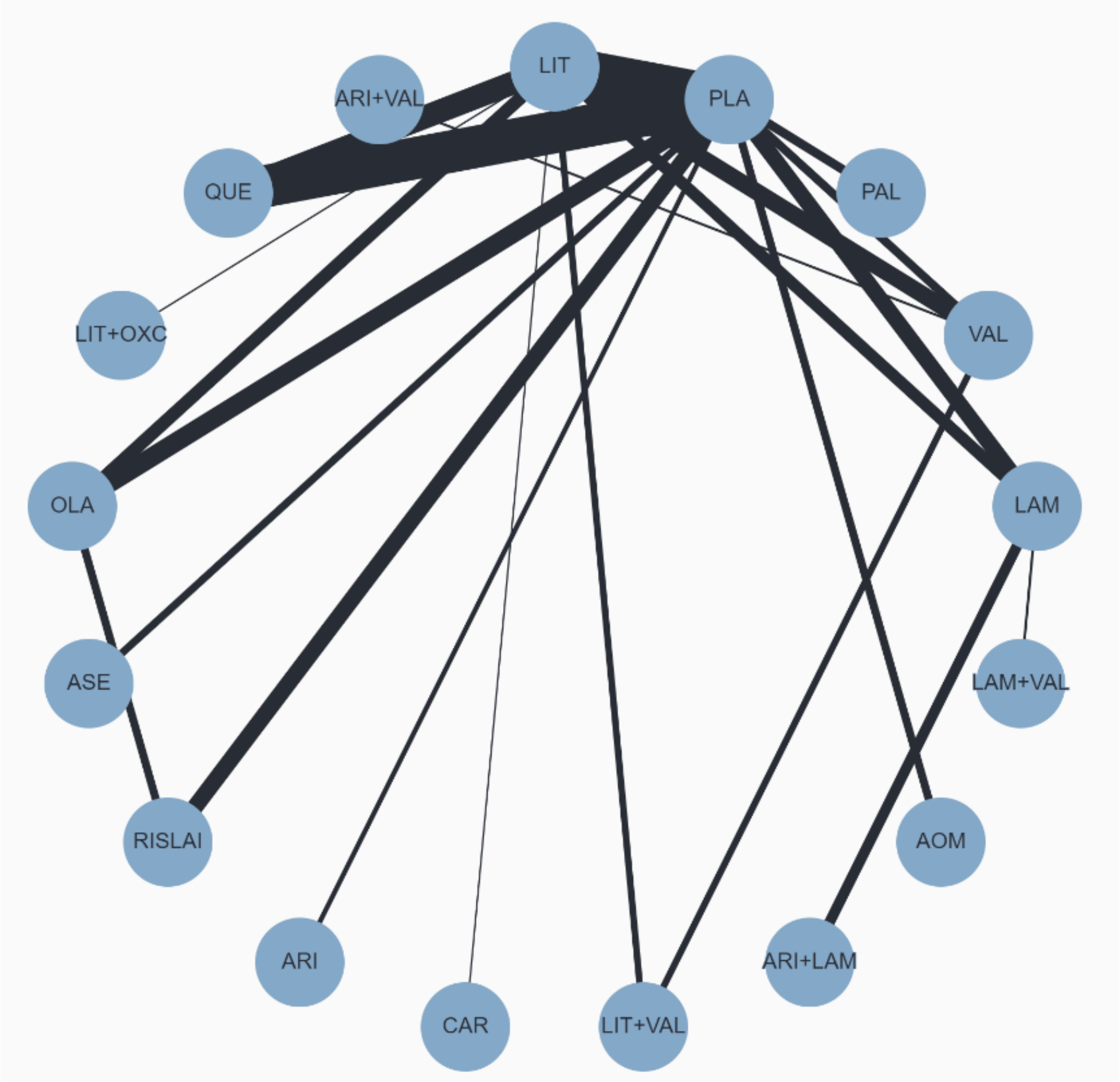

League table

Results from pairwise meta-analysis are presented in the left lower half and results from network meta-analysis in the upper right half.

The boldface result indicates statistical significance.

AOM	0.726 (0.288, 1.832)	0.590 (0.242, 1.439)	0.586 (0.121, 2.848)	1.451 (0.480, 4.389)	0.146 (0.017, 1.280)	**0.340 (0.173, 0.668)**	0.557 (0.165, 1.875)	0.560 (0.299, 1.051)	1.004 (0.288, 3.496)	0.761 (0.368, 1.573)	0.872 (0.453, 1.679)	0.511 (0.251, 1.040)	0.545 (0.286, 1.039)	0.821 (0.417, 1.617)	**0.472 (0.243, 0.920)**	**0.302 (0.166, 0.550)**
	ARI	0.812 (0.309, 2.137)	0.807 (0.159, 4.098)	1.999 (0.621, 6.431)	0.201 (0.022, 1.821)	0.468 (0.216, 1.013)	0.767 (0.215, 2.732)	0.772 (0.371, 1.604)	1.383 (0.376, 5.088)	1.049 (0.463, 2.374)	1.201 (0.565, 2.555)	0.704 (0.315, 1.572)	0.751 (0.356, 1.583)	1.131 (0.521, 2.453)	0.651 (0.303, 1.398)	**0.416 (0.206, 0.843)**
		ARI+LAM	0.994 (0.200, 4.931)	2.461 (0.785, 7.713)	0.248 (0.028, 2.203)	0.576 (0.322, 1.031)	0.945 (0.295, 3.025)	0.950 (0.483, 1.870)	1.703 (0.477, 6.080)	1.291 (0.598, 2.787)	1.479 (0.728, 3.005)	0.866 (0.403, 1.862)	0.924 (0.458, 1.866)	1.392 (0.668, 2.900)	0.801 (0.392, 1.637)	**0.513 (0.265, 0.993)**
			ARI+VAL	2.476 (0.437, 14.030)	0.249 (0.020, 3.150)	0.579 (0.130, 2.577)	0.950 (0.157, 5.754)	0.956 (0.223, 4.096)	1.714 (0.280, 10.478)	1.299 (0.296, 5.696)	1.488 (0.339, 6.533)	0.872 (0.192, 3.957)	0.930 (0.212, 4.083)	1.401 (0.314, 6.258)	0.806 (0.192, 3.381)	0.516 (0.119, 2.228)
				ASE	**0.101 (0.010, 0.989)**	**0.234 (0.088, 0.626)**	0.384 (0.094, 1.569)	**0.386 (0.149, 0.999)**	0.692 (0.164, 2.913)	0.525 (0.190, 1.452)	0.601 (0.228, 1.583)	**0.352 (0.129, 0.964)**	**0.375 (0.143, 0.983)**	0.566 (0.211, 1.514)	**0.326 (0.123, 0.864)**	**0.208 (0.082, 0.529)**
					CAR	2.324 (0.283, 19.091)	3.811 (0.369, 39.358)	3.833 (0.480, 30.597)	6.874 (0.662, 71.350)	5.210 (0.631, 42.996)	5.967 (0.733, 48.550)	3.496 (0.419, 29.163)	3.729 (0.458, 30.365)	5.618 (0.681, 46.321)	3.234 (0.399, 26.198)	2.069 (0.257, 16.663)
		0.576 (0.322, 1.031)				LAM	1.640 (0.599, 4.494)	**1.649 (1.166, 2.333)**	2.957 (0.954, 9.170)	**2.242 (1.355, 3.709)**	**2.567 (1.712, 3.850)**	1.504 (0.915, 2.472)	**1.604 (1.082, 2.379)**	**2.417 (1.545, 3.780)**	1.391 (0.920, 2.105)	0.890 (0.650, 1.219)
						1.640 (0.599, 4.494)	LAM+VAL	1.006 (0.346, 2.920)	1.803 (0.396, 8.208)	1.367 (0.443, 4.218)	1.565 (0.528, 4.639)	0.917 (0.298, 2.822)	0.978 (0.332, 2.887)	1.474 (0.489, 4.440)	0.848 (0.285, 2.523)	0.543 (0.189, 1.560)

					3.833 (0.480, 30.597)	**1.891 (1.147, 3.117)**		LIT	1.793 (0.611, 5.265)	1.359 (0.935, 1.975)	**1.557 (1.173, 2.065)**	0.912 (0.593, 1.403)	0.973 (0.729, 1.299)	**1.465 (1.013, 2.120)**	0.843 (0.657, 1.083)	**0.540 (0.445, 0.655)**
								1.793 (0.611, 5.265)	LIT+OXC	0.758 (0.242, 2.370)	0.868 (0.285, 2.644)	0.509 (0.159, 1.622)	0.543 (0.178, 1.655)	0.817 (0.262, 2.552)	0.470 (0.156, 1.421)	**0.301 (0.101, 0.899)**
								1.333 (0.900, 1.975)		LIT+VAL	1.145 (0.721, 1.820)	0.671 (0.382, 1.178)	0.716 (0.449, 1.140)	1.078 (0.642, 1.810)	**0.621 (0.433, 0.889)**	**0.397 (0.263, 0.599)**
								**1.690 (1.120, 2.550)**			OLA	**0.586 (0.367, 0.936)**	**0.625 (0.439, 0.891)**	0.941 (0.631, 1.404)	**0.542 (0.376, 0.781)**	**0.347 (0.265, 0.453)**
												PAL	1.067 (0.677, 1.681)	1.607 (0.975, 2.649)	0.925 (0.570, 1.500)	**0.592 (0.403, 0.870)**
								0.928 (0.644, 1.337)					QUE	**1.506 (1.010, 2.247)**	0.867 (0.601, 1.251)	**0.555 (0.435, 0.707)**
											0.896 (0.478, 1.679)			RISLAI	**0.576 (0.374, 0.885)**	**0.368 (0.268, 0.507)**
			0.806 (0.192, 3.381)					0.907 (0.697, 1.180)		**0.612 (0.423, 0.886)**					VAL	**0.640 (0.477, 0.857)**
**0.302 (0.166, 0.550)**	**0.416 (0.206, 0.843)**			**0.208 (0.082, 0.529)**		0.864 (0.621, 1.202)		**0.523 (0.422, 0.648)**			**0.369 (0.265, 0.514)**		**0.535 (0.418, 0.684)**	**0.369 (0.268, 0.509)**	0.790 (0.485, 1.286)	PLA

Evaluation of heterogeneity and inconsistency

Between study variance (τ^2)	Heterogeneity assessment	Random-effects design-by-treatment interaction model		
		Q	df	p
0.01	Low	13.398	10	0.202

Inconsistency

	NMA, RR (95%CI)	Direct, RR (95%CI)	Indirect, RR (95%CI)	Inconsistency measures	
				Ratio of risk ratios	P value
LAM vs LIT	1.649 (1.166, 2.333)	1.891 (1.147, 3.117)	1.453 (0.898, 2.351)	1.302 (0.650, 2.605)	0.457
LAM vs PLA	0.890 (0.650, 1.219)	0.864 (0.621, 1.202)	1.189 (0.425, 3.327)	0.727 (0.247, 2.142)	0.563
LIT vs LIT+VAL	1.359 (0.935, 1.975)	1.333 (0.900, 1.975)	1.637 (0.482, 5.562)	0.815 (0.225, 2.944)	0.754
LIT vs OLA	1.557 (1.173, 2.065)	1.690 (1.120, 2.550)	1.446 (0.980, 2.134)	1.168 (0.663, 2.058)	0.590
LIT vs QUE	0.973 (0.729, 1.299)	0.928 (0.644, 1.337)	1.052 (0.656, 1.688)	0.882 (0.485, 1.603)	0.681
LIT vs VAL	0.843 (0.657, 1.083)	0.907 (0.697, 1.180)	0.436 (0.197, 0.965)	2.079 (0.900, 4.802)	0.086
LIT vs PLA	0.540 (0.445, 0.655)	0.523 (0.422, 0.648)	0.620 (0.396, 0.972)	0.843 (0.512, 1.388)	0.502
LIT+VAL vs VAL	0.621 (0.433, 0.889	0.612 (0.423, 0.886)	0.790 (0.167, 3.747)	0.775 (0.156, 3.836)	0.754
OLA vs RISLAI	0.941 (0.631, 1.404)	0.896 (0.478, 1.679)	0.974 (0.580, 1.635)	0.920 (0.407, 2.077)	0.841
OLA vs PLA	0.347 (0.265, 0.453)	0.369 (0.265, 0.514)	0.308 (0.195, 0.486)	1.199 (0.683, 2.105)	0.528
QUE vs PLA	0.555 (0.435, 0.707)	0.535 (0.418, 0.684)	2.085 (0.479, 9.078)	0.256 (0.058, 1.140)	0.074
RISLAI vs PLA	0.368 (0.268, 0.507)	0.369 (0.268, 0.509)	0.308 (0.025, 3.819)	1.200 (0.095, 15.194)	0.888
VAL vs PLA	0.640 (0.477, 0.857)	0.790 (0.485, 1.286)	0.568 (0.394, 0.819)	1.390 (0.755, 2.558)	0.290

Rank probabilities

	Rank 1	Rank 2	Rank 3	Rank 4	Rank 5	Rank 6	Rank 7	Rank 8	Rank 9	Rank 10	Rank 11	Rank 12	Rank 13	Rank 14	Rank 15	Rank 16	Rank 17
AOM	0.102	0.194	0.194	0.144	0.103	0.080	0.066	0.044	0.026	0.017	0.011	0.008	0.006	0.004	0.001	0.000	0.000
ARI	0.034	0.083	0.098	0.094	0.096	0.093	0.096	0.092	0.079	0.053	0.046	0.046	0.045	0.025	0.013	0.007	0.001
ARI+LAM	0.006	0.017	0.034	0.042	0.053	0.064	0.092	0.093	0.096	0.079	0.081	0.093	0.108	0.077	0.035	0.026	0.005
ARI+VAL	0.039	0.050	0.045	0.038	0.036	0.041	0.043	0.043	0.038	0.039	0.042	0.050	0.065	0.075	0.078	0.194	0.084
ASE	0.488	0.225	0.107	0.052	0.036	0.030	0.021	0.016	0.007	0.005	0.003	0.003	0.004	0.002	0.000	0.001	0.000
CAR	0.001	0.003	0.002	0.002	0.002	0.003	0.004	0.005	0.008	0.007	0.011	0.013	0.019	0.030	0.034	0.064	0.795
LAM	0.000	0.000	0.000	0.000	0.000	0.000	0.001	0.001	0.002	0.006	0.017	0.046	0.116	0.302	0.345	0.143	0.021
LAM+VAL	0.049	0.076	0.070	0.065	0.054	0.061	0.070	0.068	0.059	0.054	0.053	0.064	0.070	0.069	0.044	0.063	0.014
LIT	0.000	0.000	0.000	0.000	0.004	0.014	0.048	0.124	0.216	0.246	0.197	0.107	0.035	0.008	0.001	0.000	0.000
LIT+OXC	0.249	0.196	0.121	0.076	0.065	0.056	0.050	0.046	0.030	0.025	0.020	0.020	0.019	0.014	0.008	0.005	0.002
LIT+VAL	0.011	0.043	0.089	0.116	0.133	0.153	0.154	0.123	0.077	0.044	0.027	0.017	0.008	0.005	0.001	0.000	0.000
OLA	0.012	0.062	0.132	0.205	0.217	0.179	0.109	0.050	0.023	0.007	0.003	0.001	0.000	0.000	0.000	0.000	0.000
PAL	0.001	0.003	0.007	0.013	0.020	0.032	0.053	0.082	0.103	0.118	0.126	0.137	0.137	0.100	0.051	0.018	0.003
QUE	0.000	0.000	0.001	0.003	0.006	0.016	0.038	0.082	0.120	0.153	0.187	0.167	0.129	0.070	0.025	0.004	0.000
RISLAI	0.008	0.048	0.100	0.150	0.175	0.176	0.141	0.099	0.053	0.028	0.011	0.007	0.003	0.001	0.000	0.000	0.000
VAL	0.000	0.000	0.001	0.001	0.002	0.005	0.015	0.034	0.063	0.119	0.166	0.221	0.210	0.122	0.037	0.006	0.000
PLA	0.000	0.000	0.000	0.000	0.000	0.000	0.000	0.000	0.000	0.000	0.001	0.003	0.026	0.096	0.328	0.472	0.075

SUCRA

ASE	0.86
AOM	0.78
LIT+OXC	0.77
OLA	0.75
RISLAI	0.74
LIT+VAL	0.70
ARI	0.61
ARI+VAL	0.48
ARI+LAM	0.46
LAM+VAL	0.46
LIT	0.45
PAL	0.42
QUE	0.37
VAL	0.32
LAM	0.16
PLA	0.10
CAR	0.07

Comparison-adjusted funnel plot of studies of drugs versus placebo

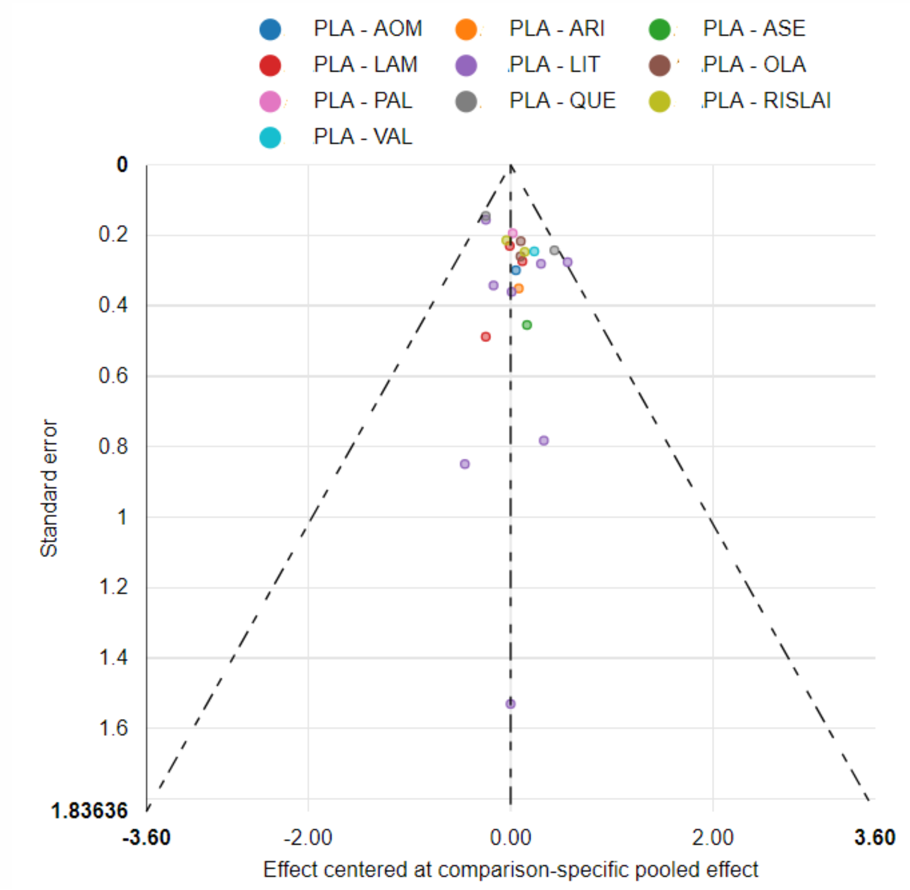

CINeMA rating

Comparison	Number of studies	Within-study bias	Reporting bias	Indirectness	Imprecision	Heterogeneity	Incoherence	Confidence rating
AOM:PLA	1	No concerns	Suspected	No concerns	No concerns	No concerns	No concerns	Moderate
ARI:PLA	1	No concerns	Suspected	No concerns	No concerns	No concerns	No concerns	Moderate
ARI+LAM:LAM	1	No concerns	Suspected	No concerns	Major concerns	No concerns	No concerns	Low
ARI+VAL:VAL	1	Some concerns	Suspected	No concerns	Major concerns	No concerns	No concerns	Very low
ASE:PLA	1	No concerns	Suspected	No concerns	No concerns	No concerns	No concerns	Moderate
CAR:LIT	1	No concerns	Suspected	No concerns	Major concerns	No concerns	No concerns	Low
LAM:LAM+VAL	1	Some concerns	Suspected	No concerns	Major concerns	No concerns	No concerns	Very low
LAM:LIT	2	Some concerns	Suspected	No concerns	No concerns	No concerns	No concerns	Low
LAM:PLA	3	Some concerns	Suspected	No concerns	Major concerns	No concerns	No concerns	Very low
LIT:LIT+OXC	1	No concerns	Suspected	No concerns	Major concerns	No concerns	No concerns	Low
LIT:LIT+VAL	1	Major concerns	Suspected	No concerns	Major concerns	No concerns	No concerns	Very low
LIT:OLA	1	No concerns	Suspected	No concerns	No concerns	No concerns	No concerns	Moderate
LIT:PLA	8	Some concerns	Suspected	No concerns	No concerns	No concerns	No concerns	Low
LIT:QUE	1	Some concerns	Suspected	No concerns	Major concerns	No concerns	No concerns	Very low
LIT:VAL	3	Major concerns	Suspected	No concerns	Major concerns	No concerns	Major concerns	Very low
LIT+VAL:VAL	1	Major concerns	Suspected	No concerns	No concerns	No concerns	No concerns	Low
OLA:PLA	2	No concerns	Suspected	No concerns	No concerns	No concerns	No concerns	Moderate
OLA:RISLAI	1	No concerns	Suspected	No concerns	Major concerns	No concerns	No concerns	Low
PAL:PLA	1	No concerns	Suspected	No concerns	No concerns	No concerns	No concerns	Moderate
PLA:QUE	2	Some concerns	Suspected	No concerns	No concerns	No concerns	Major concerns	Very low
PLA:RISLAI	2	No concerns	Suspected	No concerns	No concerns	No concerns	No concerns	Moderate
PLA:VAL	1	No concerns	Suspected	No concerns	No concerns	No concerns	No concerns	Moderate
AOM:ARI	0	No concerns	Suspected	No concerns	Major concerns	No concerns	No concerns	Low
AOM:ARI+LAM	0	No concerns	Suspected	No concerns	Major concerns	No concerns	No concerns	Low
AOM:ARI+VAL	0	No concerns	Suspected	No concerns	Major concerns	No concerns	No concerns	Low
AOM:ASE	0	No concerns	Suspected	No concerns	Major concerns	No concerns	No concerns	Low
AOM:CAR	0	No concerns	Suspected	No concerns	Major concerns	No concerns	No concerns	Low
AOM:LAM	0	Some concerns	Suspected	No concerns	No concerns	No concerns	No concerns	Low
AOM:LAM+VAL	0	Some concerns	Suspected	No concerns	Major concerns	No concerns	No concerns	Very low
AOM:LIT	0	No concerns	Suspected	No concerns	Major concerns	No concerns	No concerns	Low
AOM:LIT+OXC	0	No concerns	Suspected	No concerns	Major concerns	No concerns	No concerns	Low
AOM:LIT+VAL	0	No concerns	Suspected	No concerns	Major concerns	No concerns	No concerns	Low

AOM:OLA	0	No concerns	Suspected	No concerns	Major concerns	No concerns	No concerns	Low
AOM:PAL	0	No concerns	Suspected	No concerns	Major concerns	No concerns	No concerns	Low
AOM:QUE	0	No concerns	Suspected	No concerns	Major concerns	No concerns	No concerns	Low
AOM:RISLAI	0	No concerns	Suspected	No concerns	Major concerns	No concerns	No concerns	Low
AOM:VAL	0	No concerns	Suspected	No concerns	No concerns	No concerns	No concerns	Moderate
ARI:ARI+LAM	0	No concerns	Suspected	No concerns	Major concerns	No concerns	No concerns	Low
ARI:ARI+VAL	0	No concerns	Suspected	No concerns	Major concerns	No concerns	No concerns	Low
ARI:ASE	0	No concerns	Suspected	No concerns	Major concerns	No concerns	No concerns	Low
ARI:CAR	0	No concerns	Suspected	No concerns	Major concerns	No concerns	No concerns	Low
ARI:LAM	0	Some concerns	Suspected	No concerns	Major concerns	No concerns	No concerns	Very low
ARI:LAM+VAL	0	Some concerns	Suspected	No concerns	Major concerns	No concerns	No concerns	Very low
ARI:LIT	0	No concerns	Suspected	No concerns	Major concerns	No concerns	No concerns	Low
ARI:LIT+OXC	0	No concerns	Suspected	No concerns	Major concerns	No concerns	No concerns	Low
ARI:LIT+VAL	0	No concerns	Suspected	No concerns	Major concerns	No concerns	No concerns	Low
ARI:OLA	0	No concerns	Suspected	No concerns	Major concerns	No concerns	No concerns	Low
ARI:PAL	0	No concerns	Suspected	No concerns	Major concerns	No concerns	No concerns	Low
ARI:QUE	0	No concerns	Suspected	No concerns	Major concerns	No concerns	No concerns	Low
ARI:RISLAI	0	No concerns	Suspected	No concerns	Major concerns	No concerns	No concerns	Low
ARI:VAL	0	No concerns	Suspected	No concerns	Major concerns	No concerns	No concerns	Low
ARI+LAM:ARI+VAL	0	Some concerns	Suspected	No concerns	Major concerns	No concerns	No concerns	Very low
ARI+LAM:ASE	0	No concerns	Suspected	No concerns	Major concerns	No concerns	No concerns	Low
ARI+LAM:CAR	0	No concerns	Suspected	No concerns	Major concerns	No concerns	No concerns	Low
ARI+LAM:LAM+VAL	0	No concerns	Suspected	No concerns	Major concerns	No concerns	No concerns	Low
ARI+LAM:LIT	0	Some concerns	Suspected	No concerns	Major concerns	No concerns	No concerns	Very low
ARI+LAM:LIT+OXC	0	No concerns	Suspected	No concerns	Major concerns	No concerns	No concerns	Low
ARI+LAM:LIT+VAL	0	No concerns	Suspected	No concerns	Major concerns	No concerns	No concerns	Low
ARI+LAM:OLA	0	No concerns	Suspected	No concerns	Major concerns	No concerns	No concerns	Low
ARI+LAM:PAL	0	No concerns	Suspected	No concerns	Major concerns	No concerns	No concerns	Low
ARI+LAM:PLA	0	Some concerns	Suspected	No concerns	No concerns	Major concerns	No concerns	Very low
ARI+LAM:QUE	0	Some concerns	Suspected	No concerns	Major concerns	No concerns	No concerns	Very low
ARI+LAM:RISLAI	0	No concerns	Suspected	No concerns	Major concerns	No concerns	No concerns	Low
ARI+LAM:VAL	0	No concerns	Suspected	No concerns	Major concerns	No concerns	No concerns	Low
ARI+VAL:ASE	0	No concerns	Suspected	No concerns	Major concerns	No concerns	No concerns	Low

ARI+VAL:CAR	0	No concerns	Suspected	No concerns	Major concerns	No concerns	No concerns	Low
ARI+VAL:LAM	0	Some concerns	Suspected	No concerns	Major concerns	No concerns	No concerns	Very low
ARI+VAL:LAM+VAL	0	Some concerns	Suspected	No concerns	Major concerns	No concerns	No concerns	Very low
ARI+VAL:LIT	0	Some concerns	Suspected	No concerns	Major concerns	No concerns	No concerns	Very low
ARI+VAL:LIT+OXC	0	No concerns	Suspected	No concerns	Major concerns	No concerns	No concerns	Low
ARI+VAL:LIT+VAL	0	Major concerns	Suspected	No concerns	Major concerns	No concerns	No concerns	Very low
ARI+VAL:OLA	0	No concerns	Suspected	No concerns	Major concerns	No concerns	No concerns	Low
ARI+VAL:PAL	0	No concerns	Suspected	No concerns	Major concerns	No concerns	No concerns	Low
ARI+VAL:PLA	0	Some concerns	Suspected	No concerns	Major concerns	No concerns	No concerns	Very low
ARI+VAL:QUE	0	Some concerns	Suspected	No concerns	Major concerns	No concerns	No concerns	Very low
ARI+VAL:RISLAI	0	No concerns	Suspected	No concerns	Major concerns	No concerns	No concerns	Low
ASE:CAR	0	No concerns	Suspected	No concerns	No concerns	Major concerns	No concerns	Low
ASE:LAM	0	Some concerns	Suspected	No concerns	No concerns	No concerns	No concerns	Low
ASE:LAM+VAL	0	Some concerns	Suspected	No concerns	Major concerns	No concerns	No concerns	Very low
ASE:LIT	0	No concerns	Suspected	No concerns	No concerns	Major concerns	No concerns	Low
ASE:LIT+OXC	0	No concerns	Suspected	No concerns	Major concerns	No concerns	No concerns	Low
ASE:LIT+VAL	0	No concerns	Suspected	No concerns	Major concerns	No concerns	No concerns	Low
ASE:OLA	0	No concerns	Suspected	No concerns	Major concerns	No concerns	No concerns	Low
ASE:PAL	0	No concerns	Suspected	No concerns	No concerns	Major concerns	No concerns	Low
ASE:QUE	0	No concerns	Suspected	No concerns	No concerns	Major concerns	No concerns	Low
ASE:RISLAI	0	No concerns	Suspected	No concerns	Major concerns	No concerns	No concerns	Low
ASE:VAL	0	No concerns	Suspected	No concerns	No concerns	No concerns	No concerns	Moderate
CAR:LAM	0	Some concerns	Suspected	No concerns	Major concerns	No concerns	No concerns	Very low
CAR:LAM+VAL	0	Some concerns	Suspected	No concerns	Major concerns	No concerns	No concerns	Very low
CAR:LIT+OXC	0	No concerns	Suspected	No concerns	Major concerns	No concerns	No concerns	Low
CAR:LIT+VAL	0	Major concerns	Suspected	No concerns	Major concerns	No concerns	No concerns	Very low
CAR:OLA	0	No concerns	Suspected	No concerns	Major concerns	No concerns	No concerns	Low
CAR:PAL	0	No concerns	Suspected	No concerns	Major concerns	No concerns	No concerns	Low
CAR:PLA	0	No concerns	Suspected	No concerns	Major concerns	No concerns	No concerns	Low
CAR:QUE	0	No concerns	Suspected	No concerns	Major concerns	No concerns	No concerns	Low
CAR:RISLAI	0	No concerns	Suspected	No concerns	Major concerns	No concerns	No concerns	Low
CAR:VAL	0	No concerns	Suspected	No concerns	Major concerns	No concerns	No concerns	Low
LAM:LIT+OXC	0	Some concerns	Suspected	No concerns	Major concerns	No concerns	No concerns	Very low

LAM:LIT+VAL	0	Some concerns	Suspected	No concerns	No concerns	No concerns	No concerns	Low
LAM:OLA	0	No concerns	Suspected	No concerns	No concerns	No concerns	No concerns	Moderate
LAM:PAL	0	Some concerns	Suspected	No concerns	Major concerns	No concerns	No concerns	Very low
LAM:QUE	0	Some concerns	Suspected	No concerns	No concerns	No concerns	No concerns	Low
LAM:RISLAI	0	No concerns	Suspected	No concerns	No concerns	No concerns	No concerns	Moderate
LAM:VAL	0	Some concerns	Suspected	No concerns	Major concerns	No concerns	No concerns	Very low
LAM+VAL:LIT	0	Some concerns	Suspected	No concerns	Major concerns	No concerns	No concerns	Very low
LAM+VAL:LIT+OXC	0	Some concerns	Suspected	No concerns	Major concerns	No concerns	No concerns	Very low
LAM+VAL:LIT+VAL	0	Some concerns	Suspected	No concerns	Major concerns	No concerns	No concerns	Very low
LAM+VAL:OLA	0	Some concerns	Suspected	No concerns	Major concerns	No concerns	No concerns	Very low
LAM+VAL:PAL	0	Some concerns	Suspected	No concerns	Major concerns	No concerns	No concerns	Very low
LAM+VAL:PLA	0	Some concerns	Suspected	No concerns	Major concerns	No concerns	No concerns	Very low
LAM+VAL:QUE	0	Some concerns	Suspected	No concerns	Major concerns	No concerns	No concerns	Very low
LAM+VAL:RISLAI	0	Some concerns	Suspected	No concerns	Major concerns	No concerns	No concerns	Very low
LAM+VAL:VAL	0	Some concerns	Suspected	No concerns	Major concerns	No concerns	No concerns	Very low
LIT:PAL	0	No concerns	Suspected	No concerns	Major concerns	No concerns	No concerns	Low
LIT:RISLAI	0	No concerns	Suspected	No concerns	No concerns	Major concerns	No concerns	Low
LIT+OXC:LIT+VAL	0	Major concerns	Suspected	No concerns	Major concerns	No concerns	No concerns	Very low
LIT+OXC:OLA	0	No concerns	Suspected	No concerns	Major concerns	No concerns	No concerns	Low
LIT+OXC:PAL	0	No concerns	Suspected	No concerns	Major concerns	No concerns	No concerns	Low
LIT+OXC:PLA	0	No concerns	Suspected	No concerns	No concerns	No concerns	No concerns	Moderate
LIT+OXC:QUE	0	No concerns	Suspected	No concerns	Major concerns	No concerns	No concerns	Low
LIT+OXC:RISLAI	0	No concerns	Suspected	No concerns	Major concerns	No concerns	No concerns	Low
LIT+OXC:VAL	0	No concerns	Suspected	No concerns	Major concerns	No concerns	No concerns	Low
LIT+VAL:OLA	0	No concerns	Suspected	No concerns	Major concerns	No concerns	No concerns	Low
LIT+VAL:PAL	0	No concerns	Suspected	No concerns	Major concerns	No concerns	No concerns	Low
LIT+VAL:PLA	0	Major concerns	Suspected	No concerns	No concerns	No concerns	No concerns	Low
LIT+VAL:QUE	0	Some concerns	Suspected	No concerns	Major concerns	No concerns	No concerns	Very low
LIT+VAL:RISLAI	0	No concerns	Suspected	No concerns	Major concerns	No concerns	No concerns	Low
OLA:PAL	0	No concerns	Suspected	No concerns	No concerns	No concerns	No concerns	Moderate
OLA:QUE	0	No concerns	Suspected	No concerns	No concerns	No concerns	No concerns	Moderate
OLA:VAL	0	No concerns	Suspected	No concerns	No concerns	No concerns	No concerns	Moderate
PAL:QUE	0	No concerns	Suspected	No concerns	Major concerns	No concerns	No concerns	Low

PAL:RISLAI	0	No concerns	Suspected	No concerns	Major concerns	No concerns	No concerns	Low
PAL:VAL	0	No concerns	Suspected	No concerns	Major concerns	No concerns	No concerns	Low
QUE:RISLAI	0	No concerns	Suspected	No concerns	No concerns	Major concerns	No concerns	Low
QUE:VAL	0	Some concerns	Suspected	No concerns	Major concerns	No concerns	No concerns	Very low
RISLAI:VAL	0	No concerns	Suspected	No concerns	No concerns	No concerns	No concerns	Moderate

Meta-regression analysis

	Primary analysis	Publication year	**Duration of study**	Number of total patients	Percent female	Mean age
Heterogeneity SD	0.100	0.145	**0.079**	0.092	0.141	0.162
Relative change in the variance		+45%	**-21%**	-8%	+41%	+62%
β , median (95% CI)		-0.113 (-0.529, 0.383)	**-0.497 (-0.985, -0.004)**	-0.192 (-0.422, 0.077)	0.159 (-0.535, 0.724)	-0.087 (-0.669, 0.381)
Interventions vs placebo	RR (95%CI)	RR (95%CI)	RR (95%CI)	RR (95%CI)	RR (95%CI)	RR (95%CI)
AOM	**0.302 (0.166, 0.550)**	**0.310 (0.139, 0.629)**	**0.251 (0.120, 0.480)**	**0.293 (0.144, 0.554)**	**0.285 (0.133, 0.568)**	**0.295 (0.135, 0.580)**
ARI	**0.416 (0.206, 0.843)**	**0.390 (0.158, 0.847)**	0.472 (0.209, 1.011)	**0.382 (0.172, 0.792)**	**0.338 (0.132, 0.933)**	**0.386 (0.155, 0.859)**
ARI+LAM	**0.513 (0.265, 0.993)**	0.515 (0.233, 1.128)	0.517 (0.254, 1.059)	0.532 (0.250, 1.095)	0.529 (0.220, 1.160)	0.519 (0.223, 1.165)
ARI+VAL	0.516 (0.119, 2.228)	0.614 (0.133, 2.859)	0.659 (0.135, 2.604)	0.668 (0.144, 2.992)	0.660 (0.144, 2.911)	0.633 (0.139, 2.866)
ASE	**0.208 (0.082, 0.529)**	**0.196 (0.059, 0.521)**	**0.141 (0.046, 0.346)**	**0.187 (0.066, 0.472)**	**0.187 (0.056, 0.486)**	**0.197 (0.061, 0.495)**
CAR	2.069 (0.257,16.663)	3.169 (0.476, 71.665)	3.723 (0.506, 192.481)	3.670 (0.505, 37.420)	3.001 (0.490, 68.518)	3.310 (0.486, 84.125)
LAM	0.890 (0.650, 1.219)	0.856 (0.583, 1.254)	0.851 (0.600, 1.208)	0.884 (0.625, 1.245)	0.881 (0.592, 1.300)	0.876 (0.583, 1.285)
LAM+VAL	0.543 (0.189, 1.560)	0.469 (0.137, 1.426)	0.475 (0.144, 1.273)	0.475 (0.148, 1.426)	0.471 (0.135, 1.427)	0.480 (0.138, 1.446)
LIT	**0.540 (0.445, 0.655)**	**0.528 (0.408, 0.686)**	**0.554 (0.440, 0.687)**	**0.593 (0.455, 0.755)**	**0.557 (0.410, 0.730)**	**0.529 (0.410, 0.693)**
LIT+OXC	**0.301 (0.101, 0.899)**	**0.254 (0.069, 0.810)**	**0.276 (0.083, 0.774)**	**0.298 (0.082, 0.888)**	**0.273 (0.071, 0.803)**	**0.251 (0.066, 0.789)**
LIT+VAL	**0.397 (0.263, 0.599)**	**0.380 (0.215, 0.669)**	**0.394 (0.243, 0.620)**	**0.429 (0.251, 0.696)**	**0.403 (0.217, 0.697)**	**0.385 (0.210, 0.668)**
OLA	**0.347 (0.265, 0.453)**	**0.341 (0.239, 0.479)**	**0.333 (0.243, 0.455)**	**0.366 (0.259, 0.500)**	**0.340 (0.238, 0.483)**	**0.328 (0.222, 0.480)**
PAL	**0.592 (0.403, 0.870)**	0.605 (0.345, 1.035)	1.220 (0.516, 2.903)	**0.590 (0.355, 0.949)**	0.608 (0.333, 1.042)	0.574 (0.320, 1.025)
QUE	**0.555 (0.435, 0.707)**	**0.592 (0.412, 0.877)**	**0.621 (0.459, 0.848)**	0.717 (0.467, 1.083)	**0.591 (0.416, 0.869)**	**0.565 (0.396, 0.867)**
RISLAI	**0.368 (0.268, 0.507)**	**0.373 (0.237, 0.559)**	**0.423 (0.278, 0.617)**	**0.376 (0.256, 0.550)**	**0.389 (0.224, 0.615)**	**0.349 (0.211, 0.554)**
VAL	**0.640 (0.477, 0.857)**	**0.611 (0.409, 0.910)**	**0.624 (0.447, 0.859)**	**0.684 (0.464, 0.980)**	**0.652 (0.411, 0.961)**	**0.612 (0.409, 0.924)**

Sensitivity analysis

	Primary analysis	Focusing on studies including only BDI	Focusing on studies including only non rapid-cycling BD patients	Focusing on double-blind studies	Focusing on non-enriched studies	Focusing on non-industry sponsorship studies
Heterogeneity SD	0.100	0.125	0.113	0.147	0.121	0.106
Relative change in the variance		+25%	+13%	+47%	+21%	+6%
Interventions vs placebo	RR (95%CI)	RR (95%CI)	RR (95%CI)	RR (95%CI)	RR (95%CI)	RR (95%CI)
AOM	**0.302 (0.166, 0.550)**	**0.289 (0.142, 0.561)**	**0.284 (0.139, 0.562)**	**0.293 (0.140, 0.568)**	**0.276 (0.102, 0.643)**	**0.272 (0.086, 0.635)**
ARI	**0.416 (0.206, 0.843)**	**0.396 (0.176, 0.823)**	0.380 (0.117, 1.002)	**0.387 (0.165, 0.837)**	0.361 (0.117, 1.000)	**0.375 (0.119, 0.983)**
ARI+LAM	**0.513 (0.265, 0.993)**	0.518 (0.234, 1.095)	0.521 (0.198, 1.435)	0.519 (0.229, 1.117)	0.522 (0.175, 1.683)	0.540 (0.196, 1.412)
ARI+VAL	0.516 (0.119, 2.228)	0.646 (0.150, 2.768)	0.833 (0.110, 7.103)	0.613 (0.139, 2.873)	0.888 (0.132, 6.632)	0.863 (0.102, 7.334)
ASE	**0.208 (0.082, 0.529)**	**0.191 (0.058, 0.482)**	**0.185 (0.061, 0.450)**	**0.188 (0.059, 0.480)**	**0.178 (0.028, 0.647)**	**0.166 (0.029, 0.574)**
CAR	2.069 (0.257, 16.663)	5.014 (0.365, 3,491.896)	4.527 (0.468, 199.059)	3.293 (0.460, 109.969)	3.460 (0.505, 168.865)	5.232 (0.298, 327.438)
LAM	0.890 (0.650, 1.219)	0.858 (0.594, 1.239)	0.830 (0.511, 1.312)	0.853 (0.576, 1.259)	0.841 (0.516, 1.324)	0.845 (0.525, 1.317)
LAM+VAL	0.543 (0.189, 1.560)	0.413 (0.068, 1.929)	0.408 (0.068, 1.862)	0.479 (0.144, 1.459)	0.421 (0.069, 1.912)	0.448 (0.136, 1.306)
LIT	**0.540 (0.445, 0.655)**	**0.520 (0.409, 0.670)**	**0.518 (0.385, 0.704)**	**0.531 (0.419, 0.686)**	**0.552 (0.418, 0.711)**	**0.522 (0.396, 0.694)**
LIT+OXC	**0.301 (0.101, 0.899)**	**0.220 (0.027, 0.980)**	**0.208 (0.025, 0.921)**	**0.261 (0.075, 0.792)**	**0.271 (0.067, 0.790)**	**0.241 (0.067, 0.697)**
LIT+VAL	**0.397 (0.263, 0.599)**	**0.375 (0.220, 0.636)**	**0.371 (0.185, 0.711)**	**0.384 (0.192, 0.719)**	**0.401 (0.230, 0.671)**	**0.383 (0.212, 0.654)**
OLA	**0.347 (0.265, 0.453)**	**0.335 (0.242, 0.467)**	**0.335 (0.224, 0.499)**	**0.337 (0.239, 0.476)**	**0.335 (0.226, 0.496)**	**0.333 (0.216, 0.498)**
PAL	**0.592 (0.403, 0.870)**	**0.587 (0.344, 0.963)**	**0.578 (0.348, 0.981)**	0.586 (0.333, 1.003)	0.573 (0.295, 1.084)	0.571 (0.300, 1.037)
QUE	**0.555 (0.435, 0.707)**	**0.530 (0.374, 0.808)**	**0.551 (0.372, 0.858)**	**0.573 (0.406, 0.856)**	**0.561 (0.375, 0.871)**	**0.548 (0.374, 0.839)**
RISLAI	**0.368 (0.268, 0.507)**	**0.361 (0.242, 0.534)**	**0.367 (0.243, 0.535)**	**0.364 (0.238, 0.547)**	**0.360 (0.210, 0.588)**	**0.355 (0.212, 0.575)**
VAL	**0.640 (0.477, 0.857)**	**0.611 (0.419, 0.892)**	**0.600 (0.376, 0.960)**	**0.613 (0.405, 0.916)**	**0.643 (0.427, 0.925)**	**0.617 (0.411, 0.928)**

1.4. All-cause discontinuation (29 RCTs, 6988 patients)

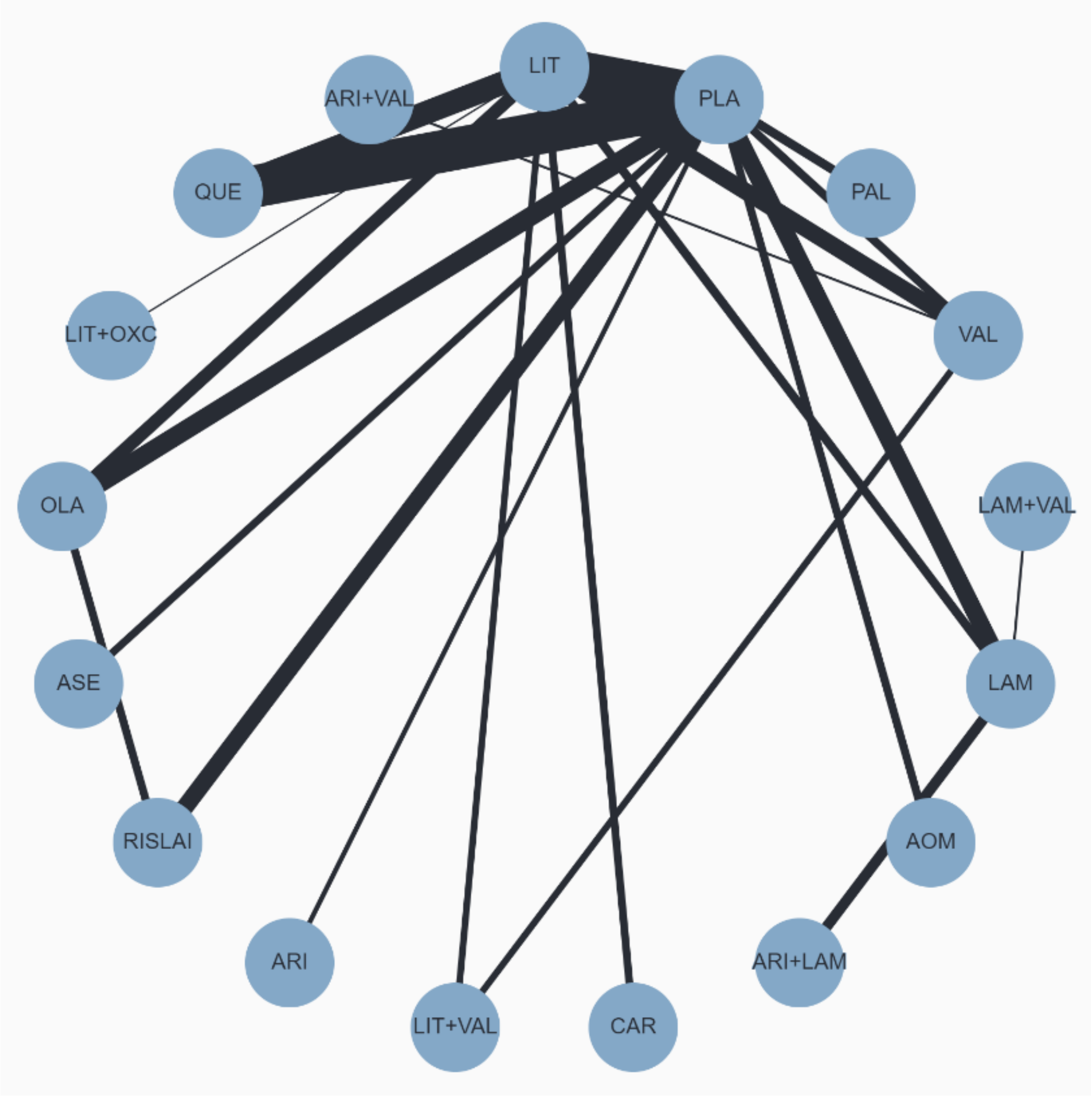

League table

Results from pairwise meta-analysis are presented in the left lower half and results from network meta-analysis in the upper right half.

The boldface result indicates statistical significance.

AOM	0.750 (0.456, 1.232)	0.964 (0.556, 1.672)	0.897 (0.429, 1.873)	1.614 (0.858, 3.038)	0.739 (0.453, 1.207)	0.882 (0.577, 1.350)	0.833 (0.453, 1.532)	0.868 (0.582, 1.293)	0.778 (0.328, 1.846)	0.998 (0.606, 1.644)	1.059 (0.692, 1.620)	0.813 (0.491, 1.344)	1.105 (0.712, 1.714)	0.924 (0.592, 1.443)	0.910 (0.590, 1.406)	0.726 (0.501, 1.054)
	ARI	1.286 (0.763, 2.167)	1.196 (0.584, 2.446)	**2.153 (1.172, 3.953)**	0.986 (0.624, 1.558)	1.177 (0.799, 1.734)	1.110 (0.619, 1.991)	1.157 (0.809, 1.656)	1.037 (0.445, 2.419)	1.331 (0.834, 2.124)	1.412 (0.958, 2.081)	1.084 (0.676, 1.737)	1.474 (0.985, 2.205)	1.232 (0.818, 1.857)	1.214 (0.816, 1.807)	0.969 (0.697, 1.345)
		ARI+LAM	0.930 (0.440, 1.967)	1.674 (0.871, 3.217)	0.767 (0.462, 1.272)	0.915 (0.645, 1.298)	0.864 (0.494, 1.511)	0.900 (0.592, 1.368)	0.807 (0.337, 1.933)	1.035 (0.618, 1.734)	1.098 (0.699, 1.725)	0.843 (0.497, 1.430)	1.146 (0.720, 1.825)	0.958 (0.597, 1.539)	0.944 (0.599, 1.488)	0.753 (0.502, 1.131)
			ARI+VAL	1.800 (0.796, 4.072)	0.825 (0.414, 1.640)	0.984 (0.507, 1.909)	0.929 (0.420, 2.053)	0.968 (0.517, 1.810)	0.868 (0.322, 2.335)	1.113 (0.570, 2.173)	1.181 (0.610, 2.286)	0.906 (0.441, 1.863)	1.233 (0.630, 2.411)	1.030 (0.522, 2.034)	1.015 (0.560, 1.841)	0.810 (0.429, 1.530)
				ASE	**0.458 (0.251, 0.837)**	**0.547 (0.315, 0.949)**	0.516 (0.255, 1.042)	**0.538 (0.316, 0.914)**	0.482 (0.190, 1.225)	0.618 (0.336, 1.138)	0.656 (0.378, 1.139)	**0.503 (0.273, 0.930)**	0.685 (0.390, 1.201)	0.572 (0.325, 1.009)	**0.564 (0.323, 0.986)**	**0.450 (0.270, 0.750)**
					CAR	1.194 (0.828, 1.722)	1.126 (0.637, 1.992)	1.174 (0.883, 1.560)	1.052 (0.464, 2.385)	1.350 (0.888, 2.052)	1.432 (0.998, 2.056)	1.099 (0.690, 1.750)	**1.495 (1.021, 2.188)**	1.250 (0.839, 1.862)	1.231 (0.872, 1.738)	0.983 (0.714, 1.352)
		0.915 (0.645, 1.298)				LAM	0.944 (0.610, 1.460)	0.983 (0.781, 1.238)	0.882 (0.396, 1.963)	1.131 (0.774, 1.653)	1.200 (0.902, 1.597)	0.921 (0.620, 1.369)	1.252 (0.921, 1.703)	1.047 (0.761, 1.441)	1.032 (0.771, 1.380)	0.823 (0.670, 1.011)
						0.944 (0.610, 1.460)	LAM+VAL	1.042 (0.636, 1.707)	0.934 (0.375, 2.326)	1.199 (0.672, 2.138)	1.272 (0.755, 2.143)	0.976 (0.541, 1.760)	1.327 (0.778, 2.263)	1.109 (0.646, 1.906)	1.093 (0.647, 1.847)	0.872 (0.538, 1.414)

					1.174 (0.883, 1.560)	0.995 (0.713, 1.388)		LIT	0.897 (0.416, 1.930)	1.150 (0.846, 1.564)	1.220 (0.977, 1.524)	0.937 (0.648, 1.353)	1.274 (0.989, 1.640)	1.065 (0.805, 1.407)	1.049 (0.864, 1.274)	**0.837 (0.725, 0.966)**
								0.897 (0.416, 1.930)	LIT+OXC	1.283 (0.562, 2.931)	1.361 (0.613, 3.025)	1.045 (0.446, 2.445)	1.420 (0.633, 3.185)	1.188 (0.525, 2.686)	1.170 (0.530, 2.581)	0.934 (0.428, 2.037)
								1.089 (0.779, 1.522)		LIT+VAL	1.061 (0.729, 1.544)	0.814 (0.506, 1.309)	1.107 (0.747, 1.642)	0.926 (0.614, 1.395)	0.912 (0.672, 1.238)	0.728 (0.522, 1.015)
								1.261 (0.891, 1.786)			OLA	0.767 (0.516, 1.141)	1.044 (0.769, 1.417)	0.872 (0.653, 1.166)	0.860 (0.646, 1.144)	**0.686 (0.558, 0.843)**
												PAL	1.360 (0.901, 2.052)	1.137 (0.748, 1.727)	1.120 (0.746, 1.682)	0.894 (0.637, 1.254)
								1.346 (0.945, 1.918)					QUE	0.836 (0.596, 1.172)	0.824 (0.604, 1.123)	**0.657 (0.520, 0.830)**
											0.689 (0.461, 1.029)			RISLAI	0.985 (0.709, 1.369)	0.786 (0.615, 1.005)
			1.015 (0.560, 1.841)					1.073 (0.876, 1.314)		0.865 (0.621, 1.206)					VAL	**0.798 (0.638, 0.999)**
0.726 (0.501, 1.054)	0.969 (0.697, 1.345)			**0.450 (0.270, 0.750)**		0.834 (0.671, 1.035)		**0.833 (0.710, 0.978)**			**0.719 (0.560, 0.924)**	0.894 (0.637, 1.254)	**0.649 (0.507, 0.830)**	**0.748 (0.580, 0.965)**	0.821 (0.576, 1.171)	PLA

Evaluation of heterogeneity and inconsistency

Between study variance (τ^2)	Heterogeneity assessment	Random-effects design-by-treatment interaction model		
		Q	df	p
0.026	Low	4.183	10	0.939

Inconsistency

	NMA, RR (95%CI)	Direct, RR (95%CI)	Indirect, RR (95%CI)	Inconsistency measures	
				Ratio of risk ratios	P value
LAM vs LIT	0.983 (0.781, 1.238)	0.995 (0.713, 1.388)	0.973 (0.708, 1.338)	1.023 (0.645, 1.621)	0.924
LAM vs PLA	0.823 (0.670, 1.011)	0.834 (0.671, 1.035)	0.729 (0.374, 1.424)	1.143 (0.566, 2.309)	0.709
LIT vs LIT+VAL	1.150 (0.846, 1.564)	1.089 (0.779, 1.522)	1.536 (0.712, 3.312)	0.709 (0.307, 1.640)	0.422
LIT vs OLA	1.220 (0.977, 1.524)	1.261 (0.891, 1.786)	1.193 (0.894, 1.593)	1.057 (0.673, 1.662)	0.809
LIT vs QUE	1.274 (0.989, 1.640)	1.346 (0.945, 1.918)	1.202 (0.837, 1.726)	1.120 (0.675, 1.859)	0.660
LIT vs VAL	1.049 (0.864, 1.274)	1.073 (0.876, 1.314)	0.806 (0.404, 1.605)	1.332 (0.650, 2.733)	0.434
LIT vs PLA	0.837 (0.725, 0.966)	0.833 (0.710, 0.978)	0.852 (0.618, 1.177)	0.978 (0.682, 1.401)	0.902
LIT+VAL vs VAL	0.912 (0.672, 1.238)	0.865 (0.621, 1.206)	1.230 (0.558, 2.711)	0.704 (0.299, 1.658)	0.422
OLA vs RISLAI	0.872 (0.653, 1.166)	0.689 (0.461, 1.029)	1.129 (0.742, 1.716)	0.610 (0.342, 1.091)	0.096
OLA vs PLA	0.686 (0.558, 0.843)	0.719 (0.560, 0.924)	0.621 (0.432, 0.892)	1.159 (0.746, 1.800)	0.512
QUE vs PLA	0.657 (0.520, 0.830)	0.649 (0.507, 0.830)	0.741 (0.350, 1.568)	0.875 (0.398, 1.926)	0.741
RISLAI vs PLA	0.786 (0.615, 1.005)	0.748 (0.580, 0.965)	1.515 (0.603, 3.805)	0.494 (0.190, 1.283)	0.147
VAL vs PLA	0.798 (0.638, 0.999)	0.821 (0.576, 1.171)	0.783 (0.586, 1.046)	1.049 (0.664, 1.658)	0.837

Rank probabilities

	Rank 1	Rank 2	Rank 3	Rank 4	Rank 5	Rank 6	Rank 7	Rank 8	Rank 9	Rank 10	Rank 11	Rank 12	Rank 13	Rank 14	Rank 15	Rank 16	Rank 17
AOM	0.036	0.120	0.118	0.102	0.093	0.078	0.074	0.064	0.053	0.046	0.047	0.040	0.041	0.028	0.024	0.022	0.014
ARI	0.001	0.006	0.011	0.015	0.021	0.027	0.034	0.037	0.041	0.047	0.056	0.075	0.095	0.111	0.126	0.151	0.147
ARI+LAM	0.025	0.081	0.087	0.080	0.085	0.084	0.077	0.068	0.062	0.057	0.055	0.051	0.046	0.043	0.038	0.037	0.024
ARI+VAL	0.041	0.090	0.066	0.055	0.052	0.047	0.046	0.043	0.044	0.039	0.041	0.045	0.047	0.052	0.061	0.090	0.141
ASE	0.753	0.119	0.047	0.027	0.014	0.011	0.008	0.006	0.004	0.003	0.003	0.001	0.001	0.001	0.001	0.001	0.001
CAR	0.000	0.001	0.002	0.006	0.011	0.013	0.021	0.023	0.029	0.038	0.046	0.074	0.095	0.114	0.147	0.193	0.186
LAM	0.000	0.001	0.003	0.013	0.029	0.053	0.083	0.110	0.132	0.144	0.147	0.124	0.087	0.048	0.020	0.005	0.001
LAM+VAL	0.013	0.046	0.041	0.049	0.049	0.049	0.051	0.049	0.047	0.048	0.054	0.061	0.074	0.073	0.081	0.096	0.120
LIT	0.000	0.000	0.000	0.002	0.007	0.025	0.052	0.106	0.152	0.193	0.187	0.152	0.082	0.035	0.008	0.001	0.000
LIT+OXC	0.054	0.096	0.054	0.044	0.043	0.041	0.035	0.034	0.033	0.032	0.032	0.038	0.040	0.046	0.056	0.092	0.231
LIT+VAL	0.021	0.090	0.102	0.119	0.102	0.102	0.088	0.082	0.062	0.054	0.044	0.042	0.030	0.024	0.018	0.015	0.005
OLA	0.014	0.106	0.173	0.183	0.165	0.125	0.087	0.058	0.034	0.025	0.014	0.009	0.004	0.001	0.001	0.000	0.000
PAL	0.004	0.016	0.023	0.033	0.035	0.055	0.057	0.053	0.056	0.059	0.068	0.084	0.096	0.098	0.095	0.096	0.073
QUE	0.035	0.197	0.210	0.170	0.133	0.086	0.061	0.036	0.027	0.019	0.010	0.007	0.005	0.003	0.001	0.000	0.000
RISLAI	0.004	0.026	0.046	0.065	0.094	0.111	0.107	0.102	0.092	0.076	0.078	0.066	0.056	0.036	0.023	0.012	0.006
VAL	0.001	0.005	0.018	0.037	0.067	0.093	0.119	0.130	0.132	0.113	0.097	0.074	0.056	0.035	0.016	0.006	0.002
PLA	0.000	0.000	0.000	0.000	0.000	0.000	0.000	0.000	0.002	0.007	0.019	0.056	0.146	0.252	0.286	0.183	0.050

SUCRA

LIT+VAL	0.87
OLA	0.82
ASE	0.67
QUE	0.62
ARI+LAM	0.61
RISLAI	0.54
AOM	0.53
VAL	0.51
ARI+VAL	0.50
LAM	0.47
LIT	0.46
LAM+VAL	0.40
LIT+OXC	0.40
PAL	0.37
ARI	0.37
CAR	0.26
PLA	0.10

Comparison-adjusted funnel plot of studies of drugs versus placebo

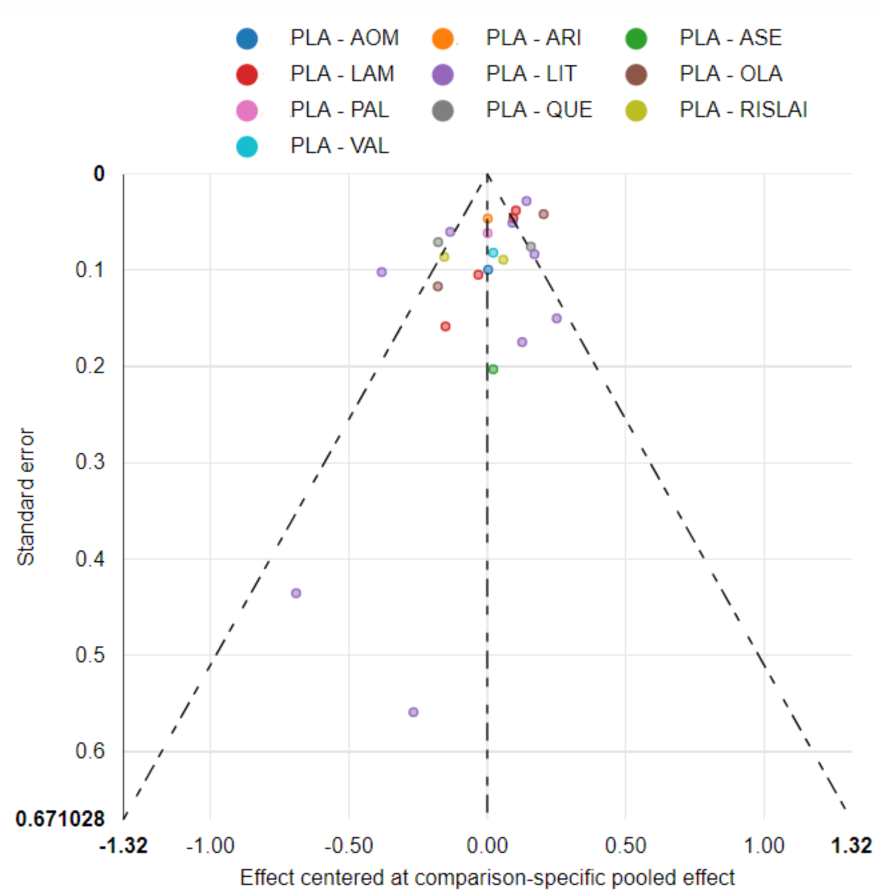

CINeMA rating

Comparison	Number of studies	Within-study bias	Reporting bias	Indirectness	Imprecision	Heterogeneity	Incoherence	Confidence rating
AOM:PLA	1	No concerns	Suspected	No concerns	Major concerns	No concerns	No concerns	Low
ARI:PLA	1	No concerns	Suspected	No concerns	Major concerns	No concerns	No concerns	Low
ARI+LAM:LAM	1	No concerns	Suspected	No concerns	Major concerns	No concerns	No concerns	Low
ARI+VAL:VAL	1	Some concerns	Suspected	No concerns	Major concerns	No concerns	No concerns	Very low
ASE:PLA	1	No concerns	Suspected	No concerns	No concerns	No concerns	No concerns	Moderate
CAR:LIT	3	Major concerns	Suspected	No concerns	Major concerns	No concerns	No concerns	Very low
LAM:LAM+VAL	1	Some concerns	Suspected	No concerns	Major concerns	No concerns	No concerns	Very low
LAM:LIT	1	Some concerns	Suspected	No concerns	Major concerns	No concerns	No concerns	Very low
LAM:PLA	3	Some concerns	Suspected	No concerns	Major concerns	No concerns	No concerns	Very low
LIT:LIT+OXC	1	No concerns	Suspected	No concerns	Major concerns	No concerns	No concerns	Low
LIT:LIT+VAL	1	Major concerns	Suspected	No concerns	Major concerns	No concerns	No concerns	Very low
LIT:OLA	1	No concerns	Suspected	No concerns	Major concerns	No concerns	No concerns	Low
LIT:PLA	6	Some concerns	Suspected	No concerns	No concerns	Major concerns	No concerns	Very low
LIT:QUE	1	Some concerns	Suspected	No concerns	Major concerns	No concerns	No concerns	Very low
LIT:VAL	3	No concerns	Suspected	No concerns	Major concerns	No concerns	No concerns	Low
LIT+VAL:VAL	1	Major concerns	Suspected	No concerns	Major concerns	No concerns	No concerns	Very low
OLA:PLA	2	No concerns	Suspected	No concerns	No concerns	Major concerns	No concerns	Low
OLA:RISLAI	1	No concerns	Suspected	No concerns	Major concerns	No concerns	No concerns	Low
PAL:PLA	1	No concerns	Suspected	No concerns	Major concerns	No concerns	No concerns	Low
PLA:QUE	2	Some concerns	Suspected	No concerns	No concerns	Major concerns	No concerns	Very low
PLA:RISLAI	2	No concerns	Suspected	No concerns	Major concerns	No concerns	No concerns	Low
PLA:VAL	1	No concerns	Suspected	No concerns	No concerns	Major concerns	No concerns	Low
AOM:ARI	0	No concerns	Suspected	No concerns	Major concerns	No concerns	No concerns	Low
AOM:ARI+LAM	0	No concerns	Suspected	No concerns	Major concerns	No concerns	No concerns	Low
AOM:ARI+VAL	0	No concerns	Suspected	No concerns	Major concerns	No concerns	No concerns	Low
AOM:ASE	0	No concerns	Suspected	No concerns	Major concerns	No concerns	No concerns	Low
AOM:CAR	0	No concerns	Suspected	No concerns	Major concerns	No concerns	No concerns	Low
AOM:LAM	0	No concerns	Suspected	No concerns	Major concerns	No concerns	No concerns	Low
AOM:LAM+VAL	0	Some concerns	Suspected	No concerns	Major concerns	No concerns	No concerns	Very low
AOM:LIT	0	No concerns	Suspected	No concerns	Major concerns	No concerns	No concerns	Low
AOM:LIT+OXC	0	No concerns	Suspected	No concerns	Major concerns	No concerns	No concerns	Low
AOM:LIT+VAL	0	No concerns	Suspected	No concerns	Major concerns	No concerns	No concerns	Low

AOM:OLA	0	No concerns	Suspected	No concerns	Major concerns	No concerns	No concerns	Low
AOM:PAL	0	No concerns	Suspected	No concerns	Major concerns	No concerns	No concerns	Low
AOM:QUE	0	No concerns	Suspected	No concerns	Major concerns	No concerns	No concerns	Low
AOM:RISLAI	0	No concerns	Suspected	No concerns	Major concerns	No concerns	No concerns	Low
AOM:VAL	0	No concerns	Suspected	No concerns	Major concerns	No concerns	No concerns	Low
ARI:ARI+LAM	0	No concerns	Suspected	No concerns	Major concerns	No concerns	No concerns	Low
ARI:ARI+VAL	0	No concerns	Suspected	No concerns	Major concerns	No concerns	No concerns	Low
ARI:ASE	0	No concerns	Suspected	No concerns	No concerns	No concerns	No concerns	Moderate
ARI:CAR	0	No concerns	Suspected	No concerns	Major concerns	No concerns	No concerns	Low
ARI:LAM	0	No concerns	Suspected	No concerns	Major concerns	No concerns	No concerns	Low
ARI:LAM+VAL	0	Some concerns	Suspected	No concerns	Major concerns	No concerns	No concerns	Very low
ARI:LIT	0	No concerns	Suspected	No concerns	Major concerns	No concerns	No concerns	Low
ARI:LIT+OXC	0	No concerns	Suspected	No concerns	Major concerns	No concerns	No concerns	Low
ARI:LIT+VAL	0	No concerns	Suspected	No concerns	Major concerns	No concerns	No concerns	Low
ARI:OLA	0	No concerns	Suspected	No concerns	Major concerns	No concerns	No concerns	Low
ARI:PAL	0	No concerns	Suspected	No concerns	Major concerns	No concerns	No concerns	Low
ARI:QUE	0	No concerns	Suspected	No concerns	Major concerns	No concerns	No concerns	Low
ARI:RISLAI	0	No concerns	Suspected	No concerns	Major concerns	No concerns	No concerns	Low
ARI:VAL	0	No concerns	Suspected	No concerns	Major concerns	No concerns	No concerns	Low
ARI+LAM:ARI+VAL	0	Some concerns	Suspected	No concerns	Major concerns	No concerns	No concerns	Very low
ARI+LAM:ASE	0	No concerns	Suspected	No concerns	Major concerns	No concerns	No concerns	Low
ARI+LAM:CAR	0	No concerns	Suspected	No concerns	Major concerns	No concerns	No concerns	Low
ARI+LAM:LAM+VAL	0	No concerns	Suspected	No concerns	Major concerns	No concerns	No concerns	Low
ARI+LAM:LIT	0	No concerns	Suspected	No concerns	Major concerns	No concerns	No concerns	Low
ARI+LAM:LIT+OXC	0	No concerns	Suspected	No concerns	Major concerns	No concerns	No concerns	Low
ARI+LAM:LIT+VAL	0	No concerns	Suspected	No concerns	Major concerns	No concerns	No concerns	Low
ARI+LAM:OLA	0	No concerns	Suspected	No concerns	Major concerns	No concerns	No concerns	Low
ARI+LAM:PAL	0	No concerns	Suspected	No concerns	Major concerns	No concerns	No concerns	Low
ARI+LAM:PLA	0	No concerns	Suspected	No concerns	Major concerns	No concerns	No concerns	Low
ARI+LAM:QUE	0	Some concerns	Suspected	No concerns	Major concerns	No concerns	No concerns	Very low
ARI+LAM:RISLAI	0	No concerns	Suspected	No concerns	Major concerns	No concerns	No concerns	Low
ARI+LAM:VAL	0	No concerns	Suspected	No concerns	Major concerns	No concerns	No concerns	Low
ARI+VAL:ASE	0	No concerns	Suspected	No concerns	Major concerns	No concerns	No concerns	Low

ARI+VAL:CAR	0	Some concerns	Suspected	No concerns	Major concerns	No concerns	No concerns	Very low
ARI+VAL:LAM	0	Some concerns	Suspected	No concerns	Major concerns	No concerns	No concerns	Very low
ARI+VAL:LAM+VAL	0	Some concerns	Suspected	No concerns	Major concerns	No concerns	No concerns	Very low
ARI+VAL:LIT	0	Some concerns	Suspected	No concerns	Major concerns	No concerns	No concerns	Very low
ARI+VAL:LIT+OXC	0	No concerns	Suspected	No concerns	Major concerns	No concerns	No concerns	Low
ARI+VAL:LIT+VAL	0	Major concerns	Suspected	No concerns	Major concerns	No concerns	No concerns	Very low
ARI+VAL:OLA	0	No concerns	Suspected	No concerns	Major concerns	No concerns	No concerns	Low
ARI+VAL:PAL	0	No concerns	Suspected	No concerns	Major concerns	No concerns	No concerns	Low
ARI+VAL:PLA	0	Some concerns	Suspected	No concerns	Major concerns	No concerns	No concerns	Very low
ARI+VAL:QUE	0	Some concerns	Suspected	No concerns	Major concerns	No concerns	No concerns	Very low
ARI+VAL:RISLAI	0	No concerns	Suspected	No concerns	Major concerns	No concerns	No concerns	Low
ASE:CAR	0	No concerns	Suspected	No concerns	No concerns	No concerns	No concerns	Moderate
ASE:LAM	0	No concerns	Suspected	No concerns	No concerns	Major concerns	No concerns	Low
ASE:LAM+VAL	0	Some concerns	Suspected	No concerns	Major concerns	No concerns	No concerns	Very low
ASE:LIT	0	No concerns	Suspected	No concerns	No concerns	Major concerns	No concerns	Low
ASE:LIT+OXC	0	No concerns	Suspected	No concerns	Major concerns	No concerns	No concerns	Low
ASE:LIT+VAL	0	No concerns	Suspected	No concerns	Major concerns	No concerns	No concerns	Low
ASE:OLA	0	No concerns	Suspected	No concerns	Major concerns	No concerns	No concerns	Low
ASE:PAL	0	No concerns	Suspected	No concerns	No concerns	Major concerns	No concerns	Low
ASE:QUE	0	No concerns	Suspected	No concerns	Major concerns	No concerns	No concerns	Low
ASE:RISLAI	0	No concerns	Suspected	No concerns	Major concerns	No concerns	No concerns	Low
ASE:VAL	0	No concerns	Suspected	No concerns	No concerns	Major concerns	No concerns	Low
CAR:LAM	0	Some concerns	Suspected	No concerns	Major concerns	No concerns	No concerns	Very low
CAR:LAM+VAL	0	Some concerns	Suspected	No concerns	Major concerns	No concerns	No concerns	Very low
CAR:LIT+OXC	0	No concerns	Suspected	No concerns	Major concerns	No concerns	No concerns	Low
CAR:LIT+VAL	0	Major concerns	Suspected	No concerns	Major concerns	No concerns	No concerns	Very low
CAR:OLA	0	No concerns	Suspected	No concerns	Major concerns	No concerns	No concerns	Low
CAR:PAL	0	No concerns	Suspected	No concerns	Major concerns	No concerns	No concerns	Low
CAR:PLA	0	No concerns	Suspected	No concerns	Major concerns	No concerns	No concerns	Low
CAR:QUE	0	Some concerns	Suspected	No concerns	No concerns	Major concerns	No concerns	Very low
CAR:RISLAI	0	No concerns	Suspected	No concerns	Major concerns	No concerns	No concerns	Low
CAR:VAL	0	Major concerns	Suspected	No concerns	Major concerns	No concerns	No concerns	Very low
LAM:LIT+OXC	0	No concerns	Suspected	No concerns	Major concerns	No concerns	No concerns	Low

LAM:LIT+VAL	0	Some concerns	Suspected	No concerns	Major concerns	No concerns	No concerns	Very low
LAM:OLA	0	No concerns	Suspected	No concerns	Major concerns	No concerns	No concerns	Low
LAM:PAL	0	No concerns	Suspected	No concerns	Major concerns	No concerns	No concerns	Low
LAM:QUE	0	Some concerns	Suspected	No concerns	Major concerns	No concerns	No concerns	Very low
LAM:RISLAI	0	No concerns	Suspected	No concerns	Major concerns	No concerns	No concerns	Low
LAM:VAL	0	Some concerns	Suspected	No concerns	Major concerns	No concerns	No concerns	Very low
LAM+VAL:LIT	0	Some concerns	Suspected	No concerns	Major concerns	No concerns	No concerns	Very low
LAM+VAL:LIT+OXC	0	Some concerns	Suspected	No concerns	Major concerns	No concerns	No concerns	Very low
LAM+VAL:LIT+VAL	0	Some concerns	Suspected	No concerns	Major concerns	No concerns	No concerns	Very low
LAM+VAL:OLA	0	Some concerns	Suspected	No concerns	Major concerns	No concerns	No concerns	Very low
LAM+VAL:PAL	0	Some concerns	Suspected	No concerns	Major concerns	No concerns	No concerns	Very low
LAM+VAL:PLA	0	Some concerns	Suspected	No concerns	Major concerns	No concerns	No concerns	Very low
LAM+VAL:QUE	0	Some concerns	Suspected	No concerns	Major concerns	No concerns	No concerns	Very low
LAM+VAL:RISLAI	0	Some concerns	Suspected	No concerns	Major concerns	No concerns	No concerns	Very low
LAM+VAL:VAL	0	Some concerns	Suspected	No concerns	Major concerns	No concerns	No concerns	Very low
LIT:PAL	0	No concerns	Suspected	No concerns	Major concerns	No concerns	No concerns	Low
LIT:RISLAI	0	No concerns	Suspected	No concerns	Major concerns	No concerns	No concerns	Low
LIT+OXC:LIT+VAL	0	No concerns	Suspected	No concerns	Major concerns	No concerns	No concerns	Low
LIT+OXC:OLA	0	No concerns	Suspected	No concerns	Major concerns	No concerns	No concerns	Low
LIT+OXC:PAL	0	No concerns	Suspected	No concerns	Major concerns	No concerns	No concerns	Low
LIT+OXC:PLA	0	No concerns	Suspected	No concerns	Major concerns	No concerns	No concerns	Low
LIT+OXC:QUE	0	No concerns	Suspected	No concerns	Major concerns	No concerns	No concerns	Low
LIT+OXC:RISLAI	0	No concerns	Suspected	No concerns	Major concerns	No concerns	No concerns	Low
LIT+OXC:VAL	0	No concerns	Suspected	No concerns	Major concerns	No concerns	No concerns	Low
LIT+VAL:OLA	0	No concerns	Suspected	No concerns	Major concerns	No concerns	No concerns	Low
LIT+VAL:PAL	0	No concerns	Suspected	No concerns	Major concerns	No concerns	No concerns	Low
LIT+VAL:PLA	0	Major concerns	Suspected	No concerns	Major concerns	No concerns	No concerns	Very low
LIT+VAL:QUE	0	Major concerns	Suspected	No concerns	Major concerns	No concerns	No concerns	Very low
LIT+VAL:RISLAI	0	No concerns	Suspected	No concerns	Major concerns	No concerns	No concerns	Low
OLA:PAL	0	No concerns	Suspected	No concerns	Major concerns	No concerns	No concerns	Low
OLA:QUE	0	No concerns	Suspected	No concerns	Major concerns	No concerns	No concerns	Low
OLA:VAL	0	No concerns	Suspected	No concerns	Major concerns	No concerns	No concerns	Low
PAL:QUE	0	No concerns	Suspected	No concerns	Major concerns	No concerns	No concerns	Low

PAL:RISLAI	0	No concerns	Suspected	No concerns	Major concerns	No concerns	No concerns	Low
PAL:VAL	0	No concerns	Suspected	No concerns	Major concerns	No concerns	No concerns	Low
QUE:RISLAI	0	No concerns	Suspected	No concerns	Major concerns	No concerns	No concerns	Low
QUE:VAL	0	No concerns	Suspected	No concerns	Major concerns	No concerns	No concerns	Low
RISLAI:VAL	0	No concerns	Suspected	No concerns	Major concerns	No concerns	No concerns	Low

Meta-regression analysis

	Primary analysis	Publication year	Duration of study	Number of total patients	Percent female	Mean age
Heterogeneity SD	0.161	0.156	0.151	0.177	0.127	0.175
Relative change in the variance		-3%	-6%	+10%	-21%	+9%
β , median (95% CI)		0.244 (-0.007, 0.494)	-0.215 (-0.490, 0.111)	-0.049 (-0.224, 0.144)	0.269 (-0.026, 0.517)	-0.179 (-0.494, 0.118)
Interventions vs placebo	RR (95%CI)	RR (95%CI)	RR (95%CI)	RR (95%CI)	RR (95%CI)	RR (95%CI)
AOM	0.726 (0.501, 1.054)	**0.636 (0.423, 0.953)**	0.681 (0.464, 1.013)	0.724 (0.475, 1.100)	0.726 (0.509, 1.010)	0.702 (0.462, 1.067)
ARI	0.969 (0.697, 1.345)	0.931 (0.654, 1.308)	1.056 (0.712, 1.510)	0.961 (0.653, 1.426)	0.838 (0.614, 1.197)	0.924 (0.618, 1.354)
ARI+LAM	0.753 (0.502, 1.131)	0.772 (0.518, 1.161)	0.745 (0.496, 1.115)	0.770 (0.490, 1.212)	0.817 (0.552, 1.142)	0.762 (0.493, 1.177)
ARI+VAL	0.810 (0.429, 1.530)	0.864 (0.450, 1.687)	0.839 (0.441, 1.568)	0.855 (0.433, 1.673)	0.924 (0.488, 1.682)	0.824 (0.420, 1.631)
ASE	**0.450 (0.270, 0.750)**	**0.385 (0.222, 0.656)**	**0.389 (0.224, 0.672)**	**0.443 (0.253, 0.768)**	**0.462 (0.274, 0.750)**	**0.444 (0.251, 0.759)**
CAR	0.983 (0.714, 1.352)	1.043 (0.759, 1.438)	1.023 (0.731, 1.403)	1.019 (0.712, 1.438)	1.117 (0.797, 1.509)	1.000 (0.709, 1.396)
LAM	0.823 (0.670, 1.011)	0.843 (0.700, 1.007)	**0.811 (0.672, 0.972)**	0.840 (0.683, 1.017)	0.889 (0.738, 1.026)	0.828 (0.675, 1.006)
LAM+VAL	0.872 (0.538, 1.414)	0.882 (0.545, 1.430)	0.846 (0.510, 1.357)	0.879 (0.516, 1.481)	0.928 (0.588, 1.442)	0.867 (0.508, 1.445)
LIT	**0.837 (0.725, 0.966)**	0.872 (0.757, 1.004)	**0.851 (0.741, 0.968)**	**0.857 (0.726, 0.995)**	0.919 (0.788, 1.045)	**0.838 (0.724, 0.967)**
LIT+OXC	0.934 (0.428, 2.037)	0.906 (0.410, 1.984)	0.887 (0.395, 1.990)	0.893 (0.386, 1.975)	0.947 (0.436, 2.031)	0.875 (0.390, 1.945)
LIT+VAL	0.728 (0.522, 1.015)	0.756 (0.534, 1.063)	0.736 (0.521, 1.017)	0.744 (0.499, 1.083)	0.805 (0.575, 1.077)	0.720 (0.494, 1.053)
OLA	**0.686 (0.558, 0.843)**	**0.673 (0.539, 0.830)**	**0.682 (0.549, 0.834)**	**0.694 (0.535, 0.877)**	**0.718 (0.581, 0.854)**	**0.659 (0.511, 0.827)**
PAL	0.894 (0.637, 1.254)	0.821 (0.565, 1.196)	1.203 (0.669, 1.962)	0.897 (0.600, 1.343)	0.948 (0.689, 1.291)	0.857 (0.582, 1.264)
QUE	**0.657 (0.520, 0.830)**	**0.622 (0.483, 0.798)**	**0.660 (0.519, 0.836)**	**0.691 (0.497, 0.952)**	**0.680 (0.548, 0.840)**	**0.638 (0.477, 0.839)**
RISLAI	0.786 (0.615, 1.005)	**0.736 (0.569, 0.965)**	0.822 (0.630, 1.057)	0.792 (0.594, 1.053)	0.854 (0.664, 1.076)	**0.731 (0.533, 0.985)**
VAL	**0.798 (0.638, 0.999)**	0.829 (0.651, 1.038)	**0.800 (0.632, 0.992)**	0.817 (0.620, 1.043)	0.884 (0.697, 1.074)	0.788 (0.611, 1.006)

Sensitivity analysis

	Primary analysis	Focusing on studies including only BDI	Focusing on studies including only non rapid-cycling BD patients	Focusing on double-blind studies	Focusing on non-enriched studies	Focusing on non-industry sponsorship studies
Heterogeneity SD	0.161	0.157	0.152	0.171	0.147	0.166
Relative change in the variance		-2%	-6%	6%	-9%	+3%
Interventions vs placebo	RR (95%CI)	RR (95%CI)	RR (95%CI)	RR (95%CI)	RR (95%CI)	RR (95%CI)
AOM	0.726 (0.501, 1.054)	0.721 (0.487, 1.063)	0.724 (0.492, 1.056)	0.723 (0.478, 1.091)	0.718 (0.468, 1.098)	0.720 (0.461, 1.132)
ARI	0.969 (0.697, 1.345)	0.970 (0.676, 1.372)	0.965 (0.673, 1.388)	0.971 (0.649, 1.413)	0.966 (0.672, 1.350)	0.965 (0.657, 1.403)
ARI+LAM	0.753 (0.502, 1.131)	0.775 (0.509, 1.170)	0.783 (0.500, 1.203)	0.768 (0.492, 1.197)	0.793 (0.515, 1.189)	0.777 (0.491, 1.247)
ARI+VAL	0.810 (0.429, 1.530)	0.860 (0.438, 1.650)	0.877 (0.375, 2.014)	0.835 (0.430, 1.650)	0.882 (0.390, 1.993)	0.881 (0.373, 2.047)
ASE	**0.450 (0.270, 0.750)**	**0.444 (0.253, 0.734)**	**0.442 (0.258, 0.729)**	**0.443 (0.250, 0.752)**	**0.436 (0.223, 0.823)**	**0.436 (0.217, 0.824)**
CAR	0.983 (0.714, 1.352)	1.052 (0.715, 1.532)	1.049 (0.717, 1.527)	0.988 (0.689, 1.406)	1.038 (0.754, 1.427)	1.034 (0.721, 1.470)
LAM	0.823 (0.670, 1.011)	0.845 (0.694, 1.008)	0.851 (0.697, 1.021)	0.836 (0.689, 1.009)	0.856 (0.704, 1.024)	0.847 (0.685, 1.032)
LAM+VAL	0.872 (0.538, 1.414)	0.864 (0.475, 1.552)	0.871 (0.483, 1.570)	0.875 (0.518, 1.454)	0.882 (0.491, 1.548)	0.883 (0.523, 1.463)
LIT	**0.837 (0.725, 0.966)**	**0.853 (0.742, 0.976)**	**0.849 (0.735, 0.972)**	**0.841 (0.723, 0.968)**	**0.865 (0.756, 0.983)**	**0.846 (0.728, 0.975)**
LIT+OXC	0.934 (0.428, 2.037)	0.832 (0.283, 2.304)	0.834 (0.282, 2.264)	0.887 (0.387, 1.984)	0.902 (0.410, 1.991)	0.887 (0.394, 1.965)
LIT+VAL	0.728 (0.522, 1.015)	0.741 (0.521, 1.060)	0.736 (0.509, 1.047)	0.732 (0.493, 1.068)	0.752 (0.535, 1.032)	0.735 (0.502, 1.050)
OLA	**0.686 (0.558, 0.843)**	**0.693 (0.550, 0.848)**	**0.689 (0.548, 0.848)**	**0.685 (0.540, 0.856)**	**0.708 (0.561, 0.869)**	**0.700 (0.538, 0.877)**
PAL	0.894 (0.637, 1.254)	0.891 (0.621, 1.267)	0.894 (0.621, 1.258)	0.893 (0.614, 1.337)	0.893 (0.622, 1.278)	0.895 (0.603, 1.331)
QUE	**0.657 (0.520, 0.830)**	**0.649 (0.505, 0.837)**	**0.659 (0.505, 0.851)**	**0.656 (0.502, 0.852)**	**0.658 (0.511, 0.850)**	**0.654 (0.499, 0.865)**
RISLAI	0.786 (0.615, 1.005)	0.784 (0.605, 1.014)	0.783 (0.608, 1.017)	0.784 (0.596, 1.023)	0.777 (0.588, 1.023)	0.779 (0.576, 1.046)
VAL	**0.798 (0.638, 0.999)**	0.816 (0.635, 1.030)	0.816 (0.627, 1.022)	0.801 (0.616, 1.019)	0.824 (0.654, 1.012)	0.809 (0.628, 1.033)

1.5. Discontinuation due to adverse event (21 RCTs, 6107 patients)

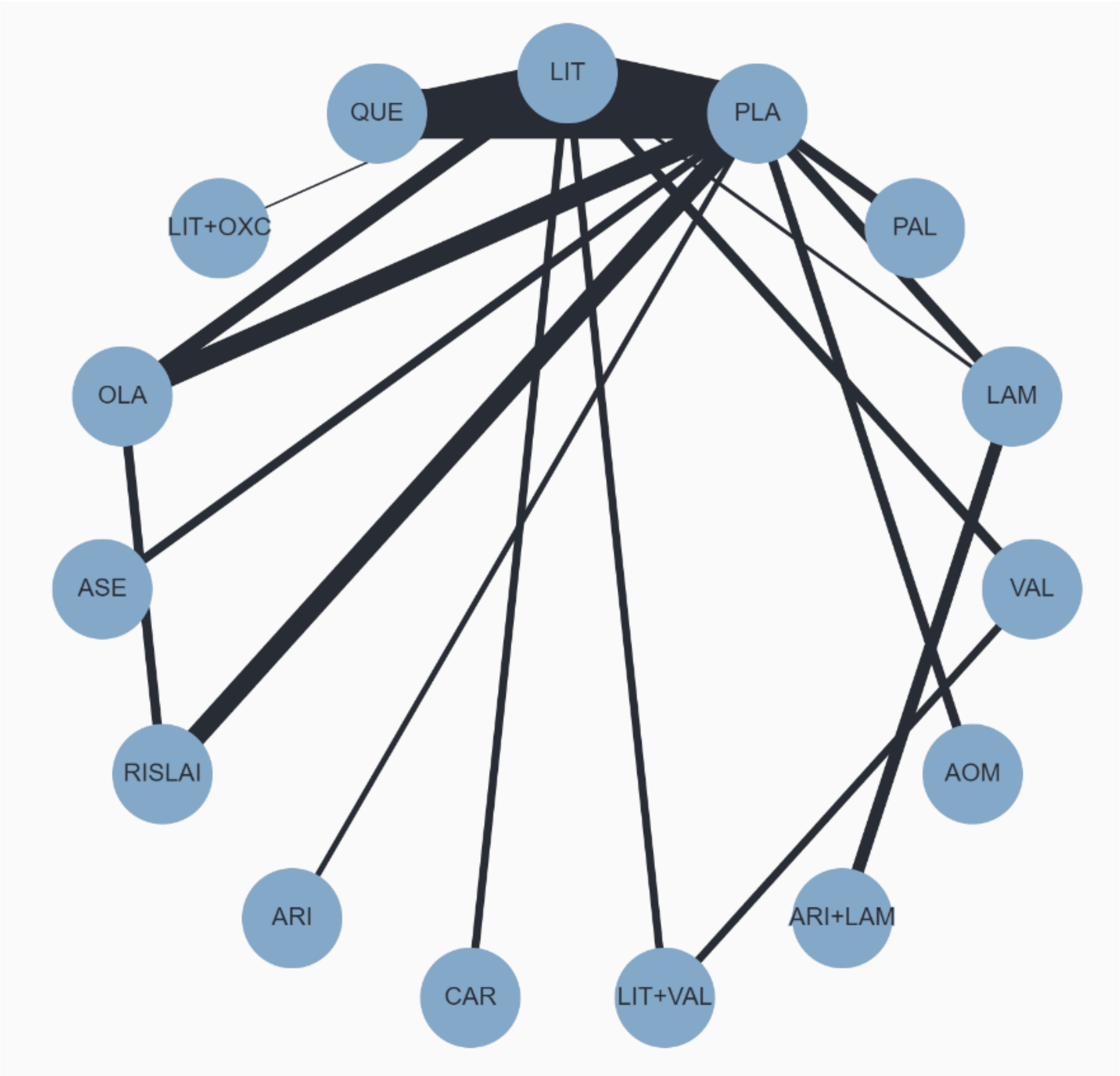

League table

Results from pairwise meta-analysis are presented in the left lower half and results from network meta-analysis in the upper right half.

The boldface result indicates statistical significance.

AOM	1.096 (0.055, 21.942)	8.156 (0.641, 103.804)	**19.292 (2.011, 185.045)**	2.524 (0.230, 27.735)	**12.682 (1.150, 139.826)**	3.128 (0.361, 27.105)	1.870 (0.116, 30.054)	1.917 (0.178, 20.606)	3.859 (0.427, 34.848)	5.751 (0.470, 70.337)	6.026 (0.686, 52.963)	3.914 (0.355, 43.216)	6.266 (0.555, 70.732)	7.000 (0.847, 57.876)
	ARI	7.439 (0.578, 95.679)	**17.595 (1.813, 170.784)**	2.302 (0.207, 25.582)	**11.568 (1.037, 128.960)**	2.853 (0.325, 25.028)	1.705 (0.105, 27.677)	1.749 (0.161, 19.009)	3.520 (0.385, 32.175)	5.246 (0.424, 64.845)	5.496 (0.618, 48.906)	3.570 (0.320, 39.857)	5.716 (0.501, 65.229)	6.385 (0.762, 53.464)
		ARI+LAM	2.366 (0.463, 12.078)	0.309 (0.054, 1.769)	1.555 (0.670, 3.612)	0.384 (0.095, 1.554)	0.229 (0.024, 2.149)	0.235 (0.042, 1.303)	0.473 (0.109, 2.063)	0.705 (0.100, 4.974)	0.739 (0.168, 3.246)	0.480 (0.081, 2.856)	0.768 (0.130, 4.557)	0.858 (0.208, 3.541)
			ASE	**0.131 (0.033, 0.525)**	0.657 (0.163, 2.655)	**0.162 (0.064, 0.408)**	**0.097 (0.013, 0.698)**	**0.099 (0.026, 0.384)**	**0.200 (0.073, 0.552)**	0.298 (0.062, 1.429)	**0.312 (0.120, 0.811)**	**0.203 (0.050, 0.821)**	0.325 (0.077, 1.365)	**0.363 (0.162, 0.812)**
				CAR	**5.025 (1.092, 23.120)**	1.239 (0.438, 3.508)	0.741 (0.097, 5.658)	0.760 (0.181, 3.190)	1.529 (0.486, 4.807)	2.279 (0.393, 13.217)	2.388 (0.726, 7.854)	1.551 (0.332, 7.246)	2.483 (0.546, 11.292)	2.773 (0.894, 8.609)
		1.555 (0.670, 3.612)			LAM	**0.247 (0.081, 0.753)**	0.147 (0.019, 1.172)	**0.151 (0.034, 0.672)**	0.304 (0.091, 1.018)	0.453 (0.078, 2.642)	0.475 (0.141, 1.604)	0.309 (0.064, 1.486)	0.494 (0.103, 2.370)	0.552 (0.177, 1.725)
				1.239 (0.438, 3.508)	**0.213 (0.060, 0.757)**	LIT	0.598 (0.104, 3.428)	0.613 (0.228, 1.647)	1.234 (0.765, 1.991)	1.839 (0.446, 7.583)	**1.927 (1.080, 3.437)**	1.251 (0.401, 3.903)	2.003 (0.666, 6.023)	**2.238 (1.430, 3.502)**
						0.598 (0.104, 3.428)	LIT+OXC	1.026 (0.138, 7.629)	2.064 (0.337, 12.627)	3.076 (0.325, 29.160)	3.223 (0.512, 20.296)	2.094 (0.260, 16.832)	3.352 (0.425, 26.417)	3.744 (0.617, 22.721)

						0.545 (0.196, 1.519)		LIT+VAL	2.013 (0.671, 6.033)	2.999 (0.533, 16.874)	3.143 (1.000, 9.877)	2.041 (0.452, 9.210)	**3.268 (1.088, 9.817)**	**3.651 (1.234, 10.801)**
						1.360 (0.819, 2.260)			OLA	1.490 (0.340, 6.540)	1.562 (0.754, 3.235)	1.014 (0.335, 3.071)	1.624 (0.489, 5.392)	1.814 (0.979, 3.361)
										PAL	1.048 (0.249, 4.415)	0.681 (0.117, 3.973)	1.090 (0.181, 6.554)	1.217 (0.317, 4.668)
						1.586 (0.742, 3.387)					QUE	0.650 (0.191, 2.210)	1.040 (0.300, 3.606)	1.162 (0.696, 1.937)
									1.008 (0.284, 3.581)			RISLAI	1.601 (0.329, 7.794)	1.788 (0.571, 5.605)
						2.003 (0.666, 6.023)		2.750 (0.853, 8.862)					VAL	1.117 (0.340, 3.666)
7.000 (0.847, 57.876)	6.385 (0.762, 53.464)		**0.363 (0.162, 0.812)**		0.898 (0.236, 3.412)	2.179 (1.322, 3.592)			4.426 (1.057, 18.541)	1.217 (0.317, 4.668)	1.111 (0.645, 1.912)	1.981 (0.476, 8.256)		PLA

Evaluation of heterogeneity and inconsistency

Between study variance (τ^2)	Heterogeneity assessment	Random-effects design-by-treatment interaction model		
		Q	df	p
0.034	Low to moderate	5.635	8	0.699

Inconsistency

	NMA, RR (95%CI)	Direct, RR (95%CI)	Indirect, RR (95%CI)	Inconsistency measures	
				Ratio of risk ratios	P value
LAM vs LIT	0.247 (0.081, 0.753)	0.213 (0.060, 0.757)	0.410 (0.039, 4.297)	0.519 (0.036, 7.490)	0.630
LAM vs PLA	0.552 (0.177, 1.725)	0.898 (0.236, 3.412)	0.149 (0.017, 1.332)	6.013 (0.463, 78.038)	0.170
LIT vs LIT+VAL	0.613 (0.228, 1.647)	0.545 (0.196, 1.519)	2.915 (0.069, 123.100)	0.187 (0.004, 9.067)	0.397
LIT vs OLA	1.234 (0.765, 1.991)	1.360 (0.819, 2.260)	0.564 (0.134, 2.375)	2.410 (0.525, 11.065)	0.258
LIT vs QUE	1.927 (1.080, 3.437)	1.586 (0.742, 3.387)	2.525 (1.033, 6.174)	0.628 (0.194, 2.029)	0.437
LIT vs PLA	2.238 (1.430, 3.502)	2.179 (1.322, 3.592)	2.494 (0.912, 6.824)	0.874 (0.284, 2.688)	0.814
LIT+VAL vs VAL	3.268 (1.088, 9.817)	2.750 (0.853, 8.862)	12.084 (0.482, 302.869)	0.228 (0.007, 7.008)	0.397
OLA vs RISLAI	1.014 (0.335, 3.071)	1.008 (0.284, 3.581)	1.036 (0.106, 10.103)	0.973 (0.072, 13.184)	0.983
OLA vs PLA	1.814 (0.979, 3.361)	4.426 (1.057, 18.541)	1.481 (0.748, 2.932)	2.990 (0.612, 14.617)	0.176
QUE vs PLA	1.162 (0.696, 1.937)	1.111 (0.645, 1.912)	1.648 (0.360, 7.552)	0.674 (0.134, 3.395)	0.633
RISLAI vs PLA	1.788 (0.571, 5.605)	1.981 (0.476, 8.256)	1.489 (0.221, 10.021)	1.330 (0.123, 14.392)	0.814

Rank probabilities

	Rank 1	Rank 2	Rank 3	Rank 4	Rank 5	Rank 6	Rank 7	Rank 8	Rank 9	Rank 10	Rank 11	Rank 12	Rank 13	Rank 14	Rank 15
AOM	0.003	0.006	0.013	0.016	0.018	0.021	0.029	0.044	0.043	0.048	0.054	0.077	0.117	0.181	0.330
ARI	0.006	0.010	0.015	0.021	0.025	0.028	0.035	0.045	0.057	0.057	0.063	0.084	0.117	0.174	0.265
ARI+LAM	0.094	0.143	0.155	0.123	0.084	0.066	0.065	0.060	0.051	0.036	0.032	0.032	0.029	0.021	0.010
ASE	0.444	0.205	0.158	0.082	0.039	0.021	0.015	0.008	0.007	0.006	0.005	0.004	0.002	0.003	0.001
CAR	0.001	0.005	0.007	0.011	0.018	0.021	0.031	0.051	0.058	0.075	0.097	0.143	0.186	0.190	0.108
LAM	0.231	0.295	0.200	0.105	0.063	0.040	0.025	0.017	0.009	0.006	0.006	0.002	0.001	0.001	0.000
LIT	0.000	0.000	0.000	0.002	0.003	0.013	0.033	0.078	0.161	0.243	0.244	0.157	0.053	0.011	0.002
LIT+OXC	0.024	0.034	0.034	0.041	0.043	0.050	0.055	0.064	0.066	0.061	0.067	0.093	0.110	0.130	0.128
LIT+VAL	0.003	0.007	0.012	0.014	0.021	0.029	0.034	0.053	0.065	0.077	0.107	0.136	0.170	0.166	0.107
OLA	0.000	0.001	0.003	0.007	0.011	0.026	0.060	0.110	0.165	0.177	0.150	0.126	0.099	0.049	0.017
PAL	0.073	0.093	0.098	0.114	0.097	0.082	0.083	0.070	0.058	0.048	0.049	0.046	0.040	0.032	0.017
QUE	0.008	0.028	0.059	0.105	0.147	0.166	0.161	0.133	0.080	0.048	0.029	0.019	0.011	0.006	0.001
RISLAI	0.018	0.041	0.059	0.081	0.089	0.085	0.099	0.110	0.098	0.079	0.072	0.065	0.056	0.035	0.014
VAL	0.096	0.125	0.143	0.160	0.116	0.091	0.083	0.064	0.048	0.028	0.021	0.014	0.008	0.003	0.001
PLA	0.001	0.007	0.045	0.120	0.227	0.260	0.193	0.095	0.034	0.012	0.005	0.002	0.000	0.000	0.000

SUCRA

ASE	0.91
LAM	0.83
PLA	0.73
ARI+LAM	0.68
VAL	0.65
QUE	0.64
PAL	0.63
RISLAI	0.45
LIT	0.37
CAR	0.34
OLA	0.33
LIT+OXC	0.27
LIT+VAL	0.27
ARI	0.20
AOM	0.18

Comparison-adjusted funnel plot of studies of drugs versus placebo

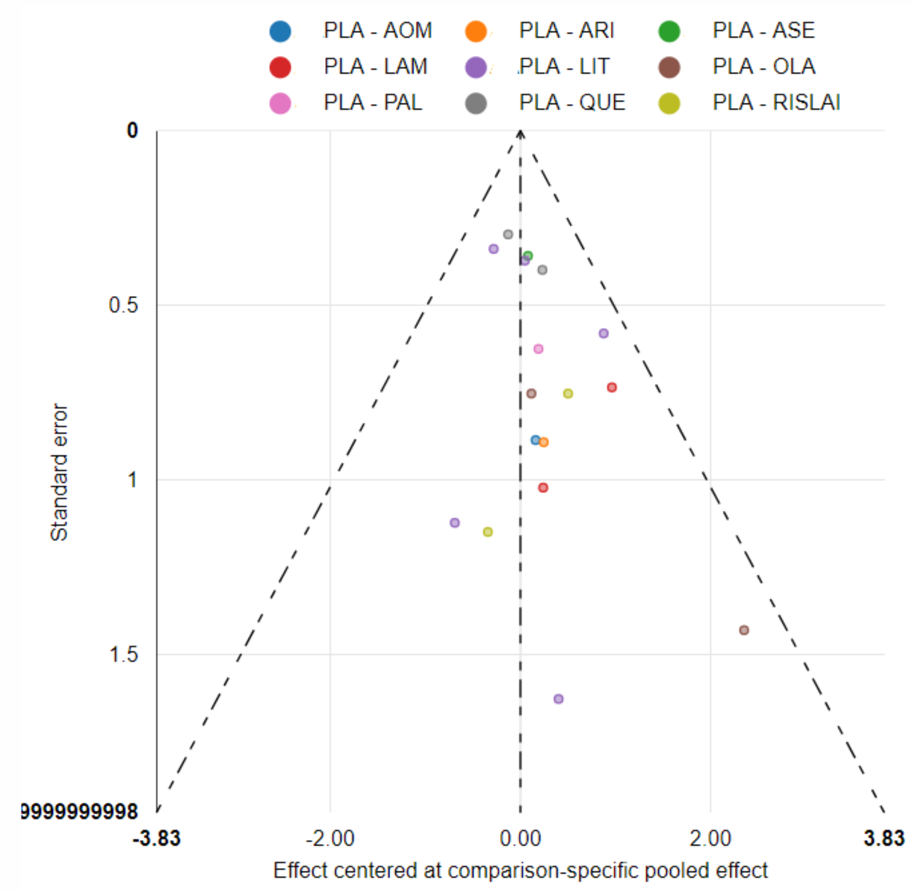

CINeMA rating

Comparison	Number of studies	Within-study bias	Reporting bias	Indirectness	Imprecision	Heterogeneity	Incoherence	Confidence rating
AOM:PLA	1	No concerns	Suspected	No concerns	Major concerns	No concerns	No concerns	Low
ARI:PLA	1	No concerns	Suspected	No concerns	Major concerns	No concerns	No concerns	Low
ARI+LAM:LAM	1	No concerns	Suspected	No concerns	Major concerns	No concerns	No concerns	Low
ASE:PLA	1	No concerns	Suspected	No concerns	No concerns	No concerns	No concerns	Moderate
CAR:LIT	2	Major concerns	Suspected	No concerns	Major concerns	No concerns	No concerns	Very low
LAM:LIT	1	Some concerns	Suspected	No concerns	No concerns	No concerns	No concerns	Low
LAM:PLA	2	Some concerns	Suspected	No concerns	Major concerns	No concerns	No concerns	Very low
LIT:LIT+OXC	1	No concerns	Suspected	No concerns	Major concerns	No concerns	No concerns	Low
LIT:LIT+VAL	1	Major concerns	Suspected	No concerns	Major concerns	No concerns	No concerns	Very low
LIT:OLA	1	No concerns	Suspected	No concerns	Major concerns	No concerns	No concerns	Low
LIT:PLA	5	Some concerns	Suspected	No concerns	No concerns	No concerns	No concerns	Low
LIT:QUE	1	Some concerns	Suspected	No concerns	No concerns	Major concerns	No concerns	Very low
LIT:VAL	2	Major concerns	Suspected	No concerns	Major concerns	No concerns	No concerns	Very low
LIT+VAL:VAL	1	Major concerns	Suspected	No concerns	No concerns	Major concerns	No concerns	Very low
OLA:PLA	2	No concerns	Suspected	No concerns	Major concerns	No concerns	No concerns	Low
OLA:RISLAI	1	No concerns	Suspected	No concerns	Major concerns	No concerns	No concerns	Low
PAL:PLA	1	No concerns	Suspected	No concerns	Major concerns	No concerns	No concerns	Low
PLA:QUE	2	No concerns	Suspected	No concerns	Major concerns	No concerns	No concerns	Low
PLA:RISLAI	2	No concerns	Suspected	No concerns	Major concerns	No concerns	No concerns	Low
AOM:ARI	0	No concerns	Suspected	No concerns	Major concerns	No concerns	No concerns	Low
AOM:ARI+LAM	0	No concerns	Suspected	No concerns	Major concerns	No concerns	No concerns	Low
AOM:ASE	0	No concerns	Suspected	No concerns	No concerns	No concerns	No concerns	Moderate
AOM:CAR	0	No concerns	Suspected	No concerns	Major concerns	No concerns	No concerns	Low
AOM:LAM	0	Some concerns	Suspected	No concerns	No concerns	Major concerns	No concerns	Very low
AOM:LIT	0	No concerns	Suspected	No concerns	Major concerns	No concerns	No concerns	Low
AOM:LIT+OXC	0	No concerns	Suspected	No concerns	Major concerns	No concerns	No concerns	Low
AOM:LIT+VAL	0	No concerns	Suspected	No concerns	Major concerns	No concerns	No concerns	Low
AOM:OLA	0	No concerns	Suspected	No concerns	Major concerns	No concerns	No concerns	Low
AOM:PAL	0	No concerns	Suspected	No concerns	Major concerns	No concerns	No concerns	Low
AOM:QUE	0	No concerns	Suspected	No concerns	Major concerns	No concerns	No concerns	Low
AOM:RISLAI	0	No concerns	Suspected	No concerns	Major concerns	No concerns	No concerns	Low
AOM:VAL	0	No concerns	Suspected	No concerns	Major concerns	No concerns	No concerns	Low

ARI:ARI+LAM	0	No concerns	Suspected	No concerns	Major concerns	No concerns	No concerns	Low
ARI:ASE	0	No concerns	Suspected	No concerns	No concerns	No concerns	No concerns	Moderate
ARI:CAR	0	No concerns	Suspected	No concerns	Major concerns	No concerns	No concerns	Low
ARI:LAM	0	Some concerns	Suspected	No concerns	No concerns	Major concerns	No concerns	Very low
ARI:LIT	0	No concerns	Suspected	No concerns	Major concerns	No concerns	No concerns	Low
ARI:LIT+OXC	0	No concerns	Suspected	No concerns	Major concerns	No concerns	No concerns	Low
ARI:LIT+VAL	0	No concerns	Suspected	No concerns	Major concerns	No concerns	No concerns	Low
ARI:OLA	0	No concerns	Suspected	No concerns	Major concerns	No concerns	No concerns	Low
ARI:PAL	0	No concerns	Suspected	No concerns	Major concerns	No concerns	No concerns	Low
ARI:QUE	0	No concerns	Suspected	No concerns	Major concerns	No concerns	No concerns	Low
ARI:RISLAI	0	No concerns	Suspected	No concerns	Major concerns	No concerns	No concerns	Low
ARI:VAL	0	No concerns	Suspected	No concerns	Major concerns	No concerns	No concerns	Low
ARI+LAM:ASE	0	No concerns	Suspected	No concerns	Major concerns	No concerns	No concerns	Low
ARI+LAM:CAR	0	No concerns	Suspected	No concerns	Major concerns	No concerns	No concerns	Low
ARI+LAM:LIT	0	Some concerns	Suspected	No concerns	Major concerns	No concerns	No concerns	Very low
ARI+LAM:LIT+OXC	0	No concerns	Suspected	No concerns	Major concerns	No concerns	No concerns	Low
ARI+LAM:LIT+VAL	0	Some concerns	Suspected	No concerns	Major concerns	No concerns	No concerns	Very low
ARI+LAM:OLA	0	No concerns	Suspected	No concerns	Major concerns	No concerns	No concerns	Low
ARI+LAM:PAL	0	No concerns	Suspected	No concerns	Major concerns	No concerns	No concerns	Low
ARI+LAM:PLA	0	Some concerns	Suspected	No concerns	Major concerns	No concerns	No concerns	Very low
ARI+LAM:QUE	0	Some concerns	Suspected	No concerns	Major concerns	No concerns	No concerns	Very low
ARI+LAM:RISLAI	0	No concerns	Suspected	No concerns	Major concerns	No concerns	No concerns	Low
ARI+LAM:VAL	0	No concerns	Suspected	No concerns	Major concerns	No concerns	No concerns	Low
ASE:CAR	0	No concerns	Suspected	No concerns	No concerns	No concerns	No concerns	Moderate
ASE:LAM	0	Some concerns	Suspected	No concerns	Major concerns	No concerns	No concerns	Very low
ASE:LIT	0	No concerns	Suspected	No concerns	No concerns	No concerns	No concerns	Moderate
ASE:LIT+OXC	0	No concerns	Suspected	No concerns	No concerns	No concerns	No concerns	Moderate
ASE:LIT+VAL	0	No concerns	Suspected	No concerns	No concerns	No concerns	No concerns	Moderate
ASE:OLA	0	No concerns	Suspected	No concerns	No concerns	No concerns	No concerns	Moderate
ASE:PAL	0	No concerns	Suspected	No concerns	Major concerns	No concerns	No concerns	Low
ASE:QUE	0	No concerns	Suspected	No concerns	No concerns	No concerns	No concerns	Moderate
ASE:RISLAI	0	No concerns	Suspected	No concerns	No concerns	Major concerns	No concerns	Low
ASE:VAL	0	No concerns	Suspected	No concerns	Major concerns	No concerns	No concerns	Low

CAR:LAM	0	Some concerns	Suspected	No concerns	No concerns	Major concerns	No concerns	Very low
CAR:LIT+OXC	0	No concerns	Suspected	No concerns	Major concerns	No concerns	No concerns	Low
CAR:LIT+VAL	0	Major concerns	Suspected	No concerns	Major concerns	No concerns	No concerns	Very low
CAR:OLA	0	No concerns	Suspected	No concerns	Major concerns	No concerns	No concerns	Low
CAR:PAL	0	No concerns	Suspected	No concerns	Major concerns	No concerns	No concerns	Low
CAR:PLA	0	Some concerns	Suspected	No concerns	Major concerns	No concerns	No concerns	Very low
CAR:QUE	0	Some concerns	Suspected	No concerns	Major concerns	No concerns	No concerns	Very low
CAR:RISLAI	0	No concerns	Suspected	No concerns	Major concerns	No concerns	No concerns	Low
CAR:VAL	0	Major concerns	Suspected	No concerns	Major concerns	No concerns	No concerns	Very low
LAM:LIT+OXC	0	Some concerns	Suspected	No concerns	Major concerns	No concerns	No concerns	Very low
LAM:LIT+VAL	0	Some concerns	Suspected	No concerns	No concerns	No concerns	No concerns	Low
LAM:OLA	0	Some concerns	Suspected	No concerns	Major concerns	No concerns	No concerns	Very low
LAM:PAL	0	Some concerns	Suspected	No concerns	Major concerns	No concerns	No concerns	Very low
LAM:QUE	0	Some concerns	Suspected	No concerns	Major concerns	No concerns	No concerns	Very low
LAM:RISLAI	0	No concerns	Suspected	No concerns	Major concerns	No concerns	No concerns	Low
LAM:VAL	0	Some concerns	Suspected	No concerns	Major concerns	No concerns	No concerns	Very low
LIT:PAL	0	No concerns	Suspected	No concerns	Major concerns	No concerns	No concerns	Low
LIT:RISLAI	0	No concerns	Suspected	No concerns	Major concerns	No concerns	No concerns	Low
LIT+OXC:LIT+VAL	0	Major concerns	Suspected	No concerns	Major concerns	No concerns	No concerns	Very low
LIT+OXC:OLA	0	No concerns	Suspected	No concerns	Major concerns	No concerns	No concerns	Low
LIT+OXC:PAL	0	No concerns	Suspected	No concerns	Major concerns	No concerns	No concerns	Low
LIT+OXC:PLA	0	No concerns	Suspected	No concerns	Major concerns	No concerns	No concerns	Low
LIT+OXC:QUE	0	No concerns	Suspected	No concerns	Major concerns	No concerns	No concerns	Low
LIT+OXC:RISLAI	0	No concerns	Suspected	No concerns	Major concerns	No concerns	No concerns	Low
LIT+OXC:VAL	0	No concerns	Suspected	No concerns	Major concerns	No concerns	No concerns	Low
LIT+VAL:OLA	0	No concerns	Suspected	No concerns	Major concerns	No concerns	No concerns	Low
LIT+VAL:PAL	0	No concerns	Suspected	No concerns	Major concerns	No concerns	No concerns	Low
LIT+VAL:PLA	0	Major concerns	Suspected	No concerns	No concerns	Major concerns	No concerns	Very low
LIT+VAL:QUE	0	Major concerns	Suspected	No concerns	Major concerns	No concerns	No concerns	Very low
LIT+VAL:RISLAI	0	No concerns	Suspected	No concerns	Major concerns	No concerns	No concerns	Low
OLA:PAL	0	No concerns	Suspected	No concerns	Major concerns	No concerns	No concerns	Low
OLA:QUE	0	No concerns	Suspected	No concerns	Major concerns	No concerns	No concerns	Low
OLA:VAL	0	No concerns	Suspected	No concerns	Major concerns	No concerns	No concerns	Low

PAL:QUE	0	No concerns	Suspected	No concerns	Major concerns	No concerns	No concerns	Low
PAL:RISLAI	0	No concerns	Suspected	No concerns	Major concerns	No concerns	No concerns	Low
PAL:VAL	0	No concerns	Suspected	No concerns	Major concerns	No concerns	No concerns	Low
PLA:VAL	0	Some concerns	Suspected	No concerns	Major concerns	No concerns	No concerns	Very low
QUE:RISLAI	0	No concerns	Suspected	No concerns	Major concerns	No concerns	No concerns	Low
QUE:VAL	0	Some concerns	Suspected	No concerns	Major concerns	No concerns	No concerns	Very low
RISLAI:VAL	0	No concerns	Suspected	No concerns	Major concerns	No concerns	No concerns	Low

Meta-regression analysis

	Primary analysis	Publication year	Duration of study	Number of total patients	Percent female	Mean age
Heterogeneity SD	0.184	0.506	0.548	0.556	0.499	0.555
Relative change in the variance		+175%	+198%	+202%	+171%	+202%
β , median (95% CI)		-0.030 (-1.566, 1.529)	-0.173 (-2.044, 1.581)	0.030 (-0.858, 1.016)	0.744 (-0.758, 2.345)	0.084 (-1.140, 1.584)
Interventions vs placebo	RR (95%CI)	RR (95%CI)	RR (95%CI)	RR (95%CI)	RR (95%CI)	RR (95%CI)
AOM	7.000 (0.847, 57.876)	4.120 (0.452, 50.658)	3.883 (0.430, 45.114)	4.110 (0.498, 44.598)	3.352 (0.474, 40.674)	3.858 (0.515, 44.710)
ARI	6.385 (0.762, 53.464)	3.685 (0.485, 44.975)	3.901 (0.428, 47.418)	3.739 (0.456, 42.449)	2.075 (0.171, 29.699)	3.674 (0.479, 51.096)
ARI+LAM	0.858 (0.208, 3.541)	0.740 (0.081, 6.542)	0.707 (0.064, 6.567)	0.743 (0.067, 6.586)	0.774 (0.091, 5.992)	0.772 (0.070, 6.854)
ASE	**0.363 (0.162, 0.812)**	0.347 (0.055, 2.099)	0.301 (0.036, 2.529)	0.346 (0.066, 1.819)	0.339 (0.069, 1.520)	0.333 (0.055, 1.667)
CAR	2.773 (0.894, 8.609)	3.087 (0.646, 13.799)	3.036 (0.579, 13.892)	2.969 (0.573, 14.172)	3.324 (0.723, 15.034)	2.984 (0.597, 14.002)
LAM	0.552 (0.177, 1.725)	0.446 (0.091, 1.801)	0.423 (0.073, 1.906)	0.444 (0.084, 1.907)	0.474 (0.098, 1.800)	0.460 (0.091, 2.000)
LIT	**2.238 (1.430, 3.502)**	2.108 (0.955, 4.386)	2.076 (0.932, 4.285)	2.071 (0.848, 4.675)	**2.268 (1.080, 4.855)**	2.083 (0.923, 4.334)
LIT+OXC	3.744 (0.617, 22.721)	2.470 (0.232, 21.170)	2.344 (0.231, 21.321)	2.436 (0.226, 26.768)	2.666 (0.256, 25.361)	2.489 (0.249, 24.247)
LIT+VAL	**3.651 (1.234, 10.801)**	3.003 (0.459, 14.611)	2.846 (0.411, 16.240)	2.829 (0.385, 15.099)	3.116 (0.565, 16.187)	2.886 (0.426, 16.273)
OLA	1.814 (0.979, 3.361)	2.082 (0.809, 6.541)	2.050 (0.758, 6.761)	2.091 (0.785, 6.920)	2.120 (0.857, 6.788)	2.134 (0.813, 7.562)
PAL	1.217 (0.317, 4.668)	0.994 (0.153, 6.493)	1.207 (0.061, 29.006)	0.985 (0.140, 6.460)	1.033 (0.175, 6.956)	0.997 (0.141, 7.003)
QUE	1.162 (0.696, 1.937)	1.121 (0.363, 3.220)	1.072 (0.371, 3.282)	1.071 (0.286, 4.044)	1.032 (0.378, 2.934)	1.101 (0.378, 3.249)
RISLAI	1.788 (0.571, 5.605)	1.366 (0.274, 5.706)	1.407 (0.295, 6.430)	1.360 (0.276, 6.290)	1.587 (0.344, 6.969)	1.447 (0.285, 7.244)
VAL	1.117 (0.340, 3.666)	0.784 (0.130, 3.395)	0.747 (0.119, 3.384)	0.758 (0.112, 3.472)	0.838 (0.157, 3.740)	0.748 (0.117, 3.520)

Sensitivity analysis

	Primary analysis	Focusing on studies including only BDI	Focusing on studies including only non rapid-cycling BD patients	Focusing on double-blind studies	Focusing on non-enriched studies	Focusing on non-industry sponsorship studies
Heterogeneity SD	0.184	0.452	0.252	0.405	0.310	0.277
Relative change in the variance		+146%	+37%	+120%	+68%	+51%
Interventions vs placebo	RR (95% CrI)	RR (95% CrI)	RR (95% CrI)	RR (95% CrI)	RR (95% CrI)	RR (95% CrI)
AOM	7.000 (0.847, 57.876)	3.955 (0.606, 55.263)	4.250 (0.713, 40.193)	3.900 (0.608, 40.169)	2.667 (0.318, 36.492)	2.835 (0.315, 33.341)
ARI	6.385 (0.762, 53.464)	3.673 (0.501, 40.399)	2.413 (0.303, 35.971)	3.683 (0.579, 37.341)	2.497 (0.263, 34.758)	2.693 (0.301, 44.934)
ARI+LAM	0.858 (0.208, 3.541)	0.744 (0.080, 6.453)	0.633 (0.061, 5.713)	0.793 (0.099, 4.953)	0.640 (0.051, 5.794)	0.609 (0.052, 5.582)
ASE	**0.363 (0.162, 0.812)**	0.333 (0.077, 1.420)	0.344 (0.110, 1.039)	0.335 (0.083, 1.294)	0.311 (0.065, 1.288)	0.315 (0.071, 1.230)
CAR	2.773 (0.894, 8.609)	3.707 (0.628, 21.141)	3.370 (0.691, 19.373)	2.642 (0.599, 12.339)	2.760 (0.615, 10.153)	3.556 (0.898, 14.122)
LAM	0.552 (0.177, 1.725)	0.453 (0.086, 1.777)	0.362 (0.050, 1.795)	0.474 (0.109, 1.674)	0.368 (0.047, 1.754)	0.334 (0.045, 1.774)
LIT	**2.238 (1.430, 3.502)**	2.083 (0.951, 4.175)	1.829 (0.929, 3.455)	2.126 (1.067, 3.965)	1.786 (0.868, 3.417)	1.795 (0.895, 3.495)
LIT+OXC	3.744 (0.617, 22.721)	1.751 (0.074, 23.917)	1.582 (0.108, 18.769)	2.457 (0.281, 20.116)	2.110 (0.262, 14.927)	2.101 (0.264, 16.898)
LIT+VAL	**3.651 (1.234, 10.801)**	2.993 (0.495, 14.693)	2.268 (0.445, 11.617)	2.555 (0.424, 14.690)	2.484 (0.544, 9.529)	2.474 (0.595, 10.119)
OLA	1.814 (0.979, 3.361)	2.015 (0.854, 6.360)	1.575 (0.722, 3.824)	1.982 (0.914, 5.638)	1.498 (0.654, 4.066)	1.526 (0.665, 4.061)
PAL	1.217 (0.317, 4.668)	0.996 (0.180, 6.406)	0.996 (0.212, 4.607)	0.995 (0.175, 5.646)	0.782 (0.081, 6.006)	0.815 (0.103, 5.996)
QUE	1.162 (0.696, 1.937)	1.117 (0.405, 2.923)	1.001 (0.421, 2.267)	1.093 (0.447, 2.646)	1.004 (0.401, 2.389)	0.991 (0.414, 2.397)
RISLAI	1.788 (0.571, 5.605)	1.369 (0.325, 5.472)	1.267 (0.350, 4.196)	1.350 (0.343, 5.150)	0.942 (0.143, 4.692)	0.991 (0.163, 4.574)
VAL	1.117 (0.340, 3.666)	0.816 (0.130, 3.628)	0.561 (0.073, 2.696)	0.635 (0.096, 3.063)	0.679 (0.134, 2.361)	0.655 (0.153, 2.573)

1.6. Mortality rate (11 RCTs, 4703 patients)

League table (odds ratio)

Results from pairwise meta-analysis are presented in the left lower half and results from network meta-analysis in the upper right half.

The boldface result indicates statistical significance.

AOM	1.950 (0.006, 611.674)	3.022 (0.019, 482.075)	1.895 (0.028, 126.318)	5.391 (0.130, 223.408)	2.645 (0.044, 159.892)	1.578 (0.024, 103.265)	0.617 (0.007, 51.511)	3.465 (0.071, 168.056)	2.176 (0.017, 282.083)	3.560 (0.057, 223.699)	3.046 (0.123, 75.445)
	ARI+LAM	1.550 (0.003, 747.026)	0.972 (0.019, 49.259)	2.765 (0.023, 325.642)	1.357 (0.009, 215.746)	0.809 (0.004, 156.132)	0.317 (0.001, 90.731)	1.777 (0.010, 314.536)	1.116 (0.003, 413.642)	1.826 (0.011, 299.645)	1.562 (0.013, 183.975)
		ASE	0.627 (0.005, 74.010)	1.784 (0.023, 139.379)	0.875 (0.008, 94.812)	0.522 (0.004, 60.637)	0.204 (0.001, 29.412)	1.147 (0.013, 102.545)	0.720 (0.003, 154.054)	1.178 (0.011, 132.013)	1.008 (0.020, 51.188)
	0.972 (0.019, 49.259)		LAM	2.844 (0.190, 42.657)	1.396 (0.056, 34.488)	0.833 (0.025, 27.699)	0.326 (0.006, 19.163)	1.828 (0.063, 53.367)	1.148 (0.014, 95.890)	1.879 (0.072, 48.769)	1.607 (0.107, 24.100)
			2.138 (0.086, 52.921)	LIT	0.491 (0.088, 2.736)	0.293 (0.024, 3.593)	0.115 (0.003, 4.121)	0.643 (0.046, 8.896)	0.404 (0.009, 18.673)	0.660 (0.108, 4.032)	0.565 (0.085, 3.736)
				0.491 (0.088, 2.736)	LIT+VAL	0.597 (0.029, 12.467)	0.233 (0.004, 12.414)	1.310 (0.057, 30.250)	0.823 (0.012, 54.949)	1.346 (0.294, 6.159)	1.151 (0.090, 14.800)
				0.201 (0.010, 4.210)		OLA	0.391 (0.007, 22.579)	2.195 (0.079, 60.971)	1.379 (0.036, 53.346)	2.256 (0.102, 49.665)	1.930 (0.132, 28.121)
							PAL	5.613 (0.132, 237.817)	3.525 (0.030, 410.510)	5.768 (0.104, 319.354)	4.934 (0.235, 103.638)
				1.110 (0.022, 56.070)				QUE	0.628 (0.009, 42.085)	1.028 (0.042, 24.961)	0.879 (0.099, 7.797)
						1.008 (0.020, 51.157)			RISLAI	1.636 (0.024, 113.488)	1.399 (0.036, 54.136)
				0.660 (0.108, 4.032)	1.346 (0.294, 6.159)					VAL	0.855 (0.063, 11.698)

3.046 (0.123, 75.445)		1.008 (0.020, 51.188)	2.138 (0.086, 52.921)	0.650 (0.080, 5.306)		1.030 (0.020, 52.311	4.934 (0.235, 103.638)	1.007 (0.104, 9.709)	1.023 (0.020, 51.915)		PLA

Evaluation of heterogeneity and inconsistency

Between study variance (τ^2)	Heterogeneity assessment	Random-effects design-by-treatment interaction model		
		Q	df	p
0.01	Low	0.472	4	0.976

Inconsistency

	NMA, RR (95%CI)	Direct, RR (95%CI)	Indirect, RR (95%CI)	Inconsistency measures	
				Ratio of risk ratios	P value
LAM vs LIT	2.844 (0.190, 42.657)	2.138 (0.086, 52.921)	5.762 (0.037, 895.874)	0.371 (0.001, 146.775)	0.745
LAM vs PLA	2.138 (0.086, 52.921)	1.607 (0.107, 24.100)	0.793 (0.005, 123.322)	2.695 (0.007, 1066.353)	0.756
LIT vs OLA	0.293 (0.024, 3.593)	0.201 (0.010, 4.210)	0.650 (0.008, 54.348)	0.309 (0.001, 66.533)	0.668
LIT vs QUE	0.643 (0.046, 8.896)	1.110 (0.022, 56.070)	0.412 (0.012, 14.188)	2.693 (0.014, 530.330)	0.713
LIT vs PLA	0.565 (0.085, 3.736)	0.650 (0.080, 5.306)	0.313 (0.004, 23.587)	2.077 (0.017, 253.966)	0.766
OLA vs RISLAI	1.379 (0.036, 53.346)	1.008 (0.020, 51.157)	10.534 (0.000, 231723.470)	0.096 (0.000, 4426.215)	0.668
OLA vs PLA	1.930 (0.132, 28.121)	1.030 (0.020, 52.311	3.332 (0.085, 130.035)	0.309 (0.001, 66.533)	0.668
QUE vs PLA	0.879 (0.099, 7.797)	1.007 (0.104, 9.709)	0.154 (0.000, 516.306)	6.555 (0.001, 30055.471)	0.662
RISLAI vs PLA	1.399 (0.036, 54.136)	1.023 (0.020, 51.915)	10.693 (0.000, 235390.237	0.096 (0.000, 4428.428)	0.668

Rank probabilities

	Rank 1	Rank 2	Rank 3	Rank 4	Rank 5	Rank 6	Rank 7	Rank 8	Rank 9	Rank 10	Rank 11	Rank 12
AOM	0.000	0.001	0.002	0.002	0.009	0.014	0.048	0.097	0.193	0.197	0.246	0.191
ARI+LAM	0.044	0.028	0.012	0.031	0.048	0.037	0.022	0.034	0.054	0.081	0.156	0.454
ASE	0.231	0.078	0.016	0.098	0.128	0.103	0.058	0.075	0.061	0.077	0.055	0.020
LAM	0.000	0.000	0.001	0.003	0.013	0.026	0.045	0.116	0.244	0.253	0.216	0.083
LIT	0.259	0.326	0.250	0.115	0.036	0.011	0.003	0.001	0.000	0.000	0.000	0.000
LIT+VAL	0.082	0.195	0.339	0.227	0.100	0.038	0.013	0.006	0.001	0.001	0.000	0.000
OLA	0.005	0.011	0.019	0.196	0.204	0.122	0.076	0.099	0.103	0.077	0.050	0.038
PAL	0.000	0.000	0.001	0.002	0.004	0.016	0.049	0.113	0.193	0.235	0.221	0.167
QUE	0.004	0.019	0.014	0.036	0.132	0.244	0.280	0.193	0.067	0.011	0.001	0.000
RISLAI	0.238	0.073	0.023	0.083	0.138	0.106	0.053	0.069	0.053	0.064	0.054	0.047
VAL	0.138	0.266	0.312	0.176	0.067	0.025	0.011	0.004	0.001	0.000	0.000	0.000
PLA	0.001	0.004	0.013	0.032	0.123	0.258	0.344	0.192	0.031	0.003	0.001	0.000

SUCRA

LIT	0.87
VAL	0.82
LIT+VAL	0.78
RISLAI	0.66
QUE	0.60
PLA	0.59
ASE	0.43
OLA	0.37
AOM	0.23
LAM	0.23
PAL	0.23
ARI+LAM	0.19

Comparison-adjusted funnel plot of studies of drugs versus placebo

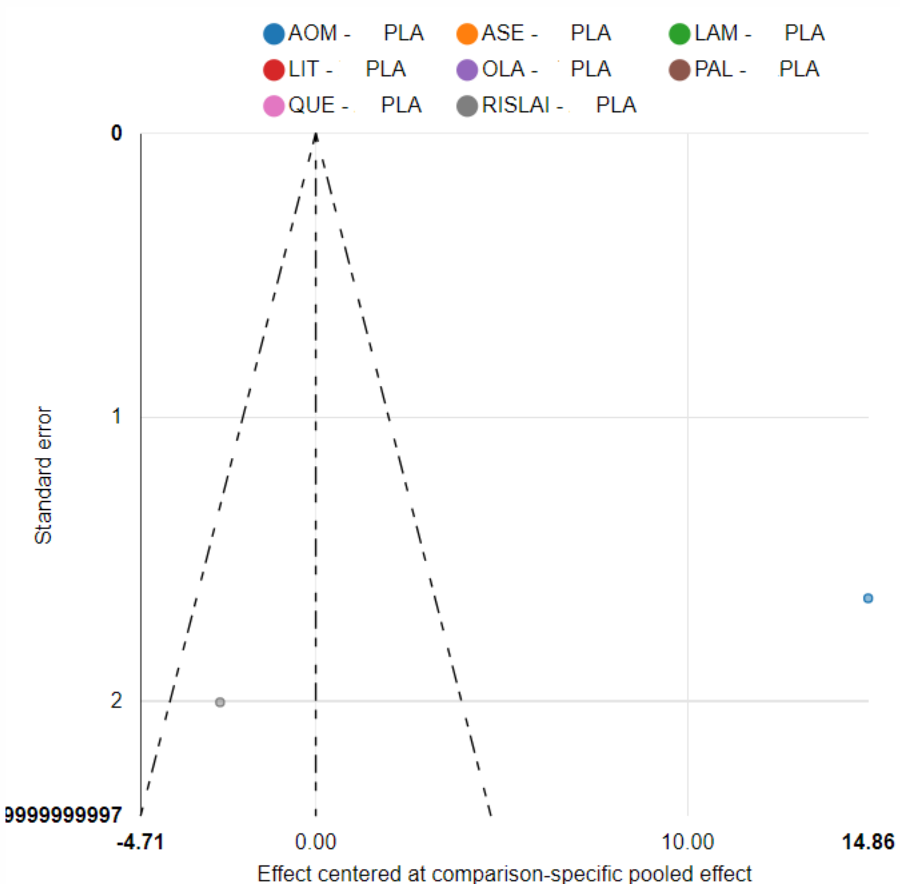

CINeMA rating

Comparison	Number of studies	Within-study bias	Reporting bias	Indirectness	Imprecision	Heterogeneity	Incoherence	Confidence rating
AOM:PLA	1	No concerns	Suspected	No concerns	Major concerns	No concerns	No concerns	Low
ARI+LAM:LAM	1	No concerns	Suspected	No concerns	Major concerns	No concerns	No concerns	Low
ASE:PLA	1	No concerns	Suspected	No concerns	Major concerns	No concerns	No concerns	Low
LAM:LIT	1	Some concerns	Suspected	No concerns	Major concerns	No concerns	No concerns	Very low
LAM:PLA	1	Some concerns	Suspected	No concerns	Major concerns	No concerns	No concerns	Very low
LIT:LIT+VAL	1	Major concerns	Suspected	No concerns	Major concerns	No concerns	No concerns	Very low
LIT:OLA	1	No concerns	Suspected	No concerns	Major concerns	No concerns	No concerns	Low
LIT:PLA	3	Some concerns	Suspected	No concerns	Major concerns	No concerns	No concerns	Very low
LIT:QUE	1	Some concerns	Suspected	No concerns	Major concerns	No concerns	No concerns	Very low
LIT:VAL	1	Major concerns	Suspected	No concerns	Major concerns	No concerns	No concerns	Very low
LIT+VAL:VAL	1	Major concerns	Suspected	No concerns	Major concerns	No concerns	No concerns	Very low
OLA:PLA	1	No concerns	Suspected	No concerns	Major concerns	No concerns	No concerns	Low
OLA:RISLAI	1	No concerns	Suspected	No concerns	Major concerns	No concerns	No concerns	Low
PAL:PLA	1	No concerns	Suspected	No concerns	Major concerns	No concerns	No concerns	Low
PLA:QUE	2	No concerns	Suspected	No concerns	Major concerns	No concerns	No concerns	Low
PLA:RISLAI	1	No concerns	Suspected	No concerns	Major concerns	No concerns	No concerns	Low
AOM:ARI+LAM	0	No concerns	Suspected	No concerns	Major concerns	No concerns	No concerns	Low
AOM:ASE	0	No concerns	Suspected	No concerns	Major concerns	No concerns	No concerns	Low
AOM:LAM	0	Some concerns	Suspected	No concerns	Major concerns	No concerns	No concerns	Very low
AOM:LIT	0	No concerns	Suspected	No concerns	Major concerns	No concerns	No concerns	Low
AOM:LIT+VAL	0	Major concerns	Suspected	No concerns	Major concerns	No concerns	No concerns	Very low
AOM:OLA	0	No concerns	Suspected	No concerns	Major concerns	No concerns	No concerns	Low
AOM:PAL	0	No concerns	Suspected	No concerns	Major concerns	No concerns	No concerns	Low
AOM:QUE	0	No concerns	Suspected	No concerns	Major concerns	No concerns	No concerns	Low
AOM:RISLAI	0	No concerns	Suspected	No concerns	Major concerns	No concerns	No concerns	Low
AOM:VAL	0	Major concerns	Suspected	No concerns	Major concerns	No concerns	No concerns	Very low
ARI+LAM:ASE	0	No concerns	Suspected	No concerns	Major concerns	No concerns	No concerns	Low
ARI+LAM:LIT	0	Some concerns	Suspected	No concerns	Major concerns	No concerns	No concerns	Very low
ARI+LAM:LIT+VAL	0	Major concerns	Suspected	No concerns	Major concerns	No concerns	No concerns	Very low
ARI+LAM:OLA	0	No concerns	Suspected	No concerns	Major concerns	No concerns	No concerns	Low
ARI+LAM:PAL	0	No concerns	Suspected	No concerns	Major concerns	No concerns	No concerns	Low
ARI+LAM:PLA	0	Some concerns	Suspected	No concerns	Major concerns	No concerns	No concerns	Very low

ARI+LAM:QUE	0	No concerns	Suspected	No concerns	Major concerns	No concerns	No concerns	Low
ARI+LAM:RISLAI	0	No concerns	Suspected	No concerns	Major concerns	No concerns	No concerns	Low
ARI+LAM:VAL	0	Major concerns	Suspected	No concerns	Major concerns	No concerns	No concerns	Very low
ASE:LAM	0	Some concerns	Suspected	No concerns	Major concerns	No concerns	No concerns	Very low
ASE:LIT	0	No concerns	Suspected	No concerns	Major concerns	No concerns	No concerns	Low
ASE:LIT+VAL	0	Major concerns	Suspected	No concerns	Major concerns	No concerns	No concerns	Very low
ASE:OLA	0	No concerns	Suspected	No concerns	Major concerns	No concerns	No concerns	Low
ASE:PAL	0	No concerns	Suspected	No concerns	Major concerns	No concerns	No concerns	Low
ASE:QUE	0	No concerns	Suspected	No concerns	Major concerns	No concerns	No concerns	Low
ASE:RISLAI	0	No concerns	Suspected	No concerns	Major concerns	No concerns	No concerns	Low
ASE:VAL	0	Major concerns	Suspected	No concerns	Major concerns	No concerns	No concerns	Very low
LAM:LIT+VAL	0	Major concerns	Suspected	No concerns	Major concerns	No concerns	No concerns	Very low
LAM:OLA	0	No concerns	Suspected	No concerns	Major concerns	No concerns	No concerns	Low
LAM:PAL	0	Some concerns	Suspected	No concerns	Major concerns	No concerns	No concerns	Very low
LAM:QUE	0	Some concerns	Suspected	No concerns	Major concerns	No concerns	No concerns	Very low
LAM:RISLAI	0	No concerns	Suspected	No concerns	Major concerns	No concerns	No concerns	Low
LAM:VAL	0	Major concerns	Suspected	No concerns	Major concerns	No concerns	No concerns	Very low
LIT:PAL	0	No concerns	Suspected	No concerns	Major concerns	No concerns	No concerns	Low
LIT:RISLAI	0	No concerns	Suspected	No concerns	Major concerns	No concerns	No concerns	Low
LIT+VAL:OLA	0	Major concerns	Suspected	No concerns	Major concerns	No concerns	No concerns	Very low
LIT+VAL:PAL	0	Major concerns	Suspected	No concerns	Major concerns	No concerns	No concerns	Very low
LIT+VAL:PLA	0	Major concerns	Suspected	No concerns	Major concerns	No concerns	No concerns	Very low
LIT+VAL:QUE	0	Major concerns	Suspected	No concerns	Major concerns	No concerns	No concerns	Very low
LIT+VAL:RISLAI	0	Major concerns	Suspected	No concerns	Major concerns	No concerns	No concerns	Very low
OLA:PAL	0	No concerns	Suspected	No concerns	Major concerns	No concerns	No concerns	Low
OLA:QUE	0	No concerns	Suspected	No concerns	Major concerns	No concerns	No concerns	Low
OLA:VAL	0	Major concerns	Suspected	No concerns	Major concerns	No concerns	No concerns	Very low
PAL:QUE	0	No concerns	Suspected	No concerns	Major concerns	No concerns	No concerns	Low
PAL:RISLAI	0	No concerns	Suspected	No concerns	Major concerns	No concerns	No concerns	Low
PAL:VAL	0	Major concerns	Suspected	No concerns	Major concerns	No concerns	No concerns	Very low
PLA:VAL	0	Major concerns	Suspected	No concerns	Major concerns	No concerns	No concerns	Very low
QUE:RISLAI	0	No concerns	Suspected	No concerns	Major concerns	No concerns	No concerns	Low
QUE:VAL	0	Major concerns	Suspected	No concerns	Major concerns	No concerns	No concerns	Very low
RISLAI:VAL	0	Major concerns	Suspected	No concerns	Major concerns	No concerns	No concerns	Very low

Raw data for mortality rate

	Intervention	n	Total patients	Intervention	n	Total patients	Intervention	n	Total patients
Amsterdam 2010	LIT			PLA					
Berwaerts 2012	PAL	2	152	PLA	0	148			
Bowden 2000	DIV			LIT			PLA		
Bowden 2003	LAM			LIT			PLA		
Bowden 2012	LAM+VAL			LAM					
Calabrese 2000	LAM			PLA					
Calabrese 2003	LAM	1	171	LIT	0	121	PLA	0	121
Calabrese 2005	DIV			LIT					
Calabrese 2017	AOM	1	132	PLA	0	133			
Carlson 2012	ARI+LAM	0	178	LAM	0	173			
Coxhead 1992	CAR			LIT					
Cundall 1972	LIT			PLA					
Dunner 1976	LIT			PLA					
Fieve 1976	LIT			PLA					
Geddes 2010	LIT	2	110	VAL	3	110	LIT+VAL	4	110
Hartong 2003	CAR			LIT					
Kane 1982	LIT			PLA					
Keck 2007	ARI			PLA					
Kleindienst 2000	CAR			LIT					
Koyama 2011	LAM			PLA					
Melia 1970	LIT			PLA					
Prien 1973a	LIT			PLA					
Prien 1973b	LIT	0	101	PLA	1	104			
Quiroz 2010	RIS-LAI			PLA					

	Intervention	n	Total patients	Intervention	n	Total patients	Intervention	n	Total patients
Szegedi 2018	ASE	0	126	PLA	0	127			
Tohen 2005	LIT	0	214	OLA	2	217			
Tohen 2006	OLA			PLA					
Vieta 2008	LIT+OXC			LIT					
Vieta 2012	OLA	0	131	RISLAI	0	132	PLA	0	135
Weisler 2011	LIT	0	364	QUE	0	404	PLA	0	404
Woo 2011	ARI+VAL			VAL					
Young 2014	QUE	1	291	PLA	1	294			

1.7. Completed suicide (25 RCTs, 6742 patients)

League table (odds ratio)

Results from pairwise meta-analysis are presented in the left lower half and results from network meta-analysis in the upper right half.

The boldface result indicates statistical significance.

AOM	0.947 (0.004, 245.452)	1.235 (0.004, 430.523)	1.000 (0.004, 258.243)	0.709 (0.007, 71.179)	1.200 (0.016, 92.333)	1.479 (0.024, 90.423)	1.607 (0.007, 362.745)	0.861 (0.010, 74.604)	0.343 (0.002, 54.615)	1.153 (0.010, 127.485)	0.974 (0.009, 110.686)	1.746 (0.018, 171.075)	1.008 (0.020, 51.152)
	ARI	1.304 (0.004, 455.958)	1.055 (0.004, 273.554)	0.749 (0.007, 75.452)	1.267 (0.016, 97.895)	1.562 (0.025, 95.899)	1.697 (0.007, 384.291)	0.909 (0.010, 79.091)	0.362 (0.002, 57.870)	1.218 (0.011, 135.125)	1.028 (0.009, 117.308)	1.843 (0.019, 181.345)	1.064 (0.021, 54.261)
		ARI+LAM	0.809 (0.002, 282.280)	0.574 (0.004, 75.573)	0.972 (0.019, 49.259)	1.198 (0.015, 98.978)	1.301 (0.005, 373.307)	0.697 (0.006, 84.184)	0.277 (0.001, 61.320)	0.934 (0.006, 143.079)	0.789 (0.005, 125.776)	1.414 (0.011, 185.082)	0.816 (0.011, 62.659)
			ASE	0.710 (0.007, 71.229)	1.201 (0.016, 92.398)	1.480 (0.024, 90.486)	1.608 (0.007, 362.963)	0.861 (0.010, 74.657)	0.343 (0.002, 54.647)	1.154 (0.010, 127.574)	0.975 (0.009, 110.753)	1.746 (0.018, 171.177)	1.008 (0.020, 51.188)
				CAR	1.692 (0.093, 30.695)	2.085 (0.261, 16.683)	2.266 (0.036, 141.189)	1.214 (0.060, 24.670)	0.483 (0.009, 26.736)	1.626 (0.053, 49.576)	1.373 (0.040, 46.726)	2.461 (0.122, 49.665)	1.420 (0.127, 15.836)
		0.972 (0.019, 49.259)			LAM	1.233 (0.164, 9.280)	1.339 (0.023, 78.878)	0.717 (0.046, 11.236)	0.285 (0.007, 11.610)	0.961 (0.041, 22.376)	0.812 (0.033, 20.136)	1.454 (0.081, 26.172)	0.839 (0.131, 5.358)
				2.085 (0.261, 16.683)	0.744 (0.046, 12.002)	LIT	1.086 (0.031, 38.621)	0.582 (0.066, 5.143)	0.232 (0.007, 7.174)	0.780 (0.052, 11.741)	0.658 (0.038, 11.371)	1.180 (0.135, 10.325)	0.681 (0.201, 2.310)
						1.000 (0.020, 50.846)	LIT+VAL	0.536 (0.008, 34.405)	0.213 (0.002, 29.309)	0.718 (0.008, 62.091)	0.606 (0.006, 56.753)	1.086 (0.031, 38.621)	0.627 (0.015, 26.243)

						0.336 (0.014, 8.305)		OLA	0.398 (0.009, 18.601)	1.340 (0.050, 36.107)	1.132 (0.057, 22.347)	2.028 (0.099, 41.421)	1.170 (0.141, 9.729)
									PAL	3.366 (0.054, 208.159)	2.843 (0.045, 181.417)	5.095 (0.095, 274.486)	2.941 (0.119, 72.762)
						1.110 (0.022, 56.070)				QUE	0.845 (0.021, 33.717)	1.514 (0.050, 45.847)	0.874 (0.065, 11.664)
								1.008 (0.020, 51.157)			RISLAI	1.792 (0.054, 59.704)	1.034 (0.074, 14.509)
						1.217 (0.125, 11.814)	1.000 (0.020, 50.846)					VAL	0.577 (0.054, 6.149)
1.008 (0.020, 51.152)	1.064 (0.021, 54.261)		1.008 (0.020, 51.188)		1.008 (0.141, 7.200)	0.737 (0.197, 2.764)		0.790 (0.049, 12.683)	2.941 (0.119, 72.762)	1.005 (0.063, 16.103)	0.993 (0.062, 15.959)	0.504 (0.010, 25.598)	PLA

Evaluation of heterogeneity and inconsistency

Between study variance (τ^2)	Heterogeneity assessment	Random-effects design-by-treatment interaction model		
		Q	df	p
0.01	Low	0.746	10	1.000

Inconsistency

	NMA, RR (95%CI)	Direct, RR (95%CI)	Indirect, RR (95%CI)	Inconsistency measures	
				Ratio of risk ratios	P value
LAM vs LIT	1.233 (0.164, 9.280)	0.744 (0.046, 12.002)	2.163 (0.115, 40.740)	0.344 (0.006, 19.613)	0.605
LAM vs PLA	0.839 (0.131, 5.358)	1.008 (0.141, 7.200)	0.195 (0.001, 50.496)	5.160 (0.014, 1870.386)	0.585
LIT vs LIT+VAL	1.086 (0.031, 38.621)	1.000 (0.020, 50.846)	1.610 (0.000, 8453.197)	0.621 (0.000, 7694.030)	0.921
LIT vs OLA	0.582 (0.066, 5.143)	0.336 (0.014, 8.305)	0.931 (0.048, 18.169)	0.361 (0.005, 28.580)	0.648
LIT vs QUE	0.780 (0.052, 11.741)	1.110 (0.022, 56.070)	0.564 (0.013, 24.085)	1.967 (0.009, 448.631)	0.807
LIT vs VAL	1.180 (0.135, 10.325)	1.217 (0.125, 11.814)	0.856 (0.001, 1236.080)	1.422 (0.001, 2902.420)	0.928
LIT vs PLA	0.681 (0.201, 2.310)	0.737 (0.197, 2.764)	0.428 (0.018, 10.463)	1.722 (0.054, 54.735)	0.758
LIT+VAL vs VAL	1.086 (0.031, 38.621)	1.000 (0.020, 50.846)	1.610 (0.000, 8453.197)	0.621 (0.000, 7694.030)	0.921
OLA vs RISLAI	1.132 (0.057, 22.347)	1.008 (0.020, 51.157)	1.326 (0.014, 130.061)	0.760 (0.002, 318.493)	0.929
OLA vs PLA	1.170 (0.141, 9.729)	0.790 (0.049, 12.683)	2.023 (0.076, 53.512)	0.390 (0.005, 28.586)	0.668
QUE vs PLA	0.874 (0.065, 11.664)	1.005 (0.063, 16.103)	0.333 (0.000, 479.191)	3.017 (0.001, 7232.210)	0.781
RISLAI vs PLA	1.034 (0.074, 14.509)	0.993 (0.062, 15.959)	1.519 (0.000, 7872.256)	0.654 (0.000, 5255.866)	0.926
VAL vs PLA	0.577 (0.054, 6.149)	0.504 (0.010, 25.598)	0.623 (0.032, 12.081)	0.808 (0.006, 110.819)	0.932

Rank probabilities

	Rank 1	Rank 2	Rank 3	Rank 4	Rank 5	Rank 6	Rank 7	Rank 8	Rank 9	Rank 10	Rank 11	Rank 12	Rank 13	Rank 14
AOM	0.085	0.078	0.087	0.090	0.096	0.088	0.066	0.067	0.060	0.065	0.073	0.062	0.050	0.032
ARI	0.101	0.098	0.078	0.080	0.074	0.073	0.073	0.065	0.061	0.053	0.062	0.061	0.065	0.057
ARI+LAM	0.095	0.115	0.080	0.068	0.065	0.062	0.062	0.046	0.045	0.038	0.046	0.060	0.079	0.141
ASE	0.113	0.074	0.062	0.062	0.056	0.058	0.074	0.072	0.057	0.054	0.054	0.067	0.103	0.095
CAR	0.000	0.005	0.013	0.020	0.025	0.038	0.046	0.061	0.087	0.105	0.120	0.129	0.151	0.200
LAM	0.109	0.158	0.140	0.099	0.105	0.097	0.071	0.061	0.035	0.024	0.032	0.032	0.024	0.012
LIT	0.021	0.060	0.104	0.140	0.178	0.192	0.134	0.105	0.053	0.012	0.001	0.000	0.000	0.000
LIT+VAL	0.172	0.133	0.123	0.125	0.088	0.070	0.057	0.046	0.039	0.035	0.032	0.033	0.035	0.013
OLA	0.000	0.003	0.009	0.028	0.052	0.074	0.113	0.125	0.135	0.135	0.118	0.105	0.070	0.034
PAL	0.000	0.000	0.000	0.000	0.000	0.001	0.004	0.009	0.022	0.053	0.108	0.183	0.255	0.365
QUE	0.130	0.112	0.093	0.071	0.073	0.050	0.067	0.085	0.064	0.054	0.054	0.064	0.058	0.025
RISLAI	0.083	0.071	0.103	0.096	0.076	0.079	0.071	0.060	0.078	0.068	0.072	0.076	0.049	0.019
VAL	0.091	0.095	0.107	0.122	0.110	0.103	0.130	0.090	0.051	0.035	0.028	0.022	0.012	0.006
PLA	0.000	0.000	0.000	0.000	0.001	0.016	0.033	0.110	0.213	0.271	0.201	0.105	0.048	0.002

SUCRA

LIT+VAL	0.84
VAL	0.83
QUE	0.75
RISLAI	0.74
LIT	0.70
PLA	0.55
ARI	0.43
AOM	0.40
ASE	0.36
OLA	0.36
LAM	0.30
CAR	0.30
ARI+LAM	0.22
PAL	0.22

Comparison-adjusted funnel plot of studies of drugs versus placebo

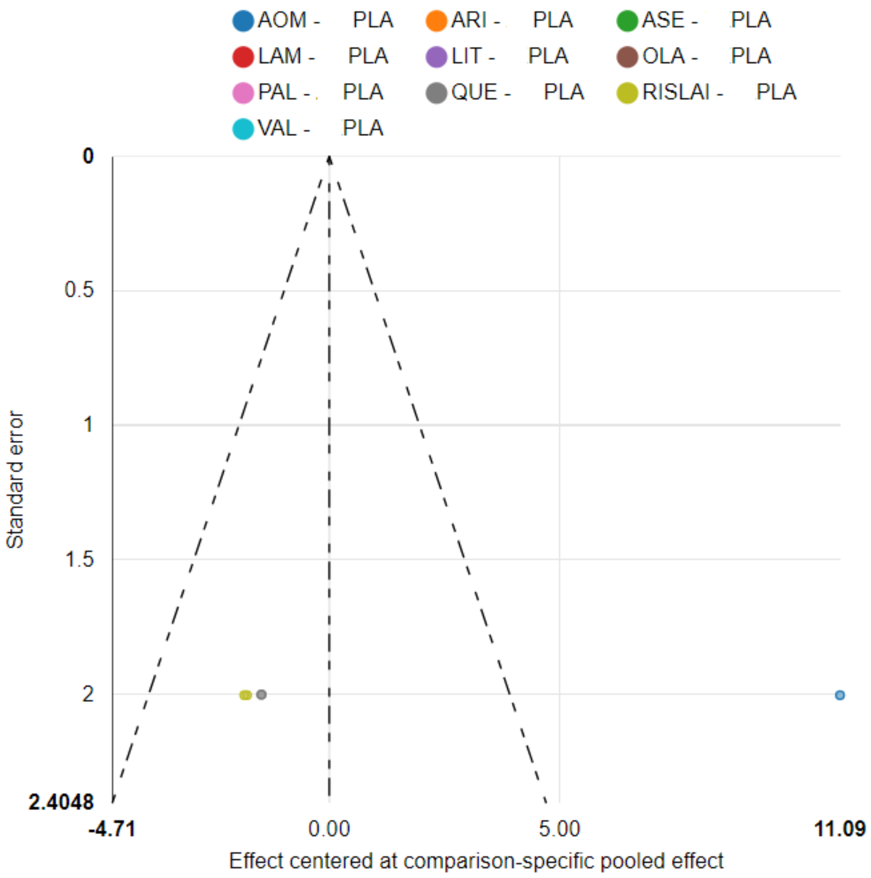

CINeMA rating

Comparison	Number of studies	Within-study bias	Reporting bias	Indirectness	Imprecision	Heterogeneity	Incoherence	Confidence rating
AOM:PLA	1	No concerns	Suspected	No concerns	Major concerns	No concerns	No concerns	Low
ARI:PLA	1	No concerns	Suspected	No concerns	Major concerns	No concerns	No concerns	Low
ARI+LAM:LAM	1	No concerns	Suspected	No concerns	Major concerns	No concerns	No concerns	Low
ASE:PLA	1	No concerns	Suspected	No concerns	Major concerns	No concerns	No concerns	Low
CAR:LIT	3	Some concerns	Suspected	No concerns	Major concerns	No concerns	No concerns	Very low
LAM:LIT	2	Some concerns	Suspected	No concerns	Major concerns	No concerns	No concerns	Very low
LAM:PLA	4	Some concerns	Suspected	No concerns	Major concerns	No concerns	No concerns	Very low
LIT:LIT+VAL	1	Major concerns	Suspected	No concerns	Major concerns	No concerns	No concerns	Very low
LIT:OLA	1	No concerns	Suspected	No concerns	Major concerns	No concerns	No concerns	Low
LIT:PLA	8	No concerns	Suspected	No concerns	Major concerns	No concerns	No concerns	Low
LIT:QUE	1	Some concerns	Suspected	No concerns	Major concerns	No concerns	No concerns	Very low
LIT:VAL	3	No concerns	Suspected	No concerns	Major concerns	No concerns	No concerns	Low
LIT+VAL:VAL	1	Major concerns	Suspected	No concerns	Major concerns	No concerns	No concerns	Very low
OLA:PLA	2	No concerns	Suspected	No concerns	Major concerns	No concerns	No concerns	Low
OLA:RISLAI	1	No concerns	Suspected	No concerns	Major concerns	No concerns	No concerns	Low
PAL:PLA	1	No concerns	Suspected	No concerns	Major concerns	No concerns	No concerns	Low
PLA:QUE	2	No concerns	Suspected	No concerns	Major concerns	No concerns	No concerns	Low
PLA:RISLAI	2	No concerns	Suspected	No concerns	Major concerns	No concerns	No concerns	Low
PLA:VAL	1	No concerns	Suspected	No concerns	Major concerns	No concerns	No concerns	Low
AOM:ARI	0	No concerns	Suspected	No concerns	Major concerns	No concerns	No concerns	Low
AOM:ARI+LAM	0	No concerns	Suspected	No concerns	Major concerns	No concerns	No concerns	Low
AOM:ASE	0	No concerns	Suspected	No concerns	Major concerns	No concerns	No concerns	Low
AOM:CAR	0	No concerns	Suspected	No concerns	Major concerns	No concerns	No concerns	Low
AOM:LAM	0	No concerns	Suspected	No concerns	Major concerns	No concerns	No concerns	Low
AOM:LIT	0	No concerns	Suspected	No concerns	Major concerns	No concerns	No concerns	Low
AOM:LIT+VAL	0	No concerns	Suspected	No concerns	Major concerns	No concerns	No concerns	Low
AOM:OLA	0	No concerns	Suspected	No concerns	Major concerns	No concerns	No concerns	Low
AOM:PAL	0	No concerns	Suspected	No concerns	Major concerns	No concerns	No concerns	Low
AOM:QUE	0	No concerns	Suspected	No concerns	Major concerns	No concerns	No concerns	Low
AOM:RISLAI	0	No concerns	Suspected	No concerns	Major concerns	No concerns	No concerns	Low
AOM:VAL	0	No concerns	Suspected	No concerns	Major concerns	No concerns	No concerns	Low
ARI:ARI+LAM	0	No concerns	Suspected	No concerns	Major concerns	No concerns	No concerns	Low

ARI:ASE	0	No concerns	Suspected	No concerns	Major concerns	No concerns	No concerns	Low
ARI:CAR	0	No concerns	Suspected	No concerns	Major concerns	No concerns	No concerns	Low
ARI:LAM	0	No concerns	Suspected	No concerns	Major concerns	No concerns	No concerns	Low
ARI:LIT	0	No concerns	Suspected	No concerns	Major concerns	No concerns	No concerns	Low
ARI:LIT+VAL	0	No concerns	Suspected	No concerns	Major concerns	No concerns	No concerns	Low
ARI:OLA	0	No concerns	Suspected	No concerns	Major concerns	No concerns	No concerns	Low
ARI:PAL	0	No concerns	Suspected	No concerns	Major concerns	No concerns	No concerns	Low
ARI:QUE	0	No concerns	Suspected	No concerns	Major concerns	No concerns	No concerns	Low
ARI:RISLAI	0	No concerns	Suspected	No concerns	Major concerns	No concerns	No concerns	Low
ARI:VAL	0	No concerns	Suspected	No concerns	Major concerns	No concerns	No concerns	Low
ARI+LAM:ASE	0	No concerns	Suspected	No concerns	Major concerns	No concerns	No concerns	Low
ARI+LAM:CAR	0	Some concerns	Suspected	No concerns	Major concerns	No concerns	No concerns	Very low
ARI+LAM:LIT	0	No concerns	Suspected	No concerns	Major concerns	No concerns	No concerns	Low
ARI+LAM:LIT+VAL	0	No concerns	Suspected	No concerns	Major concerns	No concerns	No concerns	Low
ARI+LAM:OLA	0	No concerns	Suspected	No concerns	Major concerns	No concerns	No concerns	Low
ARI+LAM:PAL	0	No concerns	Suspected	No concerns	Major concerns	No concerns	No concerns	Low
ARI+LAM:PLA	0	No concerns	Suspected	No concerns	Major concerns	No concerns	No concerns	Low
ARI+LAM:QUE	0	Some concerns	Suspected	No concerns	Major concerns	No concerns	No concerns	Very low
ARI+LAM:RISLAI	0	No concerns	Suspected	No concerns	Major concerns	No concerns	No concerns	Low
ARI+LAM:VAL	0	No concerns	Suspected	No concerns	Major concerns	No concerns	No concerns	Low
ASE:CAR	0	No concerns	Suspected	No concerns	Major concerns	No concerns	No concerns	Low
ASE:LAM	0	No concerns	Suspected	No concerns	Major concerns	No concerns	No concerns	Low
ASE:LIT	0	No concerns	Suspected	No concerns	Major concerns	No concerns	No concerns	Low
ASE:LIT+VAL	0	No concerns	Suspected	No concerns	Major concerns	No concerns	No concerns	Low
ASE:OLA	0	No concerns	Suspected	No concerns	Major concerns	No concerns	No concerns	Low
ASE:PAL	0	No concerns	Suspected	No concerns	Major concerns	No concerns	No concerns	Low
ASE:QUE	0	No concerns	Suspected	No concerns	Major concerns	No concerns	No concerns	Low
ASE:RISLAI	0	No concerns	Suspected	No concerns	Major concerns	No concerns	No concerns	Low
ASE:VAL	0	No concerns	Suspected	No concerns	Major concerns	No concerns	No concerns	Low
CAR:LAM	0	Some concerns	Suspected	No concerns	Major concerns	No concerns	No concerns	Very low
CAR:LIT+VAL	0	Major concerns	Suspected	No concerns	Major concerns	No concerns	No concerns	Very low
CAR:OLA	0	No concerns	Suspected	No concerns	Major concerns	No concerns	No concerns	Low
CAR:PAL	0	No concerns	Suspected	No concerns	Major concerns	No concerns	No concerns	Low

CAR:PLA	0	Some concerns	Suspected	No concerns	Major concerns	No concerns	No concerns	Very low
CAR:QUE	0	Some concerns	Suspected	No concerns	Major concerns	No concerns	No concerns	Very low
CAR:RISLAI	0	No concerns	Suspected	No concerns	Major concerns	No concerns	No concerns	Low
CAR:VAL	0	No concerns	Suspected	No concerns	Major concerns	No concerns	No concerns	Low
LAM:LIT+VAL	0	Major concerns	Suspected	No concerns	Major concerns	No concerns	No concerns	Very low
LAM:OLA	0	No concerns	Suspected	No concerns	Major concerns	No concerns	No concerns	Low
LAM:PAL	0	No concerns	Suspected	No concerns	Major concerns	No concerns	No concerns	Low
LAM:QUE	0	Some concerns	Suspected	No concerns	Major concerns	No concerns	No concerns	Very low
LAM:RISLAI	0	No concerns	Suspected	No concerns	Major concerns	No concerns	No concerns	Low
LAM:VAL	0	Some concerns	Suspected	No concerns	Major concerns	No concerns	No concerns	Very low
LIT:PAL	0	No concerns	Suspected	No concerns	Major concerns	No concerns	No concerns	Low
LIT:RISLAI	0	No concerns	Suspected	No concerns	Major concerns	No concerns	No concerns	Low
LIT+VAL:OLA	0	No concerns	Suspected	No concerns	Major concerns	No concerns	No concerns	Low
LIT+VAL:PAL	0	No concerns	Suspected	No concerns	Major concerns	No concerns	No concerns	Low
LIT+VAL:PLA	0	Major concerns	Suspected	No concerns	Major concerns	No concerns	No concerns	Very low
LIT+VAL:QUE	0	Major concerns	Suspected	No concerns	Major concerns	No concerns	No concerns	Very low
LIT+VAL:RISLAI	0	No concerns	Suspected	No concerns	Major concerns	No concerns	No concerns	Low
OLA:PAL	0	No concerns	Suspected	No concerns	Major concerns	No concerns	No concerns	Low
OLA:QUE	0	No concerns	Suspected	No concerns	Major concerns	No concerns	No concerns	Low
OLA:VAL	0	No concerns	Suspected	No concerns	Major concerns	No concerns	No concerns	Low
PAL:QUE	0	No concerns	Suspected	No concerns	Major concerns	No concerns	No concerns	Low
PAL:RISLAI	0	No concerns	Suspected	No concerns	Major concerns	No concerns	No concerns	Low
PAL:VAL	0	No concerns	Suspected	No concerns	Major concerns	No concerns	No concerns	Low
QUE:RISLAI	0	No concerns	Suspected	No concerns	Major concerns	No concerns	No concerns	Low
QUE:VAL	0	No concerns	Suspected	No concerns	Major concerns	No concerns	No concerns	Low
RISLAI:VAL	0	No concerns	Suspected	No concerns	Major concerns	No concerns	No concerns	Low

Raw data for completed suicide

	Intervention	n	Total patients	Intervention	n	Total patients	Intervention	n	Total patients
Amsterdam 2010	LIT	0	26	PLA	0	27			
Berwaerts 2012	PAL	1	152	PLA	0	148			
Bowden 2000	DIV	0	187	LIT	0	91	PLA	0	94
Bowden 2003	LAM	0	59	LIT	0	46	PLA	0	70
Bowden 2012	LAM+VAL			LAM					
Calabrese 2000	LAM	0	93	PLA	0	89			
Calabrese 2003	LAM	0	171	LIT	0	121	PLA	0	121
Calabrese 2005	DIV	0	28	LIT	0	32			
Calabrese 2017	AOM	0	132	PLA	0	133			
Carlson 2012	ARI+LAM	0	178	LAM	0	173			
Coxhead 1992	CAR	2	15	LIT	0	16			
Cundall 1972	LIT			PLA					
Dunner 1976	LIT			PLA					
Fieve 1976	LIT			PLA					
Geddes 2010	LIT	0	110	VAL	0	110	LIT+VAL	0	110
Hartong 2003	CAR	0	30	LIT	0	23			
Kane 1982	LIT	0	4	PLA	0	7			
Keck 2007	ARI	0	78	PLA	0	83			
Kleindienst 2000	CAR	0	85	LIT	0	86			
Koyama 2011	LAM	0	45	PLA	0	58			
Melia 1970	LIT			PLA					
Prien 1973a	LIT	0	18	PLA	1	13			
Prien 1973b	LIT	0	101	PLA	1	104			
Quiroz 2010	RIS-LAI	0	140	PLA	0	135			
Szegedi 2018	ASE	0	126	PLA	0	127			

	Intervention	n	Total patients	Intervention	n	Total patients	Intervention	n	Total patients
Tohen 2005	LIT	0	214	OLA	1	217			
Tohen 2006	OLA	0	225	PLA	0	136			
Vieta 2008	LIT+OXC			LIT					
Vieta 2012	OLA	0	131	RISLAI	0	132	PLA	0	135
Weisler 2011	LIT	0	364	QUE	0	404	PLA	0	404
Woo 2011	ARI+VAL			VAL					
Young 2014	QUE	0	291	PLA	0	294			

1.8. Extrapyramidal symptoms/use of anticholinergic agent (14 studies, 4778 patients)

League table

Results from pairwise meta-analysis are presented in the left lower half and results from network meta-analysis in the upper right half.

The boldface result indicates statistical significance.

AOM	0.217 (0.024, 1.924)	0.877 (0.251, 3.061)	0.248 (0.020, 3.070)	0.545 (0.052, 5.730)	3.498 (0.674, 18.149)	1.534 (0.540, 4.362)	0.491 (0.235, 1.029)	1.624 (0.487, 5.414)	0.829 (0.069, 9.963)	1.357 (0.565, 3.258)	1.018 (0.212, 4.892)	0.533 (0.241, 1.180)	1.636 (0.908, 2.949)
	ARI	4.043 (0.377, 43.402)	1.143 (0.045, 28.766)	2.515 (0.113, 55.757)	**16.130 (1.193, 218.045)**	7.075 (0.729, 68.621)	2.266 (0.264, 19.424)	7.490 (0.715, 78.492)	3.824 (0.156, 93.972)	6.257 (0.694, 56.441)	4.695 (0.364, 60.497)	2.458 (0.281, 21.497)	7.545 (0.922, 61.726)
		ARI+LAM	0.283 (0.020, 3.969)	0.622 (0.050, 7.810)	3.990 (0.652, 24.413)	1.750 (0.881, 3.477)	0.560 (0.195, 1.612)	1.853 (0.404, 8.494)	0.946 (0.066, 13.458)	1.548 (0.459, 5.214)	1.161 (0.187, 7.207)	0.608 (0.196, 1.883)	1.867 (0.620, 5.623)
			ARI+VAL	2.200 (0.078, 62.203)	14.107 (0.826, 240.832)	6.187 (0.483, 79.297)	1.982 (0.175, 22.421)	6.551 (0.457, 93.860)	3.344 (0.107, 104.074)	5.472 (0.450, 66.593)	4.106 (0.238, 70.718)	2.150 (0.198, 23.406)	6.600 (0.572, 76.195)
				ASE	6.413 (0.411, 100.053)	2.813 (0.246, 32.111)	0.901 (0.089, 9.169)	2.978 (0.243, 36.547)	1.520 (0.055, 42.026)	2.488 (0.233, 26.544)	1.867 (0.125, 27.830)	0.977 (0.094, 10.132)	3.000 (0.308, 29.239)
					CAR	0.439 (0.082, 2.344)	**0.140 (0.032, 0.612)**	0.464 (0.072, 2.988)	0.237 (0.014, 4.153)	0.388 (0.078, 1.927)	0.291 (0.035, 2.417)	**0.152 (0.033, 0.706)**	0.468 (0.101, 2.176)
		1.750 (0.881, 3.477)				LAM	**0.320 (0.143, 0.715)**	1.059 (0.272, 4.121)	0.541 (0.042, 7.027)	0.884 (0.325, 2.409)	0.664 (0.122, 3.602)	**0.347 (0.142, 0.853)**	1.067 (0.450, 2.528)
					0.140 (0.032, 0.612)	0.320 (0.139, 0.732)	LIT	**3.306 (1.056, 10.345)**	1.688 (0.145, 19.680)	**2.762 (1.462, 5.217)**	2.072 (0.452, 9.490)	1.085 (0.705, 1.670)	**3.330 (2.132, 5.203)**

								OLA	0.511 (0.037, 7.109)	0.835 (0.243, 2.869)	0.627 (0.104, 3.770)	0.328 (0.101, 1.066)	1.007 (0.353, 2.879)
									PAL	1.636 (0.134, 19.949)	1.228 (0.073, 20.588)	0.643 (0.054, 7.626)	1.973 (0.176, 22.085)
							**2.497 (1.190, 5.237)**			QUE	0.750 (0.153, 3.690)	**0.393 (0.188, 0.822)**	1.206 (0.631, 2.306)
											RISLAI	0.524 (0.111, 2.466)	1.607 (0.375, 6.884)
			2.150 (0.198, 23.406)				1.087 (0.693, 1.705)					VAL	**3.069 (1.801, 5.233)**
1.636 (0.908, 2.949)	7.545 (0.922, 61.726)			3.000 (0.308, 29.239)		1.074 (0.370, 3.118)	**3.378 (2.129, 5.360)**	1.007 (0.353, 2.879)	1.973 (0.176, 22.085)	1.196 (0.577, 2.478)	1.607 (0.375, 6.884)	3**.226 (1.672, 6.223)**	PLA

Evaluation of heterogeneity and inconsistency

Between study variance (τ^2)	Heterogeneity assessment	Random-effects design-by-treatment interaction model		
		Q	df	p
0.032	Low to moderate	4.660	4	0.324

Inconsistency

	NMA, RR (95%CI)	Direct, RR (95%CI)	Indirect, RR (95%CI)	Inconsistency measures	
				Ratio of risk ratios	P value
LAM vs LIT	0.320 (0.143, 0.715)	0.320 (0.139, 0.732)	0.331 (0.013, 8.273)	0.964 (0.035, 26.722)	0.983
LAM vs PLA	1.067 (0.450, 2.528)	1.074 (0.370, 3.118)	1.053 (0.242, 4.581)	1.020 (0.166, 6.274)	0.983
LIT vs QUE	2.762 (1.462, 5.217)	2.497 (1.190, 5.237)	3.666 (1.059, 12.694)	0.681 (0.160, 2.892)	0.603
LIT vs VAL	1.085 (0.705, 1.670)	1.087 (0.693, 1.705)	1.061 (0.235, 4.787)	1.025 (0.213, 4.940)	0.976
LIT vs PLA	3.330 (2.132, 5.203)	3.378 (2.129, 5.360)	2.719 (0.478, 15.461)	1.242 (0.206, 7.503)	0.813
QUE vs PLA	1.206 (0.631, 2.306)	1.196 (0.577, 2.478)	1.246 (0.301, 5.159)	0.959 (0.194, 4.735)	0.959
VAL vs PLA	3.069 (1.801, 5.233)	3.226 (1.672, 6.223)	2.789 (1.119, 6.950)	1.156 (0.375, 3.562)	0.800

Rank probabilities

	Rank 1	Rank 2	Rank 3	Rank 4	Rank 5	Rank 6	Rank 7	Rank 8	Rank 9	Rank 10	Rank 11	Rank 12	Rank 13	Rank 14
AOM	0.024	0.043	0.049	0.060	0.071	0.088	0.112	0.141	0.128	0.093	0.075	0.059	0.039	0.018
ARI	0.008	0.010	0.016	0.018	0.019	0.023	0.035	0.044	0.059	0.070	0.080	0.115	0.261	0.243
ARI+LAM	0.036	0.047	0.054	0.059	0.052	0.059	0.085	0.098	0.105	0.098	0.093	0.084	0.079	0.052
ARI+VAL	0.013	0.017	0.017	0.016	0.014	0.016	0.019	0.027	0.032	0.039	0.050	0.075	0.154	0.512
ASE	0.088	0.080	0.069	0.056	0.055	0.058	0.064	0.073	0.069	0.072	0.074	0.089	0.095	0.058
CAR	0.315	0.177	0.108	0.070	0.059	0.058	0.051	0.045	0.033	0.029	0.020	0.018	0.013	0.003
LAM	0.061	0.126	0.137	0.112	0.114	0.109	0.099	0.083	0.064	0.044	0.027	0.014	0.010	0.001
LIT	0.000	0.000	0.000	0.001	0.003	0.005	0.014	0.031	0.074	0.158	0.236	0.279	0.167	0.032
OLA	0.127	0.152	0.130	0.101	0.087	0.083	0.072	0.064	0.054	0.044	0.032	0.025	0.021	0.009
PAL	0.202	0.118	0.076	0.064	0.053	0.056	0.056	0.055	0.061	0.054	0.048	0.057	0.059	0.042
QUE	0.033	0.083	0.112	0.135	0.129	0.130	0.125	0.100	0.067	0.042	0.024	0.014	0.005	0.002
RISLAI	0.083	0.096	0.097	0.077	0.067	0.071	0.075	0.074	0.080	0.069	0.069	0.061	0.058	0.024
VAL	0.007	0.014	0.022	0.033	0.042	0.051	0.075	0.105	0.147	0.182	0.171	0.110	0.039	0.004
PLA	0.005	0.037	0.114	0.199	0.236	0.194	0.119	0.061	0.026	0.007	0.002	0.001	0.000	0.000

SUCRA

CAR	0.86
PLA	0.74
OLA	0.68
LAM	0.67
QUE	0.63
RISLAI	0.54
AOM	0.52
ARI_LAM	0.46
PAL	0.46
VAL	0.40
ASE	0.36
ARI_VAL	0.26
LIT	0.25
ARI	0.16

Comparison-adjusted funnel plot of studies of drugs versus placebo

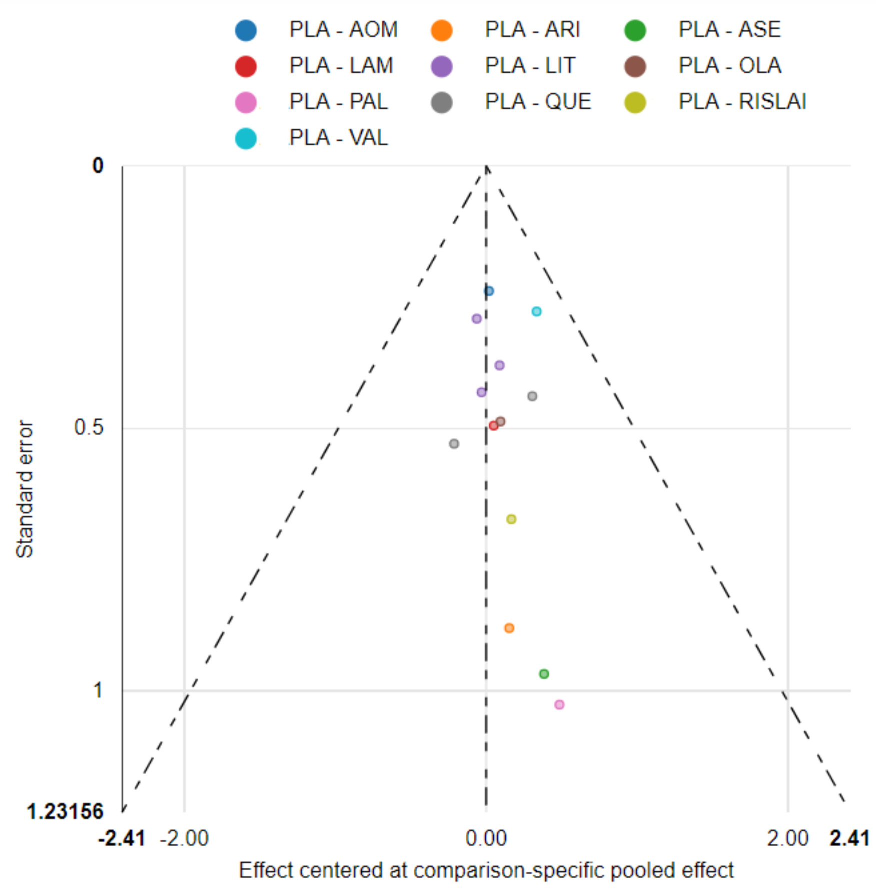

CINeMA rating

Comparison	Number of studies	Within-study bias	Reporting bias	Indirectness	Imprecision	Heterogeneity	Incoherence	Confidence rating
AOM:PLA	1	No concerns	Suspected	No concerns	Major concerns	No concerns	No concerns	Low
ARI:PLA	1	No concerns	Suspected	No concerns	Major concerns	No concerns	No concerns	Low
ARI+LAM:LAM	1	No concerns	Suspected	No concerns	Major concerns	No concerns	No concerns	Low
ARI+VAL:VAL	1	Some concerns	Suspected	No concerns	Major concerns	No concerns	No concerns	Very low
ASE:PLA	1	No concerns	Suspected	No concerns	Major concerns	No concerns	No concerns	Low
CAR:LIT	1	No concerns	Suspected	No concerns	No concerns	Major concerns	No concerns	Low
LAM:LIT	1	Some concerns	Suspected	No concerns	No concerns	Major concerns	No concerns	Very low
LAM:PLA	1	Some concerns	Suspected	No concerns	Major concerns	No concerns	No concerns	Very low
LIT:PLA	3	Some concerns	Suspected	No concerns	No concerns	No concerns	No concerns	Low
LIT:QUE	1	Some concerns	Suspected	No concerns	No concerns	Major concerns	No concerns	Very low
LIT:VAL	2	No concerns	Suspected	No concerns	Major concerns	No concerns	No concerns	Low
OLA:PLA	1	No concerns	Suspected	No concerns	Major concerns	No concerns	No concerns	Low
PAL:PLA	1	No concerns	Suspected	No concerns	Major concerns	No concerns	No concerns	Low
PLA:QUE	2	Some concerns	Suspected	No concerns	Major concerns	No concerns	No concerns	Very low
PLA:RISLAI	1	No concerns	Suspected	No concerns	Major concerns	No concerns	No concerns	Low
PLA:VAL	1	No concerns	Suspected	No concerns	No concerns	No concerns	No concerns	Moderate
AOM:ARI	0	No concerns	Suspected	No concerns	Major concerns	No concerns	No concerns	Low
AOM:ARI+LAM	0	No concerns	Suspected	No concerns	Major concerns	No concerns	No concerns	Low
AOM:ARI+VAL	0	No concerns	Suspected	No concerns	Major concerns	No concerns	No concerns	Low
AOM:ASE	0	No concerns	Suspected	No concerns	Major concerns	No concerns	No concerns	Low
AOM:CAR	0	No concerns	Suspected	No concerns	Major concerns	No concerns	No concerns	Low
AOM:LAM	0	No concerns	Suspected	No concerns	Major concerns	No concerns	No concerns	Low
AOM:LIT	0	No concerns	Suspected	No concerns	Major concerns	No concerns	No concerns	Low
AOM:OLA	0	No concerns	Suspected	No concerns	Major concerns	No concerns	No concerns	Low
AOM:PAL	0	No concerns	Suspected	No concerns	Major concerns	No concerns	No concerns	Low
AOM:QUE	0	No concerns	Suspected	No concerns	Major concerns	No concerns	No concerns	Low
AOM:RISLAI	0	No concerns	Suspected	No concerns	Major concerns	No concerns	No concerns	Low
AOM:VAL	0	No concerns	Suspected	No concerns	Major concerns	No concerns	No concerns	Low
ARI:ARI+LAM	0	No concerns	Suspected	No concerns	Major concerns	No concerns	No concerns	Low
ARI:ARI+VAL	0	No concerns	Suspected	No concerns	Major concerns	No concerns	No concerns	Low
ARI:ASE	0	No concerns	Suspected	No concerns	Major concerns	No concerns	No concerns	Low
ARI:CAR	0	No concerns	Suspected	No concerns	No concerns	Major concerns	No concerns	Low

ARI:LAM	0	No concerns	Suspected	No concerns	Major concerns	No concerns	No concerns	Low
ARI:LIT	0	No concerns	Suspected	No concerns	Major concerns	No concerns	No concerns	Low
ARI:OLA	0	No concerns	Suspected	No concerns	Major concerns	No concerns	No concerns	Low
ARI:PAL	0	No concerns	Suspected	No concerns	Major concerns	No concerns	No concerns	Low
ARI:QUE	0	No concerns	Suspected	No concerns	Major concerns	No concerns	No concerns	Low
ARI:RISLAI	0	No concerns	Suspected	No concerns	Major concerns	No concerns	No concerns	Low
ARI:VAL	0	No concerns	Suspected	No concerns	Major concerns	No concerns	No concerns	Low
ARI+LAM:ARI+VAL	0	Some concerns	Suspected	No concerns	Major concerns	No concerns	No concerns	Very low
ARI+LAM:ASE	0	No concerns	Suspected	No concerns	Major concerns	No concerns	No concerns	Low
ARI+LAM:CAR	0	No concerns	Suspected	No concerns	Major concerns	No concerns	No concerns	Low
ARI+LAM:LIT	0	No concerns	Suspected	No concerns	Major concerns	No concerns	No concerns	Low
ARI+LAM:OLA	0	No concerns	Suspected	No concerns	Major concerns	No concerns	No concerns	Low
ARI+LAM:PAL	0	No concerns	Suspected	No concerns	Major concerns	No concerns	No concerns	Low
ARI+LAM:PLA	0	No concerns	Suspected	No concerns	Major concerns	No concerns	No concerns	Low
ARI+LAM:QUE	0	Some concerns	Suspected	No concerns	Major concerns	No concerns	No concerns	Very low
ARI+LAM:RISLAI	0	No concerns	Suspected	No concerns	Major concerns	No concerns	No concerns	Low
ARI+LAM:VAL	0	No concerns	Suspected	No concerns	Major concerns	No concerns	No concerns	Low
ARI+VAL:ASE	0	No concerns	Suspected	No concerns	Major concerns	No concerns	No concerns	Low
ARI+VAL:CAR	0	No concerns	Suspected	No concerns	Major concerns	No concerns	No concerns	Low
ARI+VAL:LAM	0	Some concerns	Suspected	No concerns	Major concerns	No concerns	No concerns	Very low
ARI+VAL:LIT	0	Some concerns	Suspected	No concerns	Major concerns	No concerns	No concerns	Very low
ARI+VAL:OLA	0	No concerns	Suspected	No concerns	Major concerns	No concerns	No concerns	Low
ARI+VAL:PAL	0	No concerns	Suspected	No concerns	Major concerns	No concerns	No concerns	Low
ARI+VAL:PLA	0	Some concerns	Suspected	No concerns	Major concerns	No concerns	No concerns	Very low
ARI+VAL:QUE	0	Some concerns	Suspected	No concerns	Major concerns	No concerns	No concerns	Very low
ARI+VAL:RISLAI	0	No concerns	Suspected	No concerns	Major concerns	No concerns	No concerns	Low
ASE:CAR	0	No concerns	Suspected	No concerns	Major concerns	No concerns	No concerns	Low
ASE:LAM	0	No concerns	Suspected	No concerns	Major concerns	No concerns	No concerns	Low
ASE:LIT	0	No concerns	Suspected	No concerns	Major concerns	No concerns	No concerns	Low
ASE:OLA	0	No concerns	Suspected	No concerns	Major concerns	No concerns	No concerns	Low
ASE:PAL	0	No concerns	Suspected	No concerns	Major concerns	No concerns	No concerns	Low
ASE:QUE	0	No concerns	Suspected	No concerns	Major concerns	No concerns	No concerns	Low
ASE:RISLAI	0	No concerns	Suspected	No concerns	Major concerns	No concerns	No concerns	Low

ASE:VAL	0	No concerns	Suspected	No concerns	Major concerns	No concerns	No concerns	Low
CAR:LAM	0	No concerns	Suspected	No concerns	Major concerns	No concerns	No concerns	Low
CAR:OLA	0	No concerns	Suspected	No concerns	Major concerns	No concerns	No concerns	Low
CAR:PAL	0	No concerns	Suspected	No concerns	Major concerns	No concerns	No concerns	Low
CAR:PLA	0	No concerns	Suspected	No concerns	Major concerns	No concerns	No concerns	Low
CAR:QUE	0	No concerns	Suspected	No concerns	Major concerns	No concerns	No concerns	Low
CAR:RISLAI	0	No concerns	Suspected	No concerns	Major concerns	No concerns	No concerns	Low
CAR:VAL	0	No concerns	Suspected	No concerns	No concerns	Major concerns	No concerns	Low
LAM:OLA	0	No concerns	Suspected	No concerns	Major concerns	No concerns	No concerns	Low
LAM:PAL	0	No concerns	Suspected	No concerns	Major concerns	No concerns	No concerns	Low
LAM:QUE	0	Some concerns	Suspected	No concerns	Major concerns	No concerns	No concerns	Very low
LAM:RISLAI	0	No concerns	Suspected	No concerns	Major concerns	No concerns	No concerns	Low
LAM:VAL	0	Some concerns	Suspected	No concerns	No concerns	Major concerns	No concerns	Very low
LIT:OLA	0	No concerns	Suspected	No concerns	No concerns	Major concerns	No concerns	Low
LIT:PAL	0	No concerns	Suspected	No concerns	Major concerns	No concerns	No concerns	Low
LIT:RISLAI	0	No concerns	Suspected	No concerns	Major concerns	No concerns	No concerns	Low
OLA:PAL	0	No concerns	Suspected	No concerns	Major concerns	No concerns	No concerns	Low
OLA:QUE	0	No concerns	Suspected	No concerns	Major concerns	No concerns	No concerns	Low
OLA:RISLAI	0	No concerns	Suspected	No concerns	Major concerns	No concerns	No concerns	Low
OLA:VAL	0	No concerns	Suspected	No concerns	Major concerns	No concerns	No concerns	Low
PAL:QUE	0	No concerns	Suspected	No concerns	Major concerns	No concerns	No concerns	Low
PAL:RISLAI	0	No concerns	Suspected	No concerns	Major concerns	No concerns	No concerns	Low
PAL:VAL	0	No concerns	Suspected	No concerns	Major concerns	No concerns	No concerns	Low
QUE:RISLAI	0	No concerns	Suspected	No concerns	Major concerns	No concerns	No concerns	Low
QUE:VAL	0	No concerns	Suspected	No concerns	No concerns	Major concerns	No concerns	Low
RISLAI:VAL	0	No concerns	Suspected	No concerns	Major concerns	No concerns	No concerns	Low

1.9. Somnolence (11 studies, 4036 patients)

League table

Results from pairwise meta-analysis are presented in the left lower half and results from network meta-analysis in the upper right half.

The boldface result indicates statistical significance.

ASE	1.117 (0.063, 19.864)	0.992 (0.059, 16.819)	0.278 (0.015, 5.291)	0.203 (0.006, 7.101)	0.515 (0.029, 9.221)	0.542 (0.026, 11.171)	0.754 (0.044, 13.055)	1.000 (0.061, 16.489)
	LAM	0.889 (0.474, 1.668)	**0.249 (0.082, 0.758)**	0.182 (0.018, 1.784)	0.461 (0.186, 1.141)	0.485 (0.131, 1.799)	0.675 (0.308, 1.481)	0.896 (0.465, 1.726)
	0.691 (0.351, 1.359)	LIT	**0.280 (0.105, 0.743)**	0.204 (0.022, 1.888)	0.519 (0.257, 1.047)	0.546 (0.165, 1.806)	0.760 (0.446, 1.294)	1.008 (0.680, 1.492)
		0.078 (0.004, 1.433)	OLA	0.730 (0.068, 7.811)	1.853 (0.596, 5.760)	1.951 (0.770, 4.944)	2.714 (0.956, 7.710)	**3.600 (1.448, 8.946)**
				PAL	2.540 (0.256, 25.174)	2.674 (0.227, 31.545)	3.720 (0.392, 35.322)	4.933 (0.553, 44.027)
		**0.394 (0.170, 0.912)**			QUE	1.053 (0.280, 3.965)	1.465 (0.641, 3.347)	1.942 (0.979, 3.854)
			2.015 (0.782, 5.191)			RISLAI	1.391 (0.398, 4.856)	1.845 (0.590, 5.766)
		0.654 (0.359, 1.188)					VAL	1.326 (0.785, 2.241)
1.000 (0.061, 16.489)	1.338 (0.620, 2.887)	1.021 (0.686, 1.521)	**3.134 (1.203, 8.166)**	4.933 (0.553, 44.027)	1.588 (0.740, 3.407)	2.045 (0.574, 7.294)	1.188 (0.665, 2.124)	PLA

Evaluation of heterogeneity and inconsistency

Between study variance (τ^2)	Heterogeneity assessment	Random-effects design-by-treatment interaction model		
		Q	df	p
0.061	Low to moderate	9.609	6	0.142

Inconsistency

	NMA, RR (95%CI)	Direct, RR (95%CI)	Indirect, RR (95%CI)	Inconsistency measures	
				Ratio of risk ratios	P value
**LAM vs LIT**	**0.889 (0.474, 1.668)**	**0.691 (0.351, 1.359)**	**4.540 (0.812, 25.378)**	**0.152 (0.024, 0.967)**	**0.046**
LAM vs PLA	0.896 (0.465, 1.726)	1.338 (0.620, 2.887)	0.305 (0.087, 1.076)	4.382 (1.002, 19.165)	0.050
LIT vs OLA	0.280 (0.105, 0.743)	0.078 (0.004, 1.433)	0.329 (0.117, 0.928)	0.237 (0.011, 5.205)	0.361
LIT vs QUE	0.519 (0.257, 1.047)	0.394 (0.170, 0.912)	0.985 (0.274, 3.544)	0.400 (0.086, 1.850)	0.241
LIT vs VAL	0.760 (0.446, 1.294)	0.654 (0.359, 1.188)	1.357 (0.420, 4.382)	0.482 (0.129, 1.797)	0.277
LIT vs PLA	1.008 (0.680, 1.492)	1.021 (0.686, 1.521)	0.608 (0.054, 6.850)	1.680 (0.144, 19.545)	0.679
OLA vs RISLAI	1.951 (0.770, 4.944)	2.015 (0.782, 5.191)	0.793 (0.005, 117.378)	2.541 (0.016, 410.921)	0.719
OLA vs PLA	3.600 (1.448, 8.946)	3.134 (1.203, 8.166)	13.228 (0.701, 249.560)	0.237 (0.011, 5.205)	0.361
QUE vs PLA	1.942 (0.979, 3.854)	1.588 (0.740, 3.407)	4.478 (0.945, 21.211)	0.355 (0.063, 2.006)	0.241
RISLAI vs PLA	1.845 (0.590, 5.766)	2.045 (0.574, 7.294)	1.209 (0.093, 15.805)	1.692 (0.096, 29.767)	0.719
VAL vs PLA	1.326 (0.785, 2.241)	1.188 (0.665, 2.124)	2.151 (0.636, 7.282)	0.552 (0.143, 2.132)	0.389

Rank probabilities

	Rank 1	Rank 2	Rank 3	Rank 4	Rank 5	Rank 6	Rank 7	Rank 8	Rank 9
ASE	0.625	0.072	0.037	0.033	0.046	0.049	0.044	0.045	0.050
LAM	0.158	0.319	0.169	0.131	0.101	0.074	0.033	0.012	0.003
LIT	0.051	0.204	0.278	0.231	0.146	0.070	0.020	0.002	0.000
OLA	0.001	0.002	0.005	0.008	0.020	0.063	0.168	0.389	0.344
PAL	0.038	0.054	0.038	0.036	0.047	0.075	0.129	0.151	0.433
QUE	0.010	0.021	0.028	0.039	0.091	0.205	0.279	0.216	0.111
RISLAI	0.058	0.107	0.074	0.077	0.119	0.175	0.201	0.142	0.048
VAL	0.036	0.091	0.100	0.139	0.245	0.225	0.112	0.042	0.010
PLA	0.023	0.131	0.273	0.307	0.186	0.065	0.014	0.002	0.000

SUCRA

LAM	0.76
PLA	0.74
LIT	0.73
ASE	0.63
VAL	0.54
RISLAI	0.40
QUE	0.40
PAL	0.16
OLA	0.13

Comparison-adjusted funnel plot of studies of drugs versus placebo

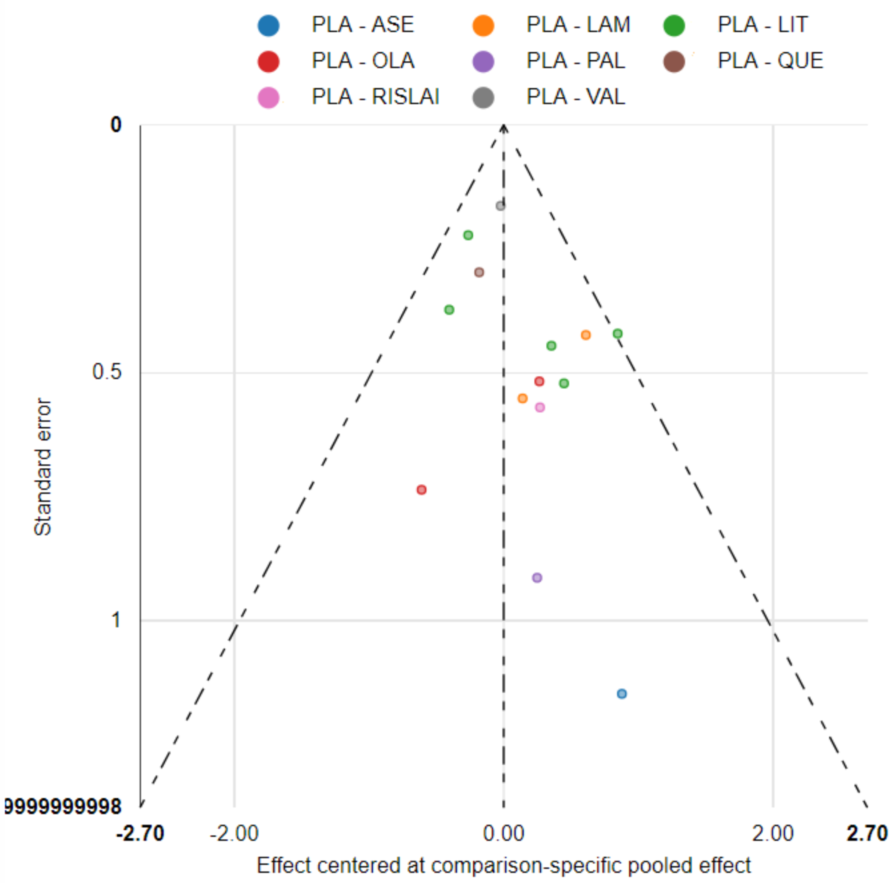

CINeMA rating

Comparison	Number of studies	Within-study bias	Reporting bias	Indirectness	Imprecision	Heterogeneity	Incoherence	Confidence rating
ASE:PLA	1	No concerns	Suspected	No concerns	Major concerns	No concerns	No concerns	Low
LAM:LIT	2	Some concerns	Suspected	No concerns	Major concerns	No concerns	No concerns	Very low
LAM:PLA	2	Some concerns	Suspected	No concerns	Major concerns	No concerns	No concerns	Very low
LIT:OLA	1	No concerns	Suspected	No concerns	No concerns	Major concerns	No concerns	Low
LIT:PLA	5	No concerns	Suspected	No concerns	Major concerns	No concerns	No concerns	Low
LIT:QUE	1	Some concerns	Suspected	No concerns	Major concerns	No concerns	No concerns	Very low
LIT:VAL	2	No concerns	Suspected	No concerns	Major concerns	No concerns	No concerns	Low
OLA:PLA	2	No concerns	Suspected	No concerns	No concerns	No concerns	No concerns	Moderate
OLA:RISLAI	1	No concerns	Suspected	No concerns	Major concerns	No concerns	No concerns	Low
PAL:PLA	1	No concerns	Suspected	No concerns	Major concerns	No concerns	No concerns	Low
PLA:QUE	1	Some concerns	Suspected	No concerns	Major concerns	No concerns	No concerns	Very low
PLA:RISLAI	1	No concerns	Suspected	No concerns	Major concerns	No concerns	No concerns	Low
PLA:VAL	1	No concerns	Suspected	No concerns	Major concerns	No concerns	No concerns	Low
ASE:LAM	0	No concerns	Suspected	No concerns	Major concerns	No concerns	No concerns	Low
ASE:LIT	0	No concerns	Suspected	No concerns	Major concerns	No concerns	No concerns	Low
ASE:OLA	0	No concerns	Suspected	No concerns	Major concerns	No concerns	No concerns	Low
ASE:PAL	0	No concerns	Suspected	No concerns	Major concerns	No concerns	No concerns	Low
ASE:QUE	0	No concerns	Suspected	No concerns	Major concerns	No concerns	No concerns	Low
ASE:RISLAI	0	No concerns	Suspected	No concerns	Major concerns	No concerns	No concerns	Low
ASE:VAL	0	No concerns	Suspected	No concerns	Major concerns	No concerns	No concerns	Low
LAM:OLA	0	No concerns	Suspected	No concerns	No concerns	Major concerns	No concerns	Low
LAM:PAL	0	No concerns	Suspected	No concerns	Major concerns	No concerns	No concerns	Low
LAM:QUE	0	Some concerns	Suspected	No concerns	Major concerns	No concerns	No concerns	Very low
LAM:RISLAI	0	No concerns	Suspected	No concerns	Major concerns	No concerns	No concerns	Low
LAM:VAL	0	Some concerns	Suspected	No concerns	Major concerns	No concerns	No concerns	Very low
LIT:PAL	0	No concerns	Suspected	No concerns	Major concerns	No concerns	No concerns	Low
LIT:RISLAI	0	No concerns	Suspected	No concerns	Major concerns	No concerns	No concerns	Low
OLA:PAL	0	No concerns	Suspected	No concerns	Major concerns	No concerns	No concerns	Low
OLA:QUE	0	No concerns	Suspected	No concerns	Major concerns	No concerns	No concerns	Low
OLA:VAL	0	No concerns	Suspected	No concerns	Major concerns	No concerns	No concerns	Low
PAL:QUE	0	No concerns	Suspected	No concerns	Major concerns	No concerns	No concerns	Low
PAL:RISLAI	0	No concerns	Suspected	No concerns	Major concerns	No concerns	No concerns	Low

PAL:VAL	0	No concerns	Suspected	No concerns	Major concerns	No concerns	No concerns	Low
QUE:RISLAI	0	No concerns	Suspected	No concerns	Major concerns	No concerns	No concerns	Low
QUE:VAL	0	Some concerns	Suspected	No concerns	Major concerns	No concerns	No concerns	Very low
RISLAI:VAL	0	No concerns	Suspected	No concerns	Major concerns	No concerns	No concerns	Low

1.10. Anxiety/use of anxiolytic (11 studies, 3247 patients

League table

Results from pairwise meta-analysis are presented in the left lower half and results from network meta-analysis in the upper right half.

The boldface result indicates statistical significance.

AOM	0.996 (0.359, 2.767)	0.628 (0.170, 2.318)	1.734 (0.259, 11.598)	1.276 (0.566, 2.878)	1.290 (0.597, 2.790)	1.668 (0.837, 3.325)	0.891 (0.370, 2.147)	1.974 (0.878, 4.437)	1.156 (0.658, 2.033)
	ARI	0.631 (0.147, 2.697)	1.741 (0.235, 12.925)	1.281 (0.455, 3.603)	1.296 (0.476, 3.525)	1.675 (0.654, 4.287)	0.894 (0.301, 2.652)	1.982 (0.706, 5.558)	1.161 (0.495, 2.722)
		ARI+LAM	2.761 (0.317, 24.013)	2.031 (0.732, 5.640)	2.054 (0.638, 6.620)	2.656 (0.783, 9.005)	1.418 (0.365, 5.510)	3.142 (0.849, 11.634)	1.841 (0.567, 5.974)
			ASE	0.736 (0.109, 4.952)	0.744 (0.113, 4.920)	0.962 (0.150, 6.163)	0.514 (0.074, 3.560)	1.138 (0.169, 7.649)	0.667 (0.109, 4.092)
		2.031 (0.732, 5.640)		LAM	1.011 (0.571, 1.791)	1.308 (0.670, 2.554)	0.698 (0.285, 1.707)	1.547 (0.682, 3.509)	0.906 (0.504, 1.628)
				0.891 (0.492, 1.614)	LIT	1.293 (0.726, 2.304)	0.690 (0.293, 1.624)	1.529 (0.708, 3.305)	0.896 (0.530, 1.515)
					0.845 (0.342, 2.091)	OLA	0.534 (0.244, 1.168)	1.183 (0.613, 2.281)	0.693 (0.466, 1.031)
							PAL	2.216 (0.909, 5.402)	1.298 (0.661, 2.550)
						0.756 (0.302, 1.890)		RISLAI	0.586 (0.328, 1.048)
1.156 (0.658, 2.033)	1.161 (0.495, 2.722)		0.667 (0.109, 4.092)	1.046 (0.566, 1.934)	1.092 (0.589, 2.024)	**0.638 (0.416, 0.979)**	1.298 (0.661, 2.550)	**0.527 (0.289, 0.959)**	PLA

Evaluation of heterogeneity and inconsistency

Between study variance (τ^2)	Heterogeneity assessment	Random-effects design-by-treatment interaction model		
		Q	df	p
0.040	Low to moderate	5.164	4	0.271

Inconsistency

	NMA, RR (95%CI)	Direct, RR (95%CI)	Indirect, RR (95%CI)	Inconsistency measures	
				Ratio of risk ratios	P value
LAM vs LIT	1.011 (0.571, 1.791)	0.891 (0.492, 1.614)	4.739 (0.595, 37.743)	0.188 (0.022, 1.627)	0.129
LAM vs PLA	0.906 (0.504, 1.628)	1.046 (0.566, 1.934)	0.216 (0.031, 1.507)	4.850 (0.631, 37.263)	0.129
LIT vs OLA	1.293 (0.726, 2.304)	0.845 (0.342, 2.091)	1.730 (0.817, 3.662)	0.488 (0.151, 1.583)	0.232
LIT vs PLA	0.896 (0.530, 1.515)	1.092 (0.589, 2.024)	0.533 (0.196, 1.451)	2.047 (0.632, 6.637)	0.232
OLA vs RISLAI	1.183 (0.613, 2.281)	0.756 (0.302, 1.890)	1.896 (0.740, 4.858)	0.399 (0.107, 1.482)	0.170
OLA vs PLA	0.693 (0.466, 1.031)	0.638 (0.416, 0.979)	1.141 (0.398, 3.269)	0.559 (0.179, 1.741)	0.316
RISLAI vs PLA	0.586 (0.328, 1.048)	0.527 (0.289, 0.959)	3.198 (0.290, 35.220)	0.165 (0.014, 1.953)	0.153

Rank probabilities

	Rank 1	Rank 2	Rank 3	Rank 4	Rank 5	Rank 6	Rank 7	Rank 8	Rank 9	Rank 10
AOM	0.026	0.043	0.054	0.071	0.081	0.103	0.135	0.178	0.178	0.131
ARI	0.050	0.068	0.071	0.079	0.089	0.088	0.109	0.137	0.175	0.134
ARI+LAM	0.030	0.038	0.032	0.036	0.040	0.049	0.057	0.085	0.164	0.468
ASE	0.428	0.105	0.075	0.065	0.054	0.049	0.045	0.054	0.064	0.062
LAM	0.060	0.103	0.122	0.147	0.147	0.121	0.110	0.109	0.070	0.010
LIT	0.070	0.117	0.170	0.190	0.160	0.113	0.083	0.060	0.032	0.006
OLA	0.091	0.224	0.259	0.174	0.107	0.069	0.040	0.024	0.009	0.003
PAL	0.026	0.037	0.044	0.061	0.074	0.089	0.111	0.161	0.227	0.171
RISLAI	0.219	0.264	0.160	0.116	0.082	0.063	0.040	0.028	0.022	0.007
PLA	0.000	0.002	0.014	0.063	0.166	0.255	0.270	0.165	0.059	0.008

SUCRA

RISLAI	0.77
OLA	0.75
LIT	0.62
ASE	0.62
LAM	0.56
PLA	0.42
ARI	0.37
AOM	0.34
PAL	0.30
ARI+LAM	0.24

Comparison-adjusted funnel plot of studies of drugs versus placebo

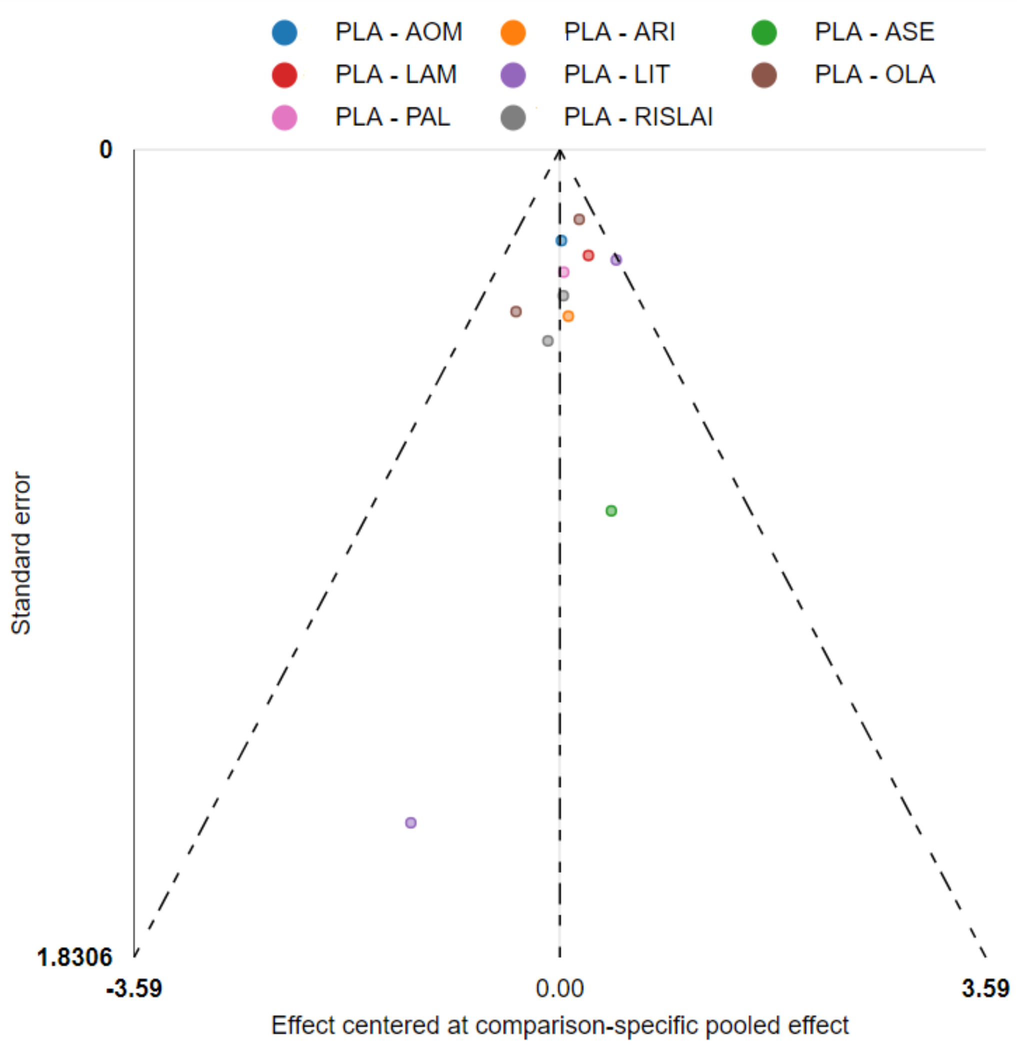

CINeMA rating

Comparison	Number of studies	Within-study bias	Reporting bias	Indirectness	Imprecision	Heterogeneity	Incoherence	Confidence rating
AOM:PLA	1	No concerns	Suspected	No concerns	Major concerns	No concerns	No concerns	Low
ARI:PLA	1	No concerns	Suspected	No concerns	Major concerns	No concerns	No concerns	Low
ARI+LAM:LAM	1	No concerns	Suspected	No concerns	Major concerns	No concerns	No concerns	Low
ASE:PLA	1	No concerns	Suspected	No concerns	Major concerns	No concerns	No concerns	Low
LAM:LIT	1	Some concerns	Suspected	No concerns	Major concerns	No concerns	No concerns	Very low
LAM:PLA	1	Some concerns	Suspected	No concerns	Major concerns	No concerns	No concerns	Very low
LIT:OLA	1	No concerns	Suspected	No concerns	Major concerns	No concerns	No concerns	Low
LIT:PLA	2	Some concerns	Suspected	No concerns	Major concerns	No concerns	No concerns	Very low
OLA:PLA	2	No concerns	Suspected	No concerns	Major concerns	No concerns	No concerns	Low
OLA:RISLAI	1	No concerns	Suspected	No concerns	Major concerns	No concerns	No concerns	Low
PAL:PLA	1	No concerns	Suspected	No concerns	Major concerns	No concerns	No concerns	Low
PLA:RISLAI	2	No concerns	Suspected	No concerns	Major concerns	No concerns	No concerns	Low
AOM:ARI	0	No concerns	Suspected	No concerns	Major concerns	No concerns	No concerns	Low
AOM:ARI+LAM	0	No concerns	Suspected	No concerns	Major concerns	No concerns	No concerns	Low
AOM:ASE	0	No concerns	Suspected	No concerns	Major concerns	No concerns	No concerns	Low
AOM:LAM	0	Some concerns	Suspected	No concerns	Major concerns	No concerns	No concerns	Very low
AOM:LIT	0	No concerns	Suspected	No concerns	Major concerns	No concerns	No concerns	Low
AOM:OLA	0	No concerns	Suspected	No concerns	Major concerns	No concerns	No concerns	Low
AOM:PAL	0	No concerns	Suspected	No concerns	Major concerns	No concerns	No concerns	Low
AOM:RISLAI	0	No concerns	Suspected	No concerns	Major concerns	No concerns	No concerns	Low
ARI:ARI+LAM	0	No concerns	Suspected	No concerns	Major concerns	No concerns	No concerns	Low
ARI:ASE	0	No concerns	Suspected	No concerns	Major concerns	No concerns	No concerns	Low
ARI:LAM	0	Some concerns	Suspected	No concerns	Major concerns	No concerns	No concerns	Very low
ARI:LIT	0	No concerns	Suspected	No concerns	Major concerns	No concerns	No concerns	Low
ARI:OLA	0	No concerns	Suspected	No concerns	Major concerns	No concerns	No concerns	Low
ARI:PAL	0	No concerns	Suspected	No concerns	Major concerns	No concerns	No concerns	Low
ARI:RISLAI	0	No concerns	Suspected	No concerns	Major concerns	No concerns	No concerns	Low
ARI+LAM:ASE	0	No concerns	Suspected	No concerns	Major concerns	No concerns	No concerns	Low
ARI+LAM:LIT	0	Some concerns	Suspected	No concerns	Major concerns	No concerns	No concerns	Very low
ARI+LAM:OLA	0	No concerns	Suspected	No concerns	Major concerns	No concerns	No concerns	Low
ARI+LAM:PAL	0	No concerns	Suspected	No concerns	Major concerns	No concerns	No concerns	Low
ARI+LAM:PLA	0	Some concerns	Suspected	No concerns	Major concerns	No concerns	No concerns	Very low

ARI+LAM:RISLAI	0	No concerns	Suspected	No concerns	Major concerns	No concerns	No concerns	Low
ASE:LAM	0	Some concerns	Suspected	No concerns	Major concerns	No concerns	No concerns	Very low
ASE:LIT	0	No concerns	Suspected	No concerns	Major concerns	No concerns	No concerns	Low
ASE:OLA	0	No concerns	Suspected	No concerns	Major concerns	No concerns	No concerns	Low
ASE:PAL	0	No concerns	Suspected	No concerns	Major concerns	No concerns	No concerns	Low
ASE:RISLAI	0	No concerns	Suspected	No concerns	Major concerns	No concerns	No concerns	Low
LAM:OLA	0	Some concerns	Suspected	No concerns	Major concerns	No concerns	No concerns	Very low
LAM:PAL	0	Some concerns	Suspected	No concerns	Major concerns	No concerns	No concerns	Very low
LAM:RISLAI	0	No concerns	Suspected	No concerns	Major concerns	No concerns	No concerns	Low
LIT:PAL	0	No concerns	Suspected	No concerns	Major concerns	No concerns	No concerns	Low
LIT:RISLAI	0	No concerns	Suspected	No concerns	Major concerns	No concerns	No concerns	Low
OLA:PAL	0	No concerns	Suspected	No concerns	Major concerns	No concerns	No concerns	Low
PAL:RISLAI	0	No concerns	Suspected	No concerns	Major concerns	No concerns	No concerns	Low

1.11. Insomnia (12 studies, 4793 patients)

League table

Results from pairwise meta-analysis are presented in the left lower half and results from network meta-analysis in the upper right half.

The boldface result indicates statistical significance.

AOM	1.486 (0.296, 7.456)	3.000 (0.396, 22.746)	0.953 (0.258, 3.515)	1.140 (0.353, 3.678)	3.042 (0.895, 10.344)	1.092 (0.254, 4.684)	2.814 (0.824, 9.607)	1.333 (0.349, 5.082)	1.000 (0.341, 2.932)
	ARI+LAM	2.019 (0.248, 16.417)	0.641 (0.249, 1.656)	0.767 (0.233, 2.522)	2.048 (0.560, 7.492)	0.735 (0.156, 3.469)	1.894 (0.511, 7.024)	0.897 (0.215, 3.737)	0.673 (0.202, 2.239)
		ASE	0.318 (0.049, 2.059)	0.380 (0.064, 2.249)	1.014 (0.165, 6.216)	0.364 (0.050, 2.629)	0.938 (0.153, 5.765)	0.444 (0.067, 2.948)	0.333 (0.060, 1.855)
	0.641 (0.249, 1.656)		LAM	1.196 (0.583, 2.455)	**3.192 (1.317, 7.736)**	1.145 (0.335, 3.915)	**2.952 (1.194, 7.299)**	1.398 (0.481, 4.062)	1.049 (0.501, 2.197)
			0.908 (0.414, 1.992)	LIT	**2.669 (1.447, 4.920)**	0.958 (0.323, 2.837)	**2.468 (1.282, 4.752)**	1.169 (0.485, 2.817)	0.877 (0.552, 1.395)
				**2.863 (1.228, 6.677)**	OLA	0.359 (0.114, 1.125)	0.925 (0.417, 2.050)	0.438 (0.191, 1.005)	**0.329 (0.183, 0.589)**
						PAL	2.578 (0.819, 8.116)	1.221 (0.345, 4.324)	0.916 (0.343, 2.446)
				1.933 (0.863, 4.328)			QUE	0.474 (0.178, 1.263)	**0.355 (0.197, 0.643)**
					0.595 (0.236, 1.504)			RISLAI	0.750 (0.338, 1.665)
1.000 (0.341, 2.932)		0.333 (0.060, 1.855)	1.500 (0.644, 3.496)	0.887 (0.526, 1.493)	**0.346 (0.168, 0.713)**	0.916 (0.343, 2.446)	**0.329 (0.177, 0.610)**	0.938 (0.400, 2.195)	PLA

Evaluation of heterogeneity and inconsistency

Between study variance (τ^2)	Heterogeneity assessment	Random-effects design-by-treatment interaction model		
		Q	df	p
0.116	Moderate to high	12.238	5	**0.032**

Inconsistency

	NMA, RR (95%CI)	Direct, RR (95%CI)	Indirect, RR (95%CI)	Inconsistency measures	
				Ratio of risk ratios	P value
LAM vs LIT	1.196 (0.583, 2.455)	0.908 (0.414, 1.992)	4.963 (0.833, 29.559)	0.183 (0.026, 1.285)	0.088
LAM vs PLA	1.049 (0.501, 2.197)	1.500 (0.644, 3.496)	0.333 (0.073, 1.515)	4.512 (0.795, 25.613)	0.089
LIT vs OLA	2.669 (1.447, 4.920)	2.863 (1.228, 6.677)	2.471 (1.020, 5.988)	1.158 (0.340, 3.943)	0.814
LIT vs QUE	2.468 (1.282, 4.752)	1.933 (0.863, 4.328)	3.974 (1.290, 12.244)	0.486 (0.122, 1.941)	0.307
LIT vs PLA	0.877 (0.552, 1.395)	0.887 (0.526, 1.493)	0.842 (0.305, 2.329)	1.052 (0.336, 3.299)	0.930
OLA vs RISLAI	0.438 (0.191, 1.005)	0.595 (0.236, 1.504)	0.126 (0.019, 0.814)	4.742 (0.589, 38.180)	0.144
OLA vs PLA	0.329 (0.183, 0.589)	0.346 (0.168, 0.713)	0.299 (0.111, 0.803)	1.158 (0.340, 3.943)	0.814
QUE vs PLA	0.355 (0.197, 0.643)	0.329 (0.177, 0.610)	0.881 (0.107, 7.287)	0.373 (0.041, 3.370)	0.380
RISLAI vs PLA	0.750 (0.338, 1.665)	0.938 (0.400, 2.195)	0.153 (0.016, 1.488)	6.110 (0.540, 69.110)	0.144

Rank probabilities

	Rank 1	Rank 2	Rank 3	Rank 4	Rank 5	Rank 6	Rank 7	Rank 8	Rank 9	Rank 10
AOM	0.021	0.031	0.053	0.103	0.115	0.101	0.100	0.095	0.119	0.263
ARI+LAM	0.051	0.067	0.109	0.184	0.136	0.105	0.080	0.070	0.095	0.104
ASE	0.480	0.127	0.128	0.085	0.050	0.034	0.027	0.021	0.020	0.028
LAM	0.001	0.005	0.012	0.037	0.090	0.131	0.149	0.171	0.217	0.188
LIT	0.000	0.002	0.018	0.088	0.200	0.243	0.214	0.141	0.067	0.026
OLA	0.265	0.388	0.234	0.078	0.023	0.007	0.003	0.001	0.001	0.000
PAL	0.015	0.032	0.057	0.121	0.131	0.118	0.101	0.107	0.118	0.202
QUE	0.155	0.322	0.316	0.129	0.045	0.016	0.008	0.006	0.003	0.001
RISLAI	0.012	0.026	0.073	0.170	0.182	0.149	0.118	0.095	0.096	0.078
PLA	0.000	0.000	0.001	0.005	0.029	0.097	0.200	0.295	0.265	0.109

SUCRA

OLA	0.87
QUE	0.81
ASE	0.77
ARI+LAM	0.51
RISLAI	0.47
LIT	0.39
PAL	0.36
AOM	0.31
PLA	0.27
LAM	0.25

Comparison-adjusted funnel plot of studies of drugs versus placebo

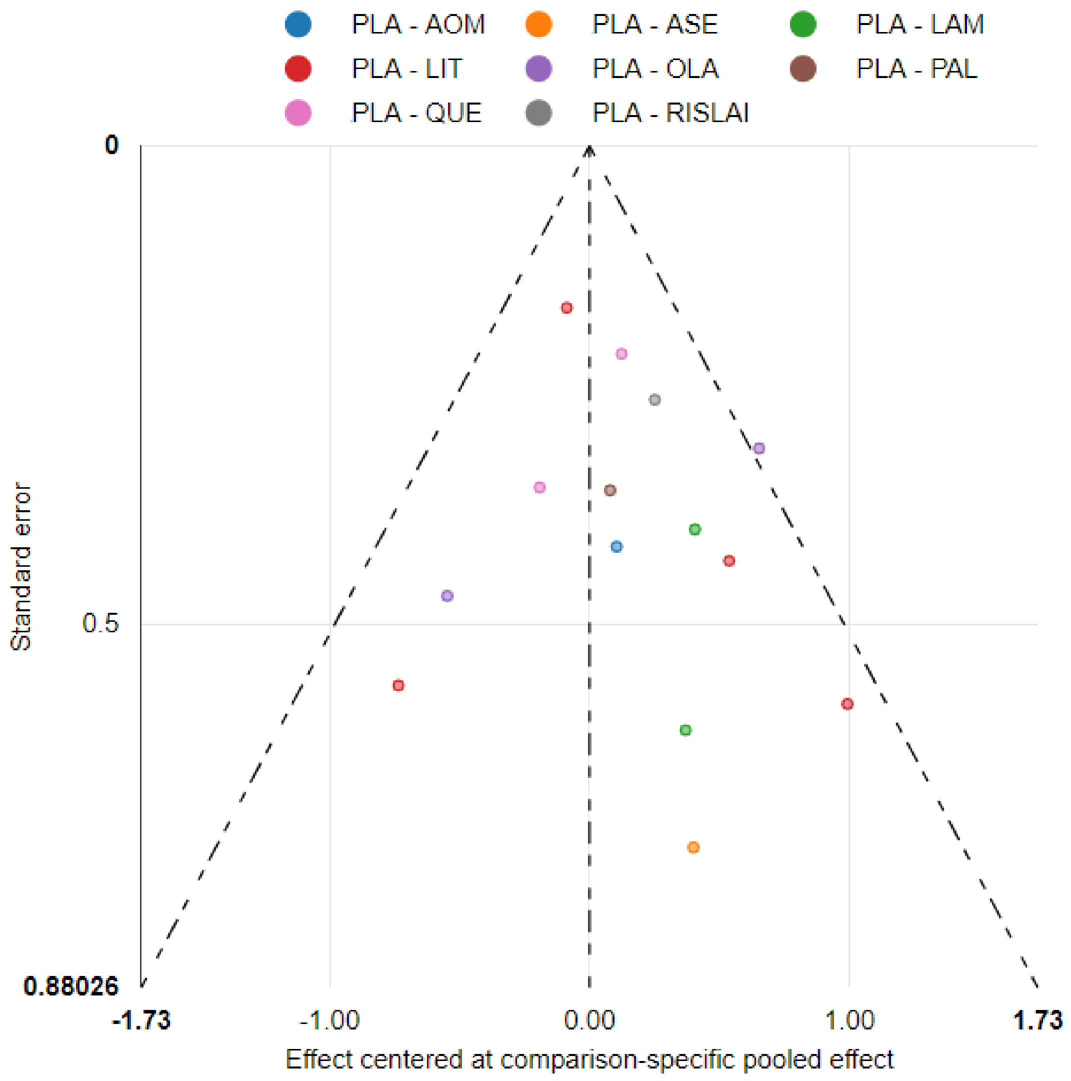

CINeMA rating

Comparison	Number of studies	Within-study bias	Reporting bias	Indirectness	Imprecision	Heterogeneity	Incoherence	Confidence rating
AOM:PLA	1	No concerns	Suspected	No concerns	Major concerns	No concerns	Major concerns	Very low
ARI+LAM:LAM	1	No concerns	Suspected	No concerns	Major concerns	No concerns	Major concerns	Very low
ASE:PLA	1	No concerns	Suspected	No concerns	Major concerns	No concerns	Major concerns	Very low
LAM:LIT	2	Some concerns	Suspected	No concerns	Major concerns	No concerns	No concerns	Very low
LAM:PLA	2	Some concerns	Suspected	No concerns	Major concerns	No concerns	No concerns	Very low
LIT:OLA	1	No concerns	Suspected	No concerns	No concerns	Major concerns	No concerns	Low
LIT:PLA	4	Some concerns	Suspected	No concerns	Major concerns	No concerns	No concerns	Very low
LIT:QUE	1	Some concerns	Suspected	No concerns	No concerns	Major concerns	No concerns	Very low
OLA:PLA	2	No concerns	Suspected	No concerns	No concerns	No concerns	No concerns	Moderate
OLA:RISLAI	1	No concerns	Suspected	No concerns	Major concerns	No concerns	No concerns	Low
PAL:PLA	1	No concerns	Suspected	No concerns	Major concerns	No concerns	Major concerns	Very low
PLA:QUE	2	Some concerns	Suspected	No concerns	No concerns	Major concerns	No concerns	Very low
PLA:RISLAI	1	No concerns	Suspected	No concerns	Major concerns	No concerns	No concerns	Low
AOM:ARI+LAM	0	No concerns	Suspected	No concerns	Major concerns	No concerns	Major concerns	Very low
AOM:ASE	0	No concerns	Suspected	No concerns	Major concerns	No concerns	Major concerns	Very low
AOM:LAM	0	Some concerns	Suspected	No concerns	Major concerns	No concerns	Major concerns	Very low
AOM:LIT	0	No concerns	Suspected	No concerns	Major concerns	No concerns	Major concerns	Very low
AOM:OLA	0	No concerns	Suspected	No concerns	Major concerns	No concerns	Major concerns	Very low
AOM:PAL	0	No concerns	Suspected	No concerns	Major concerns	No concerns	Major concerns	Very low
AOM:QUE	0	No concerns	Suspected	No concerns	Major concerns	No concerns	Major concerns	Very low
AOM:RISLAI	0	No concerns	Suspected	No concerns	Major concerns	No concerns	Major concerns	Very low
ARI+LAM:ASE	0	No concerns	Suspected	No concerns	Major concerns	No concerns	Major concerns	Very low
ARI+LAM:LIT	0	Some concerns	Suspected	No concerns	Major concerns	No concerns	Major concerns	Very low
ARI+LAM:OLA	0	No concerns	Suspected	No concerns	Major concerns	No concerns	Major concerns	Very low
ARI+LAM:PAL	0	No concerns	Suspected	No concerns	Major concerns	No concerns	Major concerns	Very low
ARI+LAM:PLA	0	Some concerns	Suspected	No concerns	Major concerns	No concerns	Major concerns	Very low
ARI+LAM:QUE	0	Some concerns	Suspected	No concerns	Major concerns	No concerns	Major concerns	Very low
ARI+LAM:RISLAI	0	No concerns	Suspected	No concerns	Major concerns	No concerns	Major concerns	Very low
ASE:LAM	0	Some concerns	Suspected	No concerns	Major concerns	No concerns	Major concerns	Very low
ASE:LIT	0	No concerns	Suspected	No concerns	Major concerns	No concerns	Major concerns	Very low
ASE:OLA	0	No concerns	Suspected	No concerns	Major concerns	No concerns	Major concerns	Very low
ASE:PAL	0	No concerns	Suspected	No concerns	Major concerns	No concerns	Major concerns	Very low

ASE:QUE	0	No concerns	Suspected	No concerns	Major concerns	No concerns	Major concerns	Very low
ASE:RISLAI	0	No concerns	Suspected	No concerns	Major concerns	No concerns	Major concerns	Very low
LAM:OLA	0	No concerns	Suspected	No concerns	No concerns	Major concerns	Major concerns	Very low
LAM:PAL	0	Some concerns	Suspected	No concerns	Major concerns	No concerns	Major concerns	Very low
LAM:QUE	0	Some concerns	Suspected	No concerns	No concerns	Major concerns	Major concerns	Very low
LAM:RISLAI	0	No concerns	Suspected	No concerns	Major concerns	No concerns	Major concerns	Very low
LIT:PAL	0	No concerns	Suspected	No concerns	Major concerns	No concerns	Major concerns	Very low
LIT:RISLAI	0	No concerns	Suspected	No concerns	Major concerns	No concerns	Major concerns	Very low
OLA:PAL	0	No concerns	Suspected	No concerns	Major concerns	No concerns	Major concerns	Very low
OLA:QUE	0	No concerns	Suspected	No concerns	Major concerns	No concerns	Major concerns	Very low
PAL:QUE	0	No concerns	Suspected	No concerns	Major concerns	No concerns	Major concerns	Very low
PAL:RISLAI	0	No concerns	Suspected	No concerns	Major concerns	No concerns	Major concerns	Very low
QUE:RISLAI	0	No concerns	Suspected	No concerns	Major concerns	No concerns	Major concerns	Very low

1.12. Prolactin-related adverse events (3 studies, 838 patients)

League table

Results from pairwise meta-analysis are presented in the left lower half and results from network meta-analysis in the upper right half.

The boldface result indicates statistical significance.

PAL	1.638 (0.169, 15.881)	7.892 (1.000, 62.321)
	RISLAI	**4.819 (1.875, 12.389)**
7.892 (1.000, 62.321)	**4.819 (1.875, 12.389)**	PLA

Evaluation of heterogeneity and inconsistency

Between study variance (τ^2)	Heterogeneity assessment	Random-effects design-by-treatment interaction model		
		Q	df	p
0.01	Low	Not estimate		

Rank probabilities

	Rank 1	Rank 2	Rank 3
PAL	0.080	0.355	0.565
RISLAI	0.038	0.533	0.429
PLA	0.882	0.112	0.006

SUCRA

PLA	0.97
RISLAI	0.34
PAL	0.19

Comparison-adjusted funnel plot of studies of drugs versus placebo

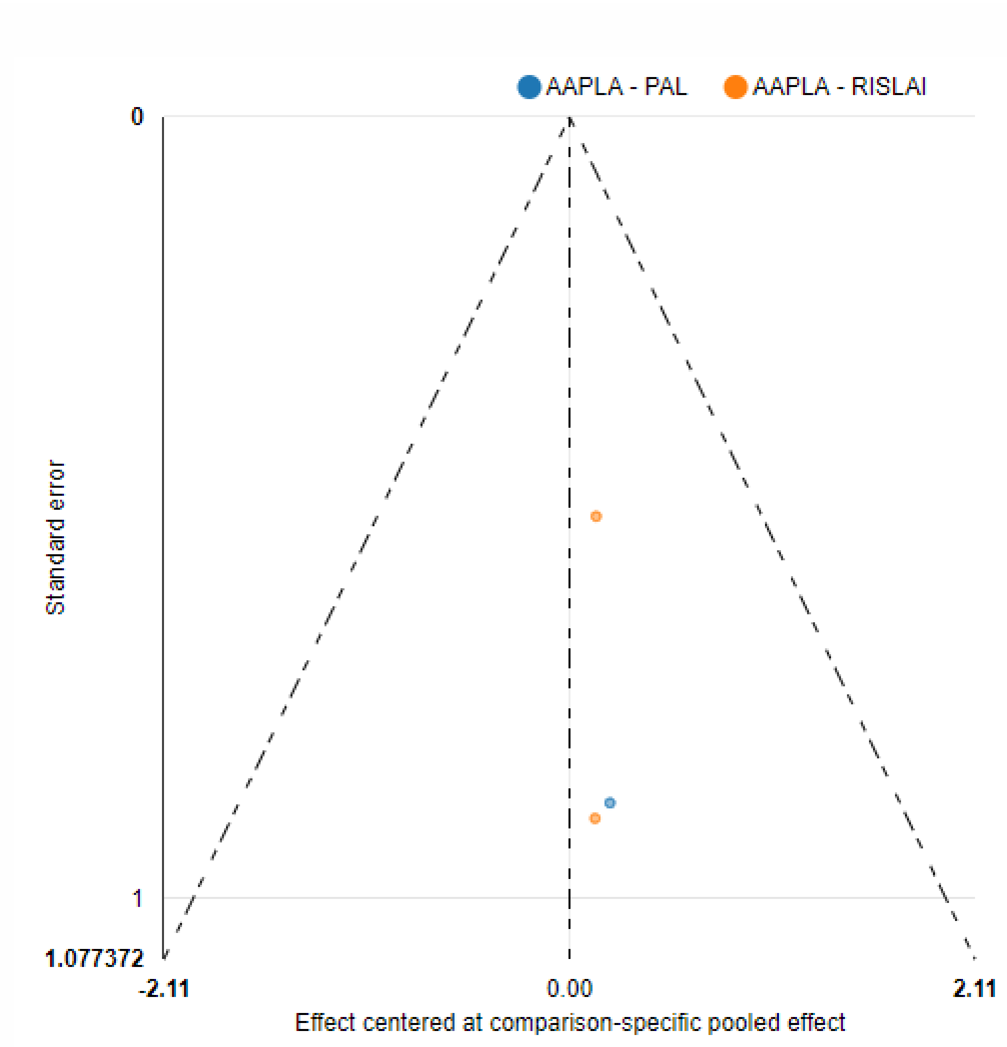

CINeMA rating

Comparison	Number of studies	Within-study bias	Reporting bias	Indirectness	Imprecision	Heterogeneity	Incoherence	Confidence rating
PAL:PLA	1	No concerns	Suspected	No concerns	Major concerns	No concerns	Major concerns	Very low
PLA:RISLAI	2	No concerns	Suspected	No concerns	No concerns	Major concerns	Major concerns	Very low
PAL:RISLAI	0	No concerns	Suspected	No concerns	Major concerns	No concerns	Major concerns	Very low

1.13. Dry mouth (7 studies, 2117 patients)

League table

Results from pairwise meta-analysis are presented in the left lower half and results from network meta-analysis in the upper right half.

The boldface result indicates statistical significance.

ARI	6.468 (0.076, 546.864)	6.021 (0.569, 63.669)	3.234 (0.336, 31.122)	2.675 (0.130, 55.009)	13.112 (0.547, 314.442)	1.829 (0.172, 19.470)	8.187 (0.833, 80.463)	6.468 (0.797, 52.510)
	ASE	0.931 (0.016, 53.958)	0.500 (0.009, 27.459)	0.414 (0.005, 36.459)	2.027 (0.021, 198.522)	0.283 (0.005, 16.456)	1.266 (0.023, 70.351)	1.000 (0.020, 50.009)
		CAR	0.537 (0.278, 1.039)	0.444 (0.039, 5.076)	2.178 (0.158, 30.039)	0.304 (0.065, 1.423)	1.360 (0.501, 3.691	1.074 (0.363, 3.178)
		0.537 (0.278, 1.039)	LIT	0.827 (0.079, 8.629)	4.054 (0.320, 51.408)	0.566 (0.140, 2.285)	**2.532 (1.196, 5.359)**	2.000 (0.845, 4.731)
				OLA	4.901 (0.193, 124.549)	0.684 (0.059, 7.863)	3.061 (0.288, 32.580)	2.418 (0.273, 21.409)
					PAL	0.139 (0.010, 1.936)	0.625 (0.048, 8.068)	0.493 (0.045, 5.382)
						QUE	**4.476 (1.071, 18.707)**	**3.536 (1.178, 10.615)**
			**2.532 (1.196, 5.359)**				VAL	0.790 (0.316, 1.972)
6.468 (0.797, 52.510)	1.000 (0.020, 50.009)		2.000 (0.845, 4.731)	2.418 (0.273, 21.409)	0.493 (0.045, 5.382)	**3.536 (1.178, 10.615)**	0.790 (0.316, 1.972)	PLA

Evaluation of heterogeneity and inconsistency

Between study variance (τ^2)	Heterogeneity assessment	Random-effects design-by-treatment interaction model		
		Q	df	p
0.15	High	Not estimate		

Rank probabilities

	Rank 1	Rank 2	Rank 3	Rank 4	Rank 5	Rank 6	Rank 7	Rank 8	Rank 9
ARI	0.001	0.018	0.033	0.042	0.062	0.077	0.123	0.216	0.428
ASE	0.923	0.036	0.010	0.006	0.006	0.004	0.004	0.004	0.007
CAR	0.008	0.108	0.167	0.166	0.146	0.150	0.113	0.081	0.061
LIT	0.001	0.007	0.039	0.081	0.127	0.205	0.267	0.194	0.081
OLA	0.005	0.091	0.155	0.109	0.116	0.115	0.147	0.138	0.124
PAL	0.055	0.558	0.137	0.072	0.054	0.043	0.039	0.029	0.014
QUE	0.001	0.017	0.036	0.046	0.072	0.105	0.162	0.289	0.272
VAL	0.007	0.146	0.286	0.216	0.145	0.093	0.063	0.031	0.013
PLA	0.000	0.019	0.137	0.263	0.271	0.208	0.082	0.017	0.002

SUCRA

PAL	0.77
VAL	0.71
CAR	0.67
PLA	0.66
LIT	0.42
OLA	0.39
ASE	0.34
QUE	0.33
ARI	0.21

Comparison-adjusted funnel plot of studies of drugs versus placebo

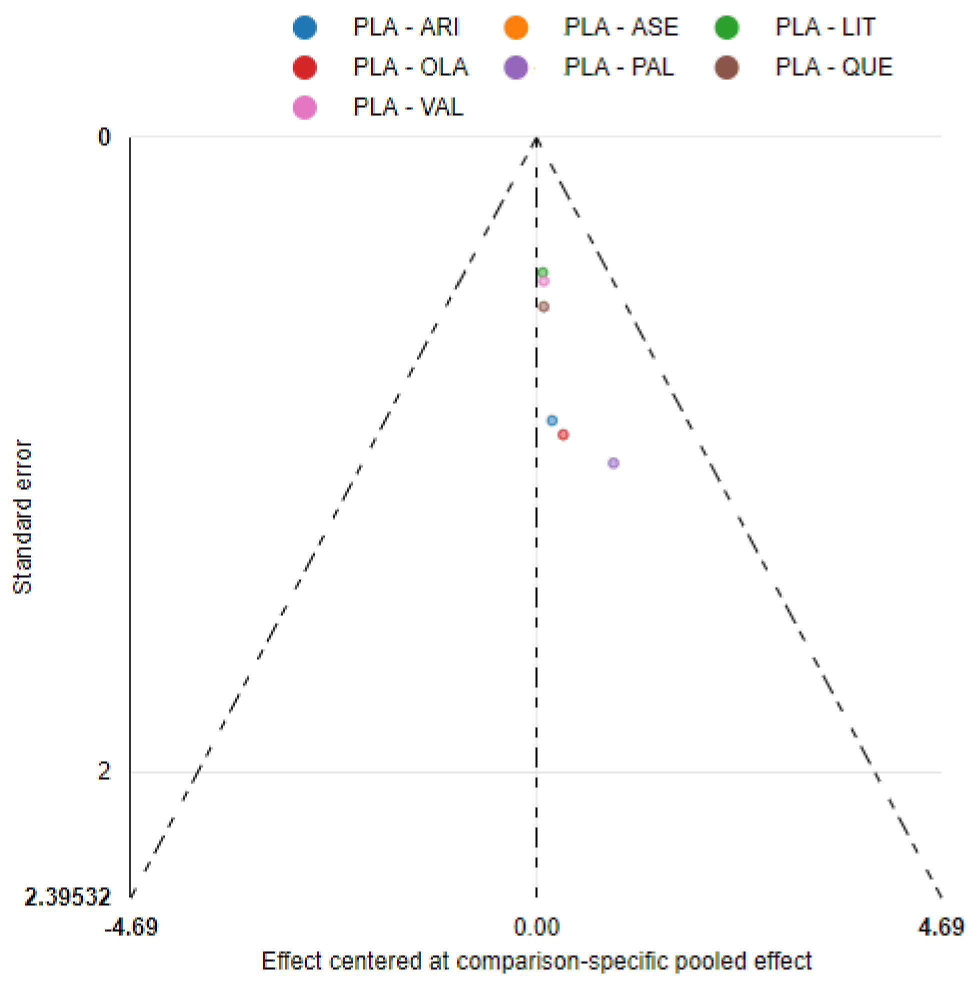

CINeMA rating

Comparison	Number of studies	Within-study bias	Reporting bias	Indirectness	Imprecision	Heterogeneity	Incoherence	Confidence rating
ARI:PLA	1	No concerns	Suspected	No concerns	Major concerns	No concerns	Major concerns	Very low
ASE:PLA	1	No concerns	Suspected	No concerns	Major concerns	No concerns	Major concerns	Very low
CAR:LIT	1	No concerns	Suspected	No concerns	Major concerns	No concerns	Major concerns	Very low
LIT:PLA	1	No concerns	Suspected	No concerns	Major concerns	No concerns	Major concerns	Very low
LIT:VAL	1	No concerns	Suspected	No concerns	No concerns	Major concerns	Major concerns	Very low
OLA:PLA	1	No concerns	Suspected	No concerns	Major concerns	No concerns	Major concerns	Very low
PAL:PLA	1	No concerns	Suspected	No concerns	Major concerns	No concerns	Major concerns	Very low
PLA:QUE	1	No concerns	Suspected	No concerns	No concerns	Major concerns	Major concerns	Very low
PLA:VAL	1	No concerns	Suspected	No concerns	Major concerns	No concerns	Major concerns	Very low
ARI:ASE	0	No concerns	Suspected	No concerns	Major concerns	No concerns	Major concerns	Very low
ARI:CAR	0	No concerns	Suspected	No concerns	Major concerns	No concerns	Major concerns	Very low
ARI:LIT	0	No concerns	Suspected	No concerns	Major concerns	No concerns	Major concerns	Very low
ARI:OLA	0	No concerns	Suspected	No concerns	Major concerns	No concerns	Major concerns	Very low
ARI:PAL	0	No concerns	Suspected	No concerns	Major concerns	No concerns	Major concerns	Very low
ARI:QUE	0	No concerns	Suspected	No concerns	Major concerns	No concerns	Major concerns	Very low
ARI:VAL	0	No concerns	Suspected	No concerns	Major concerns	No concerns	Major concerns	Very low
ASE:CAR	0	No concerns	Suspected	No concerns	Major concerns	No concerns	Major concerns	Very low
ASE:LIT	0	No concerns	Suspected	No concerns	Major concerns	No concerns	Major concerns	Very low
ASE:OLA	0	No concerns	Suspected	No concerns	Major concerns	No concerns	Major concerns	Very low
ASE:PAL	0	No concerns	Suspected	No concerns	Major concerns	No concerns	Major concerns	Very low
ASE:QUE	0	No concerns	Suspected	No concerns	Major concerns	No concerns	Major concerns	Very low
ASE:VAL	0	No concerns	Suspected	No concerns	Major concerns	No concerns	Major concerns	Very low
CAR:OLA	0	No concerns	Suspected	No concerns	Major concerns	No concerns	Major concerns	Very low
CAR:PAL	0	No concerns	Suspected	No concerns	Major concerns	No concerns	Major concerns	Very low
CAR:PLA	0	No concerns	Suspected	No concerns	Major concerns	No concerns	Major concerns	Very low
CAR:QUE	0	No concerns	Suspected	No concerns	Major concerns	No concerns	Major concerns	Very low
CAR:VAL	0	No concerns	Suspected	No concerns	Major concerns	No concerns	Major concerns	Very low
LIT:OLA	0	No concerns	Suspected	No concerns	Major concerns	No concerns	Major concerns	Very low
LIT:PAL	0	No concerns	Suspected	No concerns	Major concerns	No concerns	Major concerns	Very low
LIT:QUE	0	No concerns	Suspected	No concerns	Major concerns	No concerns	Major concerns	Very low
OLA:PAL	0	No concerns	Suspected	No concerns	Major concerns	No concerns	Major concerns	Very low
OLA:QUE	0	No concerns	Suspected	No concerns	Major concerns	No concerns	Major concerns	Very low

OLA:VAL	0	No concerns	Suspected	No concerns	Major concerns	No concerns	Major concerns	Very low
PAL:QUE	0	No concerns	Suspected	No concerns	Major concerns	No concerns	Major concerns	Very low
PAL:VAL	0	No concerns	Suspected	No concerns	Major concerns	No concerns	Major concerns	Very low
QUE:VAL	0	No concerns	Suspected	No concerns	No concerns	Major concerns	Major concerns	Very low

1.14. Headache (13 studies, 4702 patients)

League table

Results from pairwise meta-analysis are presented in the left lower half and results from network meta-analysis in the upper right half.

The boldface result indicates statistical significance.

AOM	0.650 (0.136, 3.118)	0.169 (0.004, 7.644)	0.296 (0.034, 2.603)	0.375 (0.106, 1.329)	0.372 (0.106, 1.310)	0.404 (0.102, 1.600)	0.788 (0.138, 4.507)	0.398 (0.112, 1.414)	2.316 (0.336, 15.981)	**0.036 (0.002, 0.856)**	0.444 (0.133, 1.485)
	ARI+LAM	0.259 (0.006, 10.961)	0.456 (0.058, 3.595)	0.577 (0.229, 1.456)	0.572 (0.205, 1.600)	0.622 (0.190, 2.033)	1.212 (0.243, 6.049)	0.611 (0.211, 1.771)	3.560 (0.586, 21.646)	0.056 (0.003, 1.209)	0.683 (0.251, 1.858)
		ARI+VAL	1.757 (0.031, 100.263)	2.225 (0.059, 83.730)	2.206 (0.060, 80.770)	2.397 (0.062, 93.270)	4.674 (0.101, 215.422)	2.357 (0.063, 88.668)	13.732 (0.275, 686.084)	0.215 (0.025, 1.815)	2.635 (0.071, 98.190)
			ASE	1.266 (0.200, 8.028)	1.256 (0.199, 7.928)	1.364 (0.199, 9.348)	2.661 (0.294, 24.071)	1.342 (0.211, 8.531)	7.816 (0.742, 82.294)	0.122 (0.004, 3.801)	1.500 (0.246, 9.142)
	0.577 (0.229, 1.456)			LAM	0.992 (0.634, 1.552)	1.077 (0.514, 2.257)	2.101 (0.564, 7.820)	1.060 (0.628, 1.789)	**6.172 (1.311, 29.058)**	0.097 (0.005, 1.818)	1.184 (0.811, 1.730)
				1.165 (0.665, 2.042)	LIT	1.086 (0.558, 2.116)	2.118 (0.573, 7.839)	1.068 (0.686, 1.665)	**6.223 (1.349, 28.709)**	0.097 (0.005, 1.771)	1.194 (0.835, 1.708)
					1.239 (0.488, 3.145)	OLA	1.950 (0.471, 8.081)	0.984 (0.468, 2.066)	**5.729 (1.283, 25.577)**	0.090 (0.005, 1.758)	1.099 (0.568, 2.130)
							PAL	0.504 (0.135, 1.885)	2.938 (0.412, 20.947)	0.046 (0.002, 1.108)	0.564 (0.160, 1.984)

					1.289 (0.749, 2.217)			QUE	**5.825 (1.234, 27.487)**	0.091 (0.005, 1.714)	1.118 (0.755, 1.656)
						**6.046 (1.323, 27.630)**			RISLAI	**0.016 (0.001, 0.415)**	**0.192 (0.042, 0.867)**
		0.215 (0.025, 1.815)			0.097 (0.005, 1.771)					VAL	12.257 (0.660, 227.716)
0.444 (0.133, 1.485)			1.500 (0.246, 9.142)	1.088 (0.733, 1.616)	1.071 (0.719, 1.596)	1.237 (0.513, 2.979)	0.564 (0.160, 1.984)	1.208 (0.800, 1.823)	**0.205 (0.044, 0.955)**		PLA

Evaluation of heterogeneity and inconsistency

Between study variance (τ^2)	Heterogeneity assessment	Random-effects design-by-treatment interaction model		
		Q	df	p
0.033	Low to moderate	1.576	4	0.813

Inconsistency

	NMA, RR (95%CI)	Direct, RR (95%CI)	Indirect, RR (95%CI)	Inconsistency measures	
				Ratio of risk ratios	P value
LAM vs LIT	0.992 (0.634, 1.552)	1.165 (0.665, 2.042)	0.748 (0.356, 1.571)	1.559 (0.615, 3.954)	0.350
LAM vs PLA	1.184 (0.811, 1.730)	1.088 (0.733, 1.616)	3.045 (0.813, 11.398)	0.357 (0.090, 1.418)	0.143
LIT vs OLA	1.086 (0.558, 2.116)	1.239 (0.488, 3.145)	0.946 (0.364, 2.457)	1.311 (0.345, 4.975)	0.691
LIT vs QUE	1.068 (0.686, 1.665)	1.289 (0.749, 2.217)	0.733 (0.339, 1.582)	1.759 (0.686, 4.512)	0.240
LIT vs PLA	1.194 (0.835, 1.708)	1.071 (0.719, 1.596)	1.882 (0.833, 4.257)	0.569 (0.230, 1.411)	0.224
OLA vs RISLAI	5.729 (1.283, 25.577)	6.046 (1.323, 27.630)	1.035 (0.000, 5426.773)	5.840 (0.001, 34992.884)	0.691
OLA vs PLA	1.099 (0.568, 2.130)	1.237 (0.513, 2.979)	0.943 (0.346, 2.572)	1.311 (0.345, 4.975)	0.691
QUE vs PLA	1.118 (0.755, 1.656)	1.208 (0.800, 1.823)	0.509 (0.137, 1.894)	2.374 (0.599, 9.413)	0.219
RISLAI vs PLA	0.192 (0.042, 0.867)	0.205 (0.044, 0.955)	0.044 (0.000, 72.132)	4.633 (0.002, 8872.395)	0.691

Rank probabilities

	Rank 1	Rank 2	Rank 3	Rank 4	Rank 5	Rank 6	Rank 7	Rank 8	Rank 9	Rank 10	Rank 11	Rank 12
AOM	0.134	0.319	0.229	0.126	0.064	0.034	0.022	0.017	0.018	0.024	0.010	0.004
ARI+LAM	0.020	0.082	0.159	0.188	0.140	0.077	0.060	0.062	0.070	0.079	0.042	0.020
ARI+VAL	0.043	0.057	0.057	0.056	0.042	0.031	0.024	0.025	0.033	0.077	0.434	0.123
ASE	0.030	0.081	0.105	0.114	0.085	0.054	0.045	0.046	0.057	0.193	0.109	0.082
LAM	0.000	0.001	0.006	0.025	0.059	0.093	0.133	0.173	0.226	0.191	0.074	0.020
LIT	0.000	0.005	0.020	0.054	0.107	0.154	0.175	0.194	0.164	0.099	0.026	0.004
OLA	0.001	0.021	0.070	0.139	0.158	0.141	0.102	0.101	0.106	0.102	0.046	0.014
PAL	0.094	0.229	0.240	0.151	0.082	0.042	0.031	0.032	0.036	0.037	0.018	0.009
QUE	0.001	0.006	0.027	0.070	0.110	0.134	0.153	0.154	0.158	0.122	0.050	0.016
RISLAI	0.677	0.197	0.074	0.027	0.011	0.006	0.003	0.002	0.001	0.001	0.001	0.001
VAL	0.000	0.002	0.005	0.010	0.014	0.015	0.013	0.014	0.014	0.031	0.177	0.707
PLA	0.000	0.001	0.008	0.040	0.130	0.219	0.239	0.184	0.119	0.045	0.015	0.002

SUCRA

RISLAI	0.95
AOM	0.80
PAL	0.73
ARI+LAM	0.65
PLA	0.54
OLA	0.50
QUE	0.47
LIT	0.47
ASE	0.40
LAM	0.38
ARI+VAL	0.11
VAL	0.01

Comparison-adjusted funnel plot of studies of drugs versus placebo

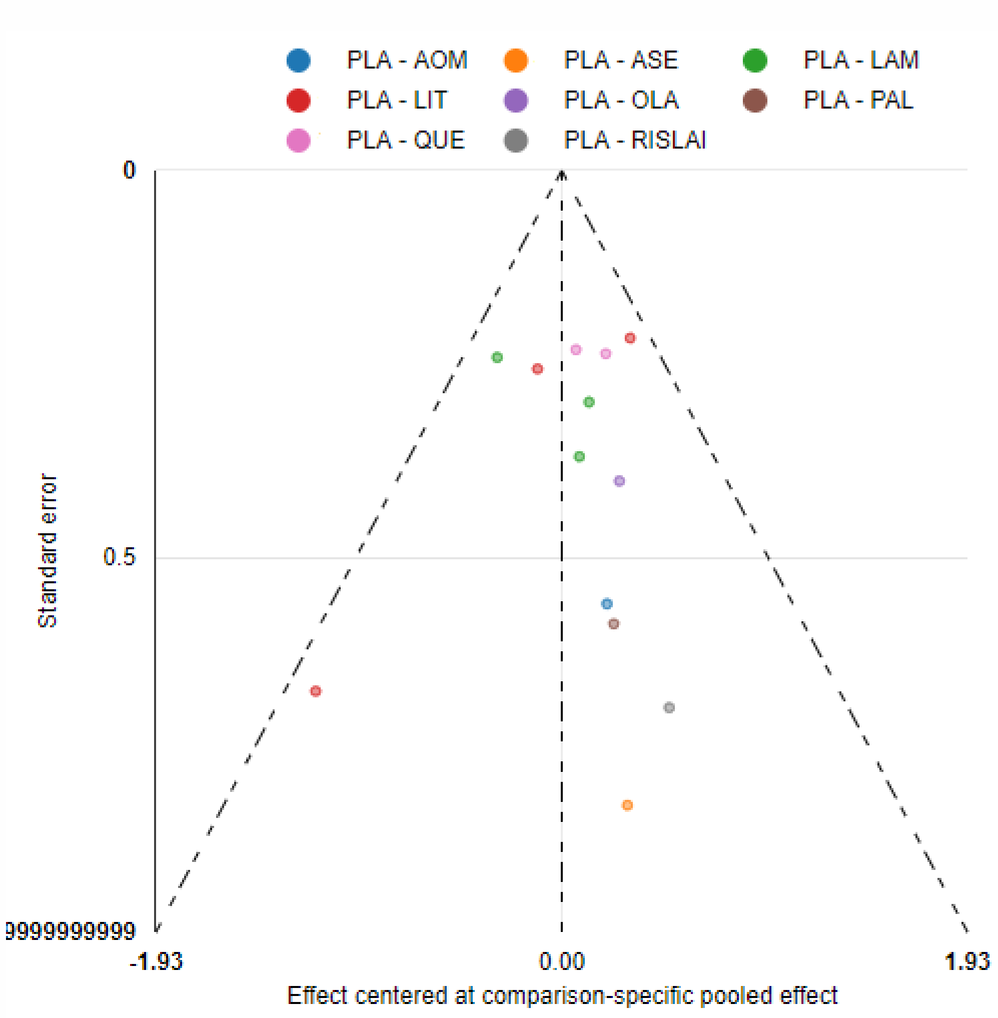

CINeMA rating

Comparison	Number of studies	Within-study bias	Reporting bias	Indirectness	Imprecision	Heterogeneity	Incoherence	Confidence rating
AOM:PLA	1	No concerns	Suspected	No concerns	Major concerns	No concerns	No concerns	Low
ARI+LAM:LAM	1	No concerns	Suspected	No concerns	Major concerns	No concerns	No concerns	Low
ARI+VAL:VAL	1	Some concerns	Suspected	No concerns	Major concerns	No concerns	No concerns	Very low
ASE:PLA	1	No concerns	Suspected	No concerns	Major concerns	No concerns	No concerns	Low
LAM:LIT	2	Some concerns	Suspected	No concerns	Major concerns	No concerns	No concerns	Very low
LAM:PLA	3	Some concerns	Suspected	No concerns	Major concerns	No concerns	No concerns	Very low
LIT:OLA	1	No concerns	Suspected	No concerns	Major concerns	No concerns	No concerns	Low
LIT:PLA	3	Some concerns	Suspected	No concerns	Major concerns	No concerns	No concerns	Very low
LIT:QUE	1	Some concerns	Suspected	No concerns	Major concerns	No concerns	No concerns	Very low
LIT:VAL	1	No concerns	Suspected	No concerns	Major concerns	No concerns	No concerns	Low
OLA:PLA	1	No concerns	Suspected	No concerns	Major concerns	No concerns	No concerns	Low
OLA:RISLAI	1	No concerns	Suspected	No concerns	No concerns	Major concerns	No concerns	Low
PAL:PLA	1	No concerns	Suspected	No concerns	Major concerns	No concerns	No concerns	Low
PLA:QUE	2	Some concerns	Suspected	No concerns	Major concerns	No concerns	No concerns	Very low
PLA:RISLAI	1	No concerns	Suspected	No concerns	No concerns	Major concerns	No concerns	Low
AOM:ARI+LAM	0	No concerns	Suspected	No concerns	Major concerns	No concerns	No concerns	Low
AOM:ARI+VAL	0	No concerns	Suspected	No concerns	Major concerns	No concerns	No concerns	Low
AOM:ASE	0	No concerns	Suspected	No concerns	Major concerns	No concerns	No concerns	Low
AOM:LAM	0	Some concerns	Suspected	No concerns	Major concerns	No concerns	No concerns	Very low
AOM:LIT	0	No concerns	Suspected	No concerns	Major concerns	No concerns	No concerns	Low
AOM:OLA	0	No concerns	Suspected	No concerns	Major concerns	No concerns	No concerns	Low
AOM:PAL	0	No concerns	Suspected	No concerns	Major concerns	No concerns	No concerns	Low
AOM:QUE	0	No concerns	Suspected	No concerns	Major concerns	No concerns	No concerns	Low
AOM:RISLAI	0	No concerns	Suspected	No concerns	Major concerns	No concerns	No concerns	Low
AOM:VAL	0	No concerns	Suspected	No concerns	No concerns	Major concerns	No concerns	Low
ARI+LAM:ARI+VAL	0	Some concerns	Suspected	No concerns	Major concerns	No concerns	No concerns	Very low
ARI+LAM:ASE	0	No concerns	Suspected	No concerns	Major concerns	No concerns	No concerns	Low
ARI+LAM:LIT	0	Some concerns	Suspected	No concerns	Major concerns	No concerns	No concerns	Very low
ARI+LAM:OLA	0	No concerns	Suspected	No concerns	Major concerns	No concerns	No concerns	Low
ARI+LAM:PAL	0	No concerns	Suspected	No concerns	Major concerns	No concerns	No concerns	Low
ARI+LAM:PLA	0	Some concerns	Suspected	No concerns	Major concerns	No concerns	No concerns	Very low
ARI+LAM:QUE	0	Some concerns	Suspected	No concerns	Major concerns	No concerns	No concerns	Very low

ARI+LAM:RISLAI	0	No concerns	Suspected	No concerns	Major concerns	No concerns	No concerns	Low
ARI+LAM:VAL	0	No concerns	Suspected	No concerns	Major concerns	No concerns	No concerns	Low
ARI+VAL:ASE	0	No concerns	Suspected	No concerns	Major concerns	No concerns	No concerns	Low
ARI+VAL:LAM	0	Some concerns	Suspected	No concerns	Major concerns	No concerns	No concerns	Very low
ARI+VAL:LIT	0	No concerns	Suspected	No concerns	Major concerns	No concerns	No concerns	Low
ARI+VAL:OLA	0	No concerns	Suspected	No concerns	Major concerns	No concerns	No concerns	Low
ARI+VAL:PAL	0	No concerns	Suspected	No concerns	Major concerns	No concerns	No concerns	Low
ARI+VAL:PLA	0	Some concerns	Suspected	No concerns	Major concerns	No concerns	No concerns	Very low
ARI+VAL:QUE	0	Some concerns	Suspected	No concerns	Major concerns	No concerns	No concerns	Very low
ARI+VAL:RISLAI	0	No concerns	Suspected	No concerns	Major concerns	No concerns	No concerns	Low
ASE:LAM	0	Some concerns	Suspected	No concerns	Major concerns	No concerns	No concerns	Very low
ASE:LIT	0	No concerns	Suspected	No concerns	Major concerns	No concerns	No concerns	Low
ASE:OLA	0	No concerns	Suspected	No concerns	Major concerns	No concerns	No concerns	Low
ASE:PAL	0	No concerns	Suspected	No concerns	Major concerns	No concerns	No concerns	Low
ASE:QUE	0	No concerns	Suspected	No concerns	Major concerns	No concerns	No concerns	Low
ASE:RISLAI	0	No concerns	Suspected	No concerns	Major concerns	No concerns	No concerns	Low
ASE:VAL	0	No concerns	Suspected	No concerns	Major concerns	No concerns	No concerns	Low
LAM:OLA	0	No concerns	Suspected	No concerns	Major concerns	No concerns	No concerns	Low
LAM:PAL	0	Some concerns	Suspected	No concerns	Major concerns	No concerns	No concerns	Very low
LAM:QUE	0	Some concerns	Suspected	No concerns	Major concerns	No concerns	No concerns	Very low
LAM:RISLAI	0	No concerns	Suspected	No concerns	No concerns	Major concerns	No concerns	Low
LAM:VAL	0	Some concerns	Suspected	No concerns	Major concerns	No concerns	No concerns	Very low
LIT:PAL	0	No concerns	Suspected	No concerns	Major concerns	No concerns	No concerns	Low
LIT:RISLAI	0	No concerns	Suspected	No concerns	No concerns	Major concerns	No concerns	Low
OLA:PAL	0	No concerns	Suspected	No concerns	Major concerns	No concerns	No concerns	Low
OLA:QUE	0	No concerns	Suspected	No concerns	Major concerns	No concerns	No concerns	Low
OLA:VAL	0	No concerns	Suspected	No concerns	Major concerns	No concerns	No concerns	Low
PAL:QUE	0	No concerns	Suspected	No concerns	Major concerns	No concerns	No concerns	Low
PAL:RISLAI	0	No concerns	Suspected	No concerns	Major concerns	No concerns	No concerns	Low
PAL:VAL	0	No concerns	Suspected	No concerns	Major concerns	No concerns	No concerns	Low
PLA:VAL	0	No concerns	Suspected	No concerns	Major concerns	No concerns	No concerns	Low
QUE:RISLAI	0	No concerns	Suspected	No concerns	No concerns	Major concerns	No concerns	Low
QUE:VAL	0	No concerns	Suspected	No concerns	Major concerns	No concerns	No concerns	Low
RISLAI:VAL	0	No concerns	Suspected	No concerns	No concerns	Major concerns	No concerns	Low

1.15. Increased weight (13 studies, 3558 patients)

League table

Results from pairwise meta-analysis are presented in the left lower half and results from network meta-analysis in the upper right half.

The boldface result indicates statistical significance.

AOM	0.333 (0.006, 17.786)	0.531 (0.005, 51.527)	1.218 (0.025, 59.377)	1.050 (0.034, 32.362)	0.376 (0.004, 37.510)	1.102 (0.044, 27.805)	1.143 (0.024, 55.031)	0.699 (0.015, 32.819)	0.588 (0.022, 15.919)	0.642 (0.017, 24.293)	1.353 (0.090, 20.344)
	ARI	1.595 (0.015, 175.107)	3.660 (0.065, 206.067)	3.155 (0.087, 114.400)	1.131 (0.010, 127.383)	3.312 (0.111, 99.236)	3.435 (0.062, 191.062)	2.100 (0.039, 114.057)	1.767 (0.055, 56.616)	1.929 (0.044, 85.123)	4.067 (0.221, 74.873)
		ARI+VAL	2.294 (0.023, 233.061)	1.978 (0.060, 65.733)	0.709 (0.007, 74.769)	2.076 (0.045, 96.467)	2.154 (0.021, 216.437)	1.317 (0.013, 129.593)	1.108 (0.019, 65.027)	1.209 (0.075, 19.511)	2.550 (0.064, 101.728)
			ASE	0.862 (0.026, 28.219)	0.309 (0.003, 32.217)	0.905 (0.034, 24.335)	0.939 (0.018, 47.660)	0.574 (0.012, 28.434)	0.483 (0.017, 13.914)	0.527 (0.013, 21.113)	1.111 (0.069, 18.017)
				LIT	0.359 (0.017, 7.720)	1.050 (0.126, 8.779)	1.089 (0.034, 35.146)	0.666 (0.021, 20.901)	0.560 (0.039, 8.060)	0.611 (0.073, 5.152)	1.289 (0.158, 10.521)
				0.359 (0.017, 7.720)	LIT+OXC	2.927 (0.070, 122.303)	3.036 (0.029, 313.093)	1.856 (0.018, 187.504)	1.562 (0.027, 91.067)	1.705 (0.041, 71.536)	3.594 (0.087, 148.117)
				0.724 (0.046, 11.479		OLA	1.037 (0.039, 27.478)	0.634 (0.025, 16.314)	0.534 (0.059, 4.866)	0.582 (0.041, 8.213)	1.228 (0.213, 7.090)
							PAL	0.611 (0.012, 29.919)	0.514 (0.018, 14.611)	0.562 (0.014, 22.200)	1.184 (0.074, 18.863)

								QUE	0.841 (0.030, 23.222)	0.919 (0.024, 35.375)	1.936 (0.126, 29.797)
						0.294 (0.018, 4.681)			RISLAI	1.092 (0.056, 21.389)	2.301 (0.351, 15.085)
		1.209 (0.075, 19.511)		0.691 (0.076, 6.288)						VAL	2.108 (0.187, 23.708)
1.353 (0.090, 20.344)	4.067 (0.221, 74.873)		1.111 (0.069, 18.017)	1.714 (0.105, 27.983)		0.874 (0.114, 6.693)	1.184 (0.074, 18.863)	1.936 (0.126, 29.797)	1.539 (0.218, 10.879)	2.801 (0.178, 44.089)	PLA

Evaluation of heterogeneity and inconsistency

Between study variance (τ^2)	Heterogeneity assessment	Random-effects design-by-treatment interaction model		
		Q	df	p
1.825	High	31.210	4	**0.000**

Inconsistency

	NMA, RR (95%CI)	Direct, RR (95%CI)	Indirect, RR (95%CI)	Inconsistency measures	
				Ratio of risk ratios	P value
LIT vs OLA	1.050 (0.126, 8.779)	0.724 (0.046, 11.479)	1.794 (0.065, 49.630)	0.404 (0.005, 30.341)	0.681
LIT vs VAL	0.611 (0.073, 5.152)	0.691 (0.076, 6.288)	0.118 (0.000, 394.729)	5.846 (0.001, 26238.944)	0.681
LIT vs PLA	1.289 (0.158, 10.521)	1.714 (0.105, 27.983)	0.890 (0.037, 21.484)	1.927 (0.028, 133.113)	0.761
OLA vs RISLAI	0.534 (0.059, 4.866)	0.294 (0.018, 4.681)	1.524 (0.039, 59.985)	0.193 (0.002, 19.161)	0.483
OLA vs PLA	1.228 (0.213, 7.090)	0.874 (0.114, 6.693)	3.263 (0.103, 103.069)	0.268 (0.005, 14.752)	0.520
RISLAI vs PLA	2.301 (0.351, 15.085)	1.539 (0.218, 10.879)	309.978 (0.336, 285614.992)	0.005 (0.000, 6.020)	0.143
VAL vs PLA	2.108 (0.187, 23.708)	2.801 (0.178, 44.089)	0.811 (0.005, 127.218)	3.455 (0.011, 1094.661)	0.673

Rank probabilities

	Rank 1	Rank 2	Rank 3	Rank 4	Rank 5	Rank 6	Rank 7	Rank 8	Rank 9	Rank 10	Rank 11	Rank 12
AOM	0.123	0.113	0.086	0.077	0.069	0.072	0.079	0.079	0.085	0.079	0.081	0.058
ARI	0.039	0.041	0.038	0.038	0.045	0.047	0.058	0.077	0.093	0.119	0.162	0.243
ARI+VAL	0.097	0.069	0.055	0.047	0.048	0.052	0.062	0.068	0.084	0.098	0.136	0.184
ASE	0.200	0.126	0.091	0.070	0.071	0.073	0.065	0.069	0.065	0.066	0.059	0.046
LIT	0.046	0.095	0.101	0.112	0.112	0.113	0.114	0.114	0.088	0.064	0.033	0.008
LIT+OXC	0.088	0.062	0.054	0.050	0.041	0.053	0.057	0.067	0.084	0.097	0.130	0.219
OLA	0.076	0.112	0.126	0.124	0.131	0.115	0.106	0.084	0.061	0.039	0.020	0.004
PAL	0.178	0.117	0.086	0.074	0.069	0.074	0.071	0.070	0.071	0.074	0.064	0.053
QUE	0.083	0.078	0.070	0.061	0.066	0.068	0.076	0.085	0.091	0.110	0.114	0.098
RISLAI	0.017	0.039	0.051	0.063	0.077	0.095	0.106	0.118	0.129	0.133	0.111	0.060
VAL	0.033	0.064	0.079	0.078	0.081	0.093	0.102	0.114	0.124	0.115	0.089	0.028
PLA	0.020	0.084	0.163	0.206	0.190	0.146	0.104	0.056	0.025	0.006	0.001	0.000

SUCRA

PLA	0.69
OLA	0.62
ASE	0.61
LIT	0.60
PAL	0.60
AOM	0.56
QUE	0.48
VAL	0.43
RISLAI	0.42
ARI+VAL	0.40
LIT+OXC	0.32
ARI	0.29

Comparison-adjusted funnel plot of studies of drugs versus placebo

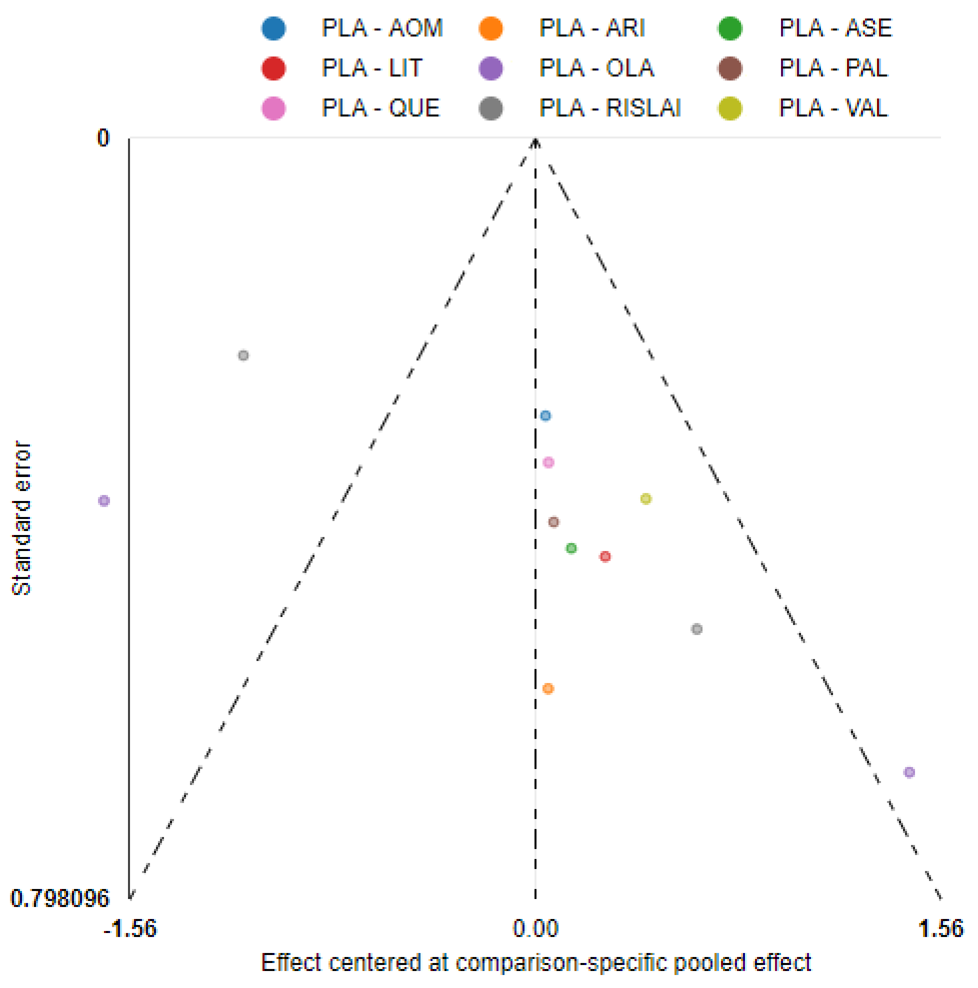

CINeMA rating

Comparison	Number of studies	Within-study bias	Reporting bias	Indirectness	Imprecision	Heterogeneity	Incoherence	Confidence rating
AOM:PLA	1	No concerns	Suspected	No concerns	Major concerns	No concerns	Major concerns	Very low
ARI:PLA	1	No concerns	Suspected	No concerns	Major concerns	No concerns	Major concerns	Very low
ARI+VAL:VAL	1	Some concerns	Suspected	No concerns	Major concerns	No concerns	Major concerns	Very low
ASE:PLA	1	No concerns	Suspected	No concerns	Major concerns	No concerns	Major concerns	Very low
LIT:LIT+OXC	1	No concerns	Suspected	No concerns	Major concerns	No concerns	Major concerns	Very low
LIT:OLA	1	No concerns	Suspected	No concerns	Major concerns	No concerns	No concerns	Low
LIT:PLA	1	No concerns	Suspected	No concerns	Major concerns	No concerns	No concerns	Low
LIT:VAL	2	No concerns	Suspected	No concerns	Major concerns	No concerns	No concerns	Low
OLA:PLA	2	No concerns	Suspected	No concerns	Major concerns	No concerns	No concerns	Low
OLA:RISLAI	1	No concerns	Suspected	No concerns	Major concerns	No concerns	No concerns	Low
PAL:PLA	1	No concerns	Suspected	No concerns	Major concerns	No concerns	Major concerns	Very low
PLA:QUE	1	No concerns	Suspected	No concerns	Major concerns	No concerns	Major concerns	Very low
PLA:RISLAI	2	No concerns	Suspected	No concerns	Major concerns	No concerns	No concerns	Low
PLA:VAL	1	No concerns	Suspected	No concerns	Major concerns	No concerns	No concerns	Low
AOM:ARI	0	No concerns	Suspected	No concerns	Major concerns	No concerns	Major concerns	Very low
AOM:ARI+VAL	0	No concerns	Suspected	No concerns	Major concerns	No concerns	Major concerns	Very low
AOM:ASE	0	No concerns	Suspected	No concerns	Major concerns	No concerns	Major concerns	Very low
AOM:LIT	0	No concerns	Suspected	No concerns	Major concerns	No concerns	Major concerns	Very low
AOM:LIT+OXC	0	No concerns	Suspected	No concerns	Major concerns	No concerns	Major concerns	Very low
AOM:OLA	0	No concerns	Suspected	No concerns	Major concerns	No concerns	Major concerns	Very low
AOM:PAL	0	No concerns	Suspected	No concerns	Major concerns	No concerns	Major concerns	Very low
AOM:QUE	0	No concerns	Suspected	No concerns	Major concerns	No concerns	Major concerns	Very low
AOM:RISLAI	0	No concerns	Suspected	No concerns	Major concerns	No concerns	Major concerns	Very low
AOM:VAL	0	No concerns	Suspected	No concerns	Major concerns	No concerns	Major concerns	Very low
ARI:ARI+VAL	0	No concerns	Suspected	No concerns	Major concerns	No concerns	Major concerns	Very low
ARI:ASE	0	No concerns	Suspected	No concerns	Major concerns	No concerns	Major concerns	Very low
ARI:LIT	0	No concerns	Suspected	No concerns	Major concerns	No concerns	Major concerns	Very low
ARI:LIT+OXC	0	No concerns	Suspected	No concerns	Major concerns	No concerns	Major concerns	Very low
ARI:OLA	0	No concerns	Suspected	No concerns	Major concerns	No concerns	Major concerns	Very low
ARI:PAL	0	No concerns	Suspected	No concerns	Major concerns	No concerns	Major concerns	Very low
ARI:QUE	0	No concerns	Suspected	No concerns	Major concerns	No concerns	Major concerns	Very low
ARI:RISLAI	0	No concerns	Suspected	No concerns	Major concerns	No concerns	Major concerns	Very low

ARI:VAL	0	No concerns	Suspected	No concerns	Major concerns	No concerns	Major concerns	Very low
ARI+VAL:ASE	0	No concerns	Suspected	No concerns	Major concerns	No concerns	Major concerns	Very low
ARI+VAL:LIT	0	No concerns	Suspected	No concerns	Major concerns	No concerns	Major concerns	Very low
ARI+VAL:LIT+OXC	0	No concerns	Suspected	No concerns	Major concerns	No concerns	Major concerns	Very low
ARI+VAL:OLA	0	No concerns	Suspected	No concerns	Major concerns	No concerns	Major concerns	Very low
ARI+VAL:PAL	0	No concerns	Suspected	No concerns	Major concerns	No concerns	Major concerns	Very low
ARI+VAL:PLA	0	No concerns	Suspected	No concerns	Major concerns	No concerns	Major concerns	Very low
ARI+VAL:QUE	0	No concerns	Suspected	No concerns	Major concerns	No concerns	Major concerns	Very low
ARI+VAL:RISLAI	0	No concerns	Suspected	No concerns	Major concerns	No concerns	Major concerns	Very low
ASE:LIT	0	No concerns	Suspected	No concerns	Major concerns	No concerns	Major concerns	Very low
ASE:LIT+OXC	0	No concerns	Suspected	No concerns	Major concerns	No concerns	Major concerns	Very low
ASE:OLA	0	No concerns	Suspected	No concerns	Major concerns	No concerns	Major concerns	Very low
ASE:PAL	0	No concerns	Suspected	No concerns	Major concerns	No concerns	Major concerns	Very low
ASE:QUE	0	No concerns	Suspected	No concerns	Major concerns	No concerns	Major concerns	Very low
ASE:RISLAI	0	No concerns	Suspected	No concerns	Major concerns	No concerns	Major concerns	Very low
ASE:VAL	0	No concerns	Suspected	No concerns	Major concerns	No concerns	Major concerns	Very low
LIT:PAL	0	No concerns	Suspected	No concerns	Major concerns	No concerns	Major concerns	Very low
LIT:QUE	0	No concerns	Suspected	No concerns	Major concerns	No concerns	Major concerns	Very low
LIT:RISLAI	0	No concerns	Suspected	No concerns	Major concerns	No concerns	Major concerns	Very low
LIT+OXC:OLA	0	No concerns	Suspected	No concerns	Major concerns	No concerns	Major concerns	Very low
LIT+OXC:PAL	0	No concerns	Suspected	No concerns	Major concerns	No concerns	Major concerns	Very low
LIT+OXC:PLA	0	No concerns	Suspected	No concerns	Major concerns	No concerns	Major concerns	Very low
LIT+OXC:QUE	0	No concerns	Suspected	No concerns	Major concerns	No concerns	Major concerns	Very low
LIT+OXC:RISLAI	0	No concerns	Suspected	No concerns	Major concerns	No concerns	Major concerns	Very low
LIT+OXC:VAL	0	No concerns	Suspected	No concerns	Major concerns	No concerns	Major concerns	Very low
OLA:PAL	0	No concerns	Suspected	No concerns	Major concerns	No concerns	Major concerns	Very low
OLA:QUE	0	No concerns	Suspected	No concerns	Major concerns	No concerns	Major concerns	Very low
OLA:VAL	0	No concerns	Suspected	No concerns	Major concerns	No concerns	Major concerns	Very low
PAL:QUE	0	No concerns	Suspected	No concerns	Major concerns	No concerns	Major concerns	Very low
PAL:RISLAI	0	No concerns	Suspected	No concerns	Major concerns	No concerns	Major concerns	Very low
PAL:VAL	0	No concerns	Suspected	No concerns	Major concerns	No concerns	Major concerns	Very low
QUE:RISLAI	0	No concerns	Suspected	No concerns	Major concerns	No concerns	Major concerns	Very low
QUE:VAL	0	No concerns	Suspected	No concerns	Major concerns	No concerns	Major concerns	Very low
RISLAI:VAL	0	No concerns	Suspected	No concerns	Major concerns	No concerns	Major concerns	Very low

1.16. Nausea (9 studies, 3929 patients)

League table

Results from pairwise meta-analysis are presented in the left lower half and results from network meta-analysis in the upper right half.

The boldface result indicates statistical significance.

ASE	2.503 (0.101, 62.253)	1.881 (0.077, 46.132)	15.255 (0.337, 689.661)	6.082 (0.113, 327.635)	4.354 (0.174, 108.984)	2.029 (0.082, 49.999)	3.000 (0.123, 72.945)
	LAM	0.751 (0.514, 1.099)	6.095 (0.743, 50.009)	2.430 (0.216, 27.311)	**1.739 (1.001, 3.021)**	0.811 (0.523, 1.258)	1.198 (0.820, 1.752)
	0.718 (0.458, 1.127)	LIT	**8.112 (1.023, 64.303)**	3.234 (0.293, 35.698)	**2.315 (1.494, 3.587)**	1.079 (0.830, 1.404)	**1.595 (1.258, 2.023)**
		8.112 (1.023, 64.303)	OLA	0.399 (0.017, 9.496)	0.285 (0.034, 2.368)	0.133 (0.017, 1.072)	0.197 (0.024, 1.580)
				PAL	0.716 (0.063, 8.117)	0.334 (0.030, 3.705)	0.493 (0.045, 5.382)
		**2.846 (1.697, 4.772)**			QUE	**0.466 (0.286, 0.759)**	0.689 (0.448, 1.061)
		1.032 (0.777, 1.372)				VAL	**1.478 (1.106, 1.976)**
3.000 (0.123, 72.945)	1.220 (0.790, 1.882)	**1.540 (1.206, 1.966)**		0.493 (0.045, 5.382)	0.709 (0.450, 1.117)	1.369 (0.969, 1.935)	PLA

Evaluation of heterogeneity and inconsistency

Between study variance (τ^2)	Heterogeneity assessment	Random-effects design-by-treatment interaction model		
		Q	df	p
0.01	Low	3.188	4	0.527

Inconsistency

	NMA, RR (95%CI)	Direct, RR (95%CI)	Indirect, RR (95%CI)	Inconsistency measures	
				Ratio of risk ratios	P value
LAM vs LIT	0.751 (0.514, 1.099)	0.718 (0.458, 1.127)	0.840 (0.413, 1.705)	0.855 (0.369, 1.980)	0.715
LAM vs PLA	1.198 (0.820, 1.752)	1.220 (0.790, 1.882)	1.132 (0.517, 2.476)	1.078 (0.440, 2.637)	0.870
LIT vs QUE	2.315 (1.494, 3.587)	2.846 (1.697, 4.772)	1.369 (0.600, 3.123)	2.079 (0.786, 5.503)	0.141
LIT vs VAL	1.079 (0.830, 1.404)	1.032 (0.777, 1.372)	1.399 (0.702, 2.788)	0.738 (0.350, 1.556)	0.425
LIT vs PLA	1.595 (1.258, 2.023)	1.540 (1.206, 1.966)	2.909 (1.064, 7.953)	0.529 (0.188, 1.490)	0.228
QUE vs PLA	0.689 (0.448, 1.061)	0.709 (0.450, 1.117)	0.533 (0.136, 2.095)	1.329 (0.314, 5.618)	0.699
VAL vs PLA	1.478 (1.106, 1.976)	1.369 (0.969, 1.935)	1.774 (1.040, 3.027)	0.772 (0.408, 1.458)	0.425

Rank probabilities table

	Rank 1	Rank 2	Rank 3	Rank 4	Rank 5	Rank 6	Rank 7	Rank 8
ASE	0.094	0.164	0.175	0.070	0.047	0.049	0.041	0.360
LAM	0.000	0.010	0.062	0.185	0.342	0.280	0.095	0.026
LIT	0.000	0.000	0.001	0.004	0.026	0.181	0.440	0.349
OLA	0.586	0.310	0.073	0.013	0.007	0.006	0.002	0.002
PAL	0.312	0.358	0.150	0.051	0.029	0.028	0.028	0.046
QUE	0.007	0.150	0.461	0.301	0.052	0.020	0.007	0.002
VAL	0.000	0.003	0.012	0.035	0.094	0.273	0.371	0.214
PLA	0.000	0.005	0.066	0.342	0.405	0.163	0.018	0.001

SUCRA

OLA	0.93
PAL	0.74
QUE	0.73
PLA	0.58
LAM	0.47
VAL	0.29
LIT	0.22
ASE	0.03

Comparison-adjusted funnel plot of studies of drugs versus placebo

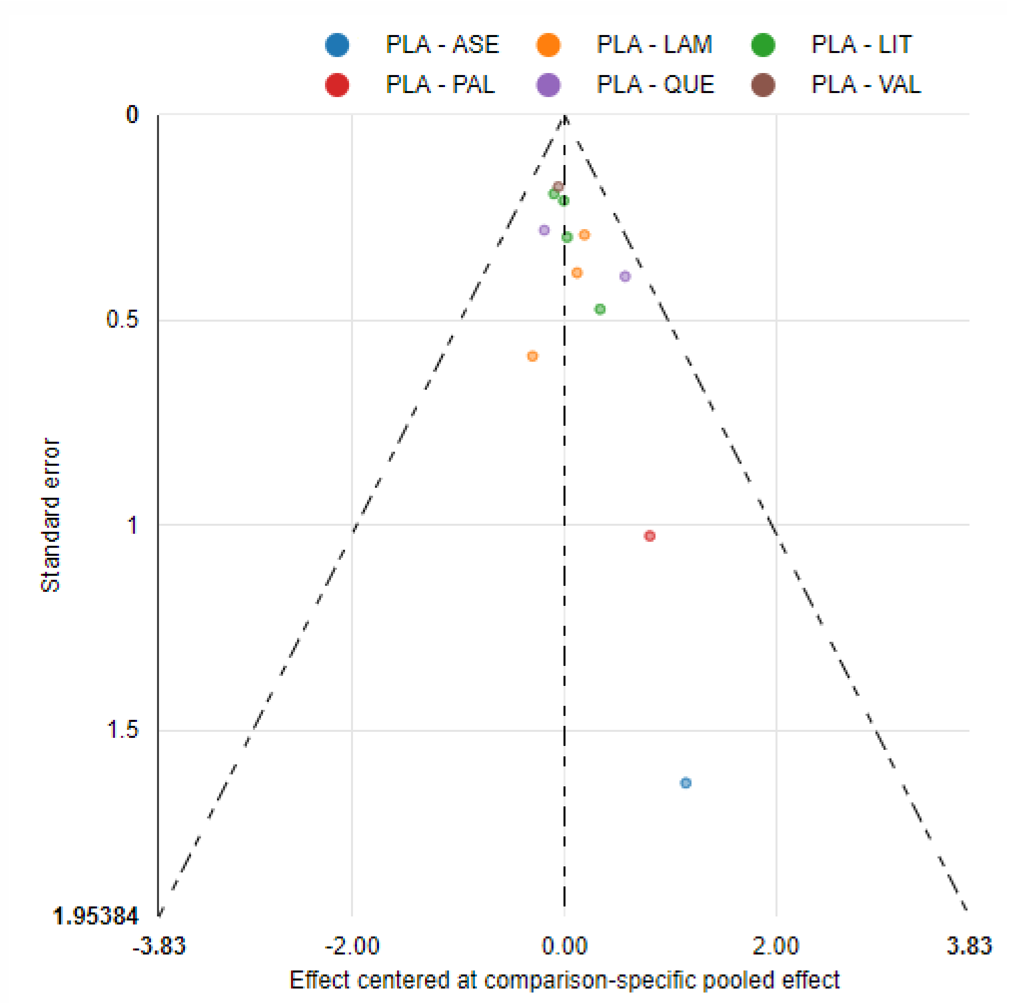

CINeMA rating

Comparison	Number of studies	Within-study bias	Reporting bias	Indirectness	Imprecision	Heterogeneity	Incoherence	Confidence rating
ASE:PLA	1	No concerns	Suspected	No concerns	Major concerns	No concerns	No concerns	Low
LAM:LIT	2	Some concerns	Suspected	No concerns	Major concerns	No concerns	No concerns	Very low
LAM:PLA	3	Some concerns	Suspected	No concerns	Major concerns	No concerns	No concerns	Very low
LIT:OLA	1	No concerns	Suspected	No concerns	No concerns	Major concerns	No concerns	Low
LIT:PLA	4	Some concerns	Suspected	No concerns	No concerns	No concerns	No concerns	Low
LIT:QUE	1	Some concerns	Suspected	No concerns	No concerns	No concerns	No concerns	Low
LIT:VAL	1	No concerns	Suspected	No concerns	Major concerns	No concerns	No concerns	Low
PAL:PLA	1	No concerns	Suspected	No concerns	Major concerns	No concerns	No concerns	Low
PLA:QUE	2	Some concerns	Suspected	No concerns	Major concerns	No concerns	No concerns	Very low
PLA:VAL	1	No concerns	Suspected	No concerns	No concerns	No concerns	No concerns	Moderate
ASE:LAM	0	Some concerns	Suspected	No concerns	Major concerns	No concerns	No concerns	Very low
ASE:LIT	0	No concerns	Suspected	No concerns	Major concerns	No concerns	No concerns	Low
ASE:OLA	0	No concerns	Suspected	No concerns	Major concerns	No concerns	No concerns	Low
ASE:PAL	0	No concerns	Suspected	No concerns	Major concerns	No concerns	No concerns	Low
ASE:QUE	0	No concerns	Suspected	No concerns	Major concerns	No concerns	No concerns	Low
ASE:VAL	0	No concerns	Suspected	No concerns	Major concerns	No concerns	No concerns	Low
LAM:OLA	0	Some concerns	Suspected	No concerns	Major concerns	No concerns	No concerns	Very low
LAM:PAL	0	Some concerns	Suspected	No concerns	Major concerns	No concerns	No concerns	Very low
LAM:QUE	0	Some concerns	Suspected	No concerns	No concerns	Major concerns	No concerns	Very low
LAM:VAL	0	Some concerns	Suspected	No concerns	Major concerns	No concerns	No concerns	Very low
LIT:PAL	0	No concerns	Suspected	No concerns	Major concerns	No concerns	No concerns	Low
OLA:PAL	0	No concerns	Suspected	No concerns	Major concerns	No concerns	No concerns	Low
OLA:PLA	0	No concerns	Suspected	No concerns	Major concerns	No concerns	No concerns	Low
OLA:QUE	0	No concerns	Suspected	No concerns	Major concerns	No concerns	No concerns	Low
OLA:VAL	0	No concerns	Suspected	No concerns	Major concerns	No concerns	No concerns	Low
PAL:QUE	0	No concerns	Suspected	No concerns	Major concerns	No concerns	No concerns	Low
PAL:VAL	0	No concerns	Suspected	No concerns	Major concerns	No concerns	No concerns	Low
QUE:VAL	0	No concerns	Suspected	No concerns	No concerns	No concerns	No concerns	Moderate

1.17. Diarrhea (5 studies, 2770 patients)

League table

Results from pairwise meta-analysis are presented in the left lower half and results from network meta-analysis in the upper right half.

The boldface result indicates statistical significance.

LAM	**0.377 (0.190, 0.749)**	0.749 (0.290, 1.932)	0.518 (0.220, 1.221)	0.656 (0.321, 1.343)
0.340 (0.165, 0.698)	LIT	1.988 (0.980, 4.033)	1.374 (0.783, 2.411)	**1.742 (1.171, 2.592)**
	2.284 (0.956, 5.459)	QUE	0.691 (0.295, 1.623)	0.877 (0.445, 1.726)
	1.285 (0.700, 2.361)		VAL	1.268 (0.712, 2.257)
0.763(0.342, 1.701)	**1.663 (1.113, 2.485)**	0.814 (0.398, 1.665)	1.167 (0.613, 2.223)	PLA

Evaluation of heterogeneity and inconsistency

Between study variance (τ^2)	Heterogeneity assessment	Random-effects design-by-treatment interaction model		
		Q	df	p
0.073	Moderate	5.667	3	0.129

Inconsistency

	NMA, RR (95%CI)	Direct, RR (95%CI)	Indirect, RR (95%CI)	Inconsistency measures	
				Ratio of risk ratios	P value
LAM vs LIT	0.340 (0.165, 0.698)	0.340 (0.165, 0.698)	1.042 (0.109, 9.959)	0.326 (0.030, 3.483)	0.353
LAM vs PLA	0.656 (0.321, 1.343)	0.763 (0.342, 1.701)	0.362 (0.073, 1.783)	2.107 (0.354, 12.557)	0.413
LIT vs QUE	1.988 (0.980, 4.033)	2.284 (0.956, 5.459)	1.517 (0.451, 5.103)	1.506 (0.338, 6.706)	0.591
LIT vs VAL	1.374 (0.783, 2.411)	1.285 (0.700, 2.361)	2.035 (0.466, 8.884)	0.632 (0.128, 3.111)	0.572
LIT vs PLA	1.742 (1.171, 2.592)	1.663 (1.113, 2.485)	14.209 (0.949, 212.725)	0.117 (0.008, 1.805)	0.124
QUE vs PLA	0.877 (0.445, 1.726)	0.814 (0.398, 1.665)	1.664 (0.203, 13.614)	0.489 (0.053, 4.502)	0.527
VAL vs PLA	1.268 (0.712, 2.257)	1.167 (0.613, 2.223)	1.770 (0.485, 6.451)	0.659 (0.155, 2.797)	0.572

Rank probabilities

	Rank 1	Rank 2	Rank 3	Rank 4	Rank 5
LAM	0.690	0.212	0.062	0.031	0.006
LIT	0.001	0.007	0.028	0.186	0.779
QUE	0.235	0.442	0.177	0.106	0.040
VAL	0.045	0.109	0.179	0.506	0.162
PLA	0.029	0.231	0.555	0.171	0.014

SUCRA

LAM	0.86
QUE	0.62
PLA	0.58
VAL	0.38
LIT	0.07

Comparison-adjusted funnel plot of studies of drugs versus placebo

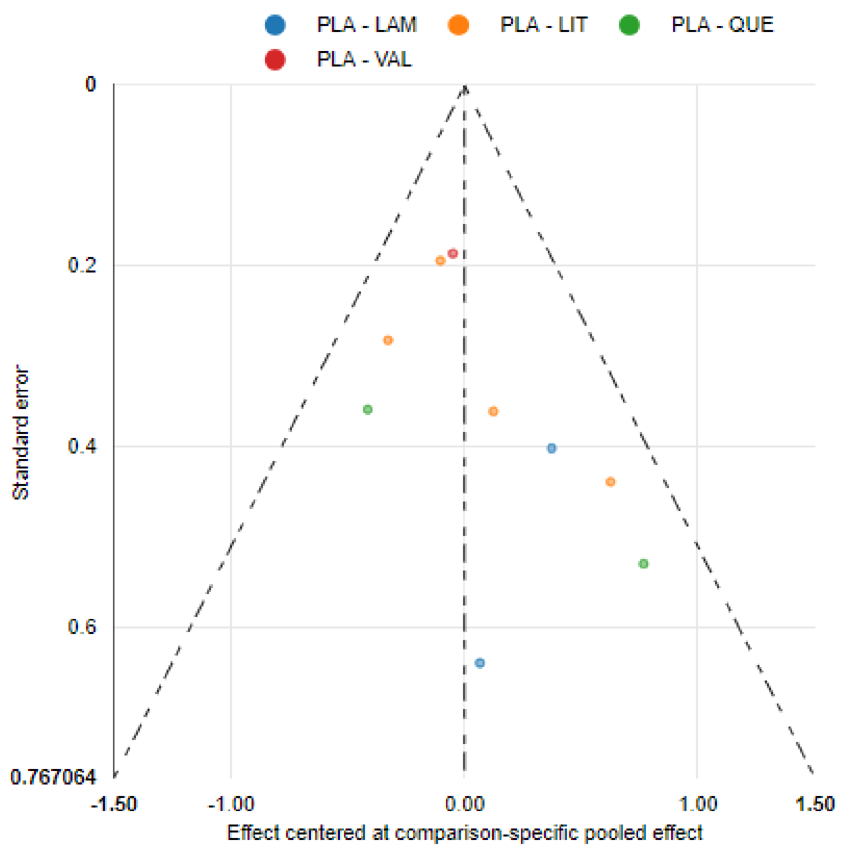

CINeMA rating

Comparison	Number of studies	Within-study bias	Reporting bias	Indirectness	Imprecision	Heterogeneity	Incoherence	Confidence rating
LAM:LIT	2	Some concerns	Suspected	No concerns	No concerns	Major concerns	No concerns	Very low
LAM:PLA	2	Some concerns	Suspected	No concerns	Major concerns	No concerns	No concerns	Very low
LIT:PLA	4	Some concerns	Suspected	No concerns	No concerns	Major concerns	No concerns	Very low
LIT:QUE	1	Some concerns	Suspected	No concerns	Major concerns	No concerns	No concerns	Very low
LIT:VAL	1	No concerns	Suspected	No concerns	Major concerns	No concerns	No concerns	Low
PLA:QUE	2	Some concerns	Suspected	No concerns	Major concerns	No concerns	No concerns	Very low
PLA:VAL	1	No concerns	Suspected	No concerns	Major concerns	No concerns	No concerns	Low
LAM:QUE	0	Some concerns	Suspected	No concerns	Major concerns	No concerns	No concerns	Very low
LAM:VAL	0	Some concerns	Suspected	No concerns	Major concerns	No concerns	No concerns	Very low
QUE:VAL	0	No concerns	Suspected	No concerns	Major concerns	No concerns	No concerns	Low

Supplementary Appendix 2. Two-dimensional plot of the result

Supplementary Appendix 2.1. Risk ratios of each drug for recurrence/relapse rate of any mood episode and for all-cause discontinuation compared to placebo

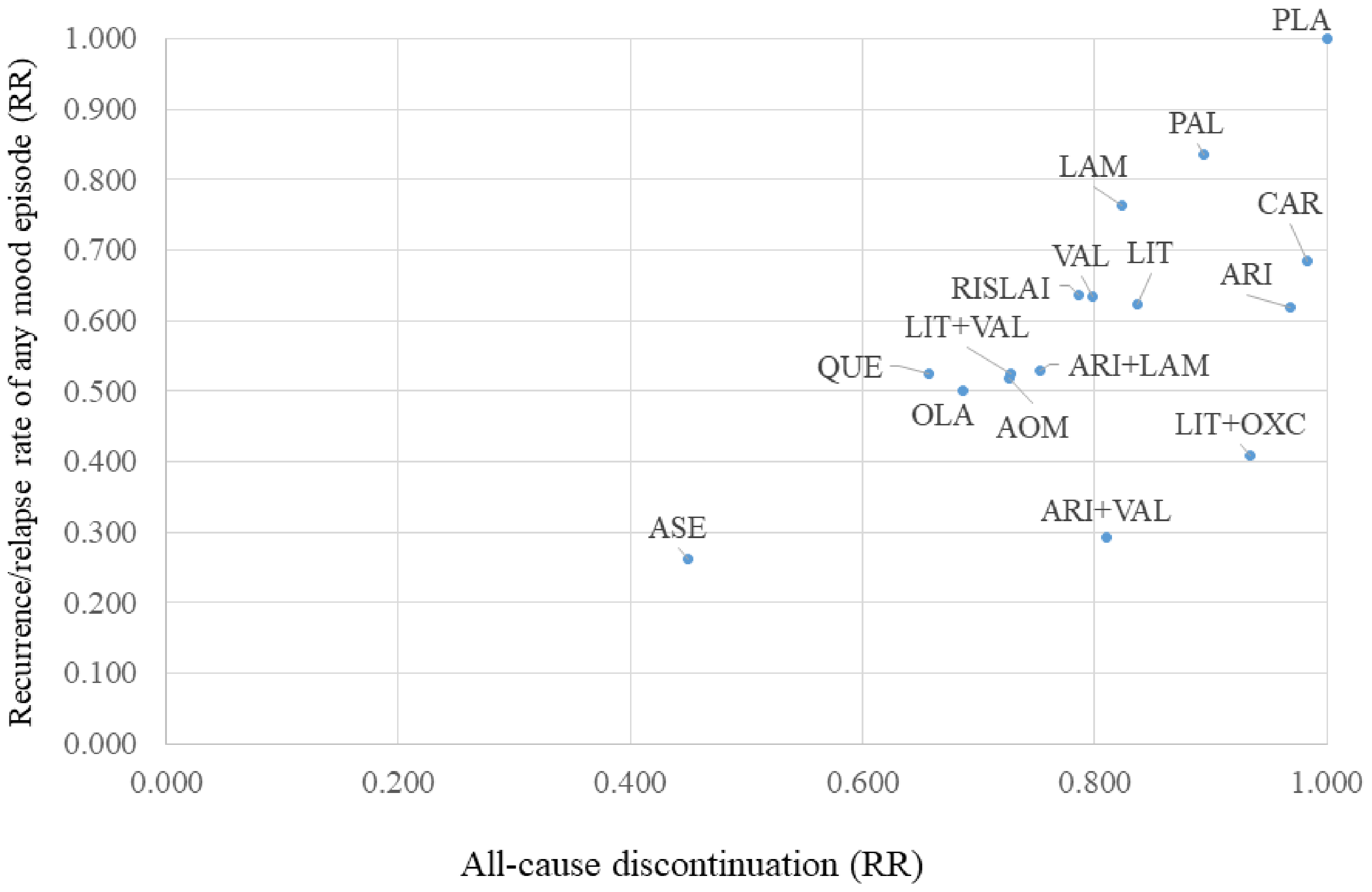

RR < 1 favors intervention

Supplementary Appendix 2.2. Risk ratios of each drug for recurrence/relapse rate of any mood episode and for discontinuation due to adverse event compared to placebo

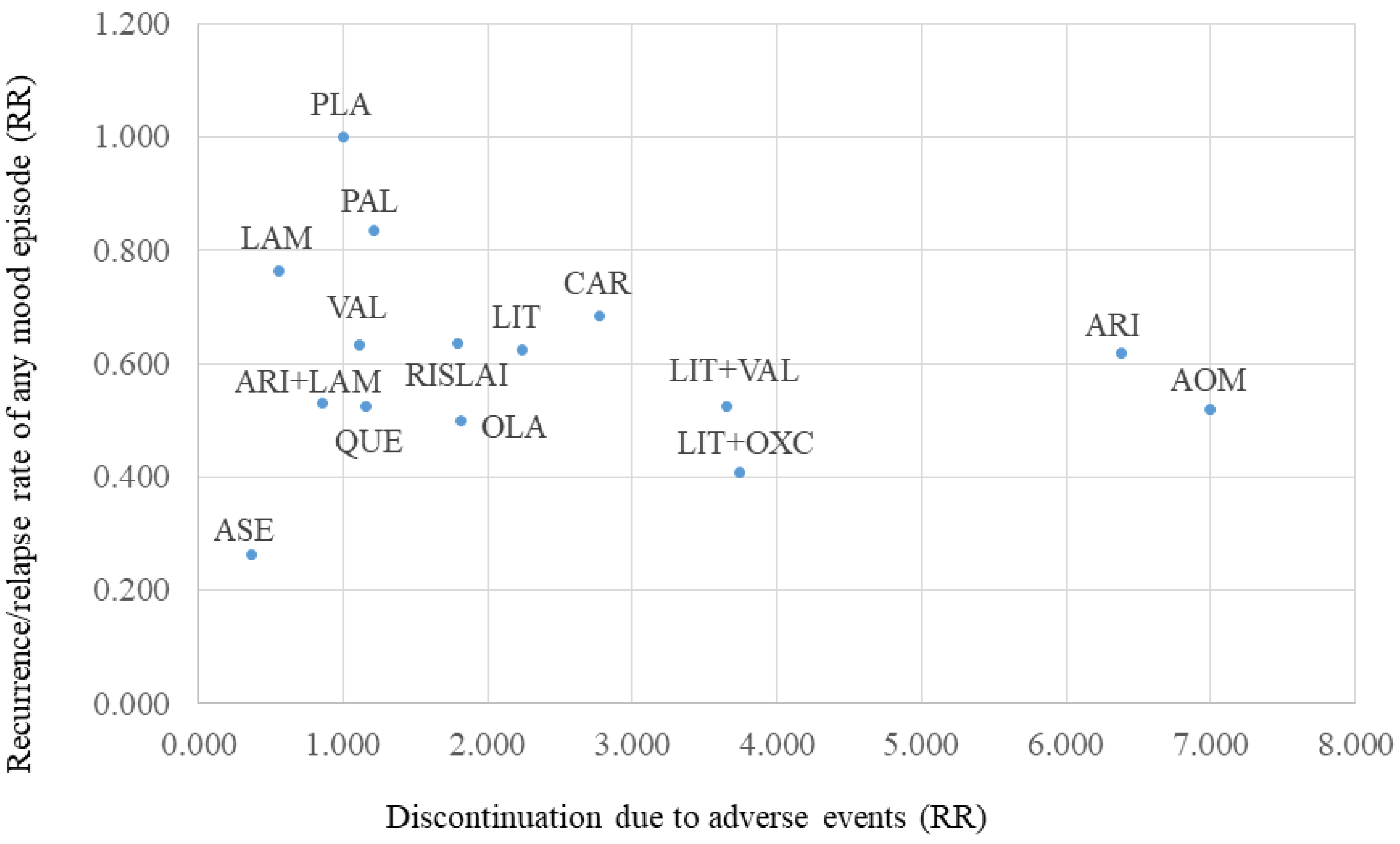

RR < 1 favors intervention

Supplementary Appendix 2.3. Risk ratios of each drug for recurrence/relapse rate of depressive episodes and for recurrence/relapse rate of manic/hypomanic/mixed episodes compared to placebo

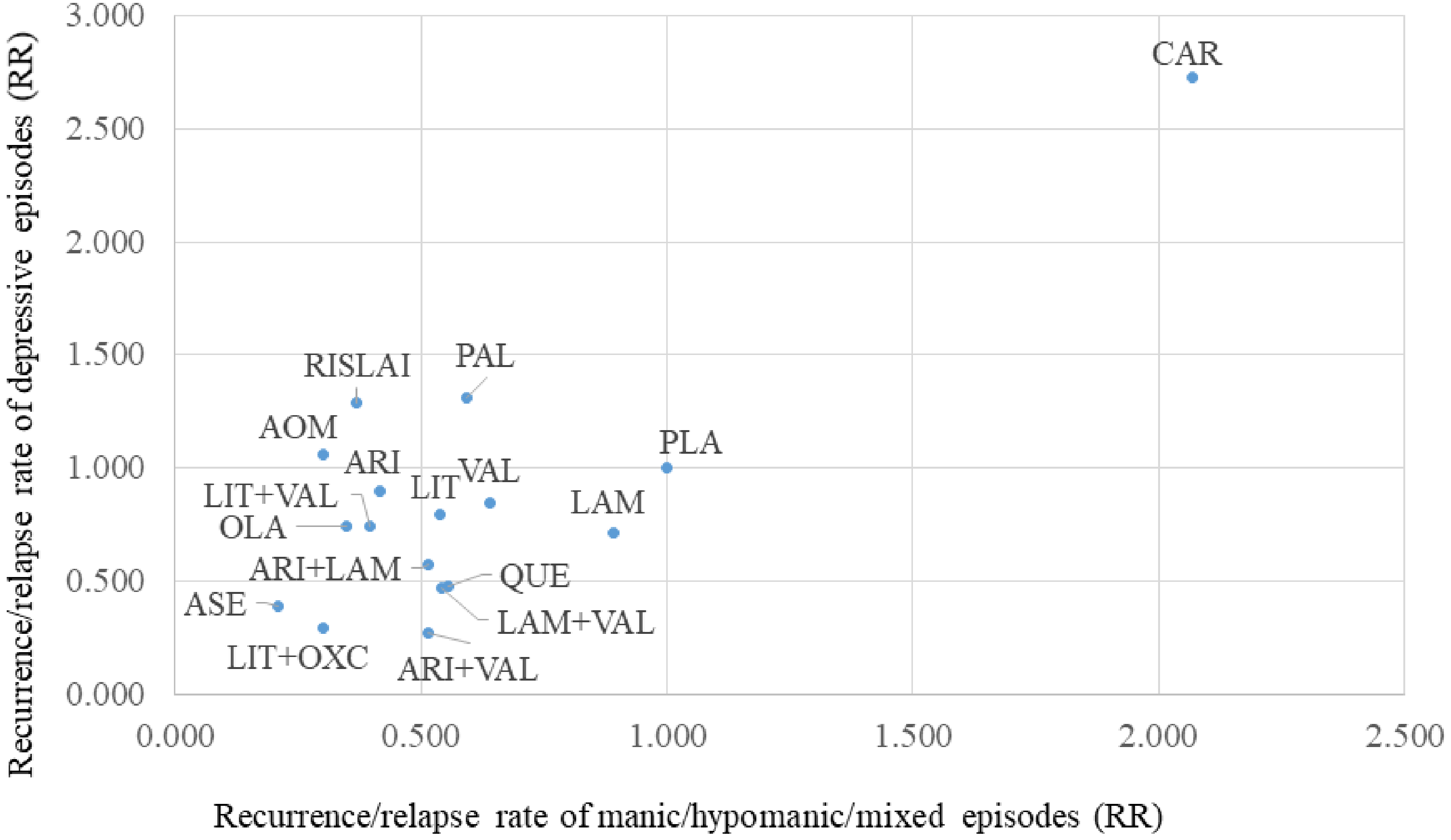

RR < 1 favors intervention

Supplementary Appendix 3. Results of second network meta-analysis

3.1. Recurrence/relapse rate of any mood episodes (5 studies, 2399 patients)

League table

Results from pairwise meta-analysis are presented in the left lower half and results from network meta-analysis in the upper right half.

The boldface result indicates statistical significance.

ARI	1.482 (0.908, 2.417)	0.633 (0.307, 1.303)	1.553 (0.998, 2.417)	0.981 (0.538, 1.789)	**0.595 (0.396, 0.894)**
	LUR	**0.427 (0.221, 0.823)**	1.048 (0.759, 1.448)	0.662 (0.394, 1.113)	**0.402 (0.306, 0.528)**
		OLA	**2.456 (1.318, 4.575)**	1.551 (0.737, 3.261)	0.941 (0.518, 1.710)
			QUE	0.631 (0.393, 1.016)	**0.383 (0.322, 0.456)**
				ZIP	**0.607 (0.390, 0.944)**
**0.595 (0.396, 0.894)**	**0.402 (0.306, 0.528)**	0.941 (0.518, 1.710)	**0.383 (0.322, 0.456)**	**0.607 (0.390, 0.944)**	PLA

Evaluation of heterogeneity and inconsistency

Between study variance (τ^2)	Heterogeneity assessment	Random-effects design-by-treatment interaction model		
		Q	df	p
0.0484	Low to moderate	Not estimate		

Rank probabilities

	Rank 1	Rank 2	Rank 3	Rank 4	Rank 5	Rank 6
ARI	0.072	0.120	0.310	0.332	0.110	0.056
LUR	0.369	0.357	0.168	0.067	0.024	0.014
OLA	0.025	0.039	0.089	0.161	0.325	0.361
QUE	0.458	0.362	0.125	0.041	0.011	0.003
ZIP	0.076	0.120	0.296	0.317	0.133	0.058
PLA	0.000	0.001	0.013	0.081	0.398	0.507

SUCRA

QUE	0.85
LUR	0.82
ZIP	0.49
ARI	0.48
OLA	0.23
PLA	0.14

Comparison-adjusted funnel plot of studies of drugs versus placebe

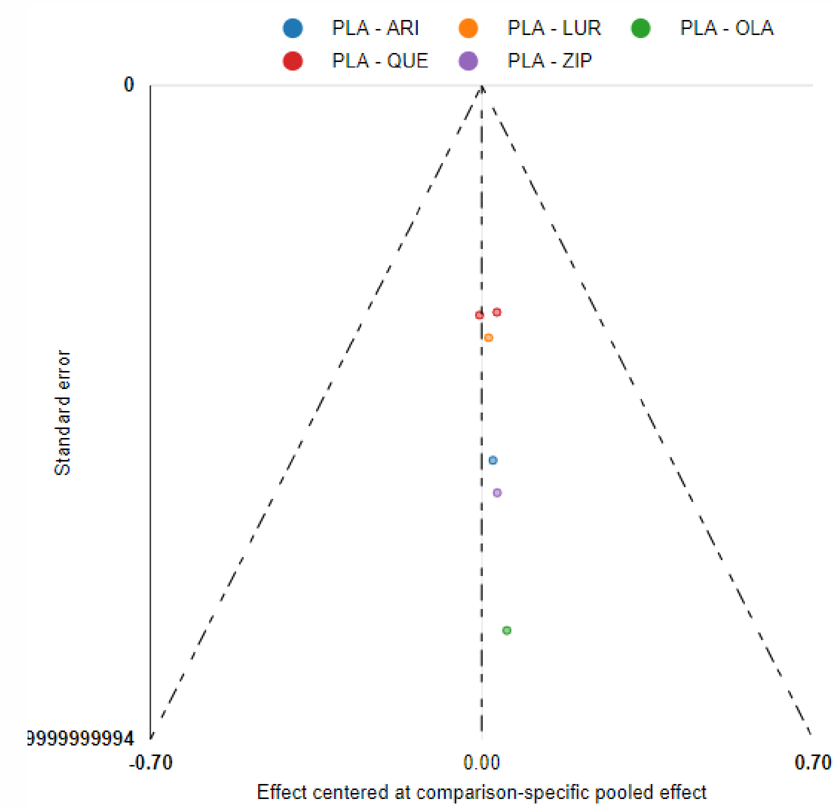

CINeMA rating

Comparison	Number of studies	Within-study bias	Reporting bias	Indirectness	Imprecision	Heterogeneity	Incoherence	Confidence rating
ARI:PLA	1	No concerns	Suspected	No concerns	No concerns	Major concerns	Major concerns	Very low
LUR:PLA	1	Some concerns	Suspected	No concerns	No concerns	Major concerns	Major concerns	Very low
OLA:PLA	1	Some concerns	Suspected	No concerns	Major concerns	No concerns	Major concerns	Very low
PLA:QUE	2	Some concerns	Suspected	No concerns	No concerns	Major concerns	Major concerns	Very low
PLA:ZIP	1	No concerns	Suspected	No concerns	No concerns	Major concerns	Major concerns	Very low
ARI:LUR	0	No concerns	Suspected	No concerns	Major concerns	No concerns	Major concerns	Very low
ARI:OLA	0	No concerns	Suspected	No concerns	Major concerns	No concerns	Major concerns	Very low
ARI:QUE	0	No concerns	Suspected	No concerns	Major concerns	No concerns	Major concerns	Very low
ARI:ZIP	0	No concerns	Suspected	No concerns	Major concerns	No concerns	Major concerns	Very low
LUR:OLA	0	Some concerns	Suspected	No concerns	No concerns	Major concerns	Major concerns	Very low
LUR:QUE	0	Some concerns	Suspected	No concerns	Major concerns	No concerns	Major concerns	Very low
LUR:ZIP	0	No concerns	Suspected	No concerns	Major concerns	No concerns	Major concerns	Very low
OLA:QUE	0	Some concerns	Suspected	No concerns	No concerns	Major concerns	Major concerns	Very low
OLA:ZIP	0	No concerns	Suspected	No concerns	Major concerns	No concerns	Major concerns	Very low
QUE:ZIP	0	No concerns	Suspected	No concerns	Major concerns	No concerns	Major concerns	Very low

3.2. Recurrence/relapse rate of depressive episodes (5 studies, 2399 patients)

League table

Results from pairwise meta-analysis are presented in the left lower half and results from network meta-analysis in the upper right half.

The boldface result indicates statistical significance.

ARI	**2.582 (1.291, 5.160)**	**2.054 (1.074, 3.930)**	0.874 (0.363, 2.102)	0.777 (0.428, 1.410)
	LUR	0.796 (0.514, 1.231)	**0.338 (0.162, 0.706)**	**0.301 (0.212, 0.429)**
		QUE	**0.425 (0.212, 0.851)**	**0.378 (0.293, 0.489)**
			ZIP	0.890 (0.467, 1.696)
0.777 (0.428, 1.410)	**0.301 (0.212, 0.429)**	**0.378 (0.293, 0.489)**	0.890 (0.467, 1.696)	PLA

Evaluation of heterogeneity and inconsistency

Between study variance (τ^2)	Heterogeneity assessment	Random-effects design-by-treatment interaction model		
		Q	df	p
0.1082	Moderate	Not estimate		

Rank probabilities

	Rank 1	Rank 2	Rank 3	Rank 4	Rank 5
ARI	0.051	0.088	0.404	0.248	0.209
LUR	0.652	0.244	0.069	0.024	0.012
QUE	0.259	0.595	0.112	0.026	0.009
ZIP	0.038	0.068	0.302	0.290	0.303
PLA	0.000	0.006	0.113	0.413	0.468

SUCRA

LUR	0.90
QUE	0.75
ARI	0.37
ZIP	0.29
PLA	0.19

Comparison-adjusted funnel plot of studies of drugs versus placebo

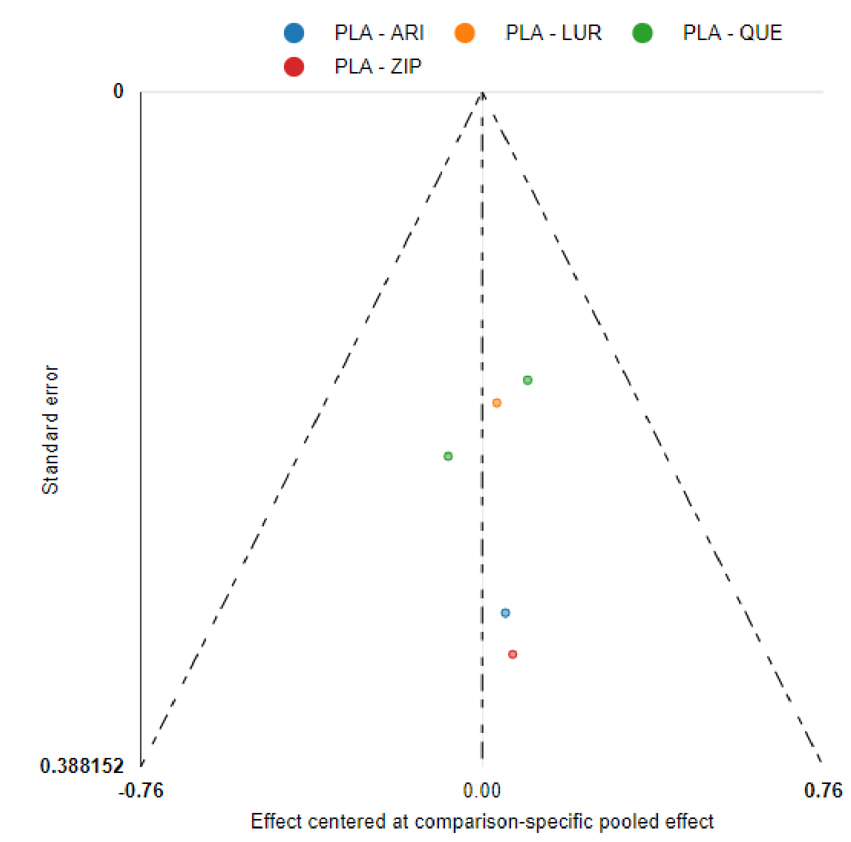

CINeMA rating

Comparison	Number of studies	Within-study bias	Reporting bias	Indirectness	Imprecision	Heterogeneity	Incoherence	Confidence rating
ARI:PLA	1	No concerns	Suspected	No concerns	Major concerns	No concerns	Major concerns	Very low
LUR:PLA	1	Some concerns	Suspected	No concerns	No concerns	Major concerns	Major concerns	Very low
PLA:QUE	2	Some concerns	Suspected	No concerns	No concerns	Major concerns	Major concerns	Very low
PLA:ZIP	1	No concerns	Suspected	No concerns	Major concerns	No concerns	Major concerns	Very low
ARI:LUR	0	No concerns	Suspected	No concerns	No concerns	Major concerns	Major concerns	Very low
ARI:QUE	0	No concerns	Suspected	No concerns	No concerns	Major concerns	Major concerns	Very low
ARI:ZIP	0	No concerns	Suspected	No concerns	Major concerns	No concerns	Major concerns	Very low
LUR:QUE	0	Some concerns	Suspected	No concerns	Major concerns	No concerns	Major concerns	Very low
LUR:ZIP	0	No concerns	Suspected	No concerns	No concerns	Major concerns	Major concerns	Very low
QUE:ZIP	0	No concerns	Suspected	No concerns	No concerns	Major concerns	Major concerns	Very low

3.3. Recurrence/relapse rate of manic/hypomanic/mixed episodes (5 studies, 2399 patients)

League table

Results from pairwise meta-analysis are presented in the left lower half and results from network meta-analysis in the upper right half.

The boldface result indicates statistical significance.

ARI	0.588 (0.219, 1.583)	0.821 (0.363, 1.857)	0.724 (0.227, 2.310)	**0.322 (0.149, 0.693)**
	LUR	1.395 (0.703, 2.769)	1.230 (0.421, 3.595)	0.547 (0.293, 1.023)
		QUE	0.882 (0.353, 2.201)	**0.392 (0.296, 0.519)**
			ZIP	0.445 (0.186, 1.063)
**0.322 (0.149, 0.693)**	0.547 (0.293, 1.023)	**0.392 (0.296, 0.519)**	0.445 (0.186, 1.063)	PLA

Evaluation of heterogeneity and inconsistency

Between study variance (τ^2)	Heterogeneity assessment	Random-effects design-by-treatment interaction model		
		Q	df	p
0.9797	Moderate	Not estimate		

Rank probabilities

	Rank 1	Rank 2	Rank 3	Rank 4	Rank 5
ARI	0.468	0.244	0.166	0.101	0.021
LUR	0.107	0.143	0.258	0.401	0.092
QUE	0.172	0.378	0.321	0.122	0.008
ZIP	0.253	0.234	0.229	0.223	0.061
PLA	0.000	0.003	0.027	0.153	0.817

SUCRA

ARI	0.75
QUE	0.69
ZIP	0.56
LUR	0.43
PLA	0.06

Comparison-adjusted funnel plot of studies of drugs versus placebo

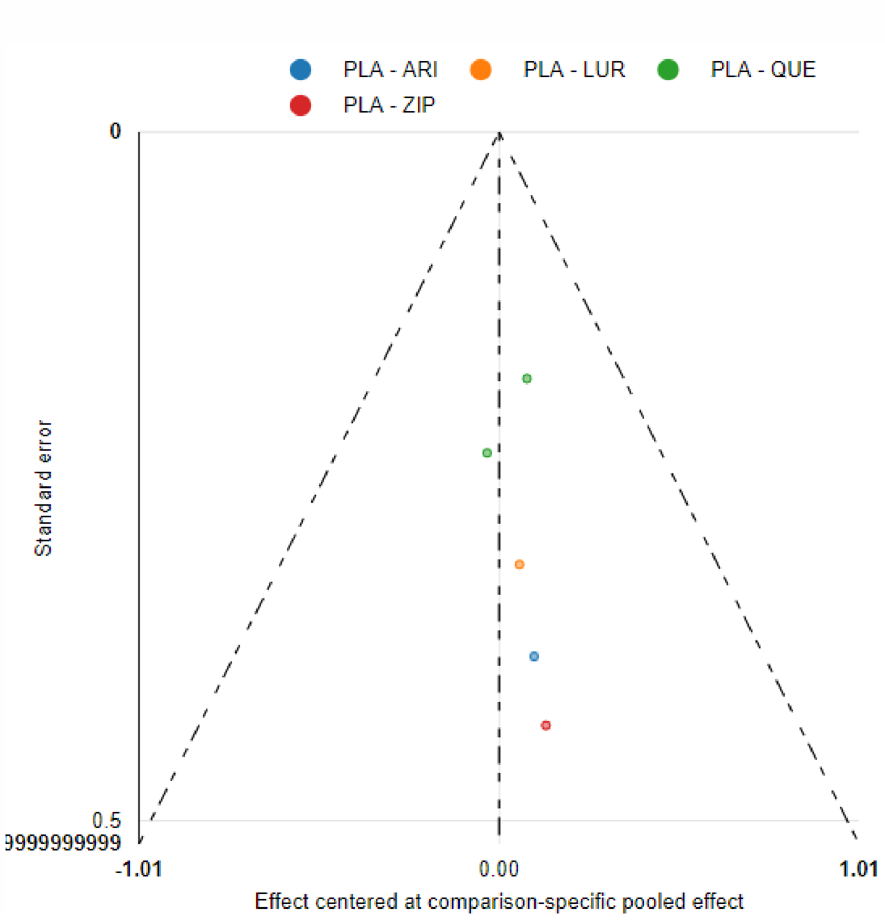

CINeMA rating

Comparison	Number of studies	Within-study bias	Reporting bias	Indirectness	Imprecision	Heterogeneity	Incoherence	Confidence rating
ARI:PLA	1	No concerns	Suspected	No concerns	No concerns	Major concerns	Major concerns	Very low
LUR:PLA	1	Some concerns	Suspected	No concerns	Major concerns	No concerns	Major concerns	Very low
PLA:QUE	2	Some concerns	Suspected	No concerns	No concerns	Major concerns	Major concerns	Very low
PLA:ZIP	1	No concerns	Suspected	No concerns	Major concerns	No concerns	Major concerns	Very low
ARI:LUR	0	No concerns	Suspected	No concerns	Major concerns	No concerns	Major concerns	Very low
ARI:QUE	0	No concerns	Suspected	No concerns	Major concerns	No concerns	Major concerns	Very low
ARI:ZIP	0	No concerns	Suspected	No concerns	Major concerns	No concerns	Major concerns	Very low
LUR:QUE	0	Some concerns	Suspected	No concerns	Major concerns	No concerns	Major concerns	Very low
LUR:ZIP	0	No concerns	Suspected	No concerns	Major concerns	No concerns	Major concerns	Very low
QUE:ZIP	0	No concerns	Suspected	No concerns	Major concerns	No concerns	Major concerns	Very low

3.4. All-cause discontinuation (6 studies, 2398 patients)

League table

Results from pairwise meta-analysis are presented in the left lower half and results from network meta-analysis in the upper right half.

The boldface result indicates statistical significance.

ARI	1.675 (0.804, 3.492)	1.067 (0.512, 2.221)	1.184 (0.631, 2.218)	1.239 (0.577, 2.663)	0.817 (0.483, 1.384)
	LUR	0.637 (0.309, 1.311)	0.706 (0.382, 1.307)	0.739 (0.348, 1.572)	**0.488 (0.293, 0.813)**
		OLA	1.109 (0.600, 2.050)	1.161 (0.547, 2.467)	0.766 (0.460, 1.276)
			QUE	1.047 (0.546, 2.009)	**0.691 (0.491, 0.972)**
				ZIP	0.660 (0.379, 1.149)
0.817 (0.483, 1.384)	**0.488 (0.293, 0.813)**	0.766 (0.460, 1.276)	**0.691 (0.491, 0.972)**	0.660 (0.379, 1.149)	PLA

Evaluation of heterogeneity and inconsistency

Between study variance (τ^2)	Heterogeneity assessment	Random-effects design-by-treatment interaction model		
		Q	df	p
0.1310	High	Not estimate		

Rank probabilities

	Rank 1	Rank 2	Rank 3	Rank 4	Rank 5	Rank 6
ARI	0.076	0.134	0.163	0.198	0.210	0.220
LUR	0.557	0.204	0.115	0.065	0.036	0.025
OLA	0.098	0.160	0.186	0.209	0.181	0.166
QUE	0.081	0.241	0.291	0.230	0.112	0.046
ZIP	0.188	0.258	0.210	0.147	0.104	0.093
PLA	0.000	0.003	0.036	0.152	0.358	0.451

SUCRA

LUR	0.80
OLA	0.76
QUE	0.58
ZIP	0.47
ARI	0.30
PLA	0.09

Comparison-adjusted funnel plot of studies of drugs versus placebo

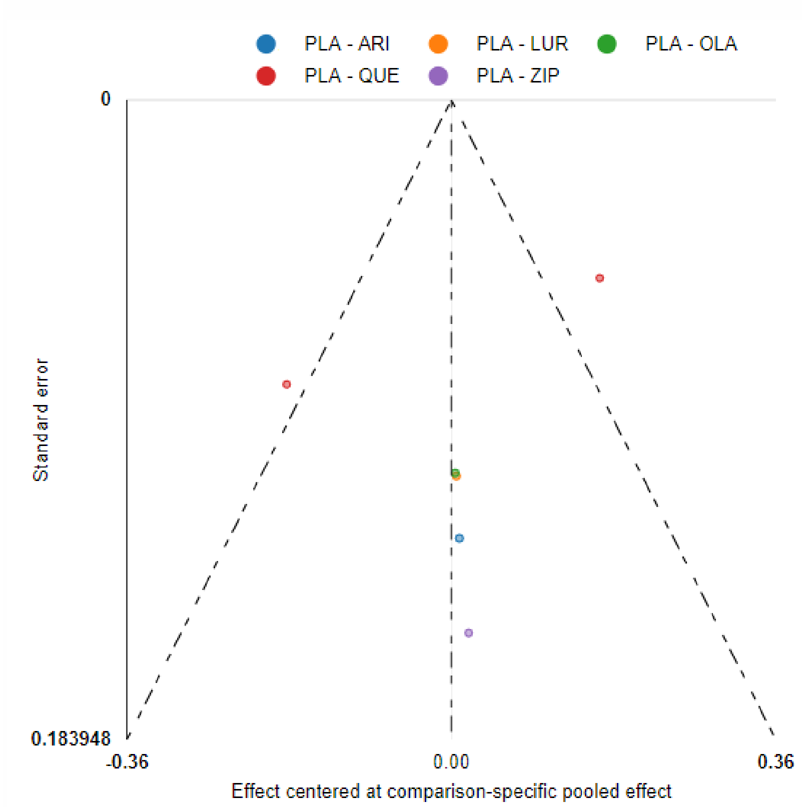

CINeMA rating

Comparison	Number of studies	Within-study bias	Reporting bias	Indirectness	Imprecision	Heterogeneity	Incoherence	Confidence rating
ARI:PLA	1	No concerns	Suspected	No concerns	Major concerns	No concerns	Major concerns	Very low
LUR:PLA	1	Some concerns	Suspected	No concerns	No concerns	Major concerns	Major concerns	Very low
OLA:PLA	1	Some concerns	Suspected	No concerns	Major concerns	No concerns	Major concerns	Very low
PLA:QUE	2	Some concerns	Suspected	No concerns	No concerns	Major concerns	Major concerns	Very low
PLA:ZIP	1	No concerns	Suspected	No concerns	Major concerns	No concerns	Major concerns	Very low
ARI:LUR	0	No concerns	Suspected	No concerns	Major concerns	No concerns	Major concerns	Very low
ARI:OLA	0	No concerns	Suspected	No concerns	Major concerns	No concerns	Major concerns	Very low
ARI:QUE	0	No concerns	Suspected	No concerns	Major concerns	No concerns	Major concerns	Very low
ARI:ZIP	0	No concerns	Suspected	No concerns	Major concerns	No concerns	Major concerns	Very low
LUR:OLA	0	Some concerns	Suspected	No concerns	Major concerns	No concerns	Major concerns	Very low
LUR:QUE	0	Some concerns	Suspected	No concerns	Major concerns	No concerns	Major concerns	Very low
LUR:ZIP	0	No concerns	Suspected	No concerns	Major concerns	No concerns	Major concerns	Very low
OLA:QUE	0	Some concerns	Suspected	No concerns	Major concerns	No concerns	Major concerns	Very low
OLA:ZIP	0	No concerns	Suspected	No concerns	Major concerns	No concerns	Major concerns	Very low
QUE:ZIP	0	No concerns	Suspected	No concerns	Major concerns	No concerns	Major concerns	Very low

3.5. Discontinuation due to adverse event (6 studies, 2398 patients)

League table

Results from pairwise meta-analysis are presented in the left lower half and results from network meta-analysis in the upper right half.

The boldface result indicates statistical significance.

ARI	1.306 (0.065, 26.177)	2.166 (0.110, 42.598)	0.599 (0.049, 7.363)	1.953 (0.109, 34.949)	1.274 (0.168, 9.644)
	LUR	1.658 (0.074, 37.159)	0.458 (0.032, 6.573)	1.495 (0.073, 30.615)	0.976 (0.107, 8.908)
		OLA	0.276 (0.020, 3.879)	0.901 (0.045, 18.112)	0.588 (0.066, 5.233)
			QUE	3.262 (0.259, 41.161)	2.129 (0.483, 9.386)
				ZIP	0.652 (0.084, 5.096)
1.274 (0.168, 9.644)	0.976 (0.107, 8.908)	0.588 (0.066, 5.233)	2.129 (0.483, 9.386)	0.652 (0.084, 5.096)	PLA

Evaluation of heterogeneity and inconsistency

Between study variance (τ^2)	Heterogeneity assessment	Random-effects design-by-treatment interaction model		
		Q	df	p
0.81	High	Not estimate		

Rank probabilities

	Rank 1	Rank 2	Rank 3	Rank 4	Rank 5	Rank 6
ARI	0.093	0.137	0.152	0.165	0.237	0.216
LUR	0.197	0.208	0.158	0.137	0.152	0.148
OLA	0.384	0.234	0.141	0.090	0.088	0.064
QUE	0.018	0.043	0.075	0.129	0.261	0.476
ZIP	0.295	0.264	0.157	0.106	0.102	0.077
PLA	0.013	0.115	0.318	0.373	0.161	0.020

SUCRA

OLA	0.71
ZIP	0.68
PLA	0.51
LUR	0.50
ARI	0.41
QUE	0.19

Comparison-adjusted funnel plot of studies of drugs versus placebo

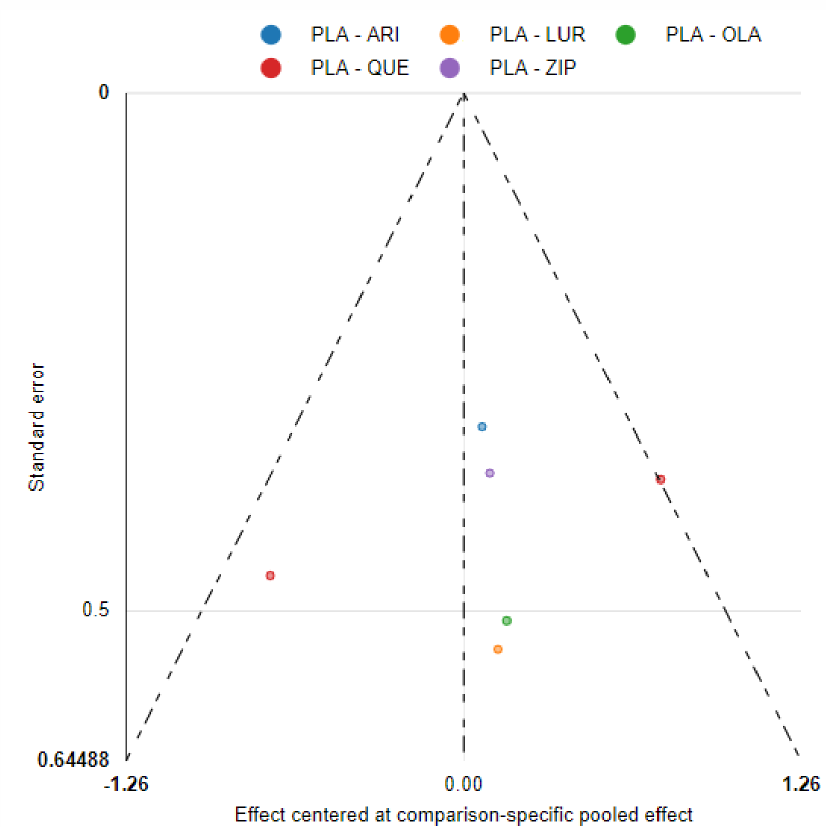

CINeMA rating

Comparison	Number of studies	Within-study bias	Reporting bias	Indirectness	Imprecision	Heterogeneity	Incoherence	Confidence rating
ARI:PLA	1	No concerns	Suspected	No concerns	Major concerns	No concerns	Major concerns	Very low
LUR:PLA	1	Some concerns	Suspected	No concerns	Major concerns	No concerns	Major concerns	Very low
OLA:PLA	1	Some concerns	Suspected	No concerns	Major concerns	No concerns	Major concerns	Very low
PLA:QUE	2	Some concerns	Suspected	No concerns	Major concerns	No concerns	Major concerns	Very low
PLA:ZIP	1	No concerns	Suspected	No concerns	Major concerns	No concerns	Major concerns	Very low
ARI:LUR	0	No concerns	Suspected	No concerns	Major concerns	No concerns	Major concerns	Very low
ARI:OLA	0	No concerns	Suspected	No concerns	Major concerns	No concerns	Major concerns	Very low
ARI:QUE	0	No concerns	Suspected	No concerns	Major concerns	No concerns	Major concerns	Very low
ARI:ZIP	0	No concerns	Suspected	No concerns	Major concerns	No concerns	Major concerns	Very low
LUR:OLA	0	Some concerns	Suspected	No concerns	Major concerns	No concerns	Major concerns	Very low
LUR:QUE	0	Some concerns	Suspected	No concerns	Major concerns	No concerns	Major concerns	Very low
LUR:ZIP	0	No concerns	Suspected	No concerns	Major concerns	No concerns	Major concerns	Very low
OLA:QUE	0	Some concerns	Suspected	No concerns	Major concerns	No concerns	Major concerns	Very low
OLA:ZIP	0	No concerns	Suspected	No concerns	Major concerns	No concerns	Major concerns	Very low
QUE:ZIP	0	No concerns	Suspected	No concerns	Major concerns	No concerns	Major concerns	Very low

Supplementary Appendix 2.6. Mortality

	Intervention	n	Total patients	Intervention	n	Total patients
Bowden 2010	ZIP+LIT/VAL	0	127	LIT/VAL	0	112
Calabrese 2017	LUR+LIT/VAL	0	246	LIT/VAL	0	150
Marcus 2011	ARI+LIT/VAL	1	168	LIT/VAL	1	169
Suppes 2009	QUE+LIT/VAL	1	310	LIT/VAL	0	313
Tohen 2004	OLA+LIT/VAL			LIT/VAL		
Vieta 2008	QUE+LIT/VAL	1	336	LIT/VAL	3	367

Supplementary Appendix 2.7. Completed suicide

	Intervention	n	Total patients	Intervention	n	Total patients
Bowden 2010	ZIP+LIT/VAL	0	127	LIT/VAL	0	112
Calabrese 2017	LUR+LIT/VAL	0	246	LIT/VAL	0	150
Marcus 2011	ARI+LIT/VAL	1	168	LIT/VAL	0	169
Suppes 2009	QUE+LIT/VAL	1	310	LIT/VAL	0	313
Tohen 2004	OLA+LIT/VAL			LIT/VAL		
Vieta 2008	QUE+LIT/VAL	1	336	LIT/VAL	1	367

3.8. Extrapyramidal symptoms (6 studies, 2398 patients)

League table

Results from pairwise meta-analysis are presented in the left lower half and results from network meta-analysis in the upper right half.

The boldface result indicates statistical significance.

ARI	1.732 (0.464, 6.465)	1.527 (0.300, 7.783)	2.362 (0.704, 7.915)	1.426 (0.278, 7.318)	2.515 (0.805, 7.861)
	LUR	0.881 (0.231, 3.359)	1.363 (0.628, 2.957)	0.823 (0.214, 3.162)	1.452 (0.750, 2.809)
		OLA	1.547 (0.451, 5.302)	0.934 (0.179, 4.873)	1.647 (0.515, 5.272)
			QUE	0.604 (0.175, 2.089)	1.065 (0.710, 1.597)
				ZIP	1.764 (0.546, 5.700)
2.515 (0.805, 7.861)	1.452 (0.750, 2.809)	1.647 (0.515, 5.272)	1.065 (0.710, 1.597)	1.764 (0.546, 5.700)	PLA

Evaluation of heterogeneity and inconsistency

Between study variance (τ^2)	Heterogeneity assessment	Random-effects design-by-treatment interaction model		
		Q	df	p
0.0961	Moderate	Not estimate		

Rank probabilities

	Rank 1	Rank 2	Rank 3	Rank 4	Rank 5	Rank 6
ARI	0.063	0.060	0.072	0.129	0.241	0.434
LUR	0.122	0.114	0.162	0.238	0.235	0.130
OLA	0.218	0.104	0.115	0.173	0.206	0.184
QUE	0.250	0.248	0.221	0.171	0.086	0.024
ZIP	0.180	0.095	0.126	0.164	0.209	0.226
PLA	0.167	0.379	0.304	0.126	0.023	0.002

SUCRA

PLA	0.78
QUE	0.70
LUR	0.49
OLA	0.41
ZIP	0.40
ARI	0.23

Comparison-adjusted funnel plot of studies of drugs versus placebo

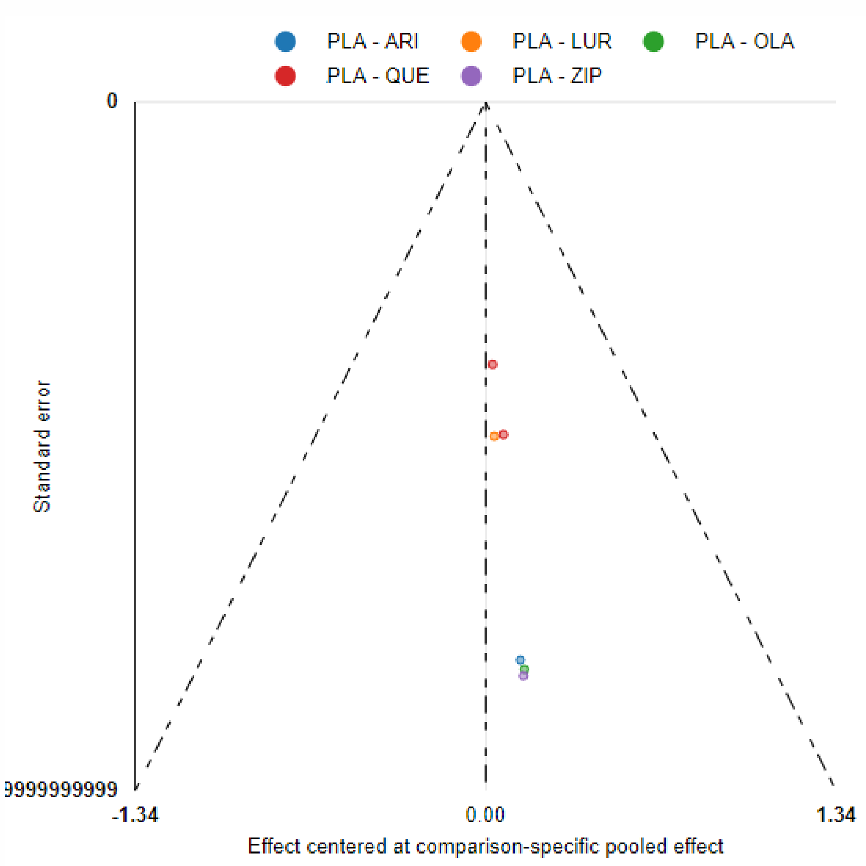

CINeMA rating

Comparison	Number of studies	Within-study bias	Reporting bias	Indirectness	Imprecision	Heterogeneity	Incoherence	Confidence rating
ARI:PLA	1	No concerns	Suspected	No concerns	Major concerns	No concerns	Major concerns	Very low
LUR:PLA	1	Some concerns	Suspected	No concerns	Major concerns	No concerns	Major concerns	Very low
OLA:PLA	1	Some concerns	Suspected	No concerns	Major concerns	No concerns	Major concerns	Very low
PLA:QUE	2	Some concerns	Suspected	No concerns	Major concerns	No concerns	Major concerns	Very low
PLA:ZIP	1	No concerns	Suspected	No concerns	Major concerns	No concerns	Major concerns	Very low
ARI:LUR	0	No concerns	Suspected	No concerns	Major concerns	No concerns	Major concerns	Very low
ARI:OLA	0	No concerns	Suspected	No concerns	Major concerns	No concerns	Major concerns	Very low
ARI:QUE	0	No concerns	Suspected	No concerns	Major concerns	No concerns	Major concerns	Very low
ARI:ZIP	0	No concerns	Suspected	No concerns	Major concerns	No concerns	Major concerns	Very low
LUR:OLA	0	Some concerns	Suspected	No concerns	Major concerns	No concerns	Major concerns	Very low
LUR:QUE	0	Some concerns	Suspected	No concerns	Major concerns	No concerns	Major concerns	Very low
LUR:ZIP	0	No concerns	Suspected	No concerns	Major concerns	No concerns	Major concerns	Very low
OLA:QUE	0	Some concerns	Suspected	No concerns	Major concerns	No concerns	Major concerns	Very low
OLA:ZIP	0	No concerns	Suspected	No concerns	Major concerns	No concerns	Major concerns	Very low
QUE:ZIP	0	No concerns	Suspected	No concerns	Major concerns	No concerns	Major concerns	Very low

3.9. Somnolence (3 studies, 1425 patients)

League table

Results from pairwise meta-analysis are presented in the left lower half and results from network meta-analysis in the upper right half.

The boldface result indicates statistical significance.

OLA	0.591 (0.097, 3.611)	2.353 (0.524, 10.568)
	QUE	**3.983 (1.450, 10.943)**
2.353 (0.524, 10.568)	**3.983 (1.450, 10.943)**	PLA

Evaluation of heterogeneity and inconsistency

Between study variance (τ^2)	Heterogeneity assessment	Random-effects design-by-treatment interaction model		
		Q	df	p
0.8910	High	Not estimate		

Rank probabilities

	Rank 1	Rank 2	Rank 3
OLA	0.218	0.482	0.300
QUE	0.036	0.278	0.686
PLA	0.747	0.240	0.014

SUCRA

PLA	0.90
OLA	0.41
QUE	0.19

Comparison-adjusted funnel plot of studies of drugs versus placebo

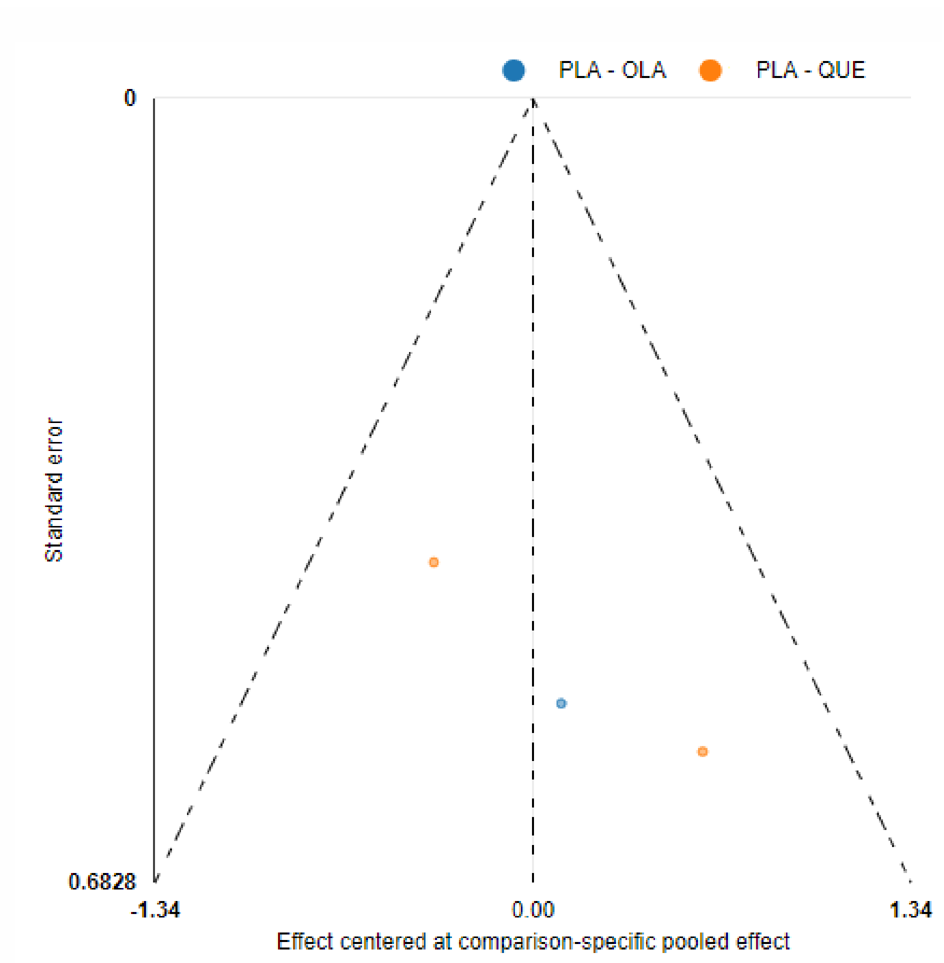

CINeMA rating

Comparison	Number of studies	Within-study bias	Reporting bias	Indirectness	Imprecision	Heterogeneity	Incoherence	Confidence rating
OLA:PLA	1	Some concerns	Suspected	No concerns	Major concerns	No concerns	Major concerns	Very low
PLA:QUE	2	Some concerns	Suspected	No concerns	No concerns	Major concerns	Major concerns	Very low
OLA:QUE	0	Some concerns	Suspected	No concerns	Major concerns	No concerns	Major concerns	Very low

3.10. Insomnia (6 studies, 2398 patients)

League table

Results from pairwise meta-analysis are presented in the left lower half and results from network meta-analysis in the upper right half.

The boldface result indicates statistical significance.

ARI	0.990 (0.244, 4.016)	3.908 (0.621, 24.611)	1.501 (0.486, 4.636)	1.100 (0.256, 4.733)	0.566 (0.211, 1.518)
	LUR	3.948 (0.624, 24.963)	1.516 (0.487, 4.714)	1.111 (0.257, 4.806)	0.572 (0.212, 1.544)
		OLA	0.384 (0.074, 1.993)	0.281 (0.043, 1.862)	**0.145 (0.031, 0.684)**
			QUE	0.733 (0.219, 2.450)	**0.377 (0.218, 0.652)**
				ZIP	0.514 (0.176, 1.508)
0.566 (0.211, 1.518)	0.572 (0.212, 1.544)	**0.145 (0.031, 0.684)**	**0.377 (0.218, 0.652)**	0.514 (0.176, 1.508)	PLA

Evaluation of heterogeneity and inconsistency

Between study variance (τ^2)	Heterogeneity assessment	Random-effects design-by-treatment interaction model		
		Q	df	p
0.4264	High	Not estimate		

Rank probabilities

	Rank 1	Rank 2	Rank 3	Rank 4	Rank 5	Rank 6
ARI	0.059	0.173	0.186	0.226	0.204	0.152
LUR	0.064	0.164	0.197	0.217	0.206	0.152
OLA	0.711	0.136	0.073	0.041	0.022	0.018
QUE	0.078	0.309	0.319	0.192	0.080	0.024
ZIP	0.089	0.216	0.201	0.197	0.173	0.124
PLA	0.000	0.003	0.024	0.127	0.316	0.530

SUCRA

OLA	0.89
QUE	0.63
ZIP	0.48
ARI	0.43
LUR	0.42
PLA	0.15

Comparison-adjusted funnel plot of studies of drugs versus placebo

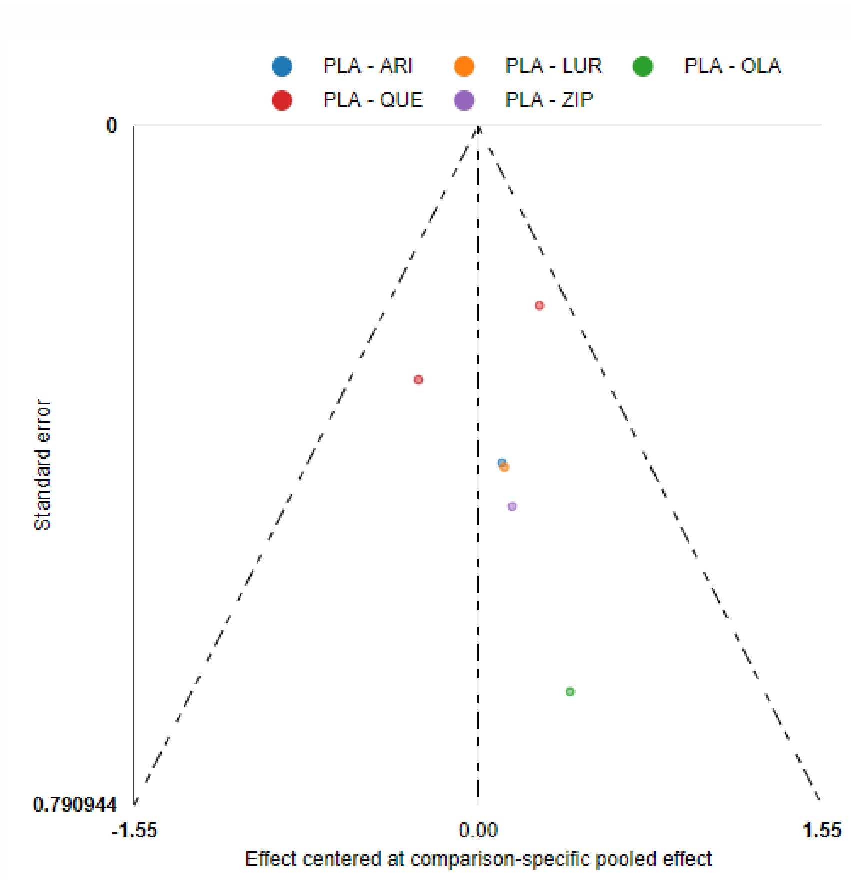

CINeMA rating

Comparison	Number of studies	Within-study bias	Reporting bias	Indirectness	Imprecision	Heterogeneity	Incoherence	Confidence rating
ARI:PLA	1	No concerns	Suspected	No concerns	Major concerns	No concerns	Major concerns	Very low
LUR:PLA	1	Some concerns	Suspected	No concerns	Major concerns	No concerns	Major concerns	Very low
OLA:PLA	1	Some concerns	Suspected	No concerns	No concerns	Major concerns	Major concerns	Very low
PLA:QUE	2	Some concerns	Suspected	No concerns	No concerns	Major concerns	Major concerns	Very low
PLA:ZIP	1	No concerns	Suspected	No concerns	Major concerns	No concerns	Major concerns	Very low
ARI:LUR	0	No concerns	Suspected	No concerns	Major concerns	No concerns	Major concerns	Very low
ARI:OLA	0	No concerns	Suspected	No concerns	Major concerns	No concerns	Major concerns	Very low
ARI:QUE	0	No concerns	Suspected	No concerns	Major concerns	No concerns	Major concerns	Very low
ARI:ZIP	0	No concerns	Suspected	No concerns	Major concerns	No concerns	Major concerns	Very low
LUR:OLA	0	Some concerns	Suspected	No concerns	Major concerns	No concerns	Major concerns	Very low
LUR:QUE	0	Some concerns	Suspected	No concerns	Major concerns	No concerns	Major concerns	Very low
LUR:ZIP	0	No concerns	Suspected	No concerns	Major concerns	No concerns	Major concerns	Very low
OLA:QUE	0	Some concerns	Suspected	No concerns	Major concerns	No concerns	Major concerns	Very low
OLA:ZIP	0	No concerns	Suspected	No concerns	Major concerns	No concerns	Major concerns	Very low
QUE:ZIP	0	No concerns	Suspected	No concerns	Major concerns	No concerns	Major concerns	Very low

3.11. Increased weight (6 studies)

League table

Results from pairwise meta-analysis are presented in the left lower half and results from network meta-analysis in the upper right half.

The boldface result indicates statistical significance.

ARI	0.569 (0.225, 1.438)	**0.263 (0.071, 0.975)**	**0.348 (0.164, 0.738)**	1.124 (0.339, 3.730)	1.157 (0.661, 2.025)
	LUR	0.463 (0.115, 1.866)	0.611 (0.250, 1.492)	1.975 (0.543, 7.192)	2.033 (0.971, 4.253)
		OLA	1.320 (0.365, 4.773)	4.269 (0.872, 20.907)	**4.392 (1.346, 14.336)**
			QUE	3.234 (1.000, 10.457)	**3.327 (2.013, 5.501)**
				ZIP	1.029 (0.356, 2.971)
1.157 (0.661, 2.025)	2.033 (0.971, 4.253)	**4.392 (1.346, 14.336)**	**3.327 (2.013, 5.501)**	1.029 (0.356, 2.971)	PLA

Evaluation of heterogeneity and inconsistency

Between study variance (τ^2)	Heterogeneity assessment	Random-effects design-by-treatment interaction model		
		Q	df	p
0.1998	High	Not estimate		

Rank probabilities

	Rank 1	Rank 2	Rank 3	Rank 4	Rank 5	Rank 6
ARI	0.252	0.233	0.276	0.148	0.064	0.028
LUR	0.073	0.094	0.156	0.363	0.209	0.105
OLA	0.021	0.031	0.059	0.141	0.245	0.503
QUE	0.004	0.017	0.051	0.171	0.420	0.337
ZIP	0.446	0.170	0.184	0.117	0.056	0.028
PLA	0.204	0.455	0.275	0.060	0.006	0.000

SUCRA

PLA	0.79
ZIP	0.72
ARI	0.68
LUR	0.44
QUE	0.23
OLA	0.13

Comparison-adjusted funnel plot of studies of drugs versus placebo

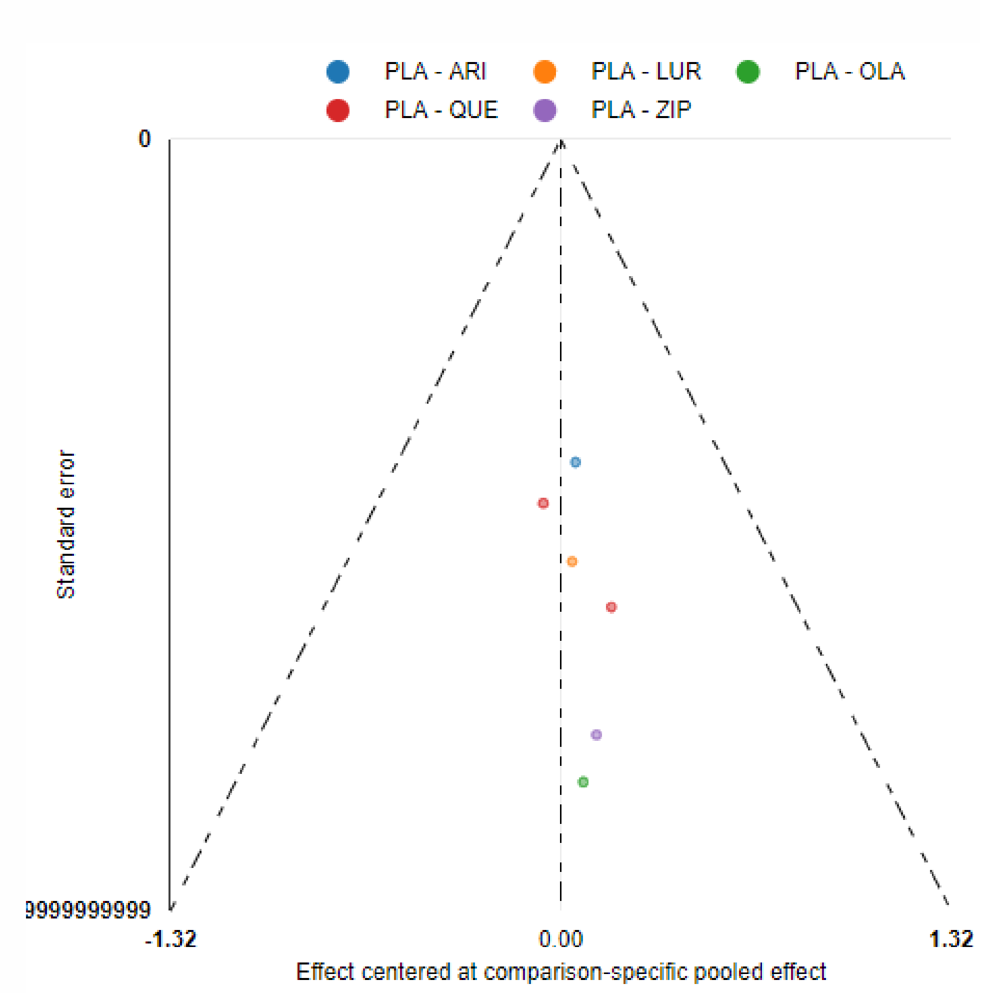

CINeMA rating

Comparison	Number of studies	Within-study bias	Reporting bias	Indirectness	Imprecision	Heterogeneity	Incoherence	Confidence rating
ARI:PLA	1	No concerns	Suspected	No concerns	Major concerns	No concerns	Major concerns	Very low
LUR:PLA	1	Some concerns	Suspected	No concerns	Major concerns	No concerns	Major concerns	Very low
OLA:PLA	1	Some concerns	Suspected	No concerns	No concerns	Major concerns	Major concerns	Very low
PLA:QUE	2	Some concerns	Suspected	No concerns	No concerns	Major concerns	Major concerns	Very low
PLA:ZIP	1	No concerns	Suspected	No concerns	Major concerns	No concerns	Major concerns	Very low
ARI:LUR	0	No concerns	Suspected	No concerns	Major concerns	No concerns	Major concerns	Very low
ARI:OLA	0	No concerns	Suspected	No concerns	No concerns	Major concerns	Major concerns	Very low
ARI:QUE	0	No concerns	Suspected	No concerns	No concerns	Major concerns	Major concerns	Very low
ARI:ZIP	0	No concerns	Suspected	No concerns	Major concerns	No concerns	Major concerns	Very low
LUR:OLA	0	Some concerns	Suspected	No concerns	Major concerns	No concerns	Major concerns	Very low
LUR:QUE	0	Some concerns	Suspected	No concerns	Major concerns	No concerns	Major concerns	Very low
LUR:ZIP	0	No concerns	Suspected	No concerns	Major concerns	No concerns	Major concerns	Very low
OLA:QUE	0	Some concerns	Suspected	No concerns	Major concerns	No concerns	Major concerns	Very low
OLA:ZIP	0	No concerns	Suspected	No concerns	Major concerns	No concerns	Major concerns	Very low
QUE:ZIP	0	No concerns	Suspected	No concerns	Major concerns	No concerns	Major concerns	Very low
